# The predictive accuracy of cardiovascular disease risk prediction tools in inflammatory arthritis and psoriasis: an observational validation study using the Clinical Practice Research Datalink

**DOI:** 10.1093/rheumatology/kead610

**Published:** 2023-11-15

**Authors:** David M Hughes, Jose Ignacio Cuitun Coronado, Pieta Schofield, Zenas Z N Yiu, Sizheng Steven Zhao

**Affiliations:** Department of Health Data Science, University of Liverpool, Liverpool, UK; Department of Population Health Sciences, University of Bristol, Bristol, UK; Institute of Population Health, University of Liverpool, Liverpool, UK; Centre for Dermatology Research, Northern Care Alliance NHS Foundation Trust, The University of Manchester, Manchester Academic Health Science Centre, National Institute for Health and Care Research Manchester Biomedical Research Centre, Manchester, UK; Centre for Epidemiology Versus Arthritis, Division of Musculoskeletal and Dermatological Science, School of Biological Sciences, Faculty of Biological Medicine and Health, The University of Manchester, Manchester Academic Health Science Centre, Manchester, UK

**Keywords:** cardiovascular risk prediction, psoriatic arthritis, ankylosing spondylitis, rheumatoid arthritis, psoriasis

## Abstract

**Objectives:**

Cardiovascular risk prediction tools developed for the general population often underperform for individuals with RA, and their predictive accuracy are unclear for other inflammatory conditions that also have increased cardiovascular risk. We investigated the performance of QRISK-3, the Framingham Risk Score (FRS) and the Reynolds Risk Score (RRS) in RA, psoriatic disease (PsA and psoriasis) and AS. We considered OA as a non-inflammatory comparator.

**Methods:**

We utilized primary care records from the Clinical Practice Research Datalink (CPRD) Aurum database to identify individuals with each condition and calculated 10-year cardiovascular risk using each prediction tool. The discrimination and calibration of each tool was assessed for each disease.

**Results:**

The time-dependent area under the curve (AUC) for QRISK3 was 0.752 for RA (95% CI 0.734–0.777), 0.794 for AS (95% CI 0.764–0.812), 0.764 for PsA (95% CI 0.741–0.791), 0.815 for psoriasis (95% CI 0.789–0.835) and 0.698 for OA (95% CI 0.670–0.717), indicating reasonably good predictive performance. The AUCs for the FRS were similar, and slightly lower for the RRS. The FRS was reasonably well calibrated for each condition but underpredicted risk for patients with RA. The RRS tended to underpredict CVD risk, while QRISK3 overpredicted CVD risk, especially for the most high-risk individuals.

**Conclusion:**

CVD risk for individuals with RA, AS and psoriatic disease was generally less accurately predicted using each of the three CVD risk prediction tools than the reported accuracies in the original publications. Individuals with OA also had less accurate predictions, suggesting inflammation is not the sole reason for underperformance. Disease-specific risk prediction tools may be required.

Rheumatology key messages

- CVD risk for RA, AS or psoriatic disease is less accurately predicted using each of the three risk prediction tools than the reported accuracies in the original publications.
- QRISK3 overestimates CVD risk in RA, AS and psoriatic disease.
- Underperformance of CVD risk tools are often attributed to inflammatory burden, yet our results showed equally limited performance in an OA cohort.

## Introduction

Cardiovascular diseases (CVDs) are the leading cause of premature death and a major cause of disability. CVD risk is increased among people with chronic inflammatory conditions, such as RA, AS or psoriatic disease (namely PsA and psoriasis), compared with the general population [1, 2]. Screening for CVDs using risk models gives an assessment of an individual’s risk, which can assist clinical judgement regarding preventative responses or treatment strategies for individuals deemed to be at higher risk. Many CVD-risk prediction models have been developed, including the Framingham Risk Score (FRS) [3], QRisk3 [4] and the Reynolds Risk Score (RRS) [5, 6]. 

A number of studies have shown that CVD risk tools developed for the general population often underestimate CVD risk in patients with RA [1, 7]. The EULAR 2017 recommendations for cardiovascular risk management acknowledged this and suggested multiplying any risk calculated using general population tools by 1.5 for patients with RA [8]. The EULAR multiplier for RA has been used as a justification for applying a 1.5 multiplication factor for patients with psoriasis with over 10% body surface area involvement or who qualify for systemic therapy or phototherapy [9].

However, these proposals are not supported by substantial evidence, leading to at least two attempts to develop an RA-specific CVD risk calculator. The Expanded cardiovascular Risk prediction Score for RA patients (ERS-RA) has shown good performance in both the US Corrona registry [10] and in a Swedish cohort [11]. However, the ERS-RA did not perform well in a large multinational cohort of RA patients [12]. Crowson *et al.* also developed a RA-specific CVD risk calculator, but this had no better discriminatory ability than the general population risk calculators [13]. Although it is known that general population CVD risk calculators generally underestimate CVD risk in RA, RA-specific CVD risk tools that outperform general population tools have so far proved elusive.

Inflammation is proposed as a driver of increased CVD risk [13, 14]. CVD risk prediction is less well studied in other inflammatory diseases such as AS or psoriatic disease [1], in which CVD risk is also higher compared with the general population [2, 15]. Dedicated study of cardiovascular risk prediction in these diseases is needed, since pathophysiology, systemic inflammatory burden, and comorbidities relevant for cardiovascular risk are recognized to differ.

We assessed the performance of three commonly used general population CVD risk prediction tools in RA, AS and psoriatic disease. We included RA to validate the prior research and to contextualize CVD risk prediction in AS and psoriatic disease, the latter comprising the pathological spectrum of psoriasis and PsA.

## Methods

### Data source

We used data from the Clinical Practice Research Datalink (CPRD). This is a large database of anonymized patient data from general practitioner (GP) practices in England representative of the general UK population. CPRD contains data for over 60 million patients, with over 18 million currently registered patients. We also linked the individuals’ GP records in CPRD to hospital episode statistics (HES). This provided additional details of the diagnosis of CVD outcomes during hospital admissions.

The study protocol and access to the CPRD data was approved by the Independent Scientific Advisory committee (Study protocol 21_000490). The study description can be found at https://cprd.com/protocol/cardiovascular-risk-prediction-rheumatic-diseases

### Disease definitions

We considered three inflammatory arthritides for the primary analysis, namely, RA, AS and PsA. We included psoriasis because it is also part of the psoriatic disease spectrum. Since we hypothesized that inflammation is a key reason for general population risk prediction tools underperforming, we also included OA—not typically considered an inflammatory disease—as a comparative outcome. We chose OA, rather than a general population control, on the premise that this group has more comparable functional limitation with that of the inflammatory arthritides. The OA group was included not as a strict control group for other disease groups, but to see whether previously reported poor performance of CVD risk tools was limited to inflammatory conditions.

Individuals were identified as having one of the five conditions considered in this paper (RA, AS, PsA, psoriasis or OA) if they had a at least one Read code for the condition (code lists in the Supplementary Material, available at *Rheumatology* online). The date of the first recorded code for each condition was treated as the index date from which the CVD risk was calculated. Individuals could belong to more than one of the disease groups, for example, if they had both PsA and psoriasis. This was because we were not directly comparing between groups, but simply assessing the performance of CVD risk prediction within each disease. However, we did exclude from the OA group individuals with any of the other conditions.

### Definitions of cardiovascular outcome

Cardiovascular outcomes were determined using Read codes. The definition of CVD outcome differs slightly for each of QRISK3, FRS and RRS, but broadly speaking includes coronary heart disease, cerebrovascular events, peripheral artery disease, and heart failure. We defined three CVD outcomes, specific to the definition of each risk prediction tool. We used ICD-10 codes when linking the CPRD data to the HES data to identify additional CVD diagnoses. For QRISK-3, these ICD-10 codes followed exactly those reported in the initial publication [4]. All Read code and ICD-10 code lists used in this analysis are in the supplementary material, available at *Rheumatology* online.

We excluded individuals on statins, and those who had a record for the relevant CVD event for each risk tool before the index date (the time at which we made a prediction of 10-year risk using the risk prediction tool). 

### Risk prediction variables

All risk prediction scores were calculated using the published risk prediction algorithms for each model. Table 1 shows the risk factors included in each model. Relevant code lists for each predictor variable are included in the supplementary material. For blood measurements, height, weight and BMI, the closest observation prior to the index date was used, following the approach for the development of QRISK3. For medications, following the QRISK3 algorithm, two prescriptions prior to the index date with at least one within the preceding 28 days was used to determine that an individual was taking the medication. For conditions used as predictors of CVD risk, we defined an individual as having the condition if they had a relevant Read code prior to the index date. Individuals known to have diabetes but with missing type were assumed to have type 2 diabetes, while individuals with codes for both diabetes types were assumed to have type 1 diabetes. Both the RRS and the FRS consider smoking as a binary variable (current smoker *vs* non-smoker), while the QRISK3 tool considers five categories (non-smoker, ex-smoker, light smoker, moderate smoker or heavy smoker). Total cholesterol, high-density lipoprotein (HDL) cholesterol, and systolic blood pressure values were log-transformed for use in the FRS and RRS, as were CRP scores for RRS. The QRISK3 tool includes fractional polynomial terms for age and BMI, as well as a large number of terms for interactions between individual risk factors. 

**Table 1.** Summary of the risk factors included in QRISK3, the Framingham Risk Score and the Reynolds Risk Score

Risk factor	QRISK3	Framingham Risk Score	Reynolds Risk Score
Sex	✓	✓	✓
Age (years)	✓	✓	✓
BMI	✓		
Total cholesterol: HDL Cholesterol Ratio	✓		
Total cholesterol		✓	✓
HDL cholesterol		✓	✓
Systolic blood pressure	✓	✓	✓
S.D. of systolic blood pressure	✓		
Ethnicity	✓		
Smoking status	✓	✓	✓
Family history of coronary heart disease	✓		
Family history of premature myocardial infarction			✓
Diabetes		✓	✓
Type 1 diabetes	✓		
Type 2 diabetes	✓		
Treated hypertension	✓	✓	
RA	✓		
Atrial fibrillation	✓		
Chronic kidney disease (stages 3, 4 or 5)	✓		
Migraine	✓		
CS use	✓		
SLE	✓		
Atypical antipsychotic use	✓		
Severe mental illness	✓		
Erectile dysfunction or treatments	✓		
Townsend Score	✓		
High-sensitivity CRP			✓
HbA1c			✓

HDL: high-density lipoprotein.

### Statistical methods

Data for each individual were used to calculate each risk prediction score. Follow-up time ended at the earliest date of CPRD extraction (31 October 2021), date of incident CVD, date of death, or 10 years, to match the prediction-window of the risk prediction tools.

Multiple imputation with chained equations was used for missing continuous risk predictors or smoking status data. We imputed continuous variables using predictive mean matching, and categorical/binary variables using polytomous/logistic regression. All predictor variables (including interaction terms) for the relevant risk score were used as predictors for the imputations. Five imputed datasets were calculated (to balance computational burden and robustness of results), and the results were combined using Rubin’s Rules. Missing Townsend Scores were not imputed [4, 16].

Calibration of each risk prediction tool was assessed by comparing the observed and predicted risks in deciles of predicted risk for each disease group (RA, AS, PsA, psoriasis or OA). We additionally assessed calibration in different age groups (grouped, similarly to the QRISK3 tool, as <40, 40–59 and 60+).

Discrimination was assessed using time-dependent area-under-the-curve (AUC), sensitivity, specificity, positive predictive value (PPV) and negative predictive value (NPV) [17]. This time-dependent approach uses inverse probability of censoring weights, to account for censoring at 10 years. We report time-dependent sensitivity, specificity, PPV and NPV at the 10% and 20% thresholds of predicted risk. These broadly represent the thresholds of low-to-intermediate and intermediate-to-high risk of CVD disease [7].

We assessed a hypothetical group of 1000 individuals to describe the potential implications of changes in classification accuracy in comparison with the original tools. We extracted details of the sensitivity and specificity of the QRISK2 and FRS from a previous external validation of performance in the general population from The Health Improvement Network (THIN) [18]. For each disease, we used the prevalence in each disease cohort in CPRD for our study.

As a sensitivity analysis, we repeated all the analyses but this time at least two codes for diagnosis of each disease were required, which allowed greater confidence in the accuracy of the diagnosis.

## Results

The numbers of individuals eligible for each risk prediction tool varied due to the different CVD definitions. A summary of the cohort details for the QRISK cohort is shown in Table 2. Full details are shown in Supplementary Tables S1–S3, available at *Rheumatology* online for the QRISK cohort, the FRS and the RRS, respectively. The QRISK3 algorithm was applied to 91 750 individuals with RA (mean age 56.9 years, 29.7% male), 42 306 AS (mean age 47.3, 40.8% male), 26 375 individuals with PsA (mean age 47.2, 48.1% male), 390 664 individuals with psoriasis (mean age 42.0, 45.2% male) and 1 079 815 individuals with OA (mean age 62.79, 38.0% male). The median follow-up time in the QRISK cohort was 8.7 years (interquartile range 4.2–14.9 years).

**Table 2.** Baseline characteristics of patients in the CPRD suitable for calculating QRISK3 score

		RA			AS			PsA			Psoriasis			OA		
Risk factor		No CVD	CVD	*P*	No CVD	CVD	*P*	No CVD	CVD	*P*	No CVD	CVD	*P*	No CVD	CVD	*P*
Total	*N* (%)	81181 (88.5)	10569 (11.5)		39485 (93.3)	2821 (6.7)		24573 (93.2)	1802 (6.8)		371586 (95.1)	19078 (4.9)		844752 (87.5)	120505 (12.5)	
Sex	Male	23 265 (28.7)	3974 (37.6)	<0.001	15 910 (40.3)	1340 (47.5)	<0.001	11 677 (47.5)	1026 (56.9)	<0.001	166 082 (44.7)	10 487 (55.0)	<0.001	315 701 (37.4)	53 074 (44.0)	<0.001
Age	Mean (S.D.)	55.7 (15.1)	66.8 (11.8)	<0.001	46.2 (15.4)	62.7 (13.4)	<0.001	46.4 (13.4)	58.9 (11.8)	<0.001	41.0 (16.7)	62.2 (13.5)	<0.001	62.0 (12.4)	69.5 (11.1)	<0.001
Systolic blood pressure (mmHg)	Mean (S.D.)	132.2 (110.3)	140.0 (23.2)	<0.001	126.8 (56.4)	137.2 (19.9)	<0.001	129.2 (19.1)	137.7 (18.7)	<0.001	126.2 (58.5)	139.0 (22.3)	<0.001	135.1 (285.5)	140.4 (45.8)	<0.001
Total cholesterol (mg/dl)	Mean (S.D.)	202.7 (144.2)	206.6 (44.1)	0.051	200.8 (40.7)	209.3 (44.8)	<0.001	204.3 (86.2)	209.5 (50.4)	0.06	203.2 (81.0)	212.4 (55.8)	<0.001	204.5 (404.1)	203.3 (68.2)	0.406
High density lipoprotein cholesterol (mg/dl)	Mean (S.D.)	57.7 (55.8)	55.5 (17.7)	0.01	57.2 (41.1)	55.4 (17.0)	0.139	54.3 (44.4)	51.8 (17.6)	0.103	56.3 (33.9)	54.3 (18.0)	<0.001	58.4 (42.2)	56.0 (31.2)	<0.001
Smoking	Never	29 661 (36.5)	2940 (27.8)	<0.001	15 741 (39.9)	920 (32.6)	<0.001	9537 (38.8)	528 (29.3)	<0.001	140 788 (37.9)	5129 (26.9)	<0.001	384 992 (45.6)	45 325 (37.6)	<0.001
	Former	22 181 (27.3)	3408 (32.2)		9746 (24.7)	880 (31.2)		6778 (27.6)	595 (33.0)		83 045 (22.3)	6155 (32.3)		253 433 (30.0)	41 429 (34.4)	
	Light	15 981 (19.7)	2033 (19.2)		8046 (20.4)	578 (20.5)		5066 (20.6)	398 (22.1)		86 232 (23.2)	4675 (24.5)		131 570 (15.6)	18 908 (15.7)	
	Medium	1569 (1.9)	222 (2.1)		859 (2.2)	68 (2.4)		425 (1.7)	40 (2.2)		8826 (2.4)	491 (2.6)		8718 (1.0)	1445 (1.2)	
	Heavy	942 (1.2)	156 (1.5)		478 (1.2)	47 (1.7)		238 (1.0)	40 (2.2)		4432 (1.2)	421 (2.2)		5309 (0.6)	952 (0.8)	
Diabetes		5233 (6.4)	1177 (11.1)	<0.001	1612 (4.1)	308 (10.9)	<0.001	1385 (5.6)	206 (11.4)	<0.001	12 917 (3.5)	1937 (10.2)	<0.001	87 546 (10.4)	17 951 (14.9)	<0.001
Anti-hypertensive medication		8684 (10.7)	2361 (22.3)	<0.001	2369 (6.0)	532 (18.9)	<0.001	1660 (6.8)	340 (18.9)	<0.001	18 077 (4.9)	3751 (19.7)	<0.001	157 559 (18.6)	36 020 (29.9)	<0.001
BMI	Mean (S.D.)	584.7 (13 064.0)	570.1 (12 155.7)	0.924	557.4 (12 302.3)	366.8 (9463.4)	0.466	734.1 (14 708.4)	535.9 (13 666.8)	0.619	550.6 (12 199.9)	448.8 (11 076.0)	0.312	644.0 (70 495.8)	555.0 (13 385.9)	0.69
CRP (mg/l)	Mean (S.D.)	18.4 (30.6)	23.6 (33.6)	<0.001	10.7 (20.1)	12.2 (22.6)	0.062	12.4 (21.2)	16.3 (25.8)	<0.001	8.0 (19.0)	10.5 (22.8)	<0.001	7.4 (17.9)	9.1 (21.1)	<0.001

CVD: cardiovascular disease.

**Figure 1.**
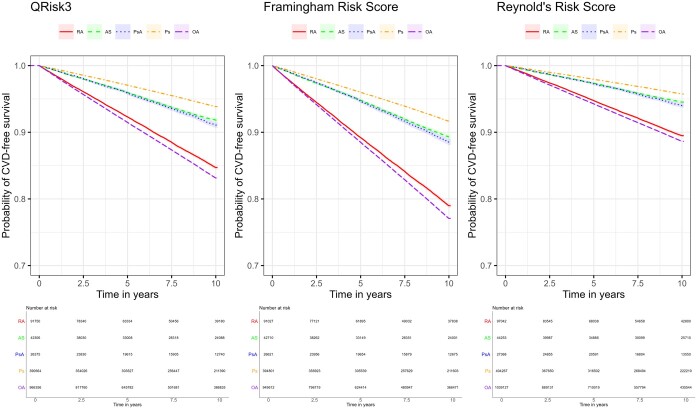
Kaplan–Meier curves showing incidence of cardiovascular disease events for each disease according to the three risk tool definitions

The Kaplan–Meier curves showing the incidence of CVD events during follow-up for each cohort are shown in Fig. 1. Regardless of the precise definition for CVD events, individuals with RA had a higher incidence of CVDs than those with AS or PsA, who had broadly similar CVD incidence. Individuals with psoriasis had the lowest incidence of CVD events. Individuals with OA had the highest incidence of CVD events, broadly similar to the incidence in RA.

Supplementary Fig. S1, available at *Rheumatology* online, shows boxplots of the calculated risk scores for each risk tool according to the disease definitions. Individuals with OA or RA were generally assigned higher risk scores than those with psoriasis, PsA or AS, reflecting the more frequent occurrence of CVD events in these patient groups in our cohort.

**Figure 2.**
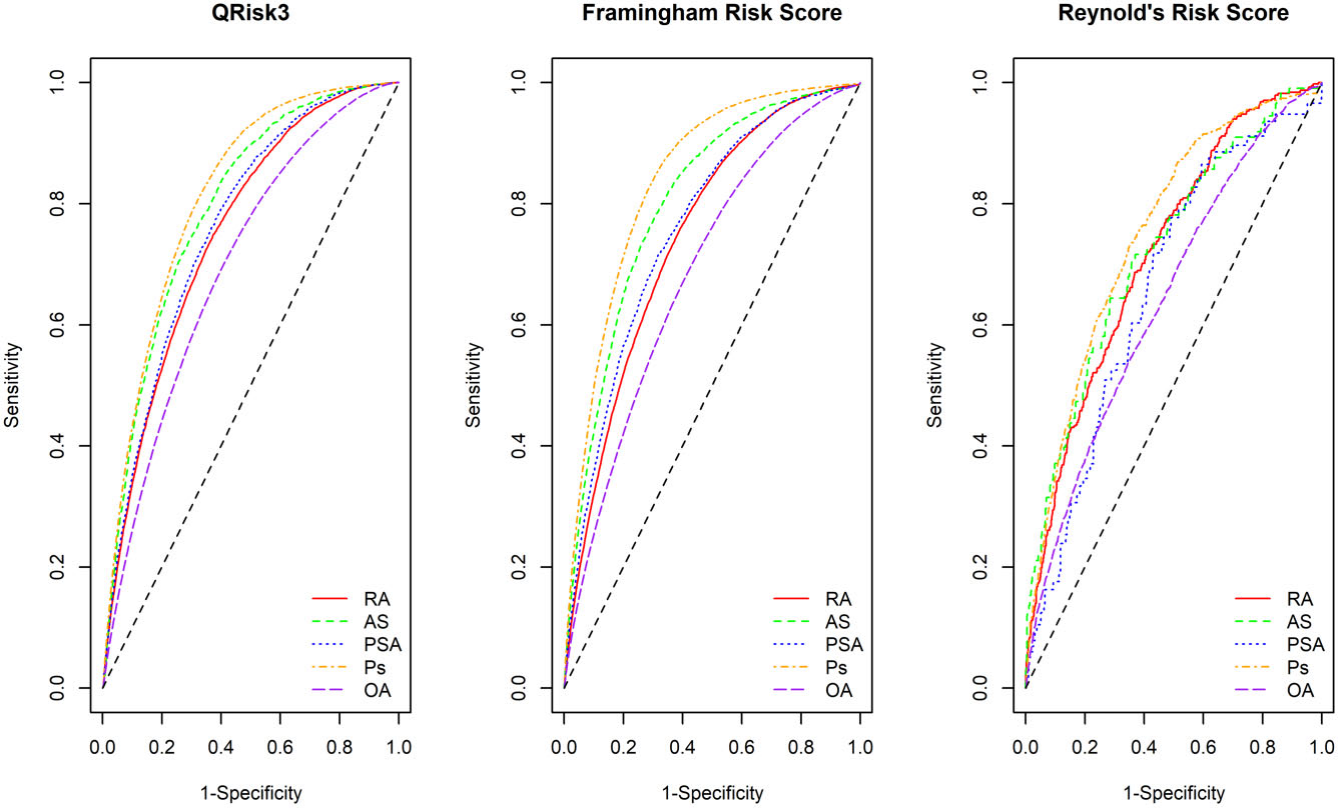
Receiver operating characteristic curves for cardiovascular risk prediction in each rheumatic disease, using the QRISK-3, Framingham and Reynolds Risk Scores

Fig. 2 shows the receiver operating characteristic (ROC) curves for each CVD risk prediction tool. The accuracy of the CVD risk prediction was broadly similar for RA and PsA for each tool. Predictions for AS were more accurate than for RA or PsA, using the QRISK3 or FRS, but similarly accurate if using RRS. CVD risk prediction was most accurate for psoriasis and least accurate for OA for all three prediction tools. Individuals with PsA had noticeably less accurate predictions that those with psoriasis (e.g. AUC of 0.764 *vs* 0.815 for QRISK3, Table 3). AUC for QRISK3 ranged between 0.698 for OA to 0.815 for psoriasis, indicating reasonably good predictive performance (Table 3). The AUCs for the FRS were similar, while the RRS achieved slightly lower AUCs in this cohort, ranging between 0.639 for OA and 0.752 for patients with psoriasis. Both the QRISK3 and FRS obtained good sensitivity and specificity levels using the 10% risk threshold. However, this threshold gave poor sensitivity for the RRS (Table 3).

**Table 3.** Prediction accuracy of each CVD risk tool at 10% and 20% thresholds, for each disease group

Risk score	Disease	AUC	10% risk threshold				20% risk threshold			
			Sensitivity	Specificity	PPV	NPV	Sensitivity	Specificity	PPV	NPV
QRISK3	RA	0.752	0.902	0.406	0.213	0.958	0.715	0.659	0.272	0.928
	AS	0.794	0.746	0.696	0.184	0.968	0.500	0.864	0.253	0.949
	PSA	0.764	0.742	0.649	0.176	0.961	0.466	0.842	0.230	0.940
	Ps	0.815	0.776	0.708	0.171	0.976	0.528	0.861	0.228	0.959
	OA	0.698	0.913	0.292	0.199	0.946	0.700	0.592	0.248	0.911
Framingham Risk Score	RA	0.746	0.777	0.589	0.335	0.909	0.422	0.852	0.432	0.847
	AS	0.799	0.723	0.744	0.253	0.957	0.381	0.915	0.351	0.925
	PSA	0.760	0.694	0.697	0.229	0.946	0.349	0.905	0.323	0.915
	Ps	0.840	0.740	0.784	0.238	0.971	0.415	0.923	0.329	0.945
	OA	0.686	0.851	0.383	0.291	0.896	0.503	0.742	0.367	0.834
Reynolds Risk Score	RA	0.713	0.160	0.954	0.282	0.910	0.060	0.991	0.436	0.904
	AS	0.733	0.142	0.980	0.338	0.941	0.066	0.996	0.533	0.937
	PSA	0.663	0.038	0.984	0.164	0.924	0.014	0.993	0.148	0.923
	Ps	0.752	0.096	0.984	0.258	0.951	0.028	0.995	0.244	0.948
	OA	0.639	0.153	0.945	0.239	0.908	0.045	0.987	0.284	0.902

CVD: cardiovascular disease; AUC: area under the curve; PPV: positive predictive value; NPV: negative predictive value.

**Figure 3.**
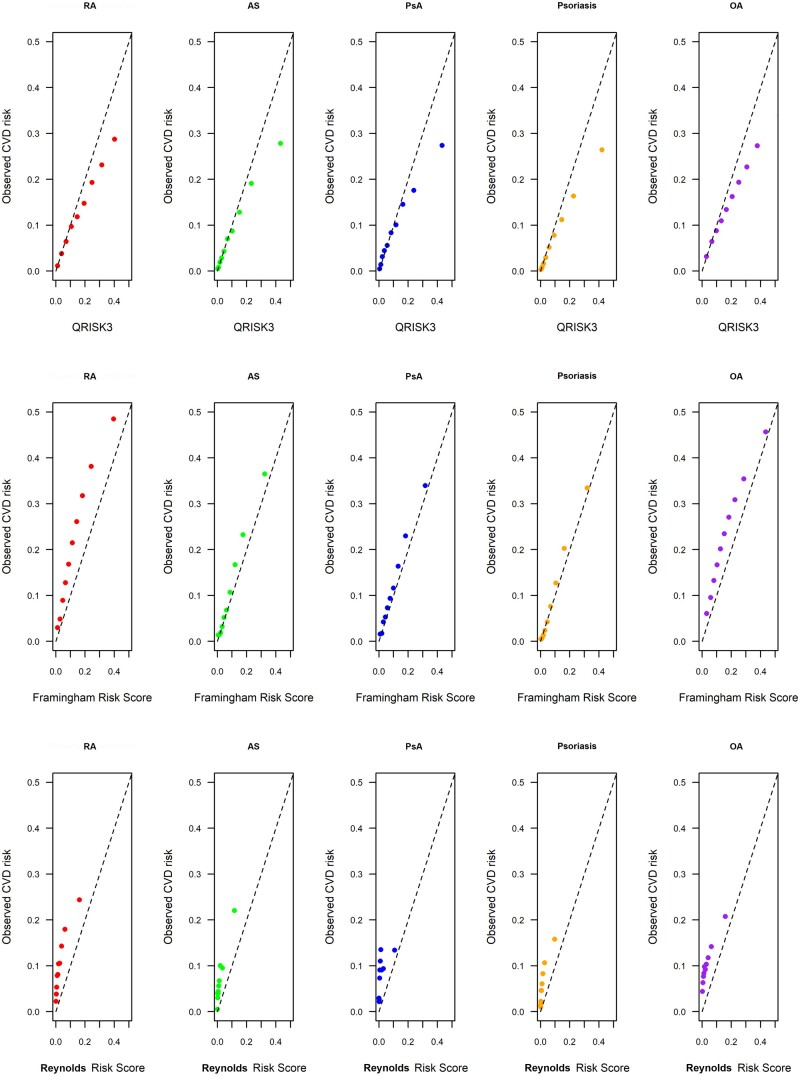
Calibration plots comparing observed CVD risk in deciles of predicted CVD risk using each of the three CVD risk tools, and for each disease cohort. CVD: cardiovascular disease

We assessed the calibration of each CVD risk prediction tool (Fig. 3). In general, the FRS was reasonably well calibrated for each condition, but tended to underestimate the CVD risk in RA or OA. The RRS tended to underpredict CVD risk, while the QRISK3 score overpredicted CVD risk, especially for the most high-risk individuals.

The following illustrates the potential implications of changes in classification accuracy among a hypothetical group of 1000 individuals. In the general population, for every 1000 patients we might expect 46 CVD cases. We anticipate 30 of these cases to be detected using QRISK3 (with a 10% risk threshold), along with 221 individuals who would be incorrectly identified as high risk. By contrast, for a hypothetical 1000 individuals with psoriasis, we anticipate 49 CVD cases, with 38 identified using QRISK3 and 278 individuals incorrectly identified as high risk. For 1000 individuals with AS, we anticipate 68 CVD cases, 51 correctly identified using QRISK3 and 283 individuals wrongly labelled as being at high risk. This show that QRISK3 tends to overpredict CVD risk in patients with rheumatic conditions. Supplementary Table S4, available at *Rheumatology* online, shows the equivalent calculations for each inflammatory condition, as well as similar assessment using the FRS and RRS, showing that the FRS and RRS often underestimate CVD risk. Supplementary Figures S2 and S3, available at *Rheumatology* online, show the same information graphically.

Supplementary Figs S4–S6, available at *Rheumatology* online show that the overprediction with QRISK3, and underprediction with FRS and RRS, are most evident in individuals aged 60+.

In the main analysis, we only required one code to define each rheumatic condition. To reduce misclassification, we repeated our analyses but required at least two codes for RA, AS, PsA, psoriasis or OA for inclusion. This reduced the number of patients included in each cohort (Supplementary Table S5, available at *Rheumatology* online), but had very little impact on the discrimination (Supplementary Table S6 and Fig. S7, available at *Rheumatology* online) or calibration (Supplementary Table S8, available at *Rheumatology* online) of the models.

## Discussion

We assessed the performance of three commonly used general population CVD risk prediction tools in RA, in by far the largest cohorts to date of PsA or AS, and for the first time in psoriasis. In general, CVD risk for RA, AS or psoriatic disease were less accurately predicted using each of the three risk prediction tools than the reported accuracies in the original publications. Underperformance of CVD risk tools are often attributed to inflammatory burden, yet our results showed equally limited performance in an OA population. This suggest that inflammation is not the sole explanation for poor CVD risk prediction in rheumatic patients.

The original QRISK3 paper reported a C-index of 0.880 for women (95% CI 0.879–0.882) and 0.858 for men (95% CI: 0.857–0.860) [4]. The C-index is similar to the time-dependent AUC we use in our analysis, except that it does not account for censoring and ignores pairs of observations in which the individual with a shorter observation period did not experience the event [19]. We did not achieve time-dependent AUCs at this level for any of the groups in our analysis. For example, the best time-dependent AUC in our study was achieved by people with psoriasis (0.815, 95% CI: 0.789–0.836). The Framingham Study reported a C-index of 0.763 for men (95% CI: 0.746–0.780) and 0.793 for women (95% CI: 0.772–0.814) [3]. Our study found broadly similar time-dependent AUCs for individuals with psoriasis (0.840, 95% CI: 0.813–0.855), PsA (0.761, 95% CI: 0.737–0.785) and AS (0.800 95% CI: 0.781–0.824), suggesting the use of FRS may be reasonably reliable. Time-dependent AUCs for individuals with RA were lower, suggesting less reliable CVD risk prediction.

Our results show that most of the overprediction and underprediction of CVD risk is occurring in individuals over 60 years of age. The average age in the original QRISK3 development cohort was 42.6 years for males and 43.3 years for females. In contrast, the average ages of our populations are higher, reflecting a diagnosis of each disease at an older age.

Arts *et al.* report underestimated risks for FRS and RRS, and overestimated risks for QRISK2 for 1157 individuals with physician-confirmed RA in the Netherlands [7]. We obtained similar results in our RA population, which suggests that, at least for RA, the potential for misclassification using diagnostic codes is unlikely to meaningfully affect our conclusions.

An Italian study of 155 individuals with PsA reported generally slightly higher AUCs (0.864 for QRISK2, 0.758 for FRS with EULAR multiplier, and 0.718 for RRS with EULAR multiplier) [20] than we observed in CPRD (0.764 for QRISK3, 0.760 for FRS and 0.663 for RRS). They also suggest that the FRS underpredicts CVD risk, and that QRISK2 and RRS underpredicted CVD risk in lower-risk individuals and overpredicted CVD risk in high-risk individuals. Our findings for the for the RRS and QRISK3 tools broadly agree with these findings. We found that the FRS was generally well calibrated and reasonably accurate in patients with PsA (*n* = 26 621).

An Italian study of 133 individuals with AS (with only 18 CVD events) reported C-indices generally lower than the time-dependent AUCs we report in Table 3 (0.66 for QRISK3, 0.72 for RRS and 0.66 for FRS) [21]. Their calibration results suggest that the highest-risk individuals have an overestimated CVD risk using all three risk tools, while some individuals, particularly those with predicted risks of between 0.05 and 0.2, have underestimated risks. Our CPRD cohort results suggest that CVD risk was generally well calibrated in patients with AS (*n* = 42 306 for the QRISK3 tool), although the RRS performed less well.

Patients with psoriasis in our study had a lower incidence of CVD events. This is in keeping with the findings of other studies [22, 23]. A possible explanation for this may be the earlier age at diagnosis in this cohort. Regardless, performance of the risk prediction tools was still generally reduced in comparison with performance in the general populations in which the risk prediction tools were developed.

We did not consider the use of the EULAR multiplier in this study [8]. It has previously been shown that this multiplier can improve the calibration of predictions in some cases, but does not improve discriminatory ability [20, 24]. In our study, QRISK already overpredicted CVD risk in general, so multiplying assigned probabilities by 1.5 would not be helpful. The EULAR multiplier may help with the calibration of predictions using FRS or RRS, but would not improve the discrimination of the model, and could result in overprescription of statins, for example.

As a practical guide for clinicians, one might consider that overestimation of risk is better than underestimation. If this is accepted, then QRISK3 would be the most appropriate tool to adopt. However, this should be weighed against the impact of increased prescription of statins, possible interaction with other medications, and burdens from overprescription. RRS in particular seems to underperform in this cohort, and the thresholds of 10% and 20% do not appear to be good thresholds for determining moderate or high CVD risk. It would be interesting for future research to compare risk stratification using risk prediction tools for JAK inhibitors, *vs* the current non-quantitative approach using risk factors from the ORAL Surveillance trial [25].

It may be that disease-specific risk models would provide more accurate assessments of CVD risk. However, initial efforts to develop RA-specific risk prediction tools have proved challenging [10, 13]. This may be due to challenges in access to suitable population-based data, since different countries have different access to health-care systems. CPRD does offer an interesting potential in this respect, because GP data would include both severe and mild versions of each disease. However, the data for large longitudinal cohorts may suffer from confounding due to changes in disease or outcome incidence rates over time. Additionally, information about treatment with biologics may not be accurately available in health records such as CPRD. Treatment for inflammation may modify CVD risk, and this would be difficult to capture in CPRD data. A key need is to identify the additional risk factors that drive increased risk. Previous attempts at developing RA-specific risk tools considered markers of inflammation such as CRP and ESR. If inflammation is not driving the increased risk, as may be suggested by our OA results, then further biomarkers must be identified that can more accurately predict CVD risk.

Analgesia (including NSAID) consumption, pain, and psychological and physical function in inflammatory arthritis are more likely comparable with those in OA than in healthy/general population controls. Our study shows that CVD risk prediction tools also perform poorly in patients with OA, both in terms of calibration and discrimination. This suggests that it is unlikely to be inflammation alone that is driving the poor performance of CVD risk prediction tools in inflammatory arthritis. Further research is needed to investigate the causes of additional CVD risk in these cohorts.

Our study is the largest assessment to date of the performance of CVD risk prediction tools in individuals with RA, AS or PsA. It is also, to our knowledge, the first in patients with psoriasis or OA. The use of primary care data, rather than more specialized registry data, allows better comparison with the populations in which the risk prediction tools were developed. Our results are also more likely to be generalizable to the broader disease population, rather than just to highly selected patients from academic centres.

All studies within CPRD are limited by the use of diagnosis codes. It is possible that some diagnoses (both of rheumatic conditions and cardiovascular events) may have been either missed or misclassified using these diagnostic codes. Where possible, we used code lists from the original publications, so that any potential misclassification is unlikely to differ from the original studies in which the prediction tools were developed [4]. We did not have access to data on cause of death. Some individuals may well have died due to CVD-related causes, but this may not be reflected in the GP record [16]. This means we may have underestimated the number of CVD events in each patient group. However, we have tried to mitigate against this by linking GP records within CPRD to HES to capture any additional diagnoses related to hospital visits. Nevertheless, this will not fully remove this limitation. It is worth noting that identifying additional cases would only worsen underprediction with FRS and RRS. In addition, our conclusions are largely the same as those of the largest study of this kind in patients with RA, which did have access to cause-of-death data [7]. We did not have data on disease severity for each condition, which may be a factor in CVD risk.

In conclusion, our study shows that CVD risk prediction is often inaccurate in patients with inflammatory rheumatic and skin conditions. Clinicians should be aware that QRISK3 tends to overestimate CVD risk, while RRS tends to underestimate risk. The FRS performed reasonably well for patients with AS, PsA or psoriasis, but underestimated risk in patients with RA or OA. Underperformance of CVD risk prediction in OA—not typically considered an inflammatory disease—suggests that future risk prediction tools in inflammatory diseases need to capture disease impact other than inflammatory burden.

## Supplementary Material

kead610_Supplementary_Data

## Data Availability

The data are available directly from CPRD. The codelists are available in the Supplementary material.
