# Supplementary material for: The predictive accuracy of cardiovascular disease risk prediction tools in inflammatory arthritis and psoriasis: an observational validation study using the Clinical Practice Research Datalink
Source: Rheumatology (Oxford). 2023 Nov 15;63(12):3432–41. doi: 10.1093/rheumatology/kead610 (PMC11636560; doi:10.1093/rheumatology/kead610)
Supplement: kead610_Supplementary_Data [file kead610_supplementary_data.docx]

**The predictive accuracy of cardiovascular risk prediction tools in inflammatory arthritis: An observational validation study using the Clinical Practice Research Datalink**

David M Hughes^1^, Jose Ignacio Cuitun Coronado^2^, Pieta Schofield^3^, Zenas Z.N. Yiu^4^, Sizheng Steven Zhao^5^

1. Department of Health Data Science, University of Liverpool, Liverpool, UK.
2. Department of Population Health Sciences, University of Bristol, Bristol, UK.
3. Institute of Population Health, University of Liverpool, Liverpool, UK.
4. Centre for Dermatology Research, Northern Care Alliance NHS Foundation Trust, The University of Manchester, Manchester Academic Health Science Centre, National Institute for Health and Care Research Manchester Biomedical Research Centre, Manchester, UK.
5. Centre for Epidemiology Versus Arthritis, Division of Musculoskeletal and Dermatological Science, School of Biological Sciences, Faculty of Biological Medicine and Health, The University of Manchester, Manchester Academic Health Science Centre, Manchester, UK.

Supplementary Table S: Baseline Characteristics of patients in the CPRD suitable for calculating QRISK3 score

|  |  | Rheumatoid Arthritis | | | Ankylosing Spondylitis | | | Psoriatic Arthritis | | | Psoriasis | | | Osteoarthritis | | |
| --- | --- | --- | --- | --- | --- | --- | --- | --- | --- | --- | --- | --- | --- | --- | --- | --- |
| Risk Factor | levels | No CVD | CVD | p | No CVD | CVD | p | No CVD | CVD | p | No CVD | CVD | p | No CVD | CVD | p |
| Total N (%) |  | 81181 (88.5) | 10569 (11.5) |  | 39485 (93.3) | 2821 (6.7) |  | 24573 (93.2) | 1802 (6.8) |  | 371586 (95.1) | 19078 (4.9) |  | 844752 (87.5) | 120505 (12.5) |  |
| Rheumatoid Arthritis |  |  |  |  | 37460 (94.9) | 2614 (92.7) | <0.001 | 3747 (15.2) | 351 (19.5) | <0.001 | 8016 (2.2) | 855 (4.5) | <0.001 |  |  |  |
| Sex | Male | 23265 (28.7) | 3974 (37.6) | <0.001 | 15910 (40.3) | 1340 (47.5) | <0.001 | 11677 (47.5) | 1026 (56.9) | <0.001 | 166082 (44.7) | 10487 (55.0) | <0.001 | 315701 (37.4) | 53074 (44.0) | <0.001 |
| Age | Mean (SD) | 55.7 (15.1) | 66.8 (11.8) | <0.001 | 46.2 (15.4) | 62.7 (13.4) | <0.001 | 46.4 (13.4) | 58.9 (11.8) | <0.001 | 41.0 (16.7) | 62.2 (13.5) | <0.001 | 62.0 (12.4) | 69.5 (11.1) | <0.001 |
| Ethnicity | White | 71718 (88.3) | 9685 (91.6) | <0.001 | 34657 (87.8) | 2535 (89.9) | 0.007 | 22176 (90.2) | 1626 (90.2) | 0.583 | 330312 (88.9) | 17623 (92.4) | <0.001 | 751020 (88.9) | 109649 (91.0) | <0.001 |
|  | Indian | 1889 (2.3) | 183 (1.7) |  | 852 (2.2) | 60 (2.1) |  | 491 (2.0) | 35 (1.9) |  | 5978 (1.6) | 262 (1.4) |  | 15360 (1.8) | 2127 (1.8) |  |
|  | Pakistani | 900 (1.1) | 82 (0.8) |  | 501 (1.3) | 30 (1.1) |  | 259 (1.1) | 18 (1.0) |  | 3437 (0.9) | 146 (0.8) |  | 7347 (0.9) | 1142 (0.9) |  |
|  | Bangladeshi | 328 (0.4) | 20 (0.2) |  | 221 (0.6) | 15 (0.5) |  | 112 (0.5) | 4 (0.2) |  | 1821 (0.5) | 54 (0.3) |  | 2672 (0.3) | 368 (0.3) |  |
|  | Other Asian | 805 (1.0) | 56 (0.5) |  | 473 (1.2) | 32 (1.1) |  | 210 (0.9) | 16 (0.9) |  | 3996 (1.1) | 125 (0.7) |  | 7639 (0.9) | 785 (0.7) |  |
|  | Black African | 668 (0.8) | 45 (0.4) |  | 428 (1.1) | 18 (0.6) |  | 59 (0.2) | 1 (0.1) |  | 2690 (0.7) | 31 (0.2) |  | 10324 (1.2) | 645 (0.5) |  |
|  | Black Caribbean | 810 (1.0) | 100 (0.9) |  | 316 (0.8) | 14 (0.5) |  | 52 (0.2) | 5 (0.3) |  | 2306 (0.6) | 88 (0.5) |  | 9644 (1.1) | 1170 (1.0) |  |
|  | Chinese | 170 (0.2) | 5 (0.0) |  | 108 (0.3) | 0 (0.0) |  | 47 (0.2) | 2 (0.1) |  | 1173 (0.3) | 21 (0.1) |  | 1615 (0.2) | 125 (0.1) |  |
|  | Other | 3893 (4.8) | 393 (3.7) |  | 1929 (4.9) | 117 (4.1) |  | 1167 (4.7) | 95 (5.3) |  | 19873 (5.3) | 728 (3.8) |  | 39131 (4.6) | 4494 (3.7) |  |
| Weight | Mean (SD) | 74.9 (18.0) | 75.0 (17.7) | 0.647 | 74.3 (17.5) | 76.2 (17.3) | <0.001 | 80.9 (19.5) | 83.4 (19.0) | <0.001 | 74.4 (19.4) | 79.4 (18.5) | <0.001 | 79.8 (18.7) | 78.9 (18.0) | <0.001 |
| Height | Mean (SD) | 165.1 (13.9) | 164.7 (14.3) | 0.041 | 167.1 (13.7) | 166.3 (12.0) | 0.004 | 168.3 (14.8) | 168.6 (13.5) | 0.464 | 167.3 (15.4) | 167.7 (13.7) | <0.001 | 166.1 (13.5) | 165.6 (13.7) | <0.001 |
| BMI | Mean (SD) | 584.7 (13064.0) | 570.1 (12155.7) | 0.924 | 557.4 (12302.3) | 366.8 (9463.4) | 0.466 | 734.1 (14708.4) | 535.9 (13666.8) | 0.619 | 550.6 (12199.9) | 448.8 (11076.0) | 0.312 | 644.0 (70495.8) | 555.0 (13385.9) | 0.69 |
| SBP | Mean (SD) | 132.2 (110.3) | 140.0 (23.2) | <0.001 | 126.8 (56.4) | 137.2 (19.9) | <0.001 | 129.2 (19.1) | 137.7 (18.7) | <0.001 | 126.2 (58.5) | 139.0 (22.3) | <0.001 | 135.1 (285.5) | 140.4 (45.8) | <0.001 |
| SBP Variability | Mean (SD) | 13.3 (217.5) | 13.8 (37.4) | 0.832 | 12.0 (92.3) | 14.1 (23.1) | 0.312 | 11.2 (47.3) | 12.8 (6.9) | 0.236 | 11.1 (87.7) | 14.1 (61.1) | <0.001 | 13.1 (156.3) | 14.4 (63.6) | 0.01 |
| Total Cholesterol | Mean (SD) | 202.7 (144.2) | 206.6 (44.1) | 0.051 | 200.8 (40.7) | 209.3 (44.8) | <0.001 | 204.3 (86.2) | 209.5 (50.4) | 0.06 | 203.2 (81.0) | 212.4 (55.8) | <0.001 | 204.5 (404.1) | 203.3 (68.2) | 0.406 |
| HDL Cholesterol | Mean (SD) | 57.7 (55.8) | 55.5 (17.7) | 0.01 | 57.2 (41.1) | 55.4 (17.0) | 0.139 | 54.3 (44.4) | 51.8 (17.6) | 0.103 | 56.3 (33.9) | 54.3 (18.0) | <0.001 | 58.4 (42.2) | 56.0 (31.2) | <0.001 |
| HDL Cholesterol Ratio | Mean (SD) | 3.8 (3.8) | 4.0 (2.9) | <0.001 | 3.8 (2.1) | 4.0 (2.0) | <0.001 | 4.1 (3.2) | 4.4 (2.3) | 0.018 | 3.9 (2.6) | 4.2 (3.6) | <0.001 | 3.8 (9.2) | 3.9 (2.9) | 0.001 |
| HbA1c | Mean (SD) | 6.3 (6.0) | 6.7 (4.1) | 0.003 | 6.4 (7.8) | 6.7 (4.1) | 0.38 | 6.2 (6.1) | 6.6 (1.7) | 0.296 | 6.5 (8.2) | 6.7 (3.3) | 0.167 | 6.2 (3.6) | 6.7 (3.3) | <0.001 |
| CRP | Mean (SD) | 18.4 (30.6) | 23.6 (33.6) | <0.001 | 10.7 (20.1) | 12.2 (22.6) | 0.062 | 12.4 (21.2) | 16.3 (25.8) | <0.001 | 8.0 (19.0) | 10.5 (22.8) | <0.001 | 7.4 (17.9) | 9.1 (21.1) | <0.001 |
| Smoking | Never | 29661 (36.5) | 2940 (27.8) | <0.001 | 15741 (39.9) | 920 (32.6) | <0.001 | 9537 (38.8) | 528 (29.3) | <0.001 | 140788 (37.9) | 5129 (26.9) | <0.001 | 384992 (45.6) | 45325 (37.6) | <0.001 |
|  | Former | 22181 (27.3) | 3408 (32.2) |  | 9746 (24.7) | 880 (31.2) |  | 6778 (27.6) | 595 (33.0) |  | 83045 (22.3) | 6155 (32.3) |  | 253433 (30.0) | 41429 (34.4) |  |
|  | Light | 15981 (19.7) | 2033 (19.2) |  | 8046 (20.4) | 578 (20.5) |  | 5066 (20.6) | 398 (22.1) |  | 86232 (23.2) | 4675 (24.5) |  | 131570 (15.6) | 18908 (15.7) |  |
|  | Medium | 1569 (1.9) | 222 (2.1) |  | 859 (2.2) | 68 (2.4) |  | 425 (1.7) | 40 (2.2) |  | 8826 (2.4) | 491 (2.6) |  | 8718 (1.0) | 1445 (1.2) |  |
|  | Heavy | 942 (1.2) | 156 (1.5) |  | 478 (1.2) | 47 (1.7) |  | 238 (1.0) | 40 (2.2) |  | 4432 (1.2) | 421 (2.2) |  | 5309 (0.6) | 952 (0.8) |  |
|  | (Missing) | 10847 (13.4) | 1810 (17.1) |  | 4615 (11.7) | 328 (11.6) |  | 2529 (10.3) | 201 (11.2) |  | 48263 (13.0) | 2207 (11.6) |  | 60730 (7.2) | 12446 (10.3) |  |
| Diabetes |  | 5233 (6.4) | 1177 (11.1) | <0.001 | 1612 (4.1) | 308 (10.9) | <0.001 | 1385 (5.6) | 206 (11.4) | <0.001 | 12917 (3.5) | 1937 (10.2) | <0.001 | 87546 (10.4) | 17951 (14.9) | <0.001 |
| Type 1 Diabetes |  | 646 (0.8) | 130 (1.2) | <0.001 | 169 (0.4) | 34 (1.2) | <0.001 | 266 (1.1) | 42 (2.3) | <0.001 | 2706 (0.7) | 357 (1.9) | <0.001 | 9990 (1.2) | 2567 (2.1) | <0.001 |
| Type 2 Diabetes |  | 4587 (5.7) | 1047 (9.9) | <0.001 | 1443 (3.7) | 274 (9.7) | <0.001 | 2773 (11.3) | 439 (24.4) | <0.001 | 26457 (7.1) | 4034 (21.1) | <0.001 | 130706 (15.5) | 27531 (22.8) | <0.001 |
| Atrial fibrillation |  | 1365 (1.7) | 549 (5.2) | <0.001 | 309 (0.8) | 122 (4.3) | <0.001 | 216 (0.9) | 71 (3.9) | <0.001 | 2397 (0.6) | 828 (4.3) | <0.001 | 20677 (2.4) | 7422 (6.2) | <0.001 |
| Erectile Dysfunction |  | 2559 (3.2) | 440 (4.2) | <0.001 | 1172 (3.0) | 181 (6.4) | <0.001 | 870 (3.5) | 115 (6.4) | <0.001 | 10116 (2.7) | 1166 (6.1) | <0.001 | 49775 (5.9) | 8309 (6.9) | <0.001 |
| Migraine |  | 5294 (6.5) | 479 (4.5) | <0.001 | 3066 (7.8) | 188 (6.7) | <0.001 | 1742 (7.1) | 119 (6.6) | 0.466 | 21870 (5.9) | 904 (4.7) | <0.001 | 53748 (6.4) | 5777 (4.8) | <0.001 |
| Chronic Kidney Disease |  | 2268 (2.8) | 507 (4.8) | <0.001 | 524 (1.3) | 117 (4.1) | <0.001 | 272 (1.1) | 72 (4.0) | <0.001 | 3331 (0.9) | 698 (3.7) | <0.001 | 37809 (4.5) | 8511 (7.1) | <0.001 |
| Severe Mental Illness |  | 578 (0.7) | 63 (0.6) | 0.199 | 273 (0.7) | 28 (1.0) | 0.085 | 181 (0.7) | 13 (0.7) | 1 | 2924 (0.8) | 223 (1.2) | <0.001 | 7609 (0.9) | 1133 (0.9) | 0.181 |
| SLE |  | 515 (0.6) | 69 (0.7) | 0.873 | 55 (0.1) | 8 (0.3) | 0.095 | 41 (0.2) | 4 (0.2) | 0.801 | 300 (0.1) | 37 (0.2) | <0.001 | 888 (0.1) | 121 (0.1) | 0.670 |
| Anti-psychotic Medication |  | 177 (0.2) | 25 (0.2) | 0.786 | 77 (0.2) | 9 (0.3) | 0.231 | 69 (0.3) | 6 (0.3) | 0.863 | 1209 (0.3) | 84 (0.4) | 0.009 | 3150 (0.4) | 470 (0.4) | 0.376 |
| Corticosteroids |  | 8251 (10.2) | 1649 (15.6) | <0.001 | 782 (2.0) | 125 (4.4) | <0.001 | 929 (3.8) | 120 (6.7) | <0.001 | 9569 (2.6) | 787 (4.1) | <0.001 | 19244 (2.3) | 3866 (3.2) | <0.001 |
| Anti-hypertensive Medication |  | 8684 (10.7) | 2361 (22.3) | <0.001 | 2369 (6.0) | 532 (18.9) | <0.001 | 1660 (6.8) | 340 (18.9) | <0.001 | 18077 (4.9) | 3751 (19.7) | <0.001 | 157559 (18.6) | 36020 (29.9) | <0.001 |
| Family History of Heart Disease |  | 15912 (19.6) | 2210 (20.9) | 0.002 | 7796 (19.7) | 682 (24.2) | <0.001 | 5185 (21.1) | 480 (26.6) | <0.001 | 59354 (16.0) | 4305 (22.6) | <0.001 | 181695 (21.5) | 25251 (21.0) | <0.001 |
| Townsend Score | Mean (SD) | 5.3 (2.9) | 5.4 (2.8) | 0.006 | 5.3 (2.9) | 5.3 (2.9) | 0.421 | 5.2 (2.9) | 5.2 (2.9) | 0.452 | 5.4 (2.9) | 5.3 (2.8) | <0.001 | 5.0 (2.9) | 5.2 (2.8) | <0.001 |
|  |  |  |  |  |  |  |  |  |  |  |  |  |  |  |  |  |

Supplementary Table S2: Baseline Characteristics of patients in the CPRD suitable for calculating Framingham Risk Score.

|  |  | Rheumatoid Arthritis | | | Ankylosing Spondylitis | | | Psoriatic Arthritis | | | Psoriasis | | | Osteoarthritis | | |
| --- | --- | --- | --- | --- | --- | --- | --- | --- | --- | --- | --- | --- | --- | --- | --- | --- |
| Risk Factor | levels | No CVD | CVD | p | No CVD | CVD | p | No CVD | CVD | p | No CVD | CVD | p | No CVD | CVD | p |
| Total N (%) |  | 76318 (83.8) | 14709 (16.2) |  | 38964 (91.2) | 3746 (8.8) |  | 24266 (91.2) | 2355 (8.8) |  | 368483 (93.3) | 26318 (6.7) |  | 785284 (82.8) | 163244 (17.2) |  |
| Rheumatoid Arthritis |  |  |  |  | 1959 (5.0) | 268 (7.2) | <0.001 | 3653 (15.1) | 465 (19.7) | <0.001 | 7701 (2.1) | 1169 (4.4) | <0.001 |  |  |  |
| Sex | M | 21548 (28.2) | 5305 (36.1) | <0.001 | 15659 (40.2) | 1717 (45.8) | <0.001 | 11521 (47.5) | 1273 (54.1) | <0.001 | 164126 (44.5) | 14009 (53.2) | <0.001 | 290694 (37.0) | 69459 (42.5) | <0.001 |
| Age | Mean (SD) | 54.3 (15.0) | 67.1 (12.1) | <0.001 | 45.3 (14.9) | 63.2 (14.0) | <0.001 | 45.8 (13.1) | 59.2 (12.2) | <0.001 | 40.2 (16.2) | 62.4 (14.0) | <0.001 | 61.0 (12.1) | 70.1 (11.2) | <0.001 |
| Ethnicity | White | 67122 (88.0) | 13513 (91.9) | <0.001 | 34141 (87.6) | 3387 (90.4) | <0.001 | 21874 (90.1) | 2151 (91.3) | 0.268 | 327069 (88.8) | 24373 (92.6) | <0.001 | 695904 (88.6) | 148954 (91.2) | <0.001 |
|  | Indian | 1847 (2.4) | 240 (1.6) |  | 841 (2.2) | 70 (1.9) |  | 487 (2.0) | 45 (1.9) |  | 5976 (1.6) | 337 (1.3) |  | 14711 (1.9) | 2652 (1.6) |  |
|  | Pakistani | 888 (1.2) | 107 (0.7) |  | 506 (1.3) | 37 (1.0) |  | 260 (1.1) | 21 (0.9) |  | 3441 (0.9) | 176 (0.7) |  | 7174 (0.9) | 1323 (0.8) |  |
|  | Bangladeshi | 334 (0.4) | 30 (0.2) |  | 227 (0.6) | 18 (0.5) |  | 119 (0.5) | 4 (0.2) |  | 1897 (0.5) | 72 (0.3) |  | 2655 (0.3) | 469 (0.3) |  |
|  | Other Asian | 778 (1.0) | 82 (0.6) |  | 474 (1.2) | 38 (1.0) |  | 209 (0.9) | 19 (0.8) |  | 4001 (1.1) | 163 (0.6) |  | 7333 (0.9) | 980 (0.6) |  |
|  | Black African | 649 (0.9) | 59 (0.4) |  | 429 (1.1) | 21 (0.6) |  | 59 (0.2) | 3 (0.1) |  | 2714 (0.7) | 54 (0.2) |  | 9970 (1.3) | 899 (0.6) |  |
|  | Black Caribbean | 786 (1.0) | 125 (0.8) |  | 315 (0.8) | 27 (0.7) |  | 52 (0.2) | 7 (0.3) |  | 2338 (0.6) | 125 (0.5) |  | 9040 (1.2) | 1656 (1.0) |  |
|  | Chinese | 167 (0.2) | 10 (0.1) |  | 109 (0.3) | 1 (0.0) |  | 47 (0.2) | 2 (0.1) |  | 1184 (0.3) | 30 (0.1) |  | 1559 (0.2) | 168 (0.1) |  |
|  | Other | 3747 (4.9) | 543 (3.7) |  | 1922 (4.9) | 147 (3.9) |  | 1159 (4.8) | 103 (4.4) |  | 19863 (5.4) | 988 (3.8) |  | 36938 (4.7) | 6143 (3.8) |  |
| Weight | Mean (SD) | 74.9 (17.9) | 74.7 (17.8) | 0.207 | 74.2 (17.5) | 75.8 (17.5) | <0.001 | 80.8 (19.4) | 83.2 (19.6) | <0.001 | 74.3 (19.4) | 79.2 (18.9) | <0.001 | 79.8 (18.6) | 78.9 (18.4) | <0.001 |
| Height | Mean (SD) | 165.1 (13.7) | 164.6 (14.4) | <0.001 | 167.2 (13.7) | 166.1 (12.6) | <0.001 | 168.3 (14.9) | 168.2 (13.5) | 0.729 | 167.3 (15.5) | 167.6 (13.4) | 0.005 | 166.2 (13.4) | 165.5 (13.7) | <0.001 |
| BMI | Mean (SD) | 576.7 (13046.3) | 496.9 (11136.5) | 0.541 | 563.7 (12364.5) | 451.2 (10557.7) | 0.626 | 775.4 (15135.3) | 412.8 (11891.5) | 0.309 | 555.3 (12264.2) | 447.3 (11023.0) | 0.212 | 646.1 (72994.0) | 554.4 (12890.6) | 0.644 |
| SBP | Mean (SD) | 131.6 (113.9) | 139.7 (22.2) | <0.001 | 126.4 (56.8) | 137.0 (20.0) | <0.001 | 129.4 (77.2) | 137.6 (18.4) | <0.001 | 125.8 (59.3) | 139.1 (67.4) | <0.001 | 134.5 (239.7) | 141.4 (384.4) | <0.001 |
| SBP Variability | Mean (SD) | 13.1 (226.1) | 13.9 (38.9) | 0.743 | 12.1 (109.5) | 15.1 (66.7) | 0.158 | 11.6 (71.3) | 12.7 (6.7) | 0.547 | 10.9 (67.0) | 13.8 (54.6) | <0.001 | 12.6 (111.6) | 15.7 (256.5) | <0.001 |
| Total Cholesterol | Mean (SD) | 203.0 (149.1) | 205.2 (43.6) | 0.216 | 200.7 (40.6) | 208.6 (44.6) | <0.001 | 204.2 (86.8) | 208.7 (48.1) | 0.065 | 203.1 (82.0) | 210.6 (52.4) | <0.001 | 205.3 (419.8) | 202.9 (64.2) | 0.068 |
| HDLCholesterol | Mean (SD) | 57.7 (57.6) | 56.5 (17.9) | 0.116 | 57.2 (41.7) | 56.0 (17.1) | 0.275 | 54.3 (44.8) | 52.8 (17.9) | 0.292 | 56.2 (34.3) | 55.5 (18.4) | 0.022 | 58.4 (43.1) | 56.9 (29.5) | <0.001 |
| HDL Cholesterol Ratio | Mean (SD) | 3.8 (2.8) | 4.0 (6.9) | 0.001 | 3.8 (2.2) | 4.0 (1.8) | 0.001 | 4.1 (3.2) | 4.3 (2.1) | 0.088 | 3.9 (2.6) | 4.1 (3.3) | <0.001 | 3.8 (9.5) | 3.8 (2.7) | 0.143 |
| HbA1c | Mean (SD) | 6.2 (6.2) | 6.5 (3.6) | 0.02 | 6.4 (7.9) | 6.7 (5.1) | 0.318 | 6.2 (6.1) | 6.6 (1.7) | 0.182 | 6.5 (8.4) | 6.6 (2.9) | 0.448 | 6.2 (3.6) | 6.7 (3.3) | <0.001 |
| CRP | Mean (SD) | 17.5 (29.0) | 25.4 (38.5) | <0.001 | 10.6 (19.7) | 12.8 (24.3) | 0.001 | 12.2 (20.9) | 16.9 (27.2) | <0.001 | 7.8 (18.8) | 10.8 (22.9) | <0.001 | 7.1 (17.0) | 9.3 (21.5) | <0.001 |
| Smoking | Never | 28249 (37.0) | 4234 (28.8) | <0.001 | 15627 (40.1) | 1245 (33.2) | <0.001 | 9517 (39.2) | 690 (29.3) | <0.001 | 140730 (38.2) | 7222 (27.4) | <0.001 | 363323 (46.3) | 62505 (38.3) | <0.001 |
|  | Former | 20375 (26.7) | 4641 (31.6) |  | 9490 (24.4) | 1148 (30.6) |  | 6613 (27.3) | 774 (32.9) |  | 81071 (22.0) | 8208 (31.2) |  | 231889 (29.5) | 55371 (33.9) |  |
|  | Light | 14957 (19.6) | 2950 (20.1) |  | 7931 (20.4) | 775 (20.7) |  | 4965 (20.5) | 549 (23.3) |  | 85272 (23.1) | 6691 (25.4) |  | 121087 (15.4) | 26062 (16.0) |  |
|  | Medium | 1451 (1.9) | 327 (2.2) |  | 849 (2.2) | 94 (2.5) |  | 426 (1.8) | 46 (2.0) |  | 8734 (2.4) | 719 (2.7) |  | 7946 (1.0) | 1986 (1.2) |  |
|  | Heavy | 858 (1.1) | 223 (1.5) |  | 462 (1.2) | 75 (2.0) |  | 230 (0.9) | 44 (1.9) |  | 4304 (1.2) | 589 (2.2) |  | 4849 (0.6) | 1312 (0.8) |  |
|  | (Missing) | 10428 (13.7) | 2334 (15.9) |  | 4605 (11.8) | 409 (10.9) |  | 2515 (10.4) | 252 (10.7) |  | 48372 (13.1) | 2889 (11.0) |  | 56190 (7.2) | 16008 (9.8) |  |
| Diabetes | 1 | 4607 (6.0) | 1493 (10.2) | <0.001 | 1471 (3.8) | 380 (10.1) | <0.001 | 1299 (5.4) | 279 (11.8) | <0.001 | 11858 (3.2) | 2613 (9.9) | <0.001 | 76155 (9.7) | 23755 (14.6) | <0.001 |
| Type 1 Diabetes | 1 | 557 (0.7) | 186 (1.3) | <0.001 | 156 (0.4) | 43 (1.1) | <0.001 | 248 (1.0) | 62 (2.6) | <0.001 | 2546 (0.7) | 465 (1.8) | <0.001 | 8653 (1.1) | 3345 (2.0) | <0.001 |
| Type 2 Diabetes | 1 | 4050 (5.3) | 1307 (8.9) | <0.001 | 1315 (3.4) | 337 (9.0) | <0.001 | 2649 (10.9) | 551 (23.4) | <0.001 | 24832 (6.7) | 5305 (20.2) | <0.001 | 116176 (14.8) | 36084 (22.1) | <0.001 |
| Atrial Fibrilation | 1 | 849 (1.1) | 689 (4.7) | <0.001 | 206 (0.5) | 161 (4.3) | <0.001 | 157 (0.6) | 81 (3.4) | <0.001 | 1645 (0.4) | 1069 (4.1) | <0.001 | 13171 (1.7) | 9547 (5.8) | <0.001 |
| Erectile Dysfunction | 1 | 2320 (3.0) | 594 (4.0) | <0.001 | 1090 (2.8) | 235 (6.3) | <0.001 | 839 (3.5) | 138 (5.9) | <0.001 | 9593 (2.6) | 1576 (6.0) | <0.001 | 44980 (5.7) | 10845 (6.6) | <0.001 |
| Migraine | 1 | 5091 (6.7) | 658 (4.5) | <0.001 | 3048 (7.8) | 244 (6.5) | 0.005 | 1713 (7.1) | 159 (6.8) | 0.606 | 21863 (5.9) | 1192 (4.5) | <0.001 | 51344 (6.5) | 7563 (4.6) | <0.001 |
| Chronic Kidney Disease | 1 | 1765 (2.3) | 758 (5.2) | <0.001 | 413 (1.1) | 185 (4.9) | <0.001 | 230 (0.9) | 92 (3.9) | <0.001 | 2672 (0.7) | 1028 (3.9) | <0.001 | 29963 (3.8) | 12229 (7.5) | <0.001 |
| Severe Mental Illness | 1 | 544 (0.7) | 95 (0.6) | 0.403 | 256 (0.7) | 47 (1.3) | <0.001 | 185 (0.8) | 16 (0.7) | 0.749 | 2812 (0.8) | 359 (1.4) | <0.001 | 6858 (0.9) | 1661 (1.0) | <0.001 |
| SLE | 1 | 485 (0.6) | 93 (0.6) | 1 | 53 (0.1) | 9 (0.2) | 0.169 | 37 (0.2) | 8 (0.3) | 0.064 | 280 (0.1) | 50 (0.2) | <0.001 | 794 (0.1) | 175 (0.1) | 0.51 |
| Anti-Psychotic Medicaion | 1 | 164 (0.2) | 35 (0.2) | 0.651 | 74 (0.2) | 12 (0.3) | 0.131 | 65 (0.3) | 7 (0.3) | 0.957 | 1156 (0.3) | 152 (0.6) | <0.001 | 2764 (0.4) | 711 (0.4) | <0.001 |
| Corticosteroids | 1 | 7218 (9.5) | 2374 (16.1) | <0.001 | 731 (1.9) | 168 (4.5) | <0.001 | 879 (3.6) | 163 (6.9) | <0.001 | 9233 (2.5) | 1133 (4.3) | <0.001 | 16535 (2.1) | 5407 (3.3) | <0.001 |
| Anti-Hypertensive Medication | 1 | 7151 (9.4) | 3146 (21.4) | <0.001 | 2088 (5.4) | 702 (18.7) | <0.001 | 1492 (6.1) | 444 (18.9) | <0.001 | 15733 (4.3) | 5090 (19.3) | <0.001 | 133502 (17.0) | 48625 (29.8) | <0.001 |
| Family History of Heart Disaese | 1 | 15165 (19.9) | 2842 (19.3) | 0.129 | 7716 (19.8) | 841 (22.5) | <0.001 | 5102 (21.0) | 610 (25.9) | <0.001 | 58618 (15.9) | 5594 (21.3) | <0.001 | 172445 (22.0) | 32086 (19.7) | <0.001 |
| Townsend Score | Mean (SD) | 5.3 (2.9) | 5.4 (2.8) | 0.004 | 5.3 (2.9) | 5.3 (2.9) | 0.233 | 5.2 (2.9) | 5.3 (2.8) | 0.074 | 5.4 (2.9) | 5.4 (2.8) | <0.001 | 5.0 (2.9) | 5.2 (2.8) | <0.001 |

Supplementary Table S3: Baseline Characteristics of patients in the CPRD suitable for calculating Reynolds Risk Score

|  |  | Rheumatoid Arthritis | | | Ankylosing Spondylitis | | | Psoriatic Arthritis | | | Psoriasis | | | Osteoarthritis | | |
| --- | --- | --- | --- | --- | --- | --- | --- | --- | --- | --- | --- | --- | --- | --- | --- | --- |
| Risk factor | levels | No CVD | CVD | p | No CVD | CVD | p | No CVD | CVD | p | No CVD | CVD | p | No CVD | CVD | p |
| Total N (%) |  | 89454 (92.2) | 7588 (7.8) |  | 42289 (95.6) | 1964 (4.4) |  | 26121 (95.4) | 1247 (4.6) |  | 390610 (96.6) | 13657 (3.4) |  | 951212 (91.6) | 86748 (8.4) |  |
| Rheumatoid Arthritis |  |  |  |  | 2192 (5.2) | 138 (7.0) | <0.001 | 3999 (15.3) | 260 (20.9) | <0.001 | 8680 (2.2) | 603 (4.4) | <0.001 |  |  |  |
| Sex | M | 25982 (29.0) | 3009 (39.7) | <0.001 | 17072 (40.4) | 953 (48.5) | <0.001 | 12427 (47.6) | 752 (60.3) | <0.001 | 174984 (44.8) | 7853 (57.5) | <0.001 | 358092 (37.6) | 39831 (45.9) | <0.001 |
| Age | Mean (SD) | 56.4 (15.5) | 67.6 (11.6) | <0.001 | 46.9 (15.8) | 64.3 (13.3) | <0.001 | 46.8 (13.6) | 59.8 (11.7) | <0.001 | 41.6 (17.0) | 63.2 (13.4) | <0.001 | 62.7 (12.5) | 70.7 (10.9) | <0.001 |
| Ethnicity | White | 79090 (88.4) | 7046 (92.9) | <0.001 | 37127 (87.8) | 1784 (90.8) | 0.003 | 23575 (90.3) | 1134 (90.9) | 0.636 | 347526 (89.0) | 12664 (92.7) | <0.001 | 846212 (89.0) | 79643 (91.8) | <0.001 |
|  | Indian | 2085 (2.3) | 95 (1.3) |  | 906 (2.1) | 38 (1.9) |  | 521 (2.0) | 23 (1.8) |  | 6255 (1.6) | 188 (1.4) |  | 17488 (1.8) | 1421 (1.6) |  |
|  | Pakistani | 980 (1.1) | 59 (0.8) |  | 538 (1.3) | 21 (1.1) |  | 275 (1.1) | 10 (0.8) |  | 3575 (0.9) | 108 (0.8) |  | 8615 (0.9) | 743 (0.9) |  |
|  | Bangladeshi | 362 (0.4) | 18 (0.2) |  | 238 (0.6) | 8 (0.4) |  | 125 (0.5) | 2 (0.2) |  | 1958 (0.5) | 32 (0.2) |  | 3152 (0.3) | 245 (0.3) |  |
|  | Other Asian | 861 (1.0) | 39 (0.5) |  | 505 (1.2) | 20 (1.0) |  | 221 (0.8) | 12 (1.0) |  | 4146 (1.1) | 85 (0.6) |  | 8490 (0.9) | 461 (0.5) |  |
|  | Black African | 712 (0.8) | 25 (0.3) |  | 453 (1.1) | 8 (0.4) |  | 62 (0.2) | 1 (0.1) |  | 2780 (0.7) | 14 (0.1) |  | 11021 (1.2) | 368 (0.4) |  |
|  | Black Caribbean | 903 (1.0) | 62 (0.8) |  | 345 (0.8) | 9 (0.5) |  | 60 (0.2) | 2 (0.2) |  | 2453 (0.6) | 64 (0.5) |  | 10929 (1.1) | 729 (0.8) |  |
|  | Chinese | 176 (0.2) | 3 (0.0) |  | 111 (0.3) | 1 (0.1) |  | 48 (0.2) | 1 (0.1) |  | 1209 (0.3) | 14 (0.1) |  | 1753 (0.2) | 71 (0.1) |  |
|  | Other | 4285 (4.8) | 241 (3.2) |  | 2066 (4.9) | 75 (3.8) |  | 1234 (4.7) | 62 (5.0) |  | 20708 (5.3) | 488 (3.6) |  | 43552 (4.6) | 3067 (3.5) |  |
| Weight | Mean (SD) | 75.0 (18.1) | 74.2 (17.0) | 0.001 | 74.4 (17.6) | 75.2 (16.9) | 0.069 | 81.1 (19.6) | 82.3 (18.2) | 0.056 | 74.6 (19.4) | 78.8 (17.7) | <0.001 | 79.9 (18.7) | 78.0 (17.3) | <0.001 |
| Height | Mean (SD) | 165.0 (14.0) | 165.0 (14.1) | 0.969 | 167.0 (13.7) | 165.9 (12.7) | 0.002 | 168.2 (14.8) | 168.6 (14.2) | 0.47 | 167.3 (15.4) | 167.9 (13.3) | <0.001 | 166.0 (13.5) | 165.6 (13.6) | <0.001 |
| BMI | Mean (SD) | 586.6 (13011.6) | 530.5 (11525.5) | 0.751 | 558.5 (12254.2) | 520.8 (11411.7) | 0.904 | 745.0 (14789.7) | 763.8 (16459.1) | 0.969 | 548.5 (12203.8) | 414.9 (10474.7) | 0.259 | 636.7 (66549.3) | 562.5 (13414.7) | 0.765 |
| SBP | Mean (SD) | 132.6 (105.2) | 140.9 (19.8) | <0.001 | 127.2 (54.7) | 138.7 (19.9) | <0.001 | 129.9 (74.4) | 138.7 (18.5) | <0.001 | 126.5 (60.2) | 140.0 (23.8) | <0.001 | 135.3 (269.9) | 141.4 (55.9) | <0.001 |
| SBP Variability | Mean (SD) | 13.3 (206.4) | 14.3 (44.1) | 0.726 | 12.4 (105.8) | 14.4 (18.1) | 0.491 | 11.7 (68.0) | 13.2 (7.3) | 0.512 | 11.2 (88.0) | 14.9 (70.1) | <0.001 | 13.3 (157.2) | 14.7 (63.5) | 0.017 |
| Total Cholesterol | Mean (SD) | 202.1 (137.2) | 206.8 (44.8) | 0.04 | 200.7 (40.9) | 211.6 (46.6) | <0.001 | 204.0 (83.6) | 210.1 (54.9) | 0.056 | 203.0 (79.1) | 212.9 (54.6) | <0.001 | 202.8 (378.5) | 202.8 (48.0) | 0.998 |
| HDLCholesterol | Mean (SD) | 57.6 (53.2) | 55.2 (17.6) | 0.019 | 57.1 (39.5) | 55.4 (17.0) | 0.239 | 54.2 (43.0) | 51.5 (18.0) | 0.144 | 56.2 (33.0) | 53.9 (18.1) | <0.001 | 58.1 (41.0) | 55.6 (35.3) | <0.001 |
| HDL Cholesterol Ratio | Mean (SD) | 3.8 (3.6) | 4.1 (3.4) | <0.001 | 3.8 (2.1) | 4.1 (2.2) | <0.001 | 4.1 (3.1) | 4.5 (3.2) | 0.005 | 3.9 (2.6) | 4.2 (1.9) | <0.001 | 3.7 (8.6) | 3.9 (3.3) | 0.001 |
| HbA1c | Mean (SD) | 6.3 (5.9) | 6.7 (3.1) | 0.01 | 6.4 (7.7) | 6.6 (1.6) | 0.686 | 6.2 (5.9) | 6.7 (1.8) | 0.202 | 6.5 (8.0) | 6.8 (2.9) | 0.1 | 6.2 (3.5) | 6.8 (3.3) | <0.001 |
| CRP | Mean (SD) | 18.6 (30.8) | 24.4 (33.1) | <0.001 | 10.8 (20.2) | 11.5 (17.9) | 0.453 | 12.4 (21.3) | 17.3 (26.5) | <0.001 | 8.0 (19.2) | 10.6 (20.9) | <0.001 | 7.6 (22.2) | 9.6 (22.6) | <0.001 |
| Smoking | Never | 32427 (36.2) | 1977 (26.1) | <0.001 | 16785 (39.7) | 600 (30.5) | <0.001 | 10106 (38.7) | 327 (26.2) | <0.001 | 147211 (37.7) | 3531 (25.9) | <0.001 | 429243 (45.1) | 31535 (36.4) | <0.001 |
|  | Former | 24783 (27.7) | 2535 (33.4) |  | 10602 (25.1) | 630 (32.1) |  | 7276 (27.9) | 407 (32.6) |  | 88472 (22.6) | 4475 (32.8) |  | 290914 (30.6) | 30572 (35.2) |  |
|  | Light | 17447 (19.5) | 1507 (19.9) |  | 8558 (20.2) | 408 (20.8) |  | 5364 (20.5) | 305 (24.5) |  | 90626 (23.2) | 3344 (24.5) |  | 147155 (15.5) | 13710 (15.8) |  |
|  | Medium | 1703 (1.9) | 170 (2.2) |  | 914 (2.2) | 56 (2.9) |  | 454 (1.7) | 26 (2.1) |  | 9285 (2.4) | 364 (2.7) |  | 9658 (1.0) | 1064 (1.2) |  |
|  | Heavy | 1040 (1.2) | 119 (1.6) |  | 524 (1.2) | 32 (1.6) |  | 260 (1.0) | 26 (2.1) |  | 4696 (1.2) | 317 (2.3) |  | 5989 (0.6) | 683 (0.8) |  |
|  | (Missing) | 12054 (13.5) | 1280 (16.9) |  | 4906 (11.6) | 238 (12.1) |  | 2661 (10.2) | 156 (12.5) |  | 50320 (12.9) | 1626 (11.9) |  | 68253 (7.2) | 9184 (10.6) |  |
| Diabetes | 1 | 6342 (7.1) | 910 (12.0) | <0.001 | 1910 (4.5) | 216 (11.0) | <0.001 | 1588 (6.1) | 156 (12.5) | <0.001 | 14723 (3.8) | 1489 (10.9) | <0.001 | 107439 (11.3) | 13258 (15.3) | <0.001 |
| Type 1 Diabetes | 1 | 745 (0.8) | 118 (1.6) | <0.001 | 197 (0.5) | 25 (1.3) | <0.001 | 310 (1.2) | 28 (2.2) | 0.001 | 2967 (0.8) | 283 (2.1) | <0.001 | 12276 (1.3) | 1893 (2.2) | <0.001 |
| Type 2 Diabetes | 1 | 5597 (6.3) | 792 (10.4) | <0.001 | 1713 (4.1) | 191 (9.7) | <0.001 | 3095 (11.8) | 310 (24.9) | <0.001 | 29421 (7.5) | 3015 (22.1) | <0.001 | 157124 (16.5) | 20058 (23.1) | <0.001 |
| Atrial Fibrilation | 1 | 2022 (2.3) | 372 (4.9) | <0.001 | 464 (1.1) | 77 (3.9) | <0.001 | 288 (1.1) | 50 (4.0) | <0.001 | 3370 (0.9) | 567 (4.2) | <0.001 | 31179 (3.3) | 5234 (6.0) | <0.001 |
| Erectile Dysfunction | 1 | 2971 (3.3) | 312 (4.1) | <0.001 | 1335 (3.2) | 110 (5.6) | <0.001 | 969 (3.7) | 77 (6.2) | <0.001 | 11089 (2.8) | 879 (6.4) | <0.001 | 57994 (6.1) | 5983 (6.9) | <0.001 |
| Migraine | 1 | 5819 (6.5) | 296 (3.9) | <0.001 | 3273 (7.7) | 126 (6.4) | 0.035 | 1869 (7.2) | 72 (5.8) | 0.0702 | 23052 (5.9) | 587 (4.3) | <0.001 | 60124 (6.3) | 3750 (4.3) | <0.001 |
| Chronic Kidney Disease | 1 | 2837 (3.2) | 378 (5.0) | <0.001 | 653 (1.5) | 83 (4.2) | <0.001 | 332 (1.3) | 50 (4.0) | <0.001 | 4058 (1.0) | 477 (3.5) | <0.001 | 48564 (5.1) | 6236 (7.2) | <0.001 |
| Severe Mental Illness | 1 | 660 (0.7) | 36 (0.5) | 0.011 | 304 (0.7) | 20 (1.0) | 0.166 | 203 (0.8) | 8 (0.6) | 0.712 | 3150 (0.8) | 145 (1.1) | 0.001 | 8766 (0.9) | 721 (0.8) | 0.008 |
| SLE | 1 | 571 (0.6) | 39 (0.5) | 0.215 | 60 (0.1) | 6 (0.3) | 0.124 | 43 (0.2) | 2 (0.2) | 1 | 333 (0.1) | 24 (0.2) | 0.001 | 1032 (0.1) | 72 (0.1) | 0.031 |
| Anti-Psychotic Medication | 1 | 205 (0.2) | 17 (0.2) | 1 | 85 (0.2) | 5 (0.3) | 0.796 | 73 (0.3) | 4 (0.3) | 1 | 1315 (0.3) | 52 (0.4) | 0.425 | 3711 (0.4) | 291 (0.3) | 0.014 |
| Corticosteroids | 1 | 9496 (10.6) | 1152 (15.2) | <0.001 | 878 (2.1) | 89 (4.5) | <0.001 | 1040 (4.0) | 75 (6.0) | 0.001 | 10356 (2.7) | 548 (4.0) | <0.001 | 23321 (2.5) | 2731 (3.1) | <0.001 |
| Anti-hypertensive medication | 1 | 10646 (11.9) | 1680 (22.1) | <0.001 | 2868 (6.8) | 391 (19.9) | <0.001 | 1934 (7.4) | 239 (19.2) | <0.001 | 21121 (5.4) | 2730 (20.0) | <0.001 | 196335 (20.6) | 27166 (31.3) | <0.001 |
| Family History of Heart Disease | 1 | 17861 (20.0) | 1529 (20.2) | 0.712 | 8506 (20.1) | 459 (23.4) | <0.001 | 5584 (21.4) | 339 (27.2) | <0.001 | 63434 (16.2) | 3010 (22.0) | <0.001 | 208988 (22.0) | 17756 (20.5) | <0.001 |
| Townsend Score | Mean (SD) | 5.3 (2.9) | 5.3 (2.8) | 0.783 | 5.3 (2.9) | 5.3 (2.8) | 0.972 | 5.2 (2.9) | 5.0 (2.8) | 0.049 | 5.4 (2.9) | 5.3 (2.8) | <0.001 | 5.1 (2.9) | 5.1 (2.8) | <0.001 |

Supplementary Table S4: Illustration of the expected number of cases detected and missed per 1000 people for each disease using QRISK3, Framingham Risk Score and Reynold's Risk Score

| **Score** | **Disease** | **CVD (%)** | **Sensitivity (%)** | **Expected cases per 1000 people** | **Detected cases per 1000 people** | **Missed cases per 1000 people** | **Specificity (%)** | **Incorrectly predicted cases per 1000 people** |
| --- | --- | --- | --- | --- | --- | --- | --- | --- |
| **QRISK3** | Original Score | 4.6 | 65.9 | 46 | 30 | 16 | 76.8 | 221 |
|  | Psoriasis | 4.9 | 77.6 | 49 | 38 | 11 | 70.8 | 278 |
|  | Psoriatic Arthritis | 6.8 | 74.2 | 68 | 50 | 18 | 64.9 | 327 |
|  | Rheumatoid Arthritis | 11.4 | 90.2 | 114 | 103 | 11 | 40.6 | 526 |
|  | Ankylosing Spondylitis | 6.8 | 74.6 | 68 | 51 | 17 | 69.6 | 283 |
|  | Osteoarthritis | 12.6 | 91.3 | 126 | 115 | 11 | 29.2 | 619 |
| **Framingham Risk Score** | Original Score | 13.8 | 74.7 | 138 | 103 | 35 | 64.3 | 308 |
|  | Psoriasis | 6.7 | 74 | 67 | 50 | 17 | 78.4 | 202 |
|  | Psoriatic Arthritis | 8.8 | 69.4 | 88 | 61 | 27 | 69.7 | 276 |
|  | Rheumatoid Arthritis | 16.1 | 77.7 | 161 | 125 | 36 | 58.9 | 345 |
|  | Ankylosing Spondylitis | 8.9 | 72.3 | 89 | 64 | 25 | 74.2 | 235 |
|  | Osteoarthritis | 17.3 | 85.1 | 173 | 147 | 26 | 38.3 | 510 |
| **Reynold’s Risk Score** | Psoriasis | 3.4 | 9.6 | 34 | 3 | 31 | 98.4 | 15 |
|  | Psoriatic Arthritis | 4.6 | 3.8 | 46 | 2 | 44 | 98.4 | 15 |
|  | Rheumatoid Arthritis | 7.8 | 16 | 78 | 12 | 66 | 95.4 | 42 |
|  | Ankylosing Spondylitis | 4.5 | 14.2 | 45 | 6 | 39 | 98 | 19 |
|  | Osteoarthritis | 8.4 | 15.3 | 84 | 13 | 71 | 94.5 | 50 |


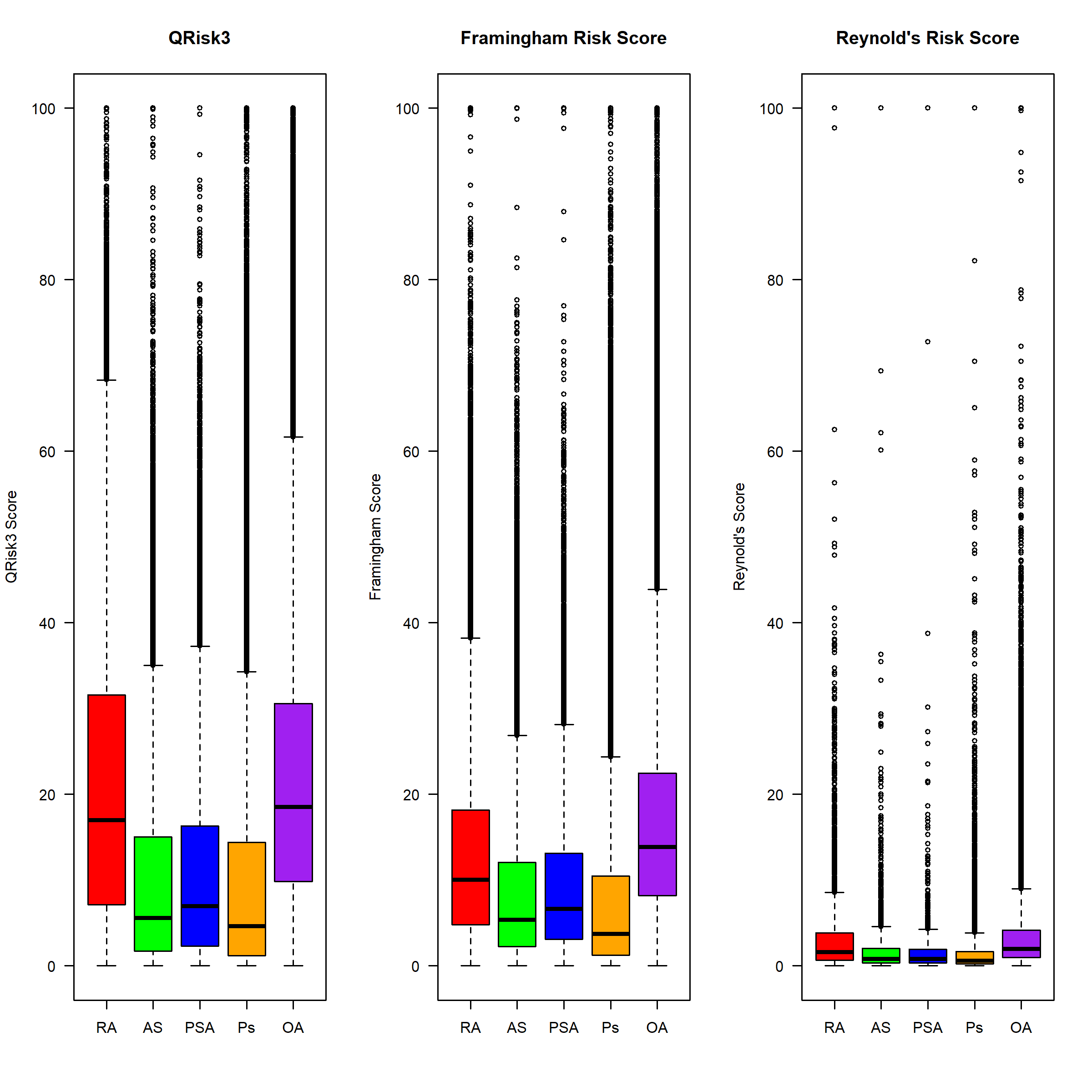


Supplementary Figure S: Boxplots showing predicted risk by disease category for each of the CVD risk tools. The results shown are from one of the 5 imputed datasets, with the results for the remaining four imputed datasets showing similar trends.


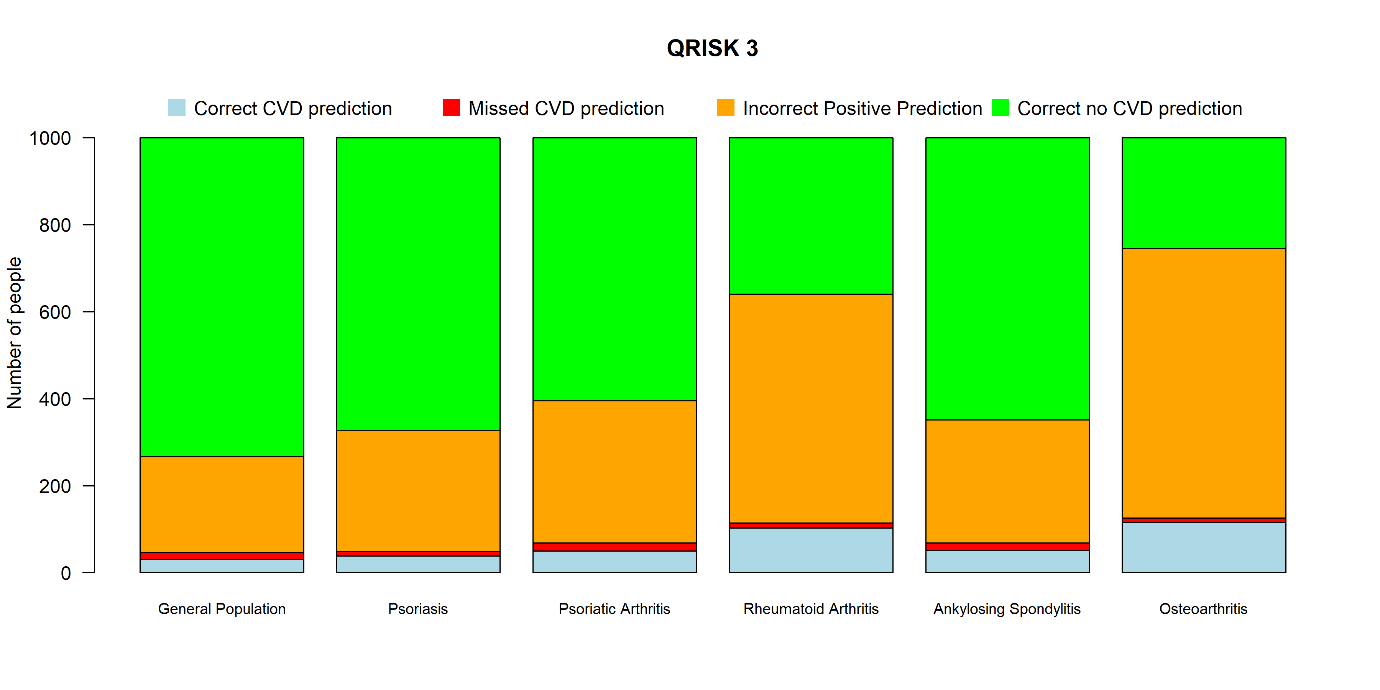


Supplementary Figure S2: Illustration of the predictive accuracy per 1000 individuals for each disease using the QRISK3 risk prediction tool.


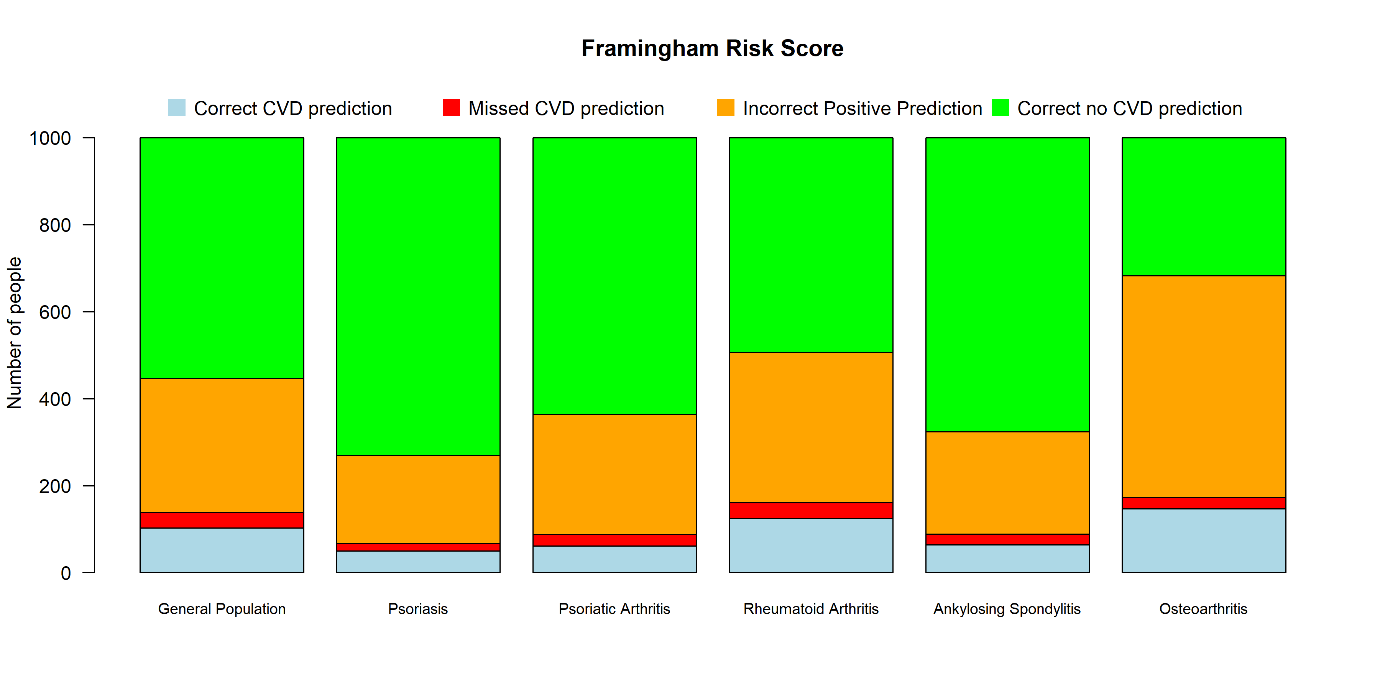


Supplementary Figure S3: Illustration of the predictive accuracy per 1000 individuals for each disease using the Framingham Risk Score prediction tool.


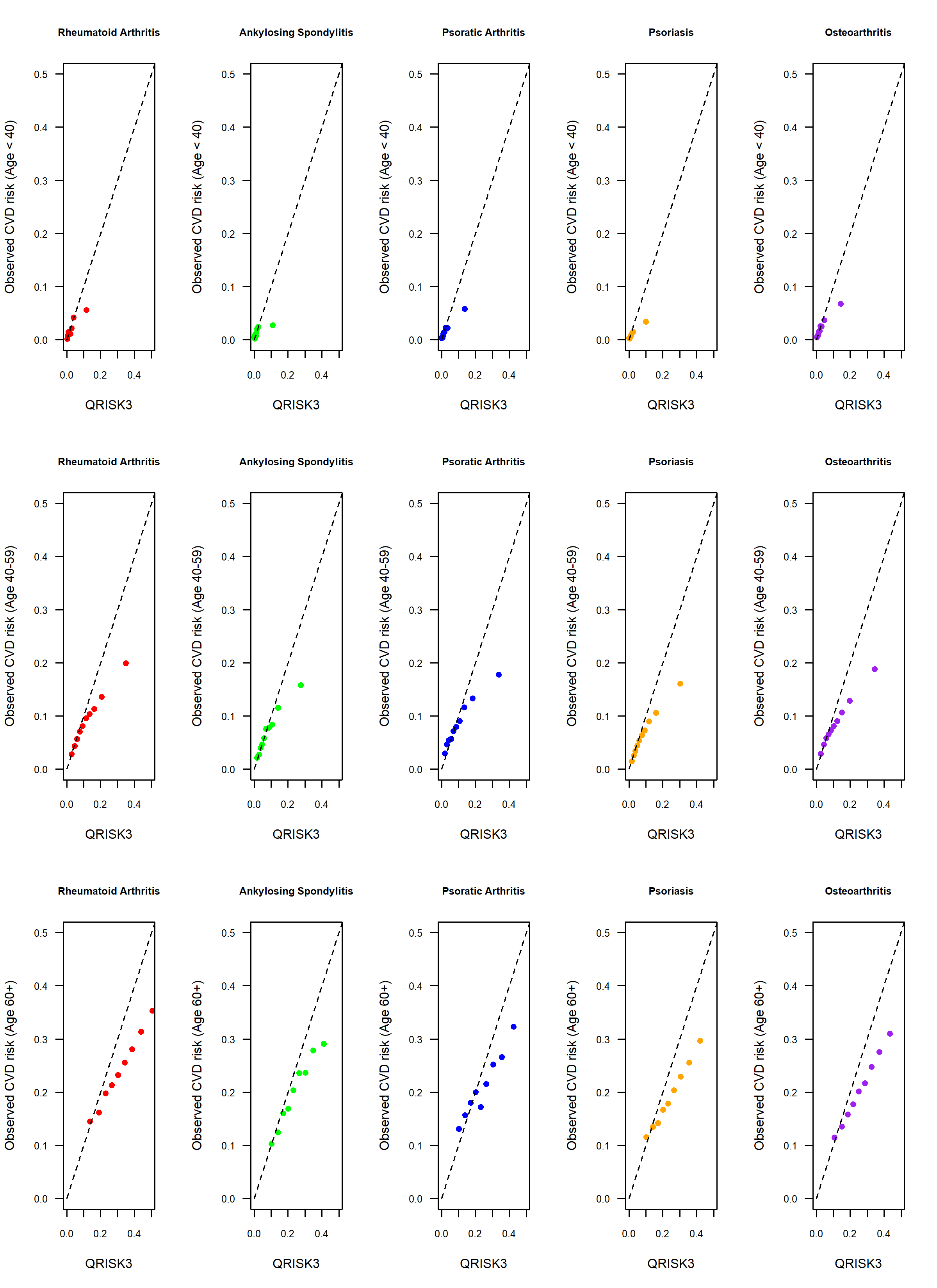


Supplementary Figure S4: Age stratified calibration plots comparing observed CVD risk in deciles of predicted CVD risk using the QRISK3 tool for each disease cohort.


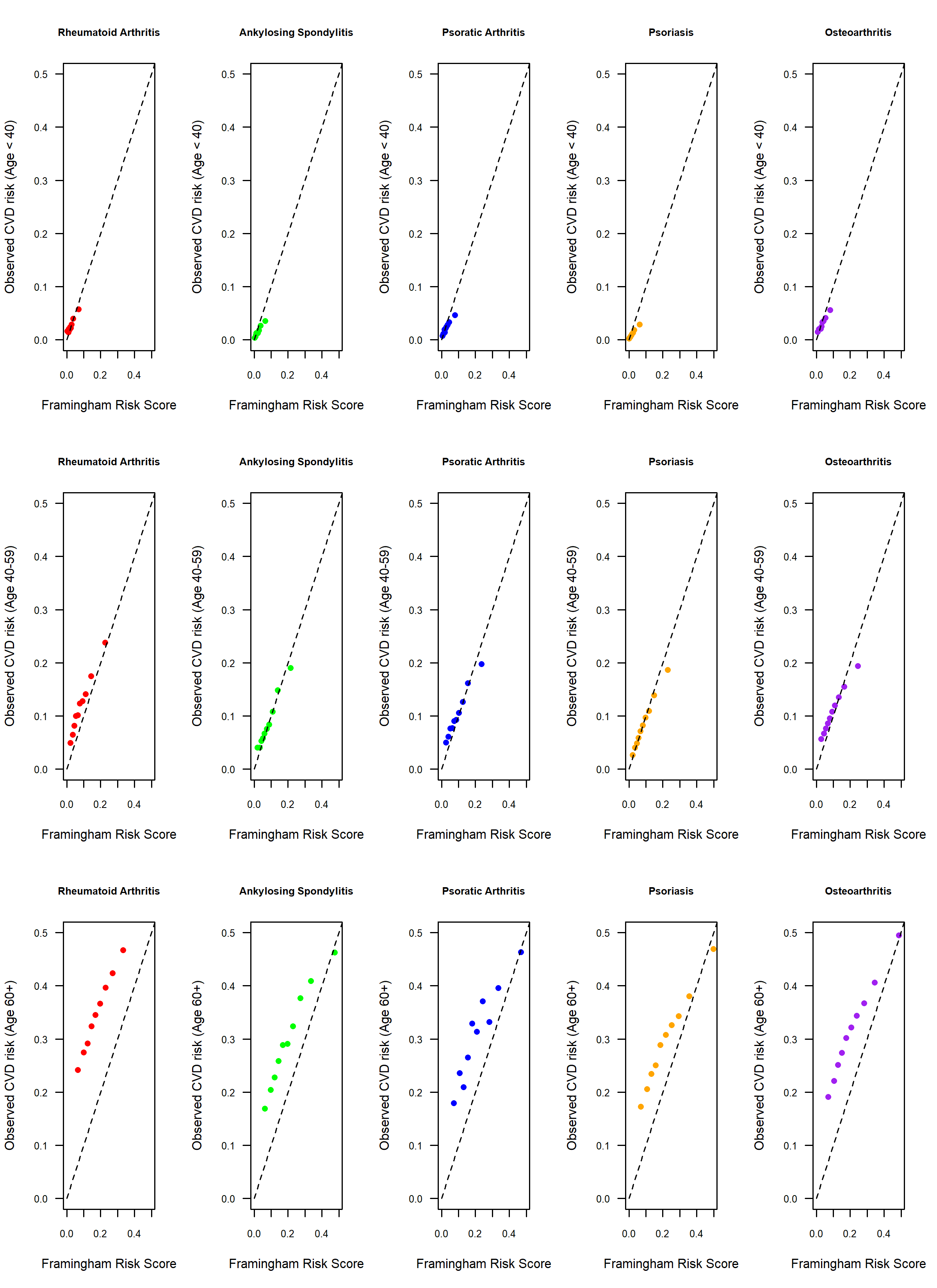


Supplementary Figure S5: Age stratified calibration plots comparing observed CVD risk in deciles of predicted CVD risk using the Framingham Risk Score for each disease cohort.


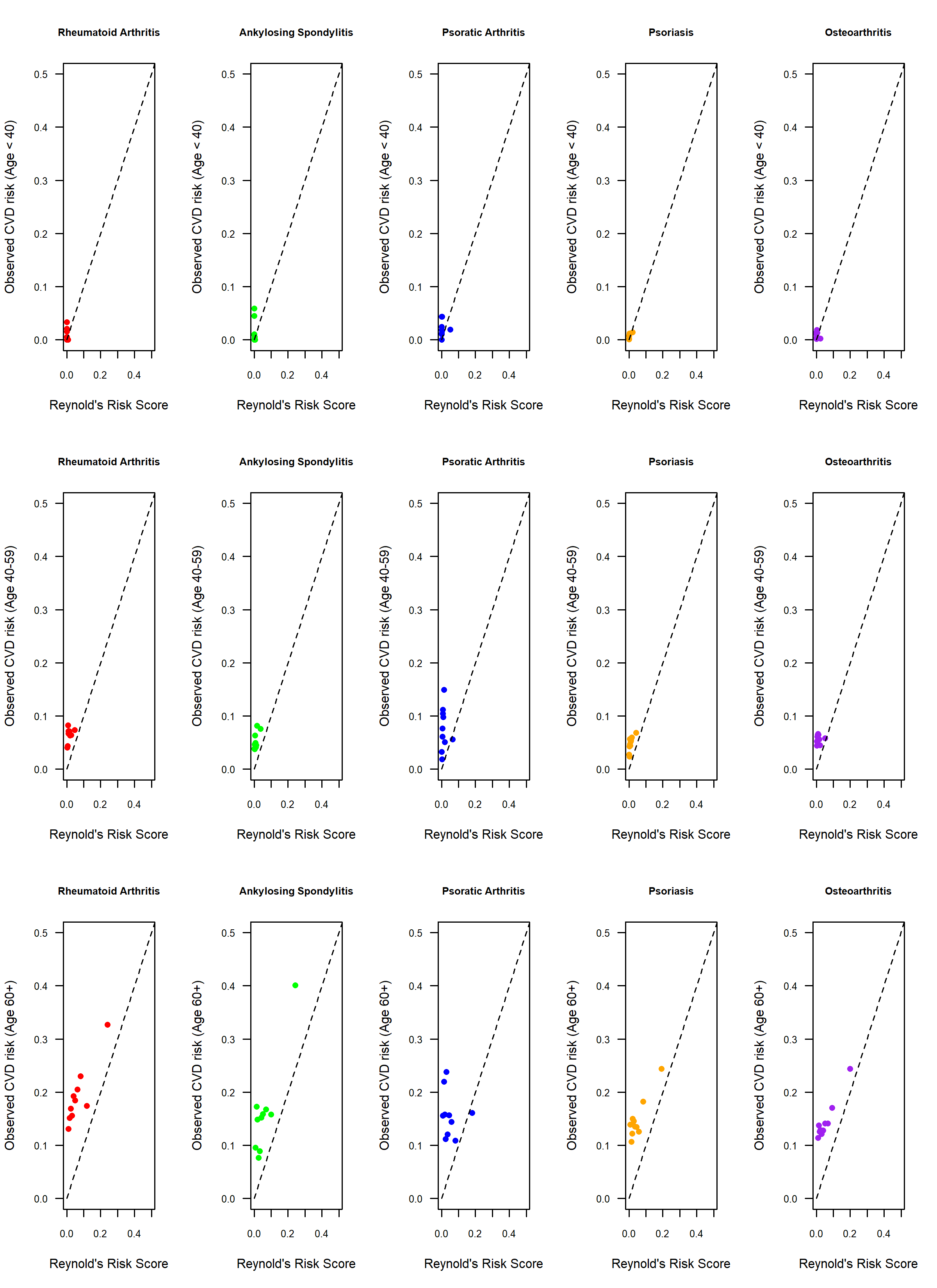


Supplementary Figure S6: Age stratified calibration plots comparing observed CVD risk in deciles of predicted CVD risk using the Reynolds’ Risk Score for each disease cohort.

**Sensitivity Analysis**

In the main analysis, we only require one code for a rheumatic condtions in order to include the individual. To be more certain of diagnoses we can require two codes. We re-ran our analysis but requiring two codes for rheumatoid arthritis, ankylosing spondylitis, psoriatic arthritis, psoriasis or osteoarthritis for inclusion. This reduced the number of patients included in each cohort (Table S5), but had very little impact on the discrimination (Table S6 and Figure S7) and calibration (Table S8) of the models.

Supplementary Table S5: Number of individuals with each condition when requiring two codes for diagnosis

|  | QRISK3 | Framingham Risk Score | Reynold’s Risk Score |
| --- | --- | --- | --- |
| Rheumatoid Arthritis | 77,349 | 76,914 | 81,357 |
| Ankylosing Spondylitis | 16,325 | 16,541 | 16,969 |
| Psoriatic Arthritis | 17,595 | 17,769 | 18,209 |
| Psoriasis | 181,033 | 182,680 | 187,044 |
| Osteoarthritis | 569,420 | 560,687 | 611,178 |

Supplementary Table S6: Prediction accuracy of each CVD risk tool at 10% and 20% thresholds, for each disease group when requiring two codes for diagnosis of rheumatic disease.

| Risk Score | Disease | AUC | 10% risk threshold | | | | 20% risk threshold | | | |
| --- | --- | --- | --- | --- | --- | --- | --- | --- | --- | --- |
|  |  |  | Sensitivity | Specificity | PPV | NPV | Sensitivity | Specificity | PPV | NPV |
| QRISK3 | RA | 0.745 | 0.898 | 0.403 | 0.205 | 0.958 | 0.700 | 0.661 | 0.261 | 0.928 |
|  | AS | 0.782 | 0.668 | 0.746 | 0.178 | 0.965 | 0.407 | 0.895 | 0.241 | 0.948 |
|  | PSA | 0.765 | 0.729 | 0.662 | 0.171 | 0.962 | 0.444 | 0.852 | 0.223 | 0.941 |
|  | Ps | 0.801 | 0.779 | 0.679 | 0.171 | 0.973 | 0.524 | 0.848 | 0.227 | 0.955 |
|  | OA | 0.686 | 0.917 | 0.265 | 0.199 | 0.942 | 0.699 | 0.573 | 0.245 | 0.905 |
| Framingham Risk Score | RA | 0.739 | 0.764 | 0.590 | 0.321 | 0.908 | 0.406 | 0.855 | 0.414 | 0.850 |
|  | AS | 0.797 | 0.696 | 0.761 | 0.233 | 0.960 | 0.347 | 0.923 | 0.319 | 0.931 |
|  | PSA | 0.758 | 0.671 | 0.712 | 0.217 | 0.948 | 0.327 | 0.915 | 0.314 | 0.920 |
|  | Ps | 0.827 | 0.740 | 0.762 | 0.233 | 0.968 | 0.405 | 0.916 | 0.318 | 0.940 |
|  | OA | 0.675 | 0.850 | 0.365 | 0.287 | 0.890 | 0.501 | 0.732 | 0.360 | 0.830 |
| Reynold’s Risk Score | RA | 0.702 | 0.164 | 0.954 | 0.274 | 0.914 | 0.056 | 0.992 | 0.443 | 0.908 |
|  | AS | 0.727 | 0.100 | 0.985 | 0.292 | 0.946 | 0.046 | 0.995 | 0.389 | 0.944 |
|  | PSA | 0.600 | 0.016 | 0.983 | 0.062 | 0.932 | 0.003 | 0.993 | 0.032 | 0.932 |
|  | Ps | 0.742 | 0.088 | 0.985 | 0.242 | 0.951 | 0.026 | 0.995 | 0.228 | 0.948 |
|  | OA | 0.626 | 0.145 | 0.943 | 0.225 | 0.906 | 0.039 | 0.987 | 0.255 | 0.900 |


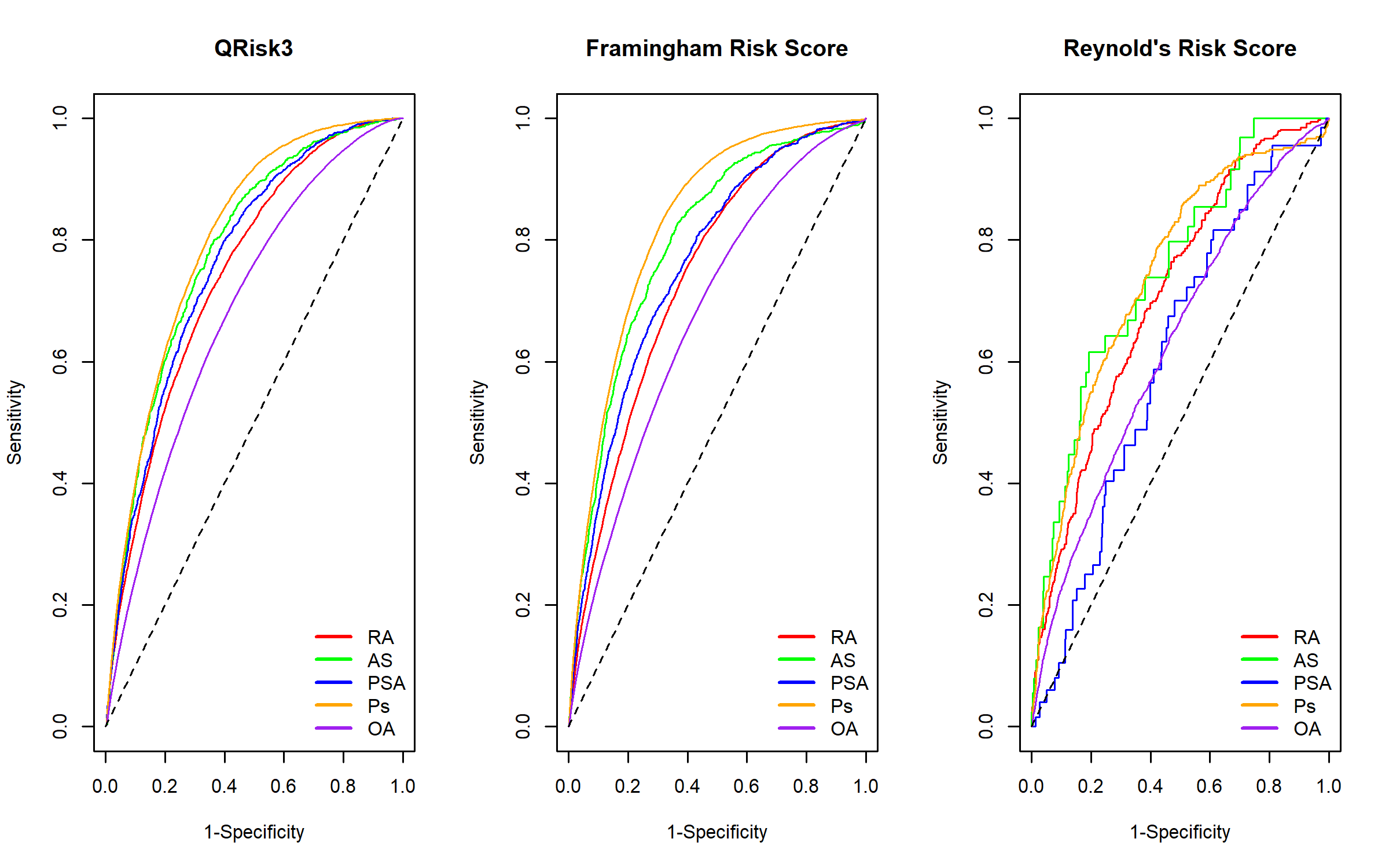


Supplementary Figure S7: ROC curves for cardiovascular risk prediction in each rheumatic disease using the QRISK-3, Framingham and Reynolds Risk Scores when requiring two codes for diagnosis of rheumatic disease.


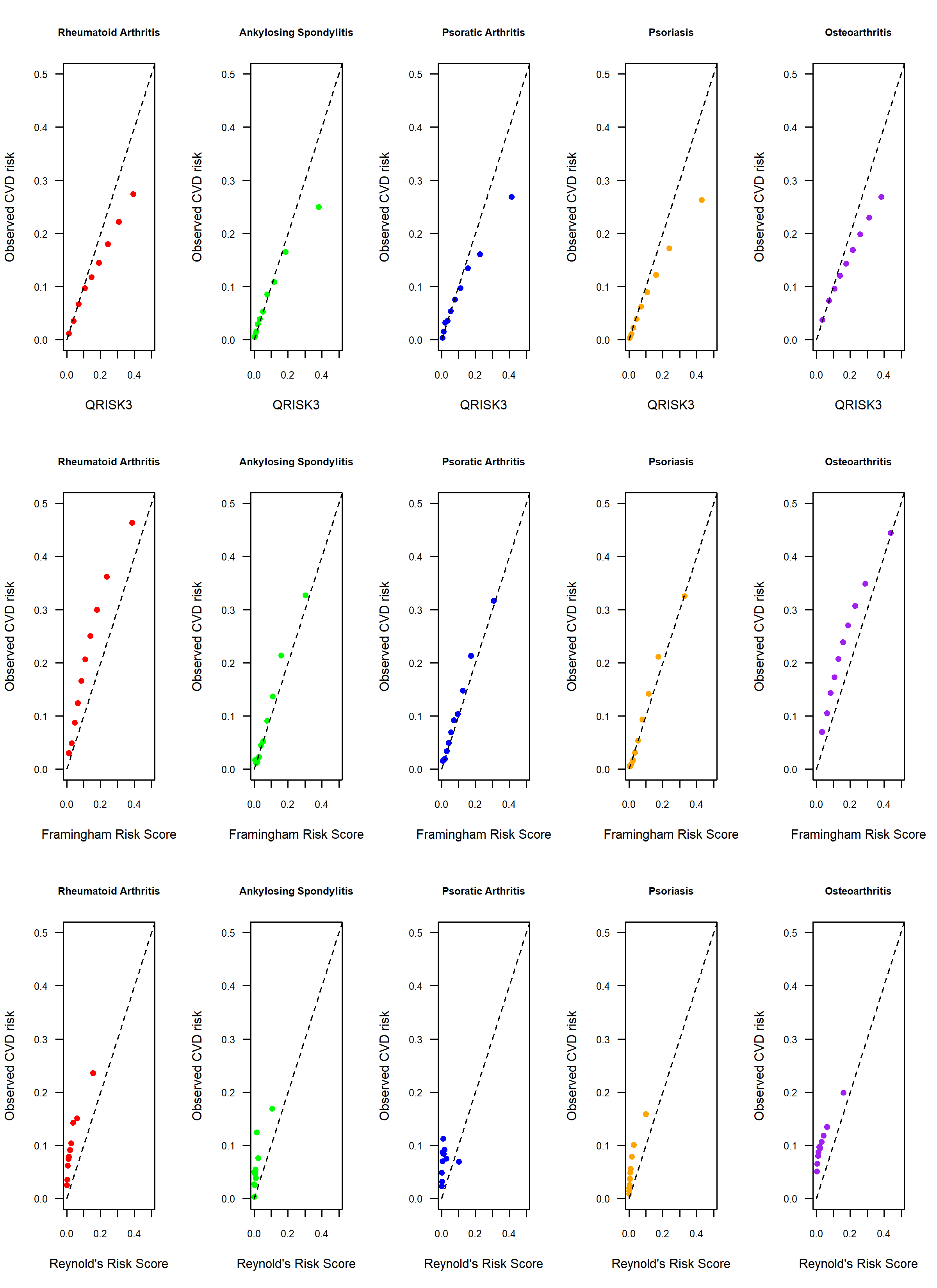


Supplementary Figure S8: Calibration plots comparing observed CVD risk in deciles of predicted CVD risk using each of the three CVD risk tools, and for each disease cohort when requiring two codes for diagnosis of rheumatic disease.

**CPRD Code Lists**

1. **Inflammatory Disease Codes**

Supplementary Table S7: Read Codes for Rheumatoid Arthritis

| MedCodeId | Term | Original Read Code | Cleansed Read Code | Snomed CT Concept Id | Snomed CT Description Id | Emis Code Category Id |
| --- | --- | --- | --- | --- | --- | --- |
| 251794010 | H/O: rheumatoid arthritis | 14G1 | 14G1.00 | 161567008 | 251794010 | 32 |
| 219021000000114 | Rheumatoid arthrit. monitoring | 66H-3 | 66H..13 | 275902004 | 411871019 | 1 |
| 2196791000000119 | Rheumatoid arthritis annual review | 66HB0 | 66HB000 | 847261000000104 | 2196791000000119 | 1 |
| 371261000000111 | Delivery of rehabilitation for rheumatoid arthritis | 7P203 | 7P20300 | 231041000000107 | 371261000000111 | 32 |
| 297544012 | Polyneuropathy in rheumatoid arthritis | F3712 | F371200 | 193180002 | 297544012 | 32 |
| 297641010 | Myopathy due to rheumatoid arthritis | F3964 | F396400 | 193250002 | 297641010 | 32 |
| 300221014 | Rheumatoid myocarditis | G5y8 | G5y8.00 | 195136004 | 300221014 | 32 |
| 48361011 | Rheumatoid carditis | G5yA | G5yA.00 | 28880005 | 48361011 | 32 |
| 162301000006117 | Rheumatoid lung | H570 | H570.00 | 398726004 | 1786545019 | 32 |
| 168751000006115 | Rheumatoid arthritis and other inflammatory polyarthropathy | N04 | N04..00 | 69896004 | 116082011 | 32 |
| 116082011 | Rheumatoid arthritis | N040 | N040.00 | 69896004 | 116082011 | 32 |
| 309787016 | Rheumatoid arthritis of cervical spine | N0400 | N040000 | 201764007 | 309787016 | 32 |
| 309788014 | Other rheumatoid arthritis of spine | N0401 | N040100 | 9631008 | 16840014 | 32 |
| 309789018 | Rheumatoid arthritis of shoulder | N0402 | N040200 | 201766009 | 309789018 | 32 |
| 309791014 | Rheumatoid arthritis of acromioclavicular joint | N0404 | N040400 | 201768005 | 309791014 | 32 |
| 309792019 | Rheumatoid arthritis of elbow | N0405 | N040500 | 201769002 | 309792019 | 32 |
| 162051000006115 | Rheumatoid arthritis of distal radio-ulnar joint | N0406 | N040600 | 201770001 | 309793012 | 32 |
| 309794018 | Rheumatoid arthritis of wrist | N0407 | N040700 | 201771002 | 309794018 | 32 |
| 162111000006117 | Rheumatoid arthritis of MCP joint | N0408 | N040800 | 201772009 | 309795017 | 32 |
| 162131000006111 | Rheumatoid arthritis of PIP joint of finger | N0409 | N040900 | 201773004 | 309796016 | 32 |
| 162041000006117 | Rheumatoid arthritis of DIP joint of finger | N040A | N040A00 | 201774005 | 309797013 | 32 |
| 309798015 | Rheumatoid arthritis of hip | N040B | N040B00 | 201775006 | 309798015 | 32 |
| 162141000006118 | Rheumatoid arthritis of sacro-iliac joint | N040C | N040C00 | 201776007 | 309799011 | 32 |
| 309800010 | Rheumatoid arthritis of knee | N040D | N040D00 | 201777003 | 309800010 | 32 |
| 309802019 | Rheumatoid arthritis of ankle | N040F | N040F00 | 201779000 | 309802019 | 32 |
| 309803012 | Rheumatoid arthritis of subtalar joint | N040G | N040G00 | 201780002 | 309803012 | 32 |
| 309804018 | Rheumatoid arthritis of talonavicular joint | N040H | N040H00 | 201781003 | 309804018 | 32 |
| 309805017 | Rheumatoid arthritis of other tarsal joint | N040J | N040J00 | 429192004 | 2696026017 | 32 |
| 168761000006118 | Rheumatoid arthritis of 1st MTP joint | N040K | N040K00 | 201783000 | 309807013 | 32 |
| 162101000006115 | Rheumatoid arthritis of lesser MTP joint | N040L | N040L00 | 201784006 | 309808015 | 32 |
| 162081000006111 | Rheumatoid arthritis of IP joint of toe | N040M | N040M00 | 201785007 | 309809011 | 32 |
| 1779323014 | Rheumatoid vasculitis | N040N | N040N00 | 400054000 | 1779323014 | 32 |
| 359292012 | Seronegative rheumatoid arthritis | N040P | N040P00 | 239792003 | 359292012 | 32 |
| 309812014 | Rheumatoid bursitis | N040Q | N040Q00 | 84017003 | 139318017 | 32 |
| 162341000006115 | Rheumatoid nodule | N040R | N040R00 | 33719002 | 485614018 | 32 |
| 426510015 | Rheumatoid arthritis - multiple joint | N040S | N040S00 | 287006005 | 426510015 | 32 |
| 309816012 | Flare of rheumatoid arthritis | N040T | N040T00 | 201791009 | 309816012 | 32 |
| 95067016 | Felty's syndrome | N041 | N041.00 | 57160007 | 95067016 | 32 |
| 16911000006114 | Other rheumatoid arthropathy + visceral/systemic involvement | N042 | N042.00 | 239793008 | 359293019 | 32 |
| 39771016 | Rheumatic carditis | N0420 | N042000 | 23685000 | 39771016 | 32 |
| 1778239014 | Rheumatoid lung disease | N0421 | N042100 | 398726004 | 1778239014 | 32 |
| 485614018 | Rheumatoid nodule | N0422 | N042200 | 33719002 | 485614018 | 32 |
| 162231000006117 | Rheumatoid arthropathy + visceral/systemic involvement NOS | N042z | N042z00 | 239793008 | 359293019 | 32 |
| 451461014 | Seropositive errosive rheumatoid arthritis | N047 | N047.00 | 308143008 | 3517964017 | 32 |
| 149911000006117 | Seropositive rheumatoid arthritis, unspecified | N04X | N04X.00 | 239791005 | 359291017 | 32 |
| 1786545019 | Rheumatoid lung | N04y0 | N04y000 | 398726004 | 1786545019 | 32 |
| 1786505011 | Caplan's syndrome | N04y0-1 | N04y011 | 398640008 | 1786506012 | 32 |
| 311496011 | Fibrosing alveolitis associated with rheumatoid arthritis | N04y0-2 | N04y012 | 10713006 | 311496011 | 32 |
| 424981000006114 | [X]Rheumatoid arthritis+involvement/other organs or systems | Nyu10 | Nyu1000 | 239793008 | 359293019 | 32 |
| 312518013 | [X]Other seropositive rheumatoid arthritis | Nyu11 | Nyu1100 | 239791005 | 359291017 | 32 |
| 312520011 | [X]Other specified rheumatoid arthritis | Nyu12 | Nyu1200 | 69896004 | 116082011 | 32 |
| 312534011 | [X]Seropositive rheumatoid arthritis, unspecified | Nyu1G | Nyu1G00 | 239791005 | 359291017 | 32 |

Supplementary Table S8: Read Codes for Ankylosing Spondylitis

| Med Code Id | Term | Original Read Code | Cleansed Read Code | Snomed CT Concept Id | Snomed CT Description Id | Emis Code Category Id |
| --- | --- | --- | --- | --- | --- | --- |
| 16833013 | Ankylosing spondylitis | N100 | N100.00 | 9631008 | 16833013 | 32 |
| 2692996011 | BASDAI - Bath ankylosing spondylitis disease activity index | 388p | 388p.00 | 441562009 | 2817026019 | 3 |
| 311040011 | Inflammatory spondylopathies | N10 | N10..00 | 202649003 | 311040011 | 32 |
| 311043013 | Inflammatory spondylopathies in diseases EC | N10y0 | N10y000 | 202652006 | 311043013 | 32 |
| 718001000006112 | Marie - Strumpell spondylitis | N100-1 | N100.11 | 9631008 | 1235977013 | 32 |
| 311042015 | Other inflammatory spondylopathies | N10y | N10y.00 | 202649003 | 311040011 | 32 |
| 359848019 | Ankylosis of spine NOS | N14z-1 | N14z.11 | 47049007 | 78420015 | 32 |
| 311044019 | Other inflammatory spondylopathies NOS | N10yz | N10yz00 | 202649003 | 311040011 | 32 |
| 311041010 | Sacroiliitis NEC | N102 | N102.00 | 55146009 | 2115291000000119 | 32 |
| 17967011 | Spinal enthesopathy | N101 | N101.00 | 10317009 | 17967011 | 32 |
| 311045018 | Spondylitis NOS | N10z | N10z.00 | 84172003 | 139589017 | 32 |
| 311283012 | Cervical spine ankylosis | N1482 | N148200 | 202814008 | 311283012 | 32 |
| 544811000006116 | Cervico-thoracic ankylosis | N1483 | N148300 | 202815009 | 311284018 | 32 |
| 311287013 | Lumbar spine ankylosis | N1486 | N148600 | 202818006 | 311287013 | 32 |
| 1235278014 | Lumbosacral ankylosis | N1460 | N146000 | 87823004 | 1235278014 | 32 |
| 311267012 | Sacral ankylosis NOS | N1462 | N146200 | 16065006 | 27193016 | 32 |
| 1221279016 | Sacroiliac ankylosis | N1461 | N146100 | 16065006 | 1221279016 | 32 |
| 311285017 | Thoracic spine ankylosis | N1484 | N148400 | 202816005 | 311285017 | 32 |
| 106931000006114 | Thoraco-lumbar ankylosis | N1485 | N148500 | 202817001 | 311286016 | 32 |

Supplementary Table S9: Read Codes for Psoriatic Arthritis

| MedCode Id | Term | Original Read Code | Cleansed Read Code | Snomed CT Concept Id | Snomed CT Description Id | Emis Code Category Id |
| --- | --- | --- | --- | --- | --- | --- |
| 55627011 | Psoriatic arthropathy | M160 | M160.00 | 33339001 | 55627011 | 32 |
| 55628018 | Psoriatic arthritis | M160-1 | M160.11 | 33339001 | 55628018 | 32 |
| 308730016 | Psoriatic arthropathy NOS | M160z | M160z00 | 33339001 | 55627011 | 32 |
| 198881000006116 | Psoriasis spondylitica | M1600 | M160000 | 200956002 | 308727011 | 32 |
| 32869011 | Arthritis mutilans | M1602 | M160200 | 19514005 | 32869011 | 32 |
| 359321011 | Distal interphalangeal psoriatic arthropathy | M1601 | M160100 | 239812005 | 359321011 | 32 |
| 312521010 | [X]Other psoriatic arthropathies | Nyu13 | Nyu1300 | 33339001 | 55626019 | 32 |

Supplementary Table S10: Read Codes for psoriasis

| MedCode Id | Term | Original Read Code | Cleansed Read Code | Snomed CT Concept Id | Snomed CT Description Id | Emis Code Category Id |
| --- | --- | --- | --- | --- | --- | --- |
| 308732012 | Psoriasis unspecified | M1610 | M161000 | 9014002 | 15886015 | 32 |
| 308753015 | Psoriasis NOS | M161z | M161z00 | 9014002 | 15886015 | 32 |
| 128227017 | Pityriasis rosea | M163 | M163.00 | 77252004 | 128227017 | 32 |
| 308725015 | Psoriasis and similar disorders | M16 | M16..00 | 9014002 | 15886015 | 32 |
| 61797015 | Guttate psoriasis | M1616 | M161600 | 37042000 | 61797015 | 32 |
| 55627011 | Psoriatic arthropathy | M160 | M160.00 | 33339001 | 55627011 | 32 |
| 357623013 | Scalp psoriasis | M16y0 | M16y000 | 238608008 | 357623013 | 32 |
| 308747015 | Pustular psoriasis | M161D | M161D00 | 200973000 | 308747015 | 32 |
| 55628018 | Psoriatic arthritis | M160-1 | M160.11 | 33339001 | 55628018 | 32 |
| 308776017 | Psoriasis and similar disorders NOS | M16z | M16z.00 | 9014002 | 15886015 | 32 |
| 1629311000006117 | Chronic large plaque psoriasis | M161F-1 | M161F11 | 402307000 | 1781467015 | 32 |
| 308745011 | Psoriasis plantaris | M161B | M161B00 | 200971003 | 308745011 | 32 |
| 308731017 | Other psoriasis | M161 | M161.00 | 9014002 | 15886015 | 32 |
| 308730016 | Psoriatic arthropathy NOS | M160z | M160z00 | 33339001 | 55627011 | 32 |
| 308744010 | Psoriasis palmaris | M161A | M161A00 | 200970002 | 308744010 | 32 |
| 308750017 | Psoriasis vulgaris | M161F | M161F00 | 200975007 | 308750017 | 32 |
| 308752013 | Erythrodermic psoriasis | M161H | M161H00 | 200977004 | 308752013 | 32 |
| 198791000006112 | Psoriasis discoidea | M1614 | M161400 | 200965009 | 308736010 | 32 |
| 483572016 | Palmoplantar pustular psoriasis | M166 | M166.00 | 27520001 | 483572016 | 32 |
| 198881000006116 | Psoriasis spondylitica | M1600 | M160000 | 200956002 | 308727011 | 32 |
| 243501000006111 | Parapsoriasis lichenoides | M1625 | M162500 | 200983001 | 308758012 | 32 |
| 308775018 | Other psoriasis and similar disorders | M16y | M16y.00 | 399937000 | 1773474010 | 32 |
| 308733019 | Psoriasis annularis | M1611 | M161100 | 200962007 | 308733019 | 32 |
| 308749017 | Psoriasis universalis | M161E | M161E00 | 200974006 | 308749017 | 32 |
| 308735014 | Psoriasis diffusa | M1613 | M161300 | 200964008 | 308735014 | 32 |
| 32869011 | Arthritis mutilans | M1602 | M160200 | 19514005 | 32869011 | 32 |
| 308746012 | Psoriasis punctata | M161C | M161C00 | 200972005 | 308746012 | 32 |
| 308734013 | Psoriasis circinata | M1612 | M161200 | 200963002 | 308734013 | 32 |
| 359321011 | Distal interphalangeal psoriatic arthropathy | M1601 | M160100 | 239812005 | 359321011 | 32 |
| 308739015 | Psoriasis geographica | M1615 | M161500 | 200966005 | 308739015 | 32 |
| 1887571000006111 | Flexural psoriasis | M161J | M161J00 | 238600001 | 357612014 | 32 |
| 308741019 | Psoriasis inveterata | M1618 | M161800 | 200968006 | 308741019 | 32 |
| 308740018 | Psoriasis gyrata | M1617 | M161700 | 200967001 | 308740018 | 32 |
| 198841000006110 | Psoriasis ostracea | M1619 | M161900 | 200969003 | 308742014 | 32 |

Supplementary Table S11: Read Codes for osteoarthritis

| MedCode Id | Term | Original Read Code | Cleansed Read Code | Snomed CT Concept Id | Snomed CT Description Id | Emis Code Category Id |
| --- | --- | --- | --- | --- | --- | --- |
| 41261000006115 | Osteoarthritis and allied disorders | N05 | N05..00 | 396275006 | 1776248011 | 32 |
| 800731000006117 | Generalised osteoarthritis - OA | N050 | N050.00 | 201819000 | 309860012 | 32 |
| 309862016 | Generalised osteoarthritis of unspecified site | N0500 | N050000 | 201819000 | 309858010 | 32 |
| 523471000006111 | Bouchards' nodes | N0501-2 | N050112 | 20243008 | 480522017 | 32 |
| 400175010 | Generalised osteoarthritis of the hand | N0501 | N050100 | 267889007 | 400175010 | 32 |
| 819391000006114 | Heberdens' nodes | N0501-1 | N050111 | 371598009 | 1210227011 | 32 |
| 800751000006112 | Generalised osteoarthritis of multiple sites | N0502 | N050200 | 201819000 | 309854012 | 32 |
| 889861000006112 | Osteoarthritis -multiple joint | N0502-99 | N050299 | 201819000 | 889861000006112 | 32 |
| 523461000006116 | Bouchard's nodes with arthropathy | N0503 | N050300 | 20243008 | 480522017 | 32 |
| 359399017 | Primary generalized osteoarthrosis | N0504 | N050400 | 239862000 | 359394010 | 32 |
| 309876015 | Secondary multiple arthrosis | N0505 | N050500 | 201825001 | 309876015 | 32 |
| 309877012 | Erosive osteoarthrosis | N0506 | N050600 | 201826000 | 309877012 | 32 |
| 309878019 | Heberden's nodes with arthropathy | N0507 | N050700 | 201827009 | 309878019 | 32 |
| 309880013 | Generalised osteoarthritis NOS | N050z | N050z00 | 201819000 | 309860012 | 32 |
| 309881012 | Localised, primary osteoarthritis | N051 | N051.00 | 201829007 | 309881012 | 32 |
| 309884016 | Localised, primary osteoarthritis of unspecified site | N0510 | N051000 | 201829007 | 309881012 | 32 |
| 309885015 | Localised, primary osteoarthritis of the shoulder region | N0511 | N051100 | 201831003 | 309885015 | 32 |
| 309888018 | Localised, primary osteoarthritis of the upper arm | N0512 | N051200 | 313259008 | 457122015 | 32 |
| 309889014 | Localised, primary osteoarthritis of the forearm | N0513 | N051300 | 313257005 | 457118013 | 32 |
| 309892013 | Localised, primary osteoarthritis of the hand | N0514 | N051400 | 201834006 | 309892013 | 32 |
| 736911000006116 | Localised, primary osteoarthritis of the pelvic region/thigh | N0515 | N051500 | 201835007 | 309893015 | 32 |
| 309896011 | Localised, primary osteoarthritis of the lower leg | N0516 | N051600 | 201829007 | 309881012 | 32 |
| 309898012 | Localised, primary osteoarthritis of the ankle and foot | N0517 | N051700 | 201837004 | 309898012 | 32 |
| 309899016 | Localised, primary osteoarthritis of other specified site | N0518 | N051800 | 201829007 | 309881012 | 32 |
| 309901013 | Primary coxarthrosis, bilateral | N0519 | N051900 | 201839001 | 309901013 | 32 |
| 309902018 | Coxarthrosis resulting from dysplasia, bilateral | N051A | N051A00 | 201840004 | 309902018 | 32 |
| 309903011 | Primary gonarthrosis, bilateral | N051B | N051B00 | 201841000 | 309903011 | 32 |
| 309904017 | Primary arthrosis of first carpometacarpal joints, bilateral | N051C | N051C00 | 201842007 | 309904017 | 32 |
| 457118013 | Localised, primary osteoarthritis of the wrist | N051D | N051D00 | 313257005 | 457118013 | 32 |
| 457121010 | Localised, primary osteoarthritis of toe | N051E | N051E00 | 313258000 | 457121010 | 32 |
| 457122015 | Localised, primary osteoarthritis of elbow | N051F | N051F00 | 313259008 | 457122015 | 32 |
| 309911018 | Localised, primary osteoarthritis NOS | N051z | N051z00 | 201829007 | 309881012 | 32 |
| 309914014 | Localised, secondary osteoarthritis | N052 | N052.00 | 201847001 | 309914014 | 32 |
| 309915010 | Localised, secondary osteoarthritis of unspecified site | N0520 | N052000 | 201847001 | 309913015 | 32 |
| 309918012 | Localised, secondary osteoarthritis of the shoulder region | N0521 | N052100 | 201849003 | 309918012 | 32 |
| 309919016 | Localised, secondary osteoarthritis of the upper arm | N0522 | N052200 | 201847001 | 309913015 | 32 |
| 309922019 | Localised, secondary osteoarthritis of the forearm | N0523 | N052300 | 201847001 | 309913015 | 32 |
| 309923012 | Localised, secondary osteoarthritis of the hand | N0524 | N052400 | 201852006 | 309923012 | 32 |
| 736171000006110 | Localised, secondary osteoarthritis of pelvic region/thigh | N0525 | N052500 | 267890003 | 400176011 | 32 |
| 309930018 | Localised, secondary osteoarthritis of the lower leg | N0526 | N052600 | 201847001 | 309913015 | 32 |
| 309933016 | Localised, secondary osteoarthritis of the ankle and foot | N0527 | N052700 | 201855008 | 309933016 | 32 |
| 309934010 | Localised, secondary osteoarthritis of other specified site | N0528 | N052800 | 201847001 | 309913015 | 32 |
| 309936012 | Post-traumatic coxarthrosis, bilateral | N0529 | N052900 | 201857000 | 309936012 | 32 |
| 309937015 | Post-traumatic gonarthrosis, bilateral | N052A | N052A00 | 201858005 | 309937015 | 32 |
| 217381000006112 | Post-traumatic arthrosis of first carpometacarpal jt bilat | N052B | N052B00 | 201859002 | 309938013 | 32 |
| 453242017 | Post-traumatic gonarthrosis, unilateral | N052C | N052C00 | 309761000 | 453242017 | 32 |
| 309941016 | Localised, secondary osteoarthritis NOS | N052z | N052z00 | 201847001 | 309914014 | 32 |
| 309942011 | Localised osteoarthritis, unspecified | N053 | N053.00 | 33952002 | 485689019 | 32 |
| 309945013 | Localised osteoarthritis, unspecified, of unspecified site | N0530 | N053000 | 33952002 | 56680019 | 32 |
| 736711000006119 | Localised osteoarthritis, unspecified, of shoulder region | N0531 | N053100 | 67315001 | 111849010 | 32 |
| 309949019 | Localised osteoarthritis, unspecified, of the upper arm | N0532 | N053200 | 33952002 | 56680019 | 32 |
| 309950019 | Localised osteoarthritis, unspecified, of the forearm | N0533 | N053300 | 43270005 | 72194019 | 32 |
| 309953017 | Localised osteoarthritis, unspecified, of the hand | N0534 | N053400 | 22193007 | 37256012 | 32 |
| 736781000006114 | Localised osteoarthritis, unspecified, pelvic region/thigh | N0535 | N053500 | 267891004 | 400178012 | 32 |
| 359434013 | Otto's pelvis | N0535-1 | N053511 | 239881008 | 359434013 | 32 |
| 400181019 | Localised osteoarthritis, unspecified, of the lower leg | N0536 | N053600 | 33952002 | 56680019 | 32 |
| 240071000006115 | Patellofemoral osteoarthritis | N0536-1 | N053611 | 450521003 | 2915470018 | 32 |
| 309965018 | Localised osteoarthritis, unspecified, of the ankle and foot | N0537 | N053700 | 201837004 | 1476420018 | 32 |
| 736701000006117 | Localised osteoarthritis, unspecified, of other spec site | N0538 | N053800 | 33952002 | 56680019 | 32 |
| 309969012 | Arthrosis of first carpometacarpal joint, unspecified | N0539 | N053900 | 37895003 | 63223018 | 32 |
| 309971012 | Localised osteoarthritis, unspecified, NOS | N053z | N053z00 | 33952002 | 56680019 | 32 |
| 309972017 | Oligoarticular osteoarthritis, unspecified | N054 | N054.00 | 268054009 | 2692892014 | 32 |
| 265841000006115 | Oligoarticular osteoarthritis, unspec, of unspecified sites | N0540 | N054000 | 268054009 | 2692892014 | 32 |
| 264481000006111 | Oligoarticular osteoarthritis, unspecified, of shoulder | N0541 | N054100 | 396275006 | 1776248011 | 32 |
| 309975015 | Oligoarticular osteoarthritis, unspecified, of upper arm | N0542 | N054200 | 396275006 | 1776248011 | 32 |
| 309976019 | Oligoarticular osteoarthritis, unspecified, of forearm | N0543 | N054300 | 396275006 | 1776248011 | 32 |
| 309977011 | Oligoarticular osteoarthritis, unspecified, of hand | N0544 | N054400 | 22193007 | 37256012 | 32 |
| 264471000006113 | Oligoarticular osteoarthritis, unspecified, of pelvis/thigh | N0545 | N054500 | 396275006 | 1776248011 | 32 |
| 309979014 | Oligoarticular osteoarthritis, unspecified, of lower leg | N0546 | N054600 | 396275006 | 1776248011 | 32 |
| 265871000006111 | Oligoarticular osteoarthritis, unspecified, of ankle/foot | N0547 | N054700 | 396275006 | 1776248011 | 32 |
| 264501000006118 | Oligoarticular osteoarthritis, unspecified, other spec sites | N0548 | N054800 | 396275006 | 1776248011 | 32 |
| 265861000006116 | Oligoarticular osteoarthritis, unspecified, multiple sites | N0549 | N054900 | 268054009 | 2692892014 | 32 |
| 309983014 | Osteoarthritis of more than one site, unspecified, NOS | N054z | N054z00 | 268054009 | 2692892014 | 32 |
| 757071000006113 | Joint degeneration | N05z-1 | N05z.11 | 396275006 | 1776248011 | 32 |
| 41281000006113 | Osteoarthritis NOS | N05z | N05z.00 | 396275006 | 1776248011 | 32 |
| 309987010 | Osteoarthritis NOS, of unspecified site | N05z0 | N05z000 | 396275006 | 1776248011 | 32 |
| 889871000006117 | Osteoarthritis -shoulder joint | N05z1-99 | N05z199 | 666441000000103 | 889871000006117 | 32 |
| 309988017 | Osteoarthritis NOS, of shoulder region | N05z1 | N05z100 | 67315001 | 111849010 | 32 |
| 221491000000111 | Elbow osteoarthritis NOS | N05z2-1 | N05z211 | 239866002 | 359409013 | 32 |
| 889881000006119 | Osteoarthritis - elbow joint | N05z2-99 | N05z299 | 396275006 | 889881000006119 | 32 |
| 400183016 | Osteoarthritis NOS, of the upper arm | N05z2 | N05z200 | 396275006 | 1776248011 | 32 |
| 889891000006116 | Osteoarthritis - wrist joint | N05z3-99 | N05z399 | 396275006 | 889891000006116 | 32 |
| 400184010 | Osteoarthritis NOS, of the forearm | N05z3 | N05z300 | 396275006 | 1776248011 | 32 |
| 54641000006115 | Wrist osteoarthritis NOS | N05z3-1 | N05z311 | 239867006 | 359412011 | 32 |
| 359389010 | Finger osteoarthritis NOS | N05z4-1 | N05z411 | 239868001 | 2920945011 | 32 |
| 889901000006117 | Osteoarthritis - hand joint | N05z4-99 | N05z499 | 396275006 | 889901000006117 | 32 |
| 400185011 | Osteoarthritis NOS, of the hand | N05z4 | N05z400 | 22193007 | 37256012 | 32 |
| 359388019 | Thumb osteoarthritis NOS | N05z4-2 | N05z412 | 37895003 | 63223018 | 32 |
| 310020013 | Osteoarthritis NOS, of hip | N05zJ | N05zJ00 | 239872002 | 359419019 | 32 |
| 221511000000115 | Hip osteoarthritis NOS | N05z5-1 | N05z511 | 239872002 | 359419019 | 32 |
| 889911000006119 | Osteoarthritis - hip joint | N05z5-99 | N05z599 | 396275006 | 889911000006119 | 32 |
| 400186012 | Osteoarthritis NOS, pelvic region/thigh | N05z5 | N05z500 | 445478004 | 2871421010 | 32 |
| 221521000000114 | Knee osteoarthritis NOS | N05z6-1 | N05z611 | 239873007 | 359420013 | 32 |
| 889921000006110 | Osteoarthritis - knee joint | N05z6-99 | N05z699 | 637091000000105 | 889921000006110 | 32 |
| 400187015 | Osteoarthritis NOS, of the lower leg | N05z6 | N05z600 | 396275006 | 1776248011 | 32 |
| 359385016 | Ankle osteoarthritis NOS | N05z7-1 | N05z711 | 239874001 | 359423010 | 32 |
| 359384017 | Foot osteoarthritis NOS | N05z7-2 | N05z712 | 309246000 | 452614016 | 32 |
| 889931000006113 | Osteoarthritis - ankle/foot | N05z7-99 | N05z799 | 396275006 | 889931000006113 | 32 |
| 400188013 | Osteoarthritis NOS, of ankle and foot | N05z7 | N05z700 | 201837004 | 1476420018 | 32 |
| 359383011 | Toe osteoarthritis NOS | N05z7-3 | N05z713 | 239878003 | 359429014 | 32 |
| 400188013 | Osteoarthritis NOS, of ankle and foot | N05z7 | N05z700 | 201837004 | 1476420018 | 32 |
| 889941000006115 | Osteoarthritis - other joint | N05z8-99 | N05z899 | 666451000000100 | 889941000006115 | 32 |
| 310010015 | Osteoarthritis NOS, other specified site | N05z8 | N05z800 | 396275006 | 1776248011 | 32 |
| 310011016 | Osteoarthritis NOS, of shoulder | N05z9 | N05z900 | 67315001 | 1232920019 | 32 |
| 310012011 | Osteoarthritis NOS, of sternoclavicular joint | N05zA | N05zA00 | 850791000000102 | 2204391000000113 | 32 |
| 310013018 | Osteoarthritis NOS, of acromioclavicular joint | N05zB | N05zB00 | 239865003 | 359408017 | 32 |
| 310014012 | Osteoarthritis NOS, of elbow | N05zC | N05zC00 | 239866002 | 359409013 | 32 |
| 31041000006113 | Osteoarthritis NOS, of distal radio-ulnar joint | N05zD | N05zD00 | 396275006 | 1776248011 | 32 |
| 310016014 | Osteoarthritis NOS, of wrist | N05zE | N05zE00 | 239867006 | 359412011 | 32 |
| 31101000006110 | Osteoarthritis NOS, of MCP joint | N05zF | N05zF00 | 275324008 | 411290018 | 32 |
| 18051000006110 | Osteoarthritis NOS, of PIP joint of finger | N05zG | N05zG00 | 239870005 | 359416014 | 32 |
| 31031000006115 | Osteoarthritis NOS, of DIP joint of finger | N05zH | N05zH00 | 239869009 | 359415013 | 32 |
| 310020013 | Osteoarthritis NOS, of hip | N05zJ | N05zJ00 | 239872002 | 359419019 | 32 |
| 18061000006112 | Osteoarthritis NOS, of sacro-iliac joint | N05zK | N05zK00 | 55146009 | 91681011 | 32 |
| 310024016 | Osteoarthritis NOS, of knee | N05zL | N05zL00 | 239873007 | 359420013 | 32 |
| 18121000006117 | Osteoarthritis NOS, of tibio-fibular joint | N05zM | N05zM00 | 396275006 | 1776248011 | 32 |
| 310026019 | Osteoarthritis NOS, of ankle | N05zN | N05zN00 | 239874001 | 359423010 | 32 |
| 310027011 | Osteoarthritis NOS, of subtalar joint | N05zP | N05zP00 | 239876004 | 359426019 | 32 |
| 310028018 | Osteoarthritis NOS, of talonavicular joint | N05zQ | N05zQ00 | 700324005 | 2989803011 | 32 |
| 310029014 | Osteoarthritis NOS, of other tarsal joint | N05zR | N05zR00 | 396275006 | 1776248011 | 32 |
| 41301000006112 | Osteoarthritis NOS, of 1st MTP joint | N05zS | N05zS00 | 239877008 | 2983768015 | 32 |
| 31091000006116 | Osteoarthritis NOS, of lesser MTP joint | N05zT | N05zT00 | 239878003 | 359429014 | 32 |
| 31071000006117 | Osteoarthritis NOS, of IP joint of toe | N05zU | N05zU00 | 239878003 | 359429014 | 32 |
| 400182014 | Osteoarthritis NOS | N05zz | N05zz00 | 396275006 | 1776248011 | 32 |

1. **CVD Risk Codes**

Supplementary Table S12: Read Codes for QRISK3 CVD events

| MedCode Id | Term | Original Read Code | Cleansed Read Code | Snomed CT Concept Id | Snomed CT Description Id | Emis Code Category Id |
| --- | --- | --- | --- | --- | --- | --- |
| 2534664018 | Ischaemic heart disease | G3 | G3...00 | 414545008 | 2534664018 | 32 |
| 2536393012 | Arteriosclerotic heart disease | G3-1 | G3...11 | 443502000 | 2842265012 | 32 |
| 89332015 | Atherosclerotic heart disease | G3-2 | G3...12 | 443502000 | 2842265012 | 32 |
| 2537480011 | IHD - Ischaemic heart disease | G3-3 | G3...13 | 414545008 | 2537480011 | 32 |
| 94884017 | Acute myocardial infarction | G30 | G30..00 | 57054005 | 94884017 | 32 |
| 219521000000119 | Attack - heart | G30-1 | G30..11 | 22298006 | 37443015 | 32 |
| 1786197015 | Coronary thrombosis | G30-2 | G30..12 | 398274000 | 1786197015 | 32 |
| 537751000006115 | Cardiac rupture following myocardial infarction (MI) | G30-3 | G30..13 | 233847009 | 350383019 | 32 |
| 37443015 | Heart attack | G30-4 | G30..14 | 22298006 | 37443015 | 32 |
| 219531000000117 | MI - acute myocardial infarction | G30-5 | G30..15 | 57054005 | 94884017 | 32 |
| 1786198013 | Thrombosis - coronary | G30-6 | G30..16 | 398274000 | 1786198013 | 32 |
| 350376014 | Silent myocardial infarction | G30-7 | G30..17 | 233843008 | 350376014 | 32 |
| 884141000006116 | Coronary thrombosis | G30-98 | G30..98 | 57054005 | 884141000006116 | 32 |
| 884151000006119 | Myocardial Infarction | G30-99 | G30..99 | 57054005 | 884151000006119 | 32 |
| 455641000006112 | Acute anterolateral infarction | G300 | G300.00 | 70211005 | 1233238016 | 32 |
| 299707016 | Other specified anterior myocardial infarction | G301 | G301.00 | 54329005 | 1231324017 | 32 |
| 299708014 | Acute anteroapical infarction | G3010 | G301000 | 52035003 | 3038718019 | 32 |
| 455651000006114 | Acute anteroseptal infarction | G3011 | G301100 | 62695002 | 104192010 | 32 |
| 299709018 | Anterior myocardial infarction NOS | G301z | G301z00 | 54329005 | 1231324017 | 32 |
| 457531000006110 | Acute inferolateral infarction | G302 | G302.00 | 65547006 | 1232697013 | 32 |
| 1234005010 | Acute inferoposterior infarction | G303 | G303.00 | 76593002 | 1234005010 | 32 |
| 299710011 | Posterior myocardial infarction NOS | G304 | G304.00 | 233838001 | 350371016 | 32 |
| 299711010 | Lateral myocardial infarction NOS | G305 | G305.00 | 58612006 | 1231860015 | 32 |
| 299712015 | True posterior myocardial infarction | G306 | G306.00 | 194802003 | 299712015 | 32 |
| 116992017 | Acute subendocardial infarction | G307 | G307.00 | 70422006 | 116992017 | 32 |
| 450322013 | Acute non-Q wave infarction | G3070 | G307000 | 307140009 | 450322013 | 32 |
| 1780501013 | Acute non-ST segment elevation myocardial infarction | G3071 | G307100 | 401314000 | 1780501013 | 32 |
| 299714019 | Inferior myocardial infarction NOS | G308 | G308.00 | 73795002 | 122557015 | 32 |
| 447324018 | Acute Q-wave infarct | G309 | G309.00 | 304914007 | 447324018 | 32 |
| 1235655012 | Mural thrombosis | G30A | G30A.00 | 91335003 | 1235655012 | 32 |
| 967931000006114 | Acute posterolateral myocardial infarction | G30B | G30B.00 | 15990001 | 27071012 | 32 |
| 460681000006116 | Acute transmural myocardial infarction of unspecif site | G30X | G30X.00 | 57054005 | 94884017 | 32 |
| 1780491019 | Acute ST segment elevation myocardial infarction | G30X0 | G30X000 | 401303003 | 1780491019 | 32 |
| 299718016 | Other acute myocardial infarction | G30y | G30y.00 | 57054005 | 94884017 | 32 |
| 299719012 | Acute atrial infarction | G30y0 | G30y000 | 194809007 | 299719012 | 32 |
| 1218860015 | Acute papillary muscle infarction | G30y1 | G30y100 | 10273003 | 1218860015 | 32 |
| 1234306015 | Acute septal infarction | G30y2 | G30y200 | 79009004 | 1234306015 | 32 |
| 299720018 | Other acute myocardial infarction NOS | G30yz | G30yz00 | 57054005 | 94884017 | 32 |
| 299721019 | Acute myocardial infarction NOS | G30z | G30z.00 | 57054005 | 94884017 | 32 |
| 39111000006114 | Other acute and subacute ischaemic heart disease | G31 | G31..00 | 414545008 | 2534663012 | 32 |
| 884161000006117 | Acute/subacute IHD NOS | G31-99 | G31..99 | 643861000000100 | 884161000006117 | 32 |
| 109915012 | Postmyocardial infarction syndrome | G310 | G310.00 | 66189004 | 500341014 | 32 |
| 109916013 | Dressler's syndrome | G310-1 | G310.11 | 66189004 | 109916013 | 32 |
| 7844010 | Preinfarction syndrome | G311 | G311.00 | 4557003 | 494262012 | 32 |
| 7847015 | Crescendo angina | G311-1 | G311.11 | 4557003 | 7847015 | 32 |
| 494260016 | Impending infarction | G311-2 | G311.12 | 25106000 | 42087013 | 32 |
| 72571000006115 | Unstable angina | G311-3 | G311.13 | 4557003 | 7845011 | 32 |
| 482811000006113 | Angina at rest | G311-4 | G311.14 | 59021001 | 498328016 | 32 |
| 682481000006118 | Myocardial infarction aborted | G3110 | G311000 | 194821006 | 2619484018 | 32 |
| 2619484018 | MI - Myocardial infarction aborted | G3110-1 | G311011 | 194821006 | 2619484018 | 32 |
| 7845011 | Unstable angina | G3111 | G311100 | 4557003 | 7845011 | 32 |
| 498328016 | Angina at rest | G3112 | G311200 | 59021001 | 498328016 | 32 |
| 459487012 | Refractory angina | G3113 | G311300 | 315025001 | 459487012 | 32 |
| 494261017 | Worsening angina | G3114 | G311400 | 4557003 | 494261017 | 32 |
| 1488382011 | Acute coronary syndrome | G3115 | G311500 | 394659003 | 1488382011 | 32 |
| 299741012 | Preinfarction syndrome NOS | G311z | G311z00 | 4557003 | 7844010 | 32 |
| 299742017 | Coronary thrombosis not resulting in myocardial infarction | G312 | G312.00 | 194821006 | 299742017 | 32 |
| 299723016 | Other acute and subacute ischaemic heart disease | G31y | G31y.00 | 414545008 | 2534663012 | 32 |
| 299745015 | Acute coronary insufficiency | G31y0 | G31y000 | 194823009 | 299745015 | 32 |
| 931961000006117 | Acute coronary syndrome | G31y0-99 | G31y099 | 194823009 | 931961000006117 | 32 |
| 1229885017 | Microinfarction of heart | G31y1 | G31y100 | 42531007 | 1229885017 | 32 |
| 494438016 | Subendocardial ischaemia | G31y2 | G31y200 | 46109009 | 494438016 | 32 |
| 459488019 | Transient myocardial ischaemia | G31y3 | G31y300 | 315026000 | 459488019 | 32 |
| 299750014 | Other acute and subacute ischaemic heart disease NOS | G31yz | G31yz00 | 414545008 | 2534663012 | 32 |
| 4031011 | Old myocardial infarction | G32 | G32..00 | 1755008 | 4031011 | 32 |
| 4032016 | Healed myocardial infarction | G32-1 | G32..11 | 1755008 | 4032016 | 32 |
| 230021000006115 | Personal history of myocardial infarction | G32-2 | G32..12 | 399211009 | 2986492018 | 32 |
| 299757012 | Angina pectoris | G33 | G33..00 | 194828000 | 299757012 | 32 |
| 98087016 | Angina decubitus | G330 | G330.00 | 59021001 | 98087016 | 32 |
| 59952018 | Nocturnal angina | G3300 | G330000 | 35928006 | 59952018 | 32 |
| 299763015 | Angina decubitus NOS | G330z | G330z00 | 59021001 | 98087016 | 32 |
| 1235225010 | Prinzmetal's angina | G331 | G331.00 | 87343002 | 1235225010 | 32 |
| 144819018 | Variant angina pectoris | G331-1 | G331.11 | 87343002 | 1235225010 | 32 |
| 39773018 | Coronary artery spasm | G332 | G332.00 | 23687008 | 39773018 | 32 |
| 482941000006119 | Angina pectoris NOS | G33z | G33z.00 | 194828000 | 299757012 | 32 |
| 32122016 | Status anginosus | G33z0 | G33z000 | 19057007 | 32122016 | 32 |
| 299758019 | Stenocardia | G33z1 | G33z100 | 194828000 | 299758019 | 32 |
| 36036010 | Syncope anginosa | G33z2 | G33z200 | 21470009 | 36036010 | 32 |
| 442204010 | Angina on effort | G33z3 | G33z300 | 300995000 | 442204010 | 32 |
| 338974012 | Ischaemic chest pain | G33z4 | G33z400 | 225566008 | 338974012 | 32 |
| 458410010 | Post infarct angina | G33z5 | G33z500 | 314116003 | 458410010 | 32 |
| 350350014 | New onset angina | G33z6 | G33z600 | 233821000 | 350350014 | 32 |
| 350348018 | Stable angina | G33z7 | G33z700 | 233819005 | 350348018 | 32 |
| 299765010 | Angina pectoris NOS | G33zz | G33zz00 | 194828000 | 299757012 | 32 |
| 299776014 | Other chronic ischaemic heart disease | G34 | G34..00 | 413838009 | 2534672016 | 32 |
| 884171000006112 | Chr. ischaemic heart dis. NOS | G34-99 | G34..99 | 621601000000104 | 884171000006112 | 32 |
| 595731000006114 | Coronary atherosclerosis | G340 | G340.00 | 443502000 | 2842265012 | 32 |
| 350346019 | Triple vessel disease of the heart | G340-1 | G340.11 | 233817007 | 350346019 | 32 |
| 2536395017 | Coronary artery disease | G340-2 | G340.12 | 53741008 | 2536395017 | 32 |
| 299782012 | Single coronary vessel disease | G3400 | G340000 | 194842008 | 299782012 | 32 |
| 299783019 | Double coronary vessel disease | G3401 | G340100 | 194843003 | 299783019 | 32 |
| 496991000006111 | Atherosclerotic cardiovascular disease | G342 | G342.00 | 72092001 | 119795011 | 32 |
| 299796018 | Ischaemic cardiomyopathy | G343 | G343.00 | 194849004 | 299796018 | 32 |
| 350354017 | Silent myocardial ischaemia | G344 | G344.00 | 233823002 | 350354017 | 32 |
| 299800012 | Other specified chronic ischaemic heart disease | G34y | G34y.00 | 413838009 | 2534671011 | 32 |
| 2537483013 | Chronic coronary insufficiency | G34y0 | G34y000 | 413844008 | 2537483013 | 32 |
| 2534674015 | Chronic myocardial ischaemia | G34y1 | G34y100 | 413844008 | 2534674015 | 32 |
| 299804015 | Other specified chronic ischaemic heart disease NOS | G34yz | G34yz00 | 413838009 | 2534671011 | 32 |
| 299805019 | Other chronic ischaemic heart disease NOS | G34z | G34z.00 | 413838009 | 2534672016 | 32 |
| 459859010 | Asymptomatic coronary heart disease | G34z0 | G34z000 | 315348000 | 459859010 | 32 |
| 299808017 | Subsequent myocardial infarction | G35 | G35..00 | 194856005 | 299808017 | 32 |
| 299811016 | Subsequent myocardial infarction of anterior wall | G350 | G350.00 | 194857001 | 299811016 | 32 |
| 299812011 | Subsequent myocardial infarction of inferior wall | G351 | G351.00 | 194858006 | 299812011 | 32 |
| 299813018 | Subsequent myocardial infarction of other sites | G353 | G353.00 | 194856005 | 299808017 | 32 |
| 118831000006118 | Subsequent myocardial infarction of unspecified site | G35X | G35X.00 | 194856005 | 299808017 | 32 |
| 543291000006110 | Certain current complication follow acute myocardial infarct | G36 | G36..00 | 371068009 | 1209776017 | 32 |
| 813961000006116 | Haemopericardium/current comp folow acut myocard infarct | G360 | G360.00 | 194862000 | 299816014 | 32 |
| 498031000006112 | Atrial septal defect/curr comp folow acut myocardal infarct | G361 | G361.00 | 194863005 | 299818010 | 32 |
| 67081000006119 | Ventric septal defect/curr comp fol acut myocardal infarctn | G362 | G362.00 | 233846000 | 350381017 | 32 |
| 158601000006116 | Ruptur cardiac wall w'out haemopericard/cur comp fol ac MI | G363 | G363.00 | 194865003 | 299821012 | 32 |
| 158611000006118 | Ruptur chordae tendinae/curr comp fol acute myocard infarct | G364 | G364.00 | 194866002 | 299822017 | 32 |
| 159001000006119 | Rupture papillary muscle/curr comp fol acute myocard infarct | G365 | G365.00 | 194867006 | 299823010 | 32 |
| 100681000006116 | Thrombosis atrium,auric append&vent/curr comp foll acute MI | G366 | G366.00 | 194868001 | 299824016 | 32 |
| 208365015 | Postoperative myocardial infarction | G38 | G38..00 | 129574000 | 208365015 | 32 |
| 212061000006119 | Postoperative transmural myocardial infarction anterior wall | G380 | G380.00 | 311792005 | 455418016 | 32 |
| 212071000006114 | Postoperative transmural myocardial infarction inferior wall | G381 | G381.00 | 311793000 | 455419012 | 32 |
| 212081000006112 | Postoperative transmural myocardial infarction other sites | G382 | G382.00 | 129574000 | 208365015 | 32 |
| 212091000006110 | Postoperative transmural myocardial infarction unspec site | G383 | G383.00 | 129574000 | 208365015 | 32 |
| 455422014 | Postoperative subendocardial myocardial infarction | G384 | G384.00 | 311796008 | 455422014 | 32 |
| 455423016 | Postoperative myocardial infarction, unspecified | G38z | G38z.00 | 129574000 | 208365015 | 32 |
| 299834013 | Other specified ischaemic heart disease | G3y | G3y..00 | 414545008 | 2534664018 | 32 |
| 299835014 | Ischaemic heart disease NOS | G3z | G3z..00 | 414545008 | 2534664018 | 32 |
| 216351000006118 | Post infarction pericarditis | G501 | G501.00 | 233885007 | 350431013 | 32 |
| 362461000006119 | [X]Acute transmural myocardial infarction of unspecif site | Gyu34 | Gyu3400 | 57054005 | 94884017 | 32 |
| 145925010 | Amaurosis fugax | F4236 | F423600 | 88032003 | 145925010 | 32 |
| 416991000006112 | [X]Other transnt cerebral ischaemic attacks+related syndroms | Fyu55 | Fyu5500 | 266257000 | 395788015 | 32 |
| 300312010 | Cerebral infarct due to thrombosis of precerebral arteries | G63y0 | G63y000 | 195185009 | 300312010 | 32 |
| 300313017 | Cerebral infarction due to embolism of precerebral arteries | G63y1 | G63y100 | 195186005 | 300313017 | 32 |
| 1222398015 | Cerebral arterial occlusion | G64 | G64..00 | 20059004 | 1222398015 | 32 |
| 605461000006117 | CVA - cerebral artery occlusion | G64-1 | G64..11 | 230691006 | 345638019 | 32 |
| 218511000000117 | Infarction - cerebral | G64-2 | G64..12 | 432504007 | 2770034014 | 32 |
| 122361000006113 | Stroke due to cerebral arterial occlusion | G64-3 | G64..13 | 230691006 | 345638019 | 32 |
| 118689010 | Cerebral thrombosis | G640 | G640.00 | 71444005 | 118689010 | 32 |
| 300321011 | Cerebral infarction due to thrombosis of cerebral arteries | G6400 | G640000 | 195189003 | 300321011 | 32 |
| 125470015 | Cerebral embolism | G641 | G641.00 | 75543006 | 125470015 | 32 |
| 542831000006116 | Cerebral embolus | G641-1 | G641.11 | 75543006 | 125470015 | 32 |
| 300322016 | Cerebral infarction due to embolism of cerebral arteries | G6410 | G641000 | 195190007 | 300322016 | 32 |
| 395780010 | Cerebral infarction NOS | G64z | G64z.00 | 432504007 | 2770034014 | 32 |
| 345650013 | Brainstem infarction NOS | G64z-1 | G64z.11 | 95457000 | 158113017 | 32 |
| 158118014 | Cerebellar infarction | G64z-2 | G64z.12 | 95460007 | 158118014 | 32 |
| 884501000006113 | Cerebral A. occlusion NOS | G64z-99 | G64z.99 | 682621000000105 | 884501000006113 | 32 |
| 524541000006117 | Brainstem infarction | G64z0 | G64z000 | 95457000 | 158113017 | 32 |
| 57341000006119 | Wallenberg syndrome | G64z1 | G64z100 | 78569004 | 130374019 | 32 |
| 130375018 | Lateral medullary syndrome | G64z1-1 | G64z111 | 78569004 | 130375018 | 32 |
| 451133011 | Left sided cerebral infarction | G64z2 | G64z200 | 307766002 | 451133011 | 32 |
| 451134017 | Right sided cerebral infarction | G64z3 | G64z300 | 307767006 | 451134017 | 32 |
| 2474651019 | Infarction of basal ganglia | G64z4 | G64z400 | 413102000 | 2474651019 | 32 |
| 395788015 | Transient cerebral ischaemia | G65 | G65..00 | 266257000 | 395788015 | 32 |
| 102489017 | Drop attack | G65-1 | G65..11 | 61683000 | 102489017 | 32 |
| 395783012 | Transient ischaemic attack | G65-2 | G65..12 | 266257000 | 395783012 | 32 |
| 67511000006117 | Vertebro-basilar insufficiency | G65-3 | G65..13 | 64009001 | 106394016 | 32 |
| 988951000006117 | Transient Ischaemic Attacks | G65-99 | G65..99 | 266257000 | 988951000006117 | 32 |
| 106392017 | Basilar artery syndrome | G650 | G650.00 | 64009001 | 106392017 | 32 |
| 499739014 | Insufficiency - basilar artery | G650-1 | G650.11 | 64009001 | 499739014 | 32 |
| 25897016 | Subclavian steal syndrome | G652 | G652.00 | 15258001 | 25897016 | 32 |
| 300344014 | Carotid artery syndrome hemispheric | G653 | G653.00 | 195200006 | 300344014 | 32 |
| 300345010 | Multiple and bilateral precerebral artery syndromes | G654 | G654.00 | 195201005 | 300345010 | 32 |
| 106394016 | Vertebrobasilar insufficiency | G656 | G656.00 | 64009001 | 106394016 | 32 |
| 300348012 | Other transient cerebral ischaemia | G65y | G65y.00 | 266257000 | 395788015 | 32 |
| 95931000006111 | Transient cerebral ischaemia NOS | G65z | G65z.00 | 266257000 | 395788015 | 32 |
| 884511000006111 | Transient Ischaemic Attacks | G65z-99 | G65z.99 | 584181000000100 | 884511000006111 | 32 |
| 300352012 | Impending cerebral ischaemia | G65z0 | G65z000 | 195205001 | 300352012 | 32 |
| 300353019 | Intermittent cerebral ischaemia | G65z1 | G65z100 | 195206000 | 300353019 | 32 |
| 300349016 | Transient cerebral ischaemia NOS | G65zz | G65zz00 | 266257000 | 395788015 | 32 |
| 405339016 | Stroke and cerebrovascular accident unspecified | G66 | G66..00 | 230690007 | 345637012 | 32 |
| 605501000006117 | CVA unspecified | G66-1 | G66..11 | 230690007 | 345637012 | 32 |
| 122401000006115 | Stroke unspecified | G66-2 | G66..12 | 230690007 | 345637012 | 32 |
| 605491000006113 | CVA - Cerebrovascular accident unspecified | G66-3 | G66..13 | 230690007 | 345635016 | 32 |
| 884521000006115 | Stroke/CVA - undefined | G66-98 | G66..98 | 685631000000102 | 884521000006115 | 32 |
| 884531000006117 | Stroke | G66-99 | G66..99 | 685631000000102 | 884531000006117 | 32 |
| 300370010 | Left sided CVA | G667 | G667.00 | 195216008 | 300370010 | 32 |
| 300371014 | Right sided CVA | G668 | G668.00 | 195217004 | 300371014 | 32 |
| 542251000006112 | Cereb infarct due cerebral venous thrombosis, nonpyogenic | G6760 | G676000 | 195230003 | 300393016 | 32 |
| 542261000006114 | Cereb infarct due unsp occlus/stenos precerebr arteries | G6W | G6W..00 | 125081000119106 | 3042974014 | 32 |
| 543141000006110 | Cerebrl infarctn due/unspcf occlusn or sten/cerebrl artrs | G6X | G6X..00 | 20059004 | 33759015 | 32 |
| 370701000006118 | [X]Cerebrl infarctn due/unspcf occlusn or sten/cerebrl artrs | Gyu63 | Gyu6300 | 20059004 | 33759015 | 32 |
| 300941014 | [X]Other cerebral infarction | Gyu64 | Gyu6400 | 432504007 | 2770034014 | 32 |
| 300942019 | [X]Occlusion and stenosis of other precerebral arteries | Gyu65 | Gyu6500 | 266253001 | 395777014 | 32 |
| 300943012 | [X]Occlusion and stenosis of other cerebral arteries | Gyu66 | Gyu6600 | 20059004 | 33759015 | 32 |
| 1667741000000110 | [V]Personal history of transient ischaemic attack | ZV12D | ZV12D00 | 751371000000107 | 1667741000000110 | 32 |

Supplementary Table S13: Additional Read Codes for Framingham Risk Score. Framingham included all codes in Table S12, plus these additional codes for heart failure or peripheral arterial disease.

| MedCode Id | Term | Original Read Code | Cleansed Read Code | Snomed CT Concept Id | Snomed CT Description Id | Emis Code Category Id |
| --- | --- | --- | --- | --- | --- | --- |
| 251680018 | H/O: heart failure | 14A6 | 14A6.00 | 161505003 | 251680018 | 32 |
| 453099015 | H/O: Heart failure in last year | 14AM | 14AM.00 | 309634009 | 453099015 | 32 |
| 198599018 | Paroxysmal nocturnal dyspnoea | 1736 | 1736.00 | 55442000 | 198599018 | 27 |
| 1488591011 | Suspected heart failure | 1J60 | 1J60.00 | 394887005 | 1488591011 | 27 |
| 253994013 | O/E - pulmonary oedema | 23E1 | 23E1.00 | 162970000 | 253994013 | 27 |
| 833381000006119 | New York Heart Assoc classification heart failure symptoms | 388D | 388D.00 | 762994006 | 3637504017 | 3 |
| 216184014 | Congestive heart failure monitoring | 662T | 662T.00 | 134378009 | 216184014 | 1 |
| 2616470012 | New York Heart Association Classification - Class I | 662f | 662f.00 | 420300004 | 2616470012 | 1 |
| 2616471011 | New York Heart Association Classification - Class II | 662g | 662g.00 | 421704003 | 2616471011 | 1 |
| 2616472016 | New York Heart Association Classification - Class III | 662h | 662h.00 | 420913000 | 2616472016 | 1 |
| 2616473014 | New York Heart Association Classification - Class IV | 662i | 662i.00 | 422293003 | 2616473014 | 1 |
| 2645623019 | Heart failure education | 679X | 679X.00 | 423475008 | 2645623019 | 17 |
| 308301000000118 | Heart failure care plan discussed with patient | 8CL3 | 8CL3.00 | 872361000000105 | 2253411000000117 | 32 |
| 1484917012 | Heart failure follow-up | 8HBE | 8HBE.00 | 390884006 | 1484917012 | 40 |
| 308041000000118 | Referral to heart failure exercise programme | 8HHz | 8HHz.00 | 704095000 | 3011242019 | 40 |
| 303441000000116 | Discharge from practice nurse heart failure clinic | 8Hg8 | 8Hg8.00 | 200201000000101 | 303441000000116 | 40 |
| 311561000000117 | Referred to heart failure education group | 8Hk0 | 8Hk0.00 | 203791000000106 | 311561000000117 | 40 |
| 2549243014 | Seen in heart failure clinic | 9N0k | 9N0k.00 | 416717003 | 2549243014 | 37 |
| 2548316014 | Seen by community heart failure nurse | 9N2p | 9N2p.00 | 417359009 | 2548316014 | 37 |
| 303861000000118 | Did not attend practice nurse heart failure clinic | 9N4s | 9N4s.00 | 200361000000106 | 303861000000118 | 37 |
| 407181000000116 | Did not attend heart failure clinic | 9N4w | 9N4w.00 | 248571000000104 | 407181000000116 | 37 |
| 303361000000111 | Referred by heart failure nurse specialist | 9N6T | 9N6T.00 | 200171000000102 | 303361000000111 | 37 |
| 226181000000110 | Left ventricular dysfunction monitoring administration | 9On | 9On..00 | 713781000000103 | 1565021000000117 | 37 |
| 2533628012 | Left ventricular dysfunction monitoring first letter | 9On0 | 9On0.00 | 414586001 | 2533628012 | 37 |
| 2533629016 | Left ventricular dysfunction monitoring second letter | 9On1 | 9On1.00 | 414588000 | 2533629016 | 37 |
| 2533630014 | Left ventricular dysfunction monitoring third letter | 9On2 | 9On2.00 | 414589008 | 2533630014 | 37 |
| 2549089012 | Left ventricular dysfunction monitoring verbal invite | 9On3 | 9On3.00 | 416573008 | 2549089012 | 37 |
| 407001000000113 | Left ventricular dysfunction monitoring telephone invite | 9On4 | 9On4.00 | 716411000000109 | 1568961000000117 | 37 |
| 308231000000118 | Heart failure monitoring administration | 9Or | 9Or..00 | 713791000000101 | 1565031000000115 | 37 |
| 406801000000118 | Heart failure monitoring telephone invite | 9Or1 | 9Or1.00 | 717531000000102 | 1570071000000118 | 37 |
| 407041000000111 | Heart failure monitoring verbal invite | 9Or2 | 9Or2.00 | 717501000000108 | 1570041000000112 | 37 |
| 407061000000112 | Heart failure monitoring first letter | 9Or3 | 9Or3.00 | 716971000000109 | 1569521000000114 | 37 |
| 407081000000115 | Heart failure monitoring second letter | 9Or4 | 9Or4.00 | 717191000000108 | 1569731000000119 | 37 |
| 407101000000114 | Heart failure monitoring third letter | 9Or5 | 9Or5.00 | 716621000000101 | 1569171000000116 | 37 |
| 138971000000111 | Exception reporting: LVD quality indicators | 9h1 | 9h1..00 | 716551000000107 | 1569101000000112 | 37 |
| 139061000000114 | Excepted from LVD quality indicators: Patient unsuitable | 9h11 | 9h11.00 | 717181000000106 | 1569721000000116 | 37 |
| 139071000000119 | Excepted from LVD quality indicators: Informed dissent | 9h12 | 9h12.00 | 717281000000100 | 1569821000000111 | 37 |
| 407441000000115 | Exception reporting: heart failure quality indicators | 9hH | 9hH..00 | 715951000000107 | 1568511000000111 | 37 |
| 1561941000006119 | Excepted heart failure quality indicators: Patient unsuitabl | 9hH0 | 9hH0.00 | 717481000000104 | 1570021000000117 | 37 |
| 1561951000006117 | Excepted heart failure quality indicators: Informed dissent | 9hH1 | 9hH1.00 | 717491000000102 | 1570031000000115 | 37 |
| 1490256017 | Pulmonary oedema - acute | G581-2 | G581.12 | 40541001 | 492666016 | 32 |
| 139481017 | Weak heart | G58z-1 | G58z.11 | 84114007 | 139475013 | 32 |
| 301689014 | Pulmonary congestion and hypostasis | H54 | H54..00 | 196115007 | 301689014 | 32 |
| 112265015 | Pulmonary congestion | H541 | H541.00 | 67599009 | 112265015 | 32 |
| 494669012 | Chronic pulmonary oedema | H5410 | H541000 | 46847001 | 494669012 | 32 |
| 301694014 | Pulmonary oedema NOS | H541z | H541z00 | 19242006 | 479262018 | 32 |
| 301695010 | Pulmonary congestion and hypostasis NOS | H54z | H54z.00 | 196115007 | 301689014 | 32 |
| 301741013 | Acute pulmonary oedema unspecified | H584 | H584.00 | 40541001 | 492666016 | 32 |
| 301743011 | Acute pulmonary oedema NOS | H584z | H584z00 | 40541001 | 492666016 | 32 |
| 833381000006119 | New York Heart Assoc classification heart failure symptoms | 388D | 388D.00 | 762994006 | 3637504017 | 3 |
| 2675255018 | Congestive heart failure due to valvular disease | G5804 | G580400 | 426611007 | 2675255018 | 32 |
| 90135019 | Malignant hypertensive heart disease | G210 | G210.00 | 54225002 | 90135019 | 32 |
| 728681000006116 | Malignant hypertensive heart disease without CCF | G2100 | G210000 | 36315003 | 60617018 | 32 |
| 728671000006119 | Malignant hypertensive heart disease with CCF | G2101 | G210100 | 83105008 | 1236017010 | 32 |
| 504901000006118 | Benign hypertensive heart disease with CCF | G2111 | G211100 | 194767001 | 299653017 | 32 |
| 741681000006111 | Hypertensive heart disease NOS with CCF | G21z1 | G21z100 | 64715009 | 107545013 | 32 |
| 110659019 | Malignant hypertensive heart AND renal disease | G230 | G230.00 | 66610008 | 110659019 | 32 |
| 741701000006114 | Hypertensive heart&renal dis wth (congestive) heart failure | G232 | G232.00 | 194779001 | 299672017 | 32 |
| 789941000006117 | Hyperten heart&renal dis+both(congestv)heart and renal fail | G234 | G234.00 | 194781004 | 299674016 | 32 |
| 72934016 | Rheumatic left ventricular failure | G1yz1 | G1yz100 | 43736008 | 72934016 | 32 |
| 1488804017 | Heart failure confirmed | 1O1 | 1O1..00 | 395105005 | 1488804017 | 27 |
| 1484918019 | Heart failure annual review | 662W | 662W.00 | 390885007 | 1484918019 | 1 |
| 404741000000119 | Heart failure 6 month review | 662p | 662p.00 | 247361000000100 | 404741000000119 | 1 |
| 451426015 | Cardiac failure therapy | 8B29 | 8B29.00 | 308118002 | 451426015 | 32 |
| 2549208013 | Admit heart failure emergency | 8H2S | 8H2S.00 | 416683003 | 3082850014 | 40 |
| 308261000000111 | Heart failure review completed | 9Or0 | 9Or0.00 | 202231000000106 | 308261000000111 | 37 |
| 82584011 | Acute cor pulmonale | G400 | G400.00 | 49584005 | 82584011 | 32 |
| 132655012 | Chronic cor pulmonale | G41z-1 | G41z.11 | 79955004 | 132655012 | 32 |
| 1778488011 | Congestive cardiomyopathy | G5540 | G554000 | 399020009 | 1778488011 | 32 |
| 350413012 | Congestive obstructive cardiomyopathy | G5540-1 | G554011 | 233871002 | 350413012 | 32 |
| 139475013 | Heart failure | G58 | G58..00 | 84114007 | 139475013 | 32 |
| 139482012 | Cardiac failure | G58-1 | G58..11 | 84114007 | 139482012 | 32 |
| 70653017 | Congestive heart failure | G580 | G580.00 | 42343007 | 70653017 | 32 |
| 493287011 | Congestive cardiac failure | G580-1 | G580.11 | 42343007 | 70653017 | 32 |
| 206703015 | Right heart failure | G580-2 | G580.12 | 128404006 | 206703015 | 32 |
| 490972013 | Right ventricular failure | G580-3 | G580.13 | 367363000 | 490972013 | 32 |
| 510016018 | Biventricular failure | G580-4 | G580.14 | 92506005 | 153058012 | 32 |
| 18472010 | Acute congestive heart failure | G5800 | G580000 | 10633002 | 18472010 | 32 |
| 147247018 | Chronic congestive heart failure | G5801 | G580100 | 88805009 | 147247018 | 32 |
| 300179017 | Decompensated cardiac failure | G5802 | G580200 | 195111005 | 300179017 | 32 |
| 300180019 | Compensated cardiac failure | G5803 | G580300 | 195112003 | 300180019 | 32 |
| 141306010 | Left ventricular failure | G581 | G581.00 | 85232009 | 141306010 | 32 |
| 1495417010 | Asthma - cardiac | G581-1 | G581.11 | 71892000 | 119456014 | 32 |
| 411506018 | Impaired left ventricular function | G581-3 | G581.13 | 275514001 | 411506018 | 32 |
| 300190010 | Acute left ventricular failure | G5810 | G581000 | 195114002 | 300190010 | 32 |
| 94251011 | Acute heart failure | G582 | G582.00 | 56675007 | 94251011 | 32 |
| 395772015 | Heart failure NOS | G58z | G58z.00 | 84114007 | 139475013 | 32 |
| 223981000000118 | Cardiac failure NOS | G58z-2 | G58z.12 | 84114007 | 139482012 | 32 |
| 216207010 | Left ventricular systolic dysfunction | G5yy9 | G5yy900 | 134401001 | 216207010 | 32 |
| 1489358014 | Left ventricular diastolic dysfunction | G5yyA | G5yyA00 | 395704004 | 1489358014 | 32 |
| 317955011 | [D]Cardiorespiratory failure | R2y10 | R2y1000 | 410431009 | 2472092014 | 32 |
| 316833010 | Congenital cardiac failure | Q48y1 | Q48y100 | 206586007 | 316833010 | 32 |
| 450665015 | Ischaemic toe | 2G63 | 2G63.00 | 307408003 | 450665015 | 27 |
| 286570019 | Gas gangrene-foot | A3A0F | A3A0F00 | 186423003 | 286570019 | 32 |
| 223991000000116 | Aorto-iliac disease | G700-1 | G700.11 | 233956002 | 350529018 | 32 |
| 300505017 | Gangrene of toe | G7320 | G732000 | 195302000 | 300505017 | 32 |
| 41903014 | Acrocyanosis | G73y2 | G73y200 | 25003006 | 41903014 | 32 |
| 454901000006118 | Acroparaesthesia - Schultze's type | G73y4 | G73y400 | 57105000 | 1216757012 | 32 |
| 454891000006117 | Acroparaesthesia - Nothnagel's type | G73y5 | G73y500 | 77788005 | 503681010 | 32 |
| 503681010 | Nothnagel's vasomotor acroparaesthesia | G73y5-1 | G73y511 | 77788005 | 503681010 | 32 |
| 300512014 | Acroparaesthesia - unspecified | G73y6 | G73y600 | 79256006 | 131501015 | 32 |
| 124092018 | Erythrocyanosis | G73y7 | G73y700 | 74725000 | 124092018 | 32 |
| 61977017 | Erythromelalgia | G73y8 | G73y800 | 37151006 | 61977017 | 32 |
| 350564010 | Aortoiliac obstruction | G740-2 | G740.12 | 233972005 | 350564010 | 32 |
| 357894012 | Arterial leg ulcer | M2713 | M271300 | 238793001 | 357894012 | 32 |
| 357893018 | Mixed venous and arterial leg ulcer | M2714 | M271400 | 238792006 | 357893018 | 32 |
| 317347010 | [D]Gangrene of toe in diabetic | R0542 | R054200 | 195302000 | 300505017 | 32 |
| 317351012 | [D]Failure of peripheral circulation | R0550 | R055000 | 27942005 | 46780018 | 32 |
| 1222508013 | [D]Peripheral circulatory failure | R0550-1 | R055011 | 27942005 | 46780018 | 32 |
| 300539018 | Embolism and thrombosis of the femoral artery | G7424 | G742400 | 195323006 | 300539018 | 32 |
| 300540016 | Embolism and thrombosis of the popliteal artery | G7425 | G742500 | 195324000 | 300540016 | 32 |
| 300541017 | Embolism and thrombosis of the anterior tibial artery | G7426 | G742600 | 195325004 | 300541017 | 32 |
| 300542012 | Embolism and thrombosis of the dorsalis pedis artery | G7427 | G742700 | 195326003 | 300542012 | 32 |
| 300547018 | Embolism and thrombosis of a leg artery NOS | G7429 | G742900 | 195318006 | 300534011 | 32 |
| 300553018 | Embolism and/or thrombosis of the common iliac artery | G74y0 | G74y000 | 195335005 | 300553018 | 32 |
| 300554012 | Embolism and/or thrombosis of the internal iliac artery | G74y1 | G74y100 | 734298005 | 3503835018 | 32 |
| 300555013 | Embolism and/or thrombosis of the external iliac artery | G74y2 | G74y200 | 734299002 | 3503839012 | 32 |
| 300556014 | Embolism and thrombosis of the iliac artery unspecified | G74y3 | G74y300 | 266262004 | 395794011 | 32 |
| 639341000006115 | Emerg aortic bypass by anastomosis axillary to femoral art | 7A100 | 7A10000 | 175276005 | 271413015 | 32 |
| 501011000006118 | Axillo-bifemoral bypass graft | 7A102 | 7A10200 | 388997005 | 1493587018 | 32 |
| 271416011 | Axillo-unifemoral PTFE bypass graft | 7A103 | 7A10300 | 175279003 | 271416011 | 32 |
| 271431019 | Other bypass of bifurcation of aorta | 7A12 | 7A12.00 | 233373009 | 349683014 | 32 |
| 224121000000118 | Aorto bifemoral graft | 7A121-1 | 7A12111 | 233373009 | 349688017 | 32 |
| 410961010 | Dacron aortofemoral Y graft | 7A121-2 | 7A12112 | 275038008 | 410961010 | 32 |
| 488631000006116 | Aorto biiliac graft | 7A123-1 | 7A12311 | 265495003 | 394305010 | 32 |
| 410962015 | Dacron aortoiliac Y graft | 7A123-2 | 7A12312 | 275039000 | 410962015 | 32 |
| 271444012 | Other specified other bypass of bifurcation of aorta | 7A12y | 7A12y00 | 233373009 | 349683014 | 32 |
| 271445013 | Other bypass of bifurcation of aorta NOS | 7A12z | 7A12z00 | 233373009 | 349683014 | 32 |
| 271509013 | Open embolectomy of bifurcation of aorta | 7A192 | 7A19200 | 175348000 | 271509013 | 32 |
| 639471000006115 | Emerg bypass iliac art by iliac/femoral art anastomosis NEC | 7A410 | 7A41000 | 51018000 | 85012019 | 32 |
| 640081000006119 | Emergency femoro-femoral prosthetic cross over graft | 7A412-1 | 7A41211 | 275040003 | 410963013 | 32 |
| 668731000006114 | Femoro-femoral prosthetic cross over graft | 7A413-1 | 7A41311 | 175685004 | 272001019 | 32 |
| 639391000006112 | Emerg bypass comm iliac art by aorta/com iliac art anast NEC | 7A414 | 7A41400 | 116360008 | 183983017 | 32 |
| 639521000006117 | Emerg bypass leg artery by aorta/com fem art anastomosis NEC | 7A416 | 7A41600 | 116360008 | 183983017 | 32 |
| 530221000006117 | Bypass leg artery by aorta/com femoral art anastomosis NEC | 7A41B | 7A41B00 | 116360008 | 183983017 | 32 |
| 530231000006119 | Bypass leg artery by aorta/deep femoral art anastomosis NEC | 7A41C | 7A41C00 | 405482000 | 2157285016 | 32 |
| 785611000006113 | Ilio-femoral prosthetic cross over graft | 7A41F | 7A41F00 | 175594003 | 271866014 | 32 |
| 394326010 | Reconstruction of iliac artery | 7A42 | 7A42.00 | 265516003 | 394326010 | 32 |
| 349652010 | Reconstruction of common iliac artery | 7A42-1 | 7A42.11 | 233345007 | 349652010 | 32 |
| 641691000006118 | Endarterectomy and patch repair of iliac artery | 7A420 | 7A42000 | 265517007 | 394327018 | 32 |
| 410964019 | Endarterectomy and patch repair of common iliac artery | 7A420-1 | 7A42011 | 275041004 | 410964019 | 32 |
| 394328011 | Iliac endarterectomy and patch | 7A420-2 | 7A42012 | 265517007 | 394328011 | 32 |
| 394329015 | Endarterectomy of iliac artery NEC | 7A421 | 7A42100 | 307800001 | 451173017 | 32 |
| 410965018 | Endarterectomy of common iliac artery NEC | 7A421-1 | 7A42111 | 233304004 | 349571019 | 32 |
| 271880019 | Other specified reconstruction of iliac artery | 7A42y | 7A42y00 | 265516003 | 394326010 | 32 |
| 271881015 | Reconstruction of iliac artery NOS | 7A42z | 7A42z00 | 265516003 | 394326010 | 32 |
| 394330013 | Other open operations on iliac artery | 7A43 | 7A43.00 | 265511008 | 394321017 | 32 |
| 410966017 | Other open operations on common iliac artery | 7A43-1 | 7A43.11 | 265511008 | 394321017 | 32 |
| 394331012 | Repair of iliac artery NEC | 7A430 | 7A43000 | 450703003 | 2916807015 | 32 |
| 410967014 | Repair of common iliac artery NEC | 7A430-1 | 7A43011 | 450704009 | 2916913013 | 32 |
| 394332017 | Open embolectomy of iliac artery | 7A431 | 7A43100 | 265521000 | 394332017 | 32 |
| 411214011 | Open embolectomy of common iliac artery | 7A431-1 | 7A43111 | 275254000 | 411214011 | 32 |
| 455900010 | Open insertion of iliac artery stent | 7A433 | 7A43300 | 312226000 | 455900010 | 32 |
| 271901019 | Percutaneous transluminal embolectomy of iliac artery | 7A441 | 7A44100 | 175613000 | 271901019 | 32 |
| 271905011 | Insertion of iliac artery stent | 7A443 | 7A44300 | 175615007 | 271905011 | 32 |
| 455901014 | Percutaneous transluminal insertion of iliac artery stent | 7A444 | 7A44400 | 312227009 | 455901014 | 32 |
| 271907015 | Other specified transluminal operation on iliac artery | 7A44y | 7A44y00 | 265522007 | 394333010 | 32 |
| 271908013 | Transluminal operation on iliac artery NOS | 7A44z | 7A44z00 | 265522007 | 394333010 | 32 |
| 32181000006113 | Other emerg bypass femoral or popliteal art by anastomosis | 7A47-1 | 7A47.11 | 116360008 | 183983017 | 32 |
| 349669012 | Other emergency bypass of popliteal artery | 7A47-4 | 7A47.14 | 116360008 | 183983017 | 32 |
| 639431000006118 | Emerg bypass femoral art by fem/pop art anast c prosth NEC | 7A470 | 7A47000 | 112828007 | 184069011 | 32 |
| 639581000006118 | Emerg bypass popliteal art by pop/pop art anast c prosth NEC | 7A471 | 7A47100 | 233384002 | 349710011 | 32 |
| 639421000006116 | Emerg bypass femoral art by fem/pop a anast c vein graft NEC | 7A472 | 7A47200 | 112828007 | 184069011 | 32 |
| 639551000006114 | Emerg bypass pop art by pop/pop art anast c vein graft NEC | 7A473 | 7A47300 | 233384002 | 349710011 | 32 |
| 639451000006113 | Emerg bypass femoral art by fem/tib art anast c prosth NEC | 7A474 | 7A47400 | 233383008 | 349706013 | 32 |
| 639441000006111 | Emerg bypass femoral art by fem/tib a anast c vein graft NEC | 7A476 | 7A47600 | 233383008 | 349706013 | 32 |
| 639561000006111 | Emerg bypass pop art by pop/tib art anast c vein graft NEC | 7A477 | 7A47700 | 233384002 | 349710011 | 32 |
| 639541000006112 | Emerg bypass pop art by pop/peron art anast c vein graft NEC | 7A47B | 7A47B00 | 233384002 | 349710011 | 32 |
| 639601000006111 | Emerg bypass popliteal artery by pop/fem art anastomosis NEC | 7A47D | 7A47D00 | 116360008 | 183983017 | 32 |
| 31361000006114 | Other emergency bypass of femoral or popliteal artery OS | 7A47y | 7A47y00 | 405516005 | 2157318011 | 32 |
| 31351000006112 | Other emergency bypass of femoral or popliteal artery NOS | 7A47z | 7A47z00 | 116360008 | 183983017 | 32 |
| 349666017 | Other bypass of femoral or popliteal artery by anastomosis | 7A48-1 | 7A48.11 | 405516005 | 2157318011 | 32 |
| 349662015 | Other bypass of popliteal artery | 7A48-5 | 7A48.15 | 233384002 | 349710011 | 32 |
| 530811000006112 | Bypass popliteal artery by pop/pop a anast c prosthesis NEC | 7A481 | 7A48100 | 233384002 | 349710011 | 32 |
| 530821000006116 | Bypass popliteal artery by pop/pop a anast c vein graft NEC | 7A483 | 7A48300 | 233384002 | 349710011 | 32 |
| 530831000006118 | Bypass popliteal artery by pop/tib a anast c prosthesis NEC | 7A485 | 7A48500 | 233384002 | 349710011 | 32 |
| 530841000006111 | Bypass popliteal artery by pop/tib a anast c vein graft NEC | 7A487 | 7A48700 | 233384002 | 349710011 | 32 |
| 530781000006110 | Bypass popliteal art by pop/peron art anast c vein graft NEC | 7A48B | 7A48B00 | 233384002 | 349710011 | 32 |
| 530791000006113 | Bypass popliteal artery by pop/fem artery anastomosis NEC | 7A48D | 7A48D00 | 112828007 | 184069011 | 32 |
| 668741000006116 | Femoro-femoral prosthetic cross over graft | 7A48E | 7A48E00 | 175685004 | 272001019 | 32 |
| 394340011 | Reconstruction of femoral artery or popliteal artery | 7A49 | 7A49.00 | 265528006 | 394340011 | 32 |
| 410976019 | Reconstruction of common femoral artery | 7A49-1 | 7A49.11 | 275053000 | 410976019 | 32 |
| 410977011 | Reconstruction of femoral artery | 7A49-3 | 7A49.13 | 275054006 | 410977011 | 32 |
| 410974016 | Reconstruction of popliteal artery | 7A49-4 | 7A49.14 | 275051003 | 410974016 | 32 |
| 411078012 | Reconstruction of superficial femoral artery | 7A49-5 | 7A49.15 | 275145005 | 411078012 | 32 |
| 641681000006116 | Endarterectomy and patch repair of femoral artery | 7A490 | 7A49000 | 175689005 | 272011014 | 32 |
| 641701000006118 | Endarterectomy and patch repair of popliteal artery | 7A491 | 7A49100 | 175690001 | 272012019 | 32 |
| 272013012 | Endarterectomy of femoral artery NEC | 7A492 | 7A49200 | 16589005 | 28064017 | 32 |
| 272014018 | Endarterectomy of popliteal artery NEC | 7A493 | 7A49300 | 233310004 | 349583017 | 32 |
| 201771000006117 | Profundoplasty femoral artery & patch repair deep fem artery | 7A494 | 7A49400 | 175693004 | 272015017 | 32 |
| 201761000006112 | Profundoplasty and patch repair of popliteal artery | 7A495 | 7A49500 | 175694005 | 272016016 | 32 |
| 272017013 | Profundoplasty of femoral artery NEC | 7A496 | 7A49600 | 405376002 | 2157187018 | 32 |
| 272018015 | Profundoplasty of popliteal artery NEC | 7A497 | 7A49700 | 405376002 | 2157187018 | 32 |
| 272019011 | Reconstruction of femoral artery with vein graft | 7A498 | 7A49800 | 175697003 | 272019011 | 32 |
| 272020017 | Reconstruction of popliteal artery with vein graft | 7A499 | 7A49900 | 175698008 | 272020017 | 32 |
| 187331000006112 | Reconstruction of femoral or popliteal artery OS | 7A49y | 7A49y00 | 265528006 | 394340011 | 32 |
| 272022013 | Reconstruction of femoral or popliteal artery NOS | 7A49z | 7A49z00 | 265528006 | 394340011 | 32 |
| 394341010 | Other open operations on femoral artery or popliteal artery | 7A4A | 7A4A.00 | 118805000 | 183489012 | 32 |
| 410981011 | Other open operations on common femoral artery | 7A4A-1 | 7A4A.11 | 118805000 | 183489012 | 32 |
| 410979014 | Other open operations on popliteal artery | 7A4A-3 | 7A4A.13 | 118805000 | 183489012 | 32 |
| 410978018 | Other open operations on superficial femoral artery | 7A4A-4 | 7A4A.14 | 118805000 | 183489012 | 32 |
| 272029016 | Repair of femoral artery NEC | 7A4A0 | 7A4A000 | 265528006 | 394340011 | 32 |
| 272030014 | Repair of popliteal artery NEC | 7A4A1 | 7A4A100 | 405375003 | 2157186010 | 32 |
| 272031013 | Open embolectomy of femoral artery | 7A4A2 | 7A4A200 | 175704008 | 272031013 | 32 |
| 272032018 | Open thrombectomy of femoral artery | 7A4A2-1 | 7A4A211 | 175704008 | 272032018 | 32 |
| 272033011 | Open femoral embolectomy | 7A4A2-2 | 7A4A212 | 175704008 | 272033011 | 32 |
| 272035016 | Open embolectomy popliteal artery | 7A4A3 | 7A4A300 | 175705009 | 272035016 | 32 |
| 272034017 | Open thrombectomy of popliteal artery | 7A4A3-1 | 7A4A311 | 175705009 | 272034017 | 32 |
| 272041011 | Repair of femoral artery with temporary silastic shunt | 7A4A7 | 7A4A700 | 175710008 | 272041011 | 32 |
| 272042016 | Repair of popliteal artery with temporary silastic shunt | 7A4A8 | 7A4A800 | 175711007 | 272042016 | 32 |
| 24571000006113 | Other open operation on femoral or popliteal artery OS | 7A4Ay | 7A4Ay00 | 118805000 | 183489012 | 32 |
| 272055015 | Percutaneous transluminal embolectomy of femoral artery | 7A4B2 | 7A4B200 | 175717006 | 272055015 | 32 |
| 272056019 | Percutaneous transluminal embolectomy of popliteal artery | 7A4B3 | 7A4B300 | 175718001 | 272056019 | 32 |
| 272059014 | Percutaneous transluminal embolisation of femoral artery | 7A4B4 | 7A4B400 | 175719009 | 272059014 | 32 |
| 272061017 | Percutaneous transluminal embolisation of popliteal artery | 7A4B5 | 7A4B500 | 175720003 | 272061017 | 32 |
| 238621000006112 | Percut translum thrombolysis femoral graft streptokinase | 7A4B8 | 7A4B800 | 309449009 | 452880015 | 32 |
| 1548341000006118 | Percutaneous transluminal insertion of stent femoral artery | 7A4B9 | 7A4B900 | 426093007 | 2675938013 | 32 |
| 272074011 | Revision of reconstruction of artery | 7A50 | 7A50.00 | 175729002 | 272074011 | 32 |
| 272075012 | Revision of reconstruction involving aorta | 7A500 | 7A50000 | 175730007 | 272075012 | 32 |
| 272076013 | Revision of reconstruction involving iliac artery | 7A501 | 7A50100 | 175731006 | 272076013 | 32 |
| 272077016 | Revision of reconstruction involving femoral artery | 7A502 | 7A50200 | 175732004 | 272077016 | 32 |
| 272078014 | Revision of reconstruction of popliteal artery | 7A503 | 7A50300 | 175733009 | 272078014 | 32 |
| 262265016 | Femoral arteriogram abnormal | 5593 | 5593.00 | 168953002 | 262265016 | 41 |
| 262274019 | Lower limb arteriogram abnorm. | 55A2 | 55A2.00 | 168957001 | 2668096010 | 41 |
| 451376017 | H/O: Peripheral vascular disease procedure | 14NB | 14NB.00 | 308071004 | 451376017 | 32 |
| 216185010 | Peripheral vascular disease monitoring | 662U | 662U.00 | 134379001 | 216185010 | 1 |
| 530051000006117 | Bypass aorta by anastomosis axillary to femoral artery NEC | 7A101 | 7A10100 | 233379008 | 349697018 | 32 |
| 173691000006116 | Replacement of aneurysmal bifurcation of aorta | 7A11 | 7A11.00 | 175282008 | 271419016 | 32 |
| 639641000006113 | Emerg repl aneurysm bifurc aorta by anast aorta to fem art | 7A110 | 7A11000 | 175283003 | 271421014 | 32 |
| 173461000006110 | Replace aneurysm bifurc aorta by anast aorta to femoral art | 7A111 | 7A11100 | 175284009 | 271422019 | 32 |
| 639651000006110 | Emerg repl aneurysm bifurc aorta by anast aorta to iliac a | 7A112 | 7A11200 | 265492000 | 394302013 | 32 |
| 349689013 | Y graft of abdominal Aortic aneurysm (emergency) | 7A112-1 | 7A11211 | 233374003 | 2669093011 | 32 |
| 173471000006115 | Replace aneurysm bifurc aorta by anast aorta to iliac artery | 7A113 | 7A11300 | 265493005 | 394303015 | 32 |
| 410960011 | Y graft abdominal Aortic aneurysm | 7A113-1 | 7A11311 | 275037003 | 410960011 | 32 |
| 173711000006118 | Replacement of aneurysmal bifurcation of aorta OS | 7A11y | 7A11y00 | 175282008 | 271419016 | 32 |
| 271430018 | Replacement of aneurysmal bifurcation of aorta NOS | 7A11z | 7A11z00 | 175282008 | 271419016 | 32 |
| 639371000006111 | Emerg bypass bifurc aorta by anast aorta to femoral artery | 7A120 | 7A12000 | 175290008 | 271432014 | 32 |
| 530061000006115 | Bypass bifurc aorta by anastom aorta to femoral artery NEC | 7A121 | 7A12100 | 448171003 | 2900875010 | 32 |
| 639381000006114 | Emerg bypass bifurc aorta by anastom aorta to iliac artery | 7A122 | 7A12200 | 175292000 | 271438013 | 32 |
| 530071000006110 | Bypass bifurcation aorta by anastom aorta to iliac artery | 7A123 | 7A12300 | 265495003 | 394305010 | 32 |
| 639901000006118 | Emerg replace aneurysm thor aorta by anastom aorta to aorta | 7A131 | 7A13100 | 175299009 | 271449019 | 32 |
| 639891000006117 | Emerg replace aneurysm suprarenal aorta by anast aorta/aorta | 7A132 | 7A13200 | 175300001 | 271450019 | 32 |
| 639811000006110 | Emerg replace aneurysm infrarenal aorta by anast aorta/aorta | 7A133 | 7A13300 | 175301002 | 271451015 | 32 |
| 639721000006114 | Emerg replace aneurysm abdom aorta by anast aorta/aorta NEC | 7A134 | 7A13400 | 123679006 | 192168019 | 32 |
| 349694013 | Tube graft abdominal Aortic aneurysm (emergency) | 7A134-1 | 7A13411 | 233377005 | 2669005014 | 32 |
| 173651000006110 | Replace aneurysm thoracic aorta by anast of aorta/aorta NEC | 7A141 | 7A14100 | 116360008 | 183983017 | 32 |
| 173441000006111 | Replace aneurysm abdominal aorta by anast aorta to aorta NEC | 7A144 | 7A14400 | 448122008 | 2899751014 | 32 |
| 349693019 | Tube graft of Abdominal aortic aneurysm | 7A144-1 | 7A14411 | 233376001 | 349693019 | 32 |
| 1548161000006117 | Endovascular stenting infrarenal abdominal aortic aneurysm | 7A1B0 | 7A1B000 | 441612006 | 2816827015 | 32 |
| 357881000000111 | Endovascular stenting of thoracic aortic aneurysm | 7A1B2 | 7A1B200 | 378491000000109 | 745451000000110 | 32 |
| 271546012 | Percutaneous transluminal angioplasty of carotid artery | 7A220 | 7A22000 | 175380003 | 271546012 | 32 |
| 271640014 | Operation on aneurysm of subclavian artery | 7A27C | 7A27C00 | 175447009 | 271640014 | 32 |
| 271641013 | Operation on aneurysm of axillary artery | 7A27D | 7A27D00 | 175448004 | 271641013 | 32 |
| 271642018 | Operation on aneurysm of brachial artery | 7A27E | 7A27E00 | 175449007 | 271642018 | 32 |
| 239511000006111 | Percutaneous transluminal angioplasty of subclavian artery | 7A280 | 7A28000 | 175454003 | 271652019 | 32 |
| 239441000006114 | Percutaneous transluminal angioplasty of brachial artery | 7A281 | 7A28100 | 175455002 | 271653012 | 32 |
| 1235117015 | Percutaneous transluminal angioplasty of vertebral artery | 7A282 | 7A28200 | 86274005 | 1235117015 | 32 |
| 271670016 | Percutaneous transluminal angioplasty of axillary artery | 7A28C | 7A28C00 | 175464007 | 271670016 | 32 |
| 271699014 | Operation on aneurysm of renal artery | 7A313 | 7A31300 | 175485007 | 271699014 | 32 |
| 94463015 | Percutaneous transluminal angioplasty of renal artery | 7A320 | 7A32000 | 56801004 | 497697011 | 32 |
| 271775016 | Operation on aneurysm of coeliac artery NEC | 7A34C | 7A34C00 | 392242000 | 1486224017 | 32 |
| 271777012 | Operation on aneurysm of superior mesenteric artery NEC | 7A34D | 7A34D00 | 392242000 | 1486224017 | 32 |
| 271778019 | Operation on aneurysm of inferior mesenteric artery NEC | 7A34E | 7A34E00 | 392242000 | 1486224017 | 32 |
| 271779010 | Operation on aneurysm of suprarenal artery NEC | 7A34F | 7A34F00 | 392242000 | 1486224017 | 32 |
| 46601000006110 | Operation on aneurysm visceral branch of abdominal aorta NEC | 7A34K | 7A34K00 | 392242000 | 1486224017 | 32 |
| 271795010 | Percutaneous transluminal angioplasty of coeliac artery NEC | 7A350 | 7A35000 | 233270005 | 349498018 | 32 |
| 232781000006115 | Percutaneous transluminal angioplasty suprarenal artery NEC | 7A353 | 7A35300 | 233258006 | 349468013 | 32 |
| 394322012 | Replacement of aneurysmal iliac artery | 7A40 | 7A40.00 | 265512001 | 394322012 | 32 |
| 639791000006111 | Emerg replace aneurysm iliac art by iliac/femoral art anast | 7A400 | 7A40000 | 175562008 | 271828011 | 32 |
| 173661000006112 | Replace aneurysmal iliac art by iliac/femoral art anast NEC | 7A401 | 7A40100 | 265512001 | 394322012 | 32 |
| 349717014 | Replacement of aneurysmal iliac artery by anastomosis | 7A40-1 | 7A40.11 | 233388004 | 349717014 | 32 |
| 639911000006115 | Emerg replace aneurysmal iliac artery by fem/fem art anast | 7A402 | 7A40200 | 175564009 | 271830013 | 32 |
| 173671000006117 | Replace aneurysmal iliac artery by fem/fem artery anast NEC | 7A403 | 7A40300 | 265512001 | 394322012 | 32 |
| 639821000006119 | Emerg replace aneurysm leg artery by aorta/com fem art anast | 7A406 | 7A40600 | 175568007 | 271834016 | 32 |
| 639831000006116 | Emerg replace aneurysm leg artery by aorta/sup fem art anast | 7A407 | 7A40700 | 175569004 | 271835015 | 32 |
| 639801000006112 | Emerg replace aneurysm iliac artery by iliac/iliac art anast | 7A408 | 7A40800 | 175570003 | 271836019 | 32 |
| 173481000006117 | Replace aneurysm com iliac a by aorta/com iliac a anast NEC | 7A409 | 7A40900 | 116360008 | 183983017 | 32 |
| 173551000006114 | Replace aneurysm iliac art by aorta/ext iliac art anast NEC | 7A40A | 7A40A00 | 116360008 | 183983017 | 32 |
| 173571000006116 | Replace aneurysm leg artery by aorta/com fem art anast NEC | 7A40B | 7A40B00 | 116360008 | 183983017 | 32 |
| 173581000006118 | Replace aneurysm leg artery by aorta/sup fem art anast NEC | 7A40C | 7A40C00 | 116360008 | 183983017 | 32 |
| 173561000006111 | Replace aneurysm iliac artery by iliac/iliac art anast NEC | 7A40D | 7A40D00 | 116360008 | 183983017 | 32 |
| 271842015 | Other specified replacement of aneurysmal iliac artery | 7A40y | 7A40y00 | 116360008 | 183983017 | 32 |
| 271843013 | Replacement of aneurysmal iliac artery NOS | 7A40z | 7A40z00 | 116360008 | 183983017 | 32 |
| 394323019 | Other bypass of iliac artery | 7A41 | 7A41.00 | 405477007 | 2157280014 | 32 |
| 530201000006110 | Bypass iliac artery by iliac/femoral artery anastomosis NEC | 7A411 | 7A41100 | 51018000 | 1780391000000113 | 32 |
| 349677011 | Other bypass of iliac artery by anastomosis | 7A41-1 | 7A41.11 | 405477007 | 2157280014 | 32 |
| 639491000006119 | Emerg bypass iliac artery by femoral/femoral art anast NEC | 7A412 | 7A41200 | 233380006 | 349698011 | 32 |
| 530191000006112 | Bypass iliac artery by femoral/femoral art anastomosis NEC | 7A413 | 7A41300 | 233380006 | 349698011 | 32 |
| 639481000006117 | Emerg bypass iliac artery by aorta/ext iliac art anast NEC | 7A415 | 7A41500 | 116360008 | 183983017 | 32 |
| 639501000006110 | Emerg bypass iliac artery by iliac/iliac art anastomosis NEC | 7A418 | 7A41800 | 447920002 | 2900285015 | 32 |
| 530091000006111 | Bypass common iliac artery by aorta/com iliac art anast NEC | 7A419 | 7A41900 | 116360008 | 183983017 | 32 |
| 530181000006114 | Bypass iliac artery by aorta/ext iliac art anastomosis NEC | 7A41A | 7A41A00 | 116360008 | 183983017 | 32 |
| 530211000006113 | Bypass iliac artery by iliac/iliac artery anastomosis NEC | 7A41D | 7A41D00 | 116360008 | 183983017 | 32 |
| 271865013 | Emergency bypass of iliac artery by unspecified anastomosis | 7A41E | 7A41E00 | 405477007 | 2157280014 | 32 |
| 271867017 | Other specified other bypass of iliac artery | 7A41y | 7A41y00 | 116360008 | 183983017 | 32 |
| 271868010 | Other bypass of iliac artery NOS | 7A41z | 7A41z00 | 116360008 | 183983017 | 32 |
| 271891014 | Operation on aneurysm of iliac artery NEC | 7A432 | 7A43200 | 265516003 | 394326010 | 32 |
| 271900018 | Percutaneous transluminal angioplasty of iliac artery | 7A440 | 7A44000 | 175612005 | 271900018 | 32 |
| 640181000006118 | Emergency replacement of aneurysmal femoral/popliteal artery | 7A45 | 7A45.00 | 265524008 | 394336019 | 32 |
| 639761000006115 | Emerg replace aneurysm fem art by fem/pop art anast c prosth | 7A450 | 7A45000 | 175620007 | 271916016 | 32 |
| 639851000006111 | Emerg replace aneurysm pop art by pop/pop art anast c prosth | 7A451 | 7A45100 | 175621006 | 271917013 | 32 |
| 639921000006111 | Emerg replacement aneurysmal femoral/popl art by anastomosis | 7A45-1 | 7A45.11 | 275255004 | 411215012 | 32 |
| 349676019 | Emergency replacement of aneurysmal common femoral artery | 7A45-2 | 7A45.12 | 233368003 | 349676019 | 32 |
| 349675015 | Emergency replacement of aneurysmal deep femoral artery | 7A45-3 | 7A45.13 | 233367008 | 349675015 | 32 |
| 349674016 | Emergency replacement of aneurysmal popliteal artery | 7A45-4 | 7A45.14 | 233366004 | 349674016 | 32 |
| 640151000006114 | Emergency replacement aneurysmal superficial femoral artery | 7A45-5 | 7A45.15 | 233365000 | 349673010 | 32 |
| 639751000006117 | Emerg replace aneurysm fem art by fem/pop anast c vein graft | 7A452 | 7A45200 | 175622004 | 271918015 | 32 |
| 639781000006113 | Emerg replace aneurysm femoral art by fem/tib a anast c pros | 7A454 | 7A45400 | 175624003 | 271920017 | 32 |
| 639871000006118 | Emerg replace aneurysm pop art by pop/tib art anast c prosth | 7A455 | 7A45500 | 175625002 | 271921018 | 32 |
| 639861000006113 | Emerg replace aneurysm pop art by pop/tib anast c vein graft | 7A457 | 7A45700 | 175627005 | 271923015 | 32 |
| 639741000006119 | Emerg replace aneurysm fem art by fem/peron a anast c prosth | 7A458 | 7A45800 | 175628000 | 271924014 | 32 |
| 639841000006114 | Emerg replace aneurysm pop art by pop/peron a anast c prosth | 7A459 | 7A45900 | 175629008 | 271925010 | 32 |
| 639771000006110 | Emerg replace aneurysm fem artery by fem/fem art anastomosis | 7A45C | 7A45C00 | 175632006 | 271928012 | 32 |
| 639881000006115 | Emerg replace aneurysm pop artery by pop/fem art anastomosis | 7A45D | 7A45D00 | 175633001 | 271929016 | 32 |
| 640141000006112 | Emergency replacement aneurysmal femoral/popliteal artery OS | 7A45y | 7A45y00 | 265524008 | 394336019 | 32 |
| 640131000006119 | Emergency replacement aneurysmal femoral/popliteal art NOS | 7A45z | 7A45z00 | 265524008 | 394336019 | 32 |
| 394337011 | Other replacement of aneurysmal femoral artery | 7A46 | 7A46.00 | 405463009 | 2157266019 | 32 |
| 173511000006113 | Replace aneurysm fem art by fem/pop art anastom c prosth NEC | 7A460 | 7A46000 | 112828007 | 184069011 | 32 |
| 173611000006114 | Replace aneurysm pop art by pop/pop art anastom c prosth NEC | 7A461 | 7A46100 | 233384002 | 349710011 | 32 |
| 17311000006112 | Other replacement aneurysmal femoral artery by anastomosis | 7A46-1 | 7A46.11 | 405463009 | 2157266019 | 32 |
| 410970013 | Other replacement of aneurysmal common femoral artery | 7A46-2 | 7A46.12 | 405463009 | 2157266019 | 32 |
| 410972017 | Other replacement of aneurysmal deep femoral artery | 7A46-3 | 7A46.13 | 405463009 | 2157266019 | 32 |
| 410973010 | Other replacement of aneurysmal popliteal artery | 7A46-4 | 7A46.14 | 77558004 | 128741010 | 32 |
| 410969012 | Other replacement of aneurysmal superficial femoral artery | 7A46-5 | 7A46.15 | 405463009 | 2157266019 | 32 |
| 173501000006110 | Replace aneurysm fem art by fem/pop a anast c vein graft NEC | 7A462 | 7A46200 | 112828007 | 184069011 | 32 |
| 173601000006111 | Replace aneurysm pop art by pop/pop a anast c vein graft NEC | 7A463 | 7A46300 | 233384002 | 349710011 | 32 |
| 173531000006119 | Replace aneurysm fem art by fem/tib art anast c prosth NEC | 7A464 | 7A46400 | 233383008 | 349706013 | 32 |
| 173631000006115 | Replace aneurysm pop art by pop/tib art anast c prosth NEC | 7A465 | 7A46500 | 233384002 | 349710011 | 32 |
| 173521000006117 | Replace aneurysm fem art by fem/tib a anast c vein graft NEC | 7A466 | 7A46600 | 233383008 | 349706013 | 32 |
| 173621000006118 | Replace aneurysm pop art by pop/tib a anast c vein graft NEC | 7A467 | 7A46700 | 233384002 | 349710011 | 32 |
| 173491000006119 | Replace aneurysm fem art by fem/peron art anast c prosth NEC | 7A468 | 7A46800 | 233383008 | 349706013 | 32 |
| 173591000006115 | Replace aneurysm pop art by pop/peron art anast c prosth NEC | 7A469 | 7A46900 | 233384002 | 349710011 | 32 |
| 173541000006112 | Replace aneurysm fem artery by fem/fem art anastomosis NEC | 7A46C | 7A46C00 | 44662004 | 74498014 | 32 |
| 173641000006113 | Replace aneurysm popliteal artery by pop/fem anastomosis NEC | 7A46D | 7A46D00 | 112828007 | 184069011 | 32 |
| 17361000006110 | Other replacement of aneurysmal femoral/popliteal artery OS | 7A46y | 7A46y00 | 405516005 | 2157318011 | 32 |
| 17351000006113 | Other replacement of aneurysmal femoral/popliteal artery NOS | 7A46z | 7A46z00 | 405516005 | 2157318011 | 32 |
| 394338018 | Other emergency bypass of femoral artery or popliteal artery | 7A47 | 7A47.00 | 405516005 | 2157318011 | 32 |
| 349671012 | Other emergency bypass of common femoral artery | 7A47-2 | 7A47.12 | 116360008 | 183983017 | 32 |
| 349670013 | Other emergency bypass of deep femoral artery | 7A47-3 | 7A47.13 | 116360008 | 183983017 | 32 |
| 349668016 | Other emergency bypass of superficial femoral artery | 7A47-5 | 7A47.15 | 116360008 | 183983017 | 32 |
| 349667014 | Other emergency bypass of femoral artery | 7A47-6 | 7A47.16 | 405463009 | 2157266019 | 32 |
| 639461000006110 | Emerg bypass femoral artery by fem/fem art anastomosis NEC | 7A47C | 7A47C00 | 116360008 | 183983017 | 32 |
| 394339014 | Other bypass of femoral artery or popliteal artery | 7A48 | 7A48.00 | 405469008 | 2157272019 | 32 |
| 530131000006113 | Bypass femoral artery by fem/pop art anast c prosthesis NEC | 7A480 | 7A48000 | 312688006 | 1780381000000111 | 32 |
| 349665018 | Other bypass of common femoral artery | 7A48-2 | 7A48.12 | 405463009 | 2157266019 | 32 |
| 349664019 | Other bypass of deep femoral artery | 7A48-3 | 7A48.13 | 405463009 | 2157266019 | 32 |
| 349663013 | Other bypass of femoral artery | 7A48-4 | 7A48.14 | 405463009 | 2157266019 | 32 |
| 349661010 | Other bypass of superficial femoral artery | 7A48-6 | 7A48.16 | 405463009 | 2157266019 | 32 |
| 530141000006115 | Bypass femoral artery by fem/pop art anast c vein graft NEC | 7A482 | 7A48200 | 50807007 | 1780371000000114 | 32 |
| 530151000006118 | Bypass femoral artery by fem/tib art anast c prosthesis NEC | 7A484 | 7A48400 | 233383008 | 349706013 | 32 |
| 530161000006116 | Bypass femoral artery by fem/tib art anast c vein graft NEC | 7A486 | 7A48600 | 448529008 | 2899845012 | 32 |
| 530111000006119 | Bypass femoral artery by fem/peron a anast c prosthesis NEC | 7A488 | 7A48800 | 233383008 | 349706013 | 32 |
| 530121000006110 | Bypass femoral artery by fem/peron a anast c vein graft NEC | 7A48A | 7A48A00 | 448323005 | 2901040016 | 32 |
| 530171000006111 | Bypass femoral artery by femoral/femoral art anastomosis NEC | 7A48C | 7A48C00 | 44662004 | 74498014 | 32 |
| 36061000006118 | Other bypass of femoral artery or popliteal artery OS | 7A48y | 7A48y00 | 405469008 | 2157272019 | 32 |
| 272003016 | Other bypass of femoral artery or popliteal artery NOS | 7A48z | 7A48z00 | 405469008 | 2157272019 | 32 |
| 272036015 | Ligation of aneurysm of popliteal artery | 7A4A4 | 7A4A400 | 175706005 | 272036015 | 32 |
| 272037012 | Operation on aneurysm of femoral artery NEC | 7A4A5 | 7A4A500 | 265528006 | 394340011 | 32 |
| 272052017 | Percutaneous transluminal angioplasty of femoral artery | 7A4B0 | 7A4B000 | 175715003 | 272052017 | 32 |
| 239491000006117 | Percutaneous transluminal angioplasty of popliteal artery | 7A4B1 | 7A4B100 | 175716002 | 272053010 | 32 |
| 2537052019 | DNA - Did not attend peripheral vascular disease clinic | 9N4h | 9N4h.00 | 413988000 | 2537052019 | 37 |
| 84961000006116 | Type II diabetes mellitus with peripheral angiopathy | C109F-1 | C109F11 | 314902007 | 459306016 | 32 |
| 84591000006119 | Type 2 diabetes mellitus with peripheral angiopathy | C109F-2 | C109F12 | 314902007 | 459308015 | 32 |
| 913931000006116 | Type 1 diabetes mellitus with peripheral angiopathy | C10EG | C10EG00 | 31211000119101 | 3315042015 | 32 |
| 459308015 | Type 2 diabetes mellitus with peripheral angiopathy | C10FF | C10FF00 | 314902007 | 459308015 | 32 |
| 459306016 | Type II diabetes mellitus with peripheral angiopathy | C10FF-1 | C10FF11 | 314902007 | 459306016 | 32 |
| 300437018 | Thoracic aortic aneurysm which has ruptured | G711 | G711.00 | 195258006 | 300437018 | 32 |
| 300438011 | Ruptured thoracic aortic aneurysm | G711-1 | G711.11 | 195258006 | 300438011 | 32 |
| 124373014 | Thoracic aortic aneurysm without mention of rupture | G712 | G712.00 | 74883004 | 124373014 | 32 |
| 1221083011 | Abdominal aortic aneurysm which has ruptured | G713 | G713.00 | 14336007 | 1221083011 | 32 |
| 458502011 | Ruptured suprarenal aortic aneurysm | G7130 | G713000 | 314184006 | 458502011 | 32 |
| 24379014 | Ruptured abdominal aortic aneurysm | G713-1 | G713.11 | 14336007 | 24379014 | 32 |
| 300441019 | Abdominal aortic aneurysm without mention of rupture | G714 | G714.00 | 233985008 | 350580017 | 32 |
| 458503018 | Juxtarenal aortic aneurysm | G7140 | G714000 | 314185007 | 458503018 | 32 |
| 300442014 | AAA - Abdominal aortic aneurysm without mention of rupture | G714-1 | G714.11 | 233985008 | 350580017 | 32 |
| 300445011 | Ruptured aortic aneurysm NOS | G715 | G715.00 | 73067008 | 121362018 | 32 |
| 300446012 | Thoracoabdominal aortic aneurysm, ruptured | G7150 | G715000 | 195265003 | 300446012 | 32 |
| 300448013 | Thoracoabdominal aortic aneurysm, without mention of rupture | G7160 | G716000 | 233984007 | 350579015 | 32 |
| 300449017 | Leaking abdominal aortic aneurysm | G718 | G718.00 | 195268001 | 300449017 | 32 |
| 484314018 | Aneurysm of artery of arm | G720 | G720.00 | 29495008 | 484314018 | 32 |
| 300453015 | Aneurysm of brachial artery | G7200 | G720000 | 195271009 | 300453015 | 32 |
| 300454014 | Aneurysm of radial artery | G7201 | G720100 | 195272002 | 300454014 | 32 |
| 300457019 | Aneurysm of ulnar artery | G7202 | G720200 | 195273007 | 300457019 | 32 |
| 300458012 | Aneurysm of arm artery NOS | G720z | G720z00 | 29495008 | 49360017 | 32 |
| 22740018 | Aneurysm of iliac artery | G722 | G722.00 | 13290008 | 22740018 | 32 |
| 300460014 | Aneurysm of common iliac artery | G7220 | G722000 | 195275000 | 300460014 | 32 |
| 300463011 | Aneurysm of external iliac artery | G7221 | G722100 | 195276004 | 300463011 | 32 |
| 300464017 | Aneurysm of internal iliac artery | G7222 | G722200 | 195277008 | 300464017 | 32 |
| 300466015 | Aneurysm of iliac artery NOS | G722z | G722z00 | 13290008 | 22740018 | 32 |
| 480891014 | Aneurysm of leg artery | G723 | G723.00 | 20981007 | 480891014 | 32 |
| 151487012 | Aneurysm of femoral artery | G7230 | G723000 | 91445000 | 151487012 | 32 |
| 80421013 | Aneurysm of popliteal artery | G7231 | G723100 | 48273005 | 80421013 | 32 |
| 300468019 | Aneurysm of anterior tibial artery | G7232 | G723200 | 195279006 | 300468019 | 32 |
| 300469010 | Aneurysm of dorsalis pedis artery | G7233 | G723300 | 195280009 | 300469010 | 32 |
| 300472015 | Aneurysm of posterior tibial artery | G7234 | G723400 | 195281008 | 300472015 | 32 |
| 455424010 | Ruptured popliteal artery aneurysm | G7235 | G723500 | 311798009 | 455424010 | 32 |
| 216721000006114 | Post radiological femoral false aneurysm | G7236 | G723600 | 312374002 | 456075013 | 32 |
| 300477014 | Aneurysm of leg artery NOS | G723z | G723z00 | 20981007 | 35275015 | 32 |
| 63716015 | Aneurysm of subclavian artery | G72y4 | G72y400 | 40136003 | 63716015 | 32 |
| 116959011 | Aneurysm of splenic artery | G72y5 | G72y500 | 70405009 | 116959011 | 32 |
| 69629017 | Aneurysm of axillary artery | G72y6 | G72y600 | 41740005 | 69629017 | 32 |
| 199806019 | Aneurysm of coeliac artery | G72y7 | G72y700 | 111290000 | 199806019 | 32 |
| 108827017 | Aneurysm of superior mesenteric artery | G72y8 | G72y800 | 65498003 | 108827017 | 32 |
| 300482019 | Aneurysm of inferior mesenteric artery | G72y9 | G72y900 | 195289005 | 300482019 | 32 |
| 43353015 | Aneurysm of hepatic artery | G72yA | G72yA00 | 25878000 | 43353015 | 32 |
| 395791015 | Other peripheral vascular disease | G73 | G73..00 | 400047006 | 1779317016 | 32 |
| 87208017 | Thromboangiitis obliterans | G731 | G731.00 | 52403007 | 87208017 | 32 |
| 87210015 | Buerger's disease | G7310 | G731000 | 52403007 | 87210015 | 32 |
| 300510018 | Other specified peripheral vascular disease | G73y | G73y.00 | 400047006 | 1779317016 | 32 |
| 302011 | Diabetic peripheral angiopathy | G73y0 | G73y000 | 127014009 | 302011 | 32 |
| 300514010 | Other specified peripheral vascular disease NOS | G73yz | G73yz00 | 400047006 | 1779317016 | 32 |
| 235911000006116 | Peripheral vascular disease NOS | G73z | G73z.00 | 400047006 | 1779317016 | 32 |
| 105536013 | Intermittent claudication | G73z0 | G73z000 | 63491006 | 105536013 | 32 |
| 300515011 | Peripheral vascular disease NOS | G73zz | G73zz00 | 400047006 | 1779317016 | 32 |
| 458506014 | Femoral artery occlusion | G76z1 | G76z100 | 314188009 | 458506014 | 32 |
| 458507017 | Popliteal artery occlusion | G76z2 | G76z200 | 314189001 | 458507017 | 32 |
| 300963017 | [X]Other specified peripheral vascular diseases | Gyu74 | Gyu7400 | 400047006 | 1779317016 | 32 |
| 218531000000113 | Aortic atherosclerosis | G700 | G700.00 | 81817003 | 135728018 | 32 |
| 742481000006118 | Ischaemia of legs | G73-2 | G73..12 | 233961000 | 350546015 | 32 |
| 317343014 | [D]Gangrene | R054 | R054.00 | 372070002 | 1210648015 | 32 |
| 350535018 | Peripheral ischaemic vascular disease | G73-1 | G73..11 | 233958001 | 350535018 | 32 |
| 357895013 | Ischaemic leg ulcer | M271-2 | M271.12 | 238793001 | 357895013 | 32 |
| 350533013 | Peripheral ischaemia | G73-3 | G73..13 | 233958001 | 350533013 | 32 |
| 411512011 | Claudication | G73z0-1 | G73z011 | 63491006 | 3559347018 | 32 |
| 300504018 | Peripheral gangrene | G732 | G732.00 | 195301007 | 300504018 | 32 |
| 300506016 | Gangrene of foot | G7321 | G732100 | 195303005 | 300506016 | 32 |
| 292583018 | Type 2 diabetes mellitus with gangrene | C10F5 | C10F500 | 421631007 | 2618206011 | 32 |
| 300433019 | Extremity artery atheroma | G702 | G702.00 | 195254008 | 300433019 | 32 |
| 300550015 | Peripheral arterial embolism and thrombosis NOS | G742z | G742z00 | 195318006 | 300534011 | 32 |
| 300434013 | Extremity artery atheroma NOS | G702z | G702z00 | 195254008 | 300433019 | 32 |
| 300511019 | Peripheral angiopathic disease EC NOS | G73y1 | G73y100 | 400047006 | 1779317016 | 32 |
| 309177010 | Ischaemic ulcer diabetic foot | M2710 | M271000 | 201250006 | 309177010 | 32 |
| 317348017 | [D]Widespread diabetic foot gangrene | R0543 | R054300 | 195303005 | 300506016 | 32 |
| 616351000006115 | Diabetes mellitus with gangrene | C107-1 | C107.11 | 422275004 | 2616612015 | 32 |
| 616831000006118 | Diabetes with gangrene | C107-2 | C107.12 | 422275004 | 2616612015 | 32 |
| 616561000006113 | Diabetes mellitus, adult with gangrene | C1072 | C107200 | 421631007 | 2618206011 | 32 |
| 616441000006113 | Diabetes mellitus with peripheral circulatory disorder | C107 | C107.00 | 421895002 | 3688501014 | 32 |
| 317349013 | [D]Gangrene NOS | R054z | R054z00 | 372070002 | 1490849015 | 32 |
| 300502019 | Presenile gangrene | G7311 | G731100 | 195299000 | 300502019 | 32 |
| 281211000006117 | Non-insulin dependent diabetes mellitus with gangrene | C1095 | C109500 | 421631007 | 2967779019 | 32 |
| 84511000006112 | Type 2 diabetes mellitus with gangrene | C1095-2 | C109512 | 421631007 | 2618206011 | 32 |
| 280561000006111 | Non-insulin-dependent d m with peripheral angiopath | C109F | C109F00 | 314902007 | 459307013 | 32 |
| 674961000006118 | NIDDM with peripheral circulatory disorder | C1074 | C107400 | 422166005 | 2623039019 | 32 |
| 771351000006111 | Insulin dependent diabetes mellitus with gangrene | C1086 | C108600 | 422275004 | 2616612015 | 32 |
| 84881000006119 | Type II diabetes mellitus with gangrene | C1095-1 | C109511 | 421631007 | 2618206011 | 32 |
| 616581000006115 | Diabetes mellitus, adult, + peripheral circulatory disorder | C1071 | C107100 | 422166005 | 2618204014 | 32 |
| 772141000006118 | Insulin dependent diab mell with peripheral angiopathy | C108G | C108G00 | 421895002 | 2618203015 | 32 |
| 292523015 | Diabetes mellitus NOS with peripheral circulatory disorder | C107z | C107z00 | 421895002 | 2618203015 | 32 |
| 300503012 | Thromboangiitis obliterans NOS | G731z | G731z00 | 52403007 | 87208017 | 32 |
| 787101000006114 | IDDM with peripheral circulatory disorder | C1073 | C107300 | 421895002 | 2618203015 | 32 |
| 292541011 | Type 1 diabetes mellitus with gangrene | C10E6 | C10E600 | 420825003 | 2618207019 | 32 |
| 616591000006117 | Diabetes mellitus, juvenile +peripheral circulatory disorder | C1070 | C107000 | 421365002 | 2618205010 | 32 |
| 443199013 | Ischaemic foot | G733 | G733.00 | 301755001 | 443199013 | 32 |
| 1176621000000116 | Referred for peripheral artery disease assessment | 8HlP | 8HlP.00 | 527251000000101 | 1176621000000116 | 40 |
| 1715241000006112 | Vascular claudication | G73z0-2 | G73z012 | 63491006 | 105536013 | 32 |
| 292543014 | Type I diabetes mellitus with gangrene | C10E6-1 | C10E611 | 420825003 | 2618207019 | 32 |

Supplementary Table S14: Additional Read Codes for Reynold's Risk Score. The Reynolds Risk Score consists of the read codes for stroke or myocardial infarction in Table S12, plus these additional codes for coronary revascularisation.

| MedCode Id | Term | Original Read Code | Cleansed Read Code | Snomed CT Concept Id | Snomed CT Description Id | Emis Code Category Id |
| --- | --- | --- | --- | --- | --- | --- |
| 1228282017 | Saphenous vein graft replacement of coronary artery | 7920 | 7920.00 | 3546002 | 6979016 | 32 |
| 271073015 | Saphenous vein graft replacement of one coronary artery | 79200 | 7920000 | 736967001 | 3525967012 | 32 |
| 271074014 | Saphenous vein graft replacement of two coronary arteries | 79201 | 7920100 | 736969003 | 3525975018 | 32 |
| 161451000006116 | Saphenous vein graft bypass of coronary artery | 7920-1 | 7920.11 | 3546002 | 1228282017 | 32 |
| 271075010 | Saphenous vein graft replacement of three coronary arteries | 79202 | 7920200 | 736968006 | 3525971010 | 32 |
| 161491000006110 | Saphenous vein graft replacement of four+ coronary arteries | 79203 | 7920300 | 736966005 | 3525965016 | 32 |
| 271080018 | Other autograft replacement of coronary artery | 7921 | 7921.00 | 232717009 | 348640013 | 32 |
| 271081019 | Autograft replacement of one coronary artery NEC | 79210 | 7921000 | 232719007 | 348647011 | 32 |
| 271082014 | Autograft replacement of two coronary arteries NEC | 79211 | 7921100 | 232720001 | 348649014 | 32 |
| 1221554018 | Other autograft bypass of coronary artery | 7921-1 | 7921.11 | 232717009 | 348640013 | 32 |
| 271083016 | Autograft replacement of three coronary arteries NEC | 79212 | 7921200 | 232721002 | 348651013 | 32 |
| 271084010 | Autograft replacement of four of more coronary arteries NEC | 79213 | 7921300 | 232722009 | 348654017 | 32 |
| 271088013 | Allograft replacement of coronary artery | 7922 | 7922.00 | 175021005 | 271087015 | 32 |
| 271089017 | Allograft replacement of one coronary artery | 79220 | 7922000 | 736971003 | 3525979012 | 32 |
| 271092018 | Allograft replacement of two coronary arteries | 79221 | 7922100 | 736973000 | 3525983012 | 32 |
| 271087015 | Allograft bypass of coronary artery | 7922-1 | 7922.11 | 175021005 | 271087015 | 32 |
| 271093011 | Allograft replacement of three coronary arteries | 79222 | 7922200 | 736972005 | 3525981014 | 32 |
| 271094017 | Allograft replacement of four or more coronary arteries | 79223 | 7922300 | 736970002 | 3525977014 | 32 |
| 271098019 | Prosthetic replacement of coronary artery | 7923 | 7923.00 | 175029007 | 271097012 | 32 |
| 271099010 | Prosthetic replacement of one coronary artery | 79230 | 7923000 | 736963002 | 3525947015 | 32 |
| 271100019 | Prosthetic replacement of two coronary arteries | 79231 | 7923100 | 736965009 | 3525955010 | 32 |
| 271097012 | Prosthetic bypass of coronary artery | 7923-1 | 7923.11 | 175029007 | 271097012 | 32 |
| 271101015 | Prosthetic replacement of three coronary arteries | 79232 | 7923200 | 736964008 | 3525952013 | 32 |
| 271102010 | Prosthetic replacement of four or more coronary arteries | 79233 | 7923300 | 736962007 | 3525944010 | 32 |
| 271105012 | Revision of bypass for coronary artery | 7924 | 7924.00 | 175036008 | 271105012 | 32 |
| 271106013 | Revision of bypass for one coronary artery | 79240 | 7924000 | 175037004 | 271106013 | 32 |
| 271107016 | Revision of bypass for two coronary arteries | 79241 | 7924100 | 175038009 | 271107016 | 32 |
| 271108014 | Revision of bypass for three coronary arteries | 79242 | 7924200 | 175039001 | 271108014 | 32 |
| 271109018 | Revision of bypass for four or more coronary arteries | 79243 | 7924300 | 175040004 | 271109018 | 32 |
| 271110011 | Revision of connection of thoracic artery to coronary artery | 79244 | 7924400 | 175041000 | 271110011 | 32 |
| 271111010 | Revision of implantation of thoracic artery into heart | 79245 | 7924500 | 175042007 | 271111010 | 32 |
| 271114019 | Connection of mammary artery to coronary artery | 7925 | 7925.00 | 175045009 | 271114019 | 32 |
| 394286010 | Double anastomosis of mammary arteries to coronary arteries | 79250 | 7925000 | 265481001 | 394286010 | 32 |
| 411212010 | LIMA sequential anastomosis | 79250-1 | 7925011 | 275252001 | 411212010 | 32 |
| 411213017 | RIMA sequential anastomosis | 79250-2 | 7925012 | 275253006 | 411213017 | 32 |
| 628021000006118 | Double implant of mammary arteries into coronary arteries | 79251 | 7925100 | 175047001 | 271120018 | 32 |
| 271115018 | Creation of bypass from mammary artery to coronary artery | 7925-1 | 7925.11 | 175045009 | 271115018 | 32 |
| 142361000006111 | Single anast mammary art to left ant descend coronary art | 79252 | 7925200 | 175048006 | 271121019 | 32 |
| 405092018 | Single anastomosis of mammary artery to coronary artery NEC | 79253 | 7925300 | 232719007 | 348647011 | 32 |
| 411159010 | LIMA single anastomosis | 79253-1 | 7925311 | 275215001 | 411159010 | 32 |
| 411160017 | RIMA single anastomosis | 79253-2 | 7925312 | 275216000 | 411160017 | 32 |
| 271126012 | Single implantation of mammary artery into coronary artery | 79254 | 7925400 | 175050003 | 271126012 | 32 |
| 271129017 | Connection of other thoracic artery to coronary artery | 7926 | 7926.00 | 232717009 | 348640013 | 32 |
| 627951000006112 | Double anastom thoracic arteries to coronary arteries NEC | 79260 | 7926000 | 232720001 | 348649014 | 32 |
| 628031000006115 | Double implant thoracic arteries into coronary arteries NEC | 79261 | 7926100 | 232720001 | 348649014 | 32 |
| 271132019 | Single anastomosis of thoracic artery to coronary artery NEC | 79262 | 7926200 | 232719007 | 348647011 | 32 |
| 142431000006114 | Single implantation thoracic artery into coronary artery NEC | 79263 | 7926300 | 232719007 | 348647011 | 32 |
| 271136016 | Other open operations on coronary artery | 7927 | 7927.00 | 31413008 | 52509013 | 32 |
| 271138015 | Transposition of coronary artery NEC | 79273 | 7927300 | 384678000 | 1479002017 | 32 |
| 271139011 | Exploration of coronary artery | 79274 | 7927400 | 175063009 | 271139011 | 32 |
| 1221061012 | Open angioplasty of coronary artery | 79275 | 7927500 | 14201006 | 1221061012 | 32 |
| 89711000006118 | Transluminal balloon angioplasty of coronary artery | 7928 | 7928.00 | 11101003 | 19234014 | 32 |
| 238641000006117 | Percut transluminal balloon angioplasty one coronary artery | 79280 | 7928000 | 68466008 | 1233048011 | 32 |
| 238571000006114 | Percut translum balloon angioplasty mult coronary arteries | 79281 | 7928100 | 85053006 | 506356013 | 32 |
| 238821000006111 | Percutaneous balloon coronary angioplasty | 7928-1 | 7928.11 | 11101003 | 19234014 | 32 |
| 238561000006119 | Percut translum balloon angioplasty bypass graft coronary a | 79282 | 7928200 | 175066001 | 271142017 | 32 |
| 1547311000006116 | Percut translum cutting balloon angioplasty coronary artery | 79283 | 7928300 | 707828002 | 3032475016 | 32 |
| 271149014 | Other therapeutic transluminal operations on coronary artery | 7929 | 7929.00 | 31413008 | 52509013 | 32 |
| 484402013 | Percutaneous transluminal laser coronary angioplasty | 79290 | 7929000 | 29843007 | 484402013 | 32 |
| 238651000006115 | Percut transluminal coronary thrombolysis with streptokinase | 79291 | 7929100 | 175071008 | 271150014 | 32 |
| 238581000006112 | Percut translum coronary thrombolytic therapy- streptokinase | 79291-1 | 7929111 | 175071008 | 271151013 | 32 |
| 238601000006119 | Percut translum inject therap subst to coronary artery NEC | 79292 | 7929200 | 232731009 | 348671016 | 32 |
| 1785743019 | Rotary blade coronary angioplasty | 79293 | 7929300 | 397193006 | 1785743019 | 32 |
| 2536354015 | Insertion of coronary artery stent | 79294 | 7929400 | 36969009 | 2536354015 | 32 |
| 311461000000116 | Insertion of drug-eluting coronary artery stent | 79295 | 7929500 | 203741000000101 | 311461000000116 | 32 |
| 354901000000117 | Percutaneous transluminal atherectomy of coronary artery | 79296 | 7929600 | 232726007 | 354901000000117 | 32 |
| 220231000000118 | Coronary artery operations | 792 | 792..00 | 31413008 | 484868016 | 32 |
| 161481000006112 | Saphenous vein graft replacement of coronary artery OS | 7920y | 7920y00 | 3546002 | 6979016 | 32 |
| 271079016 | Saphenous vein graft replacement coronary artery NOS | 7920z | 7920z00 | 3546002 | 6979016 | 32 |
| 348644016 | Coronary artery bypass graft operations | 792-1 | 792..11 | 232717009 | 348644016 | 32 |
| 36861000006113 | Other autograft replacement of coronary artery OS | 7921y | 7921y00 | 232717009 | 348640013 | 32 |
| 271086012 | Other autograft replacement of coronary artery NOS | 7921z | 7921z00 | 232717009 | 348640013 | 32 |
| 271095016 | Other specified allograft replacement of coronary artery | 7922y | 7922y00 | 175021005 | 271087015 | 32 |
| 271096015 | Allograft replacement of coronary artery NOS | 7922z | 7922z00 | 175021005 | 271087015 | 32 |
| 271103017 | Other specified prosthetic replacement of coronary artery | 7923y | 7923y00 | 175029007 | 271097012 | 32 |
| 271104011 | Prosthetic replacement of coronary artery NOS | 7923z | 7923z00 | 175029007 | 271097012 | 32 |
| 271112015 | Other specified revision of bypass for coronary artery | 7924y | 7924y00 | 175036008 | 271105012 | 32 |
| 271113013 | Revision of bypass for coronary artery NOS | 7924z | 7924z00 | 175036008 | 271105012 | 32 |
| 589721000006114 | Connection of mammary artery to coronary artery OS | 7925y | 7925y00 | 175045009 | 271114019 | 32 |
| 271128013 | Connection of mammary artery to coronary artery NOS | 7925z | 7925z00 | 175045009 | 271114019 | 32 |
| 589781000006113 | Connection of other thoracic artery to coronary artery OS | 7926y | 7926y00 | 232717009 | 348640013 | 32 |
| 271135017 | Connection of other thoracic artery to coronary artery NOS | 7926z | 7926z00 | 232717009 | 348640013 | 32 |
| 271140013 | Other specified other open operation on coronary artery | 7927y | 7927y00 | 31413008 | 52509013 | 32 |
| 271141012 | Other open operation on coronary artery NOS | 7927z | 7927z00 | 31413008 | 52509013 | 32 |
| 89731000006112 | Transluminal balloon angioplasty of coronary artery OS | 7928y | 7928y00 | 11101003 | 19234014 | 32 |
| 271148018 | Transluminal balloon angioplasty of coronary artery NOS | 7928z | 7928z00 | 11101003 | 19234014 | 32 |
| 250941000006110 | Other therapeutic transluminal op on coronary artery OS | 7929y | 7929y00 | 31413008 | 52509013 | 32 |
| 250931000006117 | Other therapeutic transluminal op on coronary artery NOS | 7929z | 7929z00 | 31413008 | 52509013 | 32 |
| 271157012 | Diagnostic transluminal operations on coronary artery | 792A | 792A.00 | 175076003 | 271157012 | 32 |
| 619381000006119 | Diagnostic transluminal operation on coronary artery OS | 792Ay | 792Ay00 | 175076003 | 271157012 | 32 |
| 271160017 | Diagnostic transluminal operation on coronary artery NOS | 792Az | 792Az00 | 175076003 | 271157012 | 32 |
| 271161018 | Repair of coronary artery NEC | 792B | 792B.00 | 31413008 | 52509013 | 32 |
| 271162013 | Endarterectomy of coronary artery NEC | 792B0 | 792B000 | 20738002 | 34888017 | 32 |
| 271163015 | Other specified repair of coronary artery | 792By | 792By00 | 31413008 | 52509013 | 32 |
| 271164014 | Repair of coronary artery NOS | 792Bz | 792Bz00 | 31413008 | 52509013 | 32 |
| 271165010 | Other replacement of coronary artery | 792C | 792C.00 | 232717009 | 348640013 | 32 |
| 271166011 | Replacement of coronary arteries using multiple methods | 792C0 | 792C000 | 175085003 | 271166011 | 32 |
| 271167019 | Other specified replacement of coronary artery | 792Cy | 792Cy00 | 232717009 | 348640013 | 32 |
| 271168012 | Replacement of coronary artery NOS | 792Cz | 792Cz00 | 232717009 | 348640013 | 32 |
| 271169016 | Other bypass of coronary artery | 792D | 792D.00 | 232717009 | 348640013 | 32 |
| 271170015 | Other specified other bypass of coronary artery | 792Dy | 792Dy00 | 232717009 | 348640013 | 32 |
| 271171016 | Other bypass of coronary artery NOS | 792Dz | 792Dz00 | 232717009 | 348640013 | 32 |
| 271172011 | Other specified operations on coronary artery | 792y | 792y.00 | 31413008 | 52509013 | 32 |
| 271173018 | Coronary artery operations NOS | 792z | 792z.00 | 31413008 | 484868016 | 32 |
| 1547631000006117 | Perc translumin balloon angioplasty stenting coronary artery | 793G | 793G.00 | 429639007 | 2694928017 | 32 |
| 1547641000006110 | Perc translum ball angio insert 1-2 drug elut stents cor art | 793G0 | 793G000 | 936451000000108 | 2398371000000113 | 32 |
| 1547651000006112 | Perc tran ball angio ins 3 or more drug elut stents cor art | 793G1 | 793G100 | 936451000000108 | 2398371000000113 | 32 |
| 1547661000006114 | Perc translum balloon angioplasty insert 1-2 stents cor art | 793G2 | 793G200 | 429639007 | 2694928017 | 32 |
| 1547671000006119 | Percutaneous cor balloon angiop 3 more stents cor art NEC | 793G3 | 793G300 | 429639007 | 2694928017 | 32 |
| 1547691000006118 | Perc translum balloon angioplasty stenting coronary art NOS | 793Gz | 793Gz00 | 429639007 | 2694928017 | 32 |
| 1547711000006115 | Percutaneous transluminal balloon dilation cardiac conduit | 793H0 | 793H000 | 707813002 | 3032442012 | 32 |
| 272117011 | Rotary blade angioplasty | 7A545 | 7A54500 | 175764006 | 272117011 | 32 |
| 272295013 | Peroperative angioplasty | 7A6G1 | 7A6G100 | 175882004 | 272295013 | 32 |
| 456362017 | Prosthetic graft patch angioplasty | 7A6H3 | 7A6H300 | 312610006 | 456362017 | 32 |
| 456363010 | Percutaneous transluminal angioplasty of vascular graft | 7A6H4 | 7A6H400 | 312611005 | 456363010 | 32 |
| 460686018 | [V]Presence of aortocoronary bypass graft | ZV457 | ZV45700 | 399261000 | 1767181012 | 32 |
| 460687010 | [V]Presence of coronary angioplasty implant and graft | ZV458 | ZV45800 | 428375006 | 2692299011 | 32 |
| 451132018 | [V]Presence of coronary artery bypass graft | ZV45K | ZV45K00 | 399261000 | 1767181012 | 32 |
| 1227364015 | [V]Presence of coronary artery bypass graft - CABG | ZV45K-1 | ZV45K11 | 399261000 | 2986657017 | 32 |
| 453269016 | [V]Status following coronary angioplasty NOS | ZV45L | ZV45L00 | 105501005 | 169590017 | 32 |

1. **Risk Predictor Codes**

Supplementary Table S15: Read Codes for Family History of Coronary Heart Disease.

| MedCode Id | Term | Original Read Code | Cleansed Read Code | Snomed CT Concept Id | Snomed CT Description Id | Emis Code Category Id |
| --- | --- | --- | --- | --- | --- | --- |
| 411053013 | FH: Angina | 12C-4 | 12C..14 | 275121006 | 411053013 | 7 |
| 397694017 | FH: Ischaemic heart dis. <60 | 12C2 | 12C2.00 | 266895004 | 397694017 | 7 |
| 217371000000119 | FH: Myocardial infarction < 60 | 12C2-1 | 12C2.11 | 275122004 | 411054019 | 7 |
| 217381000000117 | FH: MI- Myocardial infarct <60 | 12C2-2 | 12C2.12 | 275122004 | 411054019 | 7 |
| 217391000000115 | FH: Angina < 60yrs | 12C2-3 | 12C2.13 | 275932007 | 411905011 | 7 |
| 397701010 | FH: Myocardial infarction | 12C5 | 12C5.00 | 266897007 | 397701010 | 7 |
| 411056017 | FH: Coronary thrombosis | 12C5-1 | 12C5.11 | 275124003 | 411056017 | 7 |
| 437729010 | FH: Ischaemic heart disease | 12C5-2 | 12C5.12 | 297242006 | 437729010 | 7 |
| 460138014 | FH myocardial infarction male first degree age known | 12CA | 12CA.00 | 315619001 | 460138014 | 7 |
| 460139018 | FH myocardial infarction male first degree age unknown | 12CB | 12CB.00 | 315620007 | 460139018 | 7 |
| 460140016 | FH myocardial infarction female first degree age known | 12CC | 12CC.00 | 315621006 | 460140016 | 7 |
| 460141017 | FH myocardial infarction female first degree age unknown | 12CD | 12CD.00 | 315622004 | 460141017 | 7 |
| 460142012 | FH angina male first degree age known | 12CE | 12CE.00 | 315623009 | 460142012 | 7 |
| 460144013 | FH angina male first degree age unknown | 12CF | 12CF.00 | 315625002 | 460144013 | 7 |
| 460145014 | FH angina female first degree age known | 12CG | 12CG.00 | 315626001 | 460145014 | 7 |
| 460146010 | FH angina female first degree age unknown | 12CH | 12CH.00 | 315627005 | 460146010 | 7 |
| 216245011 | FH: premature coronary heart disease | 12CI | 12CI.00 | 134439009 | 216245011 | 7 |
| 1780270012 | FH: Angina in 1st degree female relative <65 years | 12CL | 12CL.00 | 401065001 | 1780270012 | 7 |
| 1780323013 | FH: Angina in 1st degree male relative <55 years | 12CM | 12CM.00 | 401122004 | 1780323013 | 7 |
| 1780271011 | FH: Myocardial infarct in 1st degree female relative <65 yrs | 12CN | 12CN.00 | 401066000 | 2839705011 | 7 |
| 1780272016 | FH: Myocardial infarct in 1st degree male relative <55 years | 12CP | 12CP.00 | 401067009 | 1780272016 | 7 |
| 404428013 | [V]Family history of ischaemic heart disease | ZV173 | ZV17300 | 297242006 | 437728019 | 7 |
| 339481000006111 | [V]Family history of ischaemic heart disease (IHD) | ZV173-1 | ZV17311 | 297242006 | 437728019 | 7 |
| 339631000006113 | [V]Family history of myocardial infarction | ZV173-2 | ZV17312 | 266897007 | 397700011 | 7 |
| 386581000006110 | [X]Family hist/ischaemic hrt disease+oth dis/circultr system | ZVu66 | ZVu6600 | 266894000 | 397693011 | 32 |

Supplementary Table S16: Read Codes for Systemic Lupus Erythematosus

| MedCode Id | Term | Original Read Code | Cleansed Read Code | Snomed CT Concept Id | Snomed CT Description Id | Emis Code Category Id |
| --- | --- | --- | --- | --- | --- | --- |
| 92208011 | Systemic lupus erythematosus | N000 | N000.00 | 55464009 | 92208011 | 32 |
| 92209015 | Disseminated lupus erythematosus | N0000 | N000000 | 55464009 | 92209015 | 32 |
| 309408013 | Systemic lupus erythematosus NOS | N000z | N000z00 | 55464009 | 92208011 | 32 |
| 114251000006118 | Systemic lupus erythematosus with organ or sys involv | N0003 | N000300 | 239887007 | 359443016 | 32 |
| 453243010 | Systemic lupus erythematosus with pericarditis | N0004 | N000400 | 309762007 | 453243010 | 32 |
| 309405011 | Drug-induced systemic lupus erythematosus | N0002 | N000200 | 201436003 | 309405011 | 5 |
| 158372014 | Neonatal lupus erythematosus | N0005 | N000500 | 95609003 | 158372014 | 32 |
| 512234017 | Cerebral lupus | N0006 | N000600 | 95644001 | 512234017 | 32 |
| 496853017 | Libman-Sacks disease | N0001 | N000100 | 54072008 | 496853017 | 32 |

Supplementary Table S17: Read Codes for Atrial Fibrilation

| MedCode Id | Term | Original Read Code | Cleansed Read Code | Snomed CT Concept Id | Snomed CT Description Id |
| --- | --- | --- | --- | --- | --- |
| 456154015 | H/O: atrial fibrillation | 14AN | 14AN.00 | 312442005 | 456154015 |
| 2692063011 | History of atrial flutter | 14AR | 14AR.00 | 428076002 | 2692063011 |
| 294601000000110 | Atrial fibrillation resolved | 212R | 212R.00 | 196371000000102 | 294601000000110 |
| 216183015 | Atrial fibrillation monitoring | 662S | 662S.00 | 134377004 | 216183015 |
| 406861000000119 | Atrial fibrillation annual review | 6A9 | 6A9..00 | 248411000000105 | 406861000000119 |
| 406251000000118 | Atrial fibrillation monitoring administration | 9Os | 9Os..00 | 713801000000102 | 1565041000000112 |
| 408081000000116 | Atrial fibrillation monitoring first letter | 9Os0 | 9Os0.00 | 717221000000101 | 1569761000000112 |
| 408101000000110 | Atrial fibrillation monitoring second letter | 9Os1 | 9Os1.00 | 716981000000106 | 1569531000000111 |
| 408121000000118 | Atrial fibrillation monitoring third letter | 9Os2 | 9Os2.00 | 716181000000109 | 1568741000000115 |
| 408141000000113 | Atrial fibrillation monitoring verbal invite | 9Os3 | 9Os3.00 | 717011000000100 | 1570401000000119 |
| 408241000000115 | Atrial fibrillation monitoring telephone invite | 9Os4 | 9Os4.00 | 716721000000107 | 1570381000000119 |
| 294171000000112 | Exception reporting: atrial fibrillation quality indicators | 9hF | 9hF..00 | 716871000000101 | 1000011000006112 |
| 1000031000006118 | Excepted from atrial fibrillation qual indic: Inform dissent | 9hF1 | 9hF1.00 | 717381000000109 | 1000031000006118 |
| 421235014 | Paroxysmal atrial fibrillation | G5732 | G573200 | 282825002 | 421235014 |
| 636721000000112 | Permanent atrial fibrillation | G5734 | G573400 | 440028005 | 2793259018 |
| 636701000000115 | Persistent atrial fibrillation | G5735 | G573500 | 440059007 | 2793372019 |
| 256478018 | ECG: atrial fibrillation | 3272 | 3272.00 | 164889003 | 256478018 |
| 82343012 | Atrial fibrillation | G5730 | G573000 | 49436004 | 82343012 |
| 350465014 | Non-rheumatic atrial fibrillation | G5733 | G573300 | 233911009 | 350465014 |
| 300130013 | Atrial fibrillation and flutter | G573 | G573.00 | 195080001 | 300130013 |
| 300132017 | Atrial fibrillation and flutter NOS | G573z | G573z00 | 195080001 | 497951000006115 |
| 256479014 | ECG: atrial flutter | 3273 | 3273.00 | 164890007 | 256479014 |
| 9988012 | Atrial flutter | G5731 | G573100 | 5370000 | 9988012 |

Supplementary Table S18: Read Codes for Erectile Dysfunction

| MedCode Id | Term | Original Read Code | Cleansed Read Code | Snomed CT Concept Id | Snomed CT Description Id |
| --- | --- | --- | --- | --- | --- |
| 1786154013 | Erectile dysfunction | E2273-1 | E227311 | 397803000 | 2955652011 |
| 304501018 | Impotence of organic origin | K27y1 | K27y100 | 198036002 | 304501018 |
| 1777409015 | Impotence | E2273 | E227300 | 397803000 | 1777409015 |
| 424011000006115 | [X]Psychogenic impotence | Eu522-3 | Eu52213 | 73491007 | 424011000006115 |
| 396141000006114 | [X]Male erectile disorder | Eu522-2 | Eu52212 | 397803000 | 396141000006114 |
| 295111013 | Unspecified psychosexual dysfunction | E2270 | E227000 | 268637002 | 78281000006118 |
| 369101000000114 | Treatment of erectile dysfunction NEC | 7C25E | 7C25E00 | 851081000000105 | 1550781000006111 |
| 272294012 | Revascularisation for impotence | 7A6G0 | 7A6G000 | 175881006 | 272294012 |
| 1736291000000114 | Referral to erectile dysfunction clinic declined | 8IE8 | 8IE8.00 | 704049007 | 3011784011 |
| 302271000000119 | Referral to erectile dysfunction clinic | 8HTj | 8HTj.00 | 704048004 | 3010274018 |
| 401789015 | Psychosexual dysfunction NOS | E227z | E227z00 | 268637002 | 199881000006113 |
| 401788011 | Psychosexual dysfunction | E227 | E227.00 | 268637002 | 401788011 |
| 68432015 | Psychogenic dyspareunia | E2277 | E227700 | 41021005 | 68432015 |
| 73374013 | Premature ejaculation | E2276 | E227600 | 44001008 | 73374013 |
| 489201000000110 | Operations on penis for erectile dysfunction NEC | 7C25F | 7C25F00 | 71588001 | 1588211000006116 |
| 272299019 | Ligation of penile veins for impotence | 7A6G5 | 7A6G500 | 175886001 | 272299019 |
| 370382011 | Lack of libido | E227-1 | E227.11 | 248096004 | 370382011 |
| 295112018 | Inhibited sexual desire | E2271 | E227100 | 191793009 | 295112018 |
| 135863015 | Inhibited male orgasm | E2275 | E227500 | 81903006 | 135863015 |
| 371300012 | Fear of ejaculation | E227z-1 | E227z11 | 248780009 | 371300012 |
| 2243331000000115 | Erectile dysfunction due to diabetes mellitus | K27y7 | K27y700 | 867891000000101 | 2243331000000115 |
| 1734281000000117 | Diabetic erectile dysfunction review | 66Au | 66Au.00 | 473209001 | 2956267011 |
| 1734721000000114 | Diabetic assessment of erectile dysfunction | 66Av | 66Av.00 | 473208009 | 1766911000006114 |
| 1734321000000113 | C/O erectile dysfunction | 1D1B | 1D1B.00 | 473327001 | 2242321000000110 |
| 216655018 | Advice about impotence | 67IA | 67IA.00 | 135885005 | 216655018 |

Supplementary Table S19: Read Codes for Severe Mental Illness

| MedCode Id | Term | Original Read Code | Cleansed Read Code | Snomed CT Concept Id | Snomed CT Description Id | Emis Code Category Id |
| --- | --- | --- | --- | --- | --- | --- |
| 251628010 | H/O: schizophrenia | 1464 | 1464.00 | 161468000 | 251628010 | 32 |
| 996171000006114 | Psychosis, schizophrenia and bipolar affective disorder resolved | 212T | 212T.00 | 200951000000109 | 305361000000112 | 27 |
| 492821000000113 | Schizophrenia resolved | 212W | 212W.00 | 285521000000103 | 492821000000113 | 27 |
| 460273017 | [V]Personal history of schizophrenia | ZV110 | ZV11000 | 161464003 | 251624012 | 32 |
| 251188012 | Schizophrenia association member | 13Y2 | 13Y2.00 | 161103001 | 251188012 | 17 |
| 107878010 | Paranoid schizophrenia | E103 | E103.00 | 64905009 | 107878010 | 32 |
| 294754019 | Unspecified paranoid schizophrenia | E1030 | E103000 | 64905009 | 107878010 | 32 |
| 52897013 | Chronic paranoid schizophrenia | E1032 | E103200 | 31658008 | 52897013 | 32 |
| 294757014 | Acute exacerbation of subchronic paranoid schizophrenia | E1033 | E103300 | 191554003 | 294757014 | 32 |
| 294758016 | Acute exacerbation of chronic paranoid schizophrenia | E1034 | E103400 | 191555002 | 294758016 | 32 |
| 105029017 | Paranoid schizophrenia in remission | E1035 | E103500 | 63181006 | 105029017 | 32 |
| 294760019 | Paranoid schizophrenia NOS | E103z | E103z00 | 64905009 | 107878010 | 32 |
| 418221000006118 | [X]Paranoid schizophrenia | Eu200 | Eu20000 | 64905009 | 107878010 | 32 |
| 418261000006112 | [X]Paraphrenic schizophrenia | Eu200-1 | Eu20011 | 64905009 | 1232626010 | 32 |
| 819351000006115 | Hebephrenic schizophrenia | E101 | E101.00 | 35252006 | 2881679010 | 32 |
| 294735019 | Unspecified hebephrenic schizophrenia | E1010 | E101000 | 35252006 | 2881679010 | 32 |
| 294739013 | Acute exacerbation of chronic hebephrenic schizophrenia | E1014 | E101400 | 191539009 | 294739013 | 32 |
| 294740010 | Hebephrenic schizophrenia in remission | E1015 | E101500 | 31373002 | 3425623018 | 32 |
| 294741014 | Hebephrenic schizophrenia NOS | E101z | E101z00 | 35252006 | 2881679010 | 32 |
| 388741000006110 | [X]Hebephrenic schizophrenia | Eu201 | Eu20100 | 35252006 | 2881679010 | 32 |
| 378051000006119 | [X]Disorganised schizophrenia | Eu201-1 | Eu20111 | 35252006 | 486103011 | 32 |
| 294742019 | Catatonic schizophrenia | E102 | E102.00 | 191542003 | 294742019 | 32 |
| 294744018 | Unspecified catatonic schizophrenia | E1020 | E102000 | 191542003 | 294742019 | 32 |
| 294753013 | Catatonic schizophrenia NOS | E102z | E102z00 | 191542003 | 294742019 | 32 |
| 370451000006110 | [X]Catatonic schizophrenia | Eu202 | Eu20200 | 191542003 | 294742019 | 32 |
| 370461000006112 | [X]Catatonic stupor | Eu202-1 | Eu20211 | 191542003 | 294742019 | 32 |
| 425641000006111 | [X]Schizophrenic catalepsy | Eu202-2 | Eu20212 | 191542003 | 294742019 | 32 |
| 425651000006113 | [X]Schizophrenic catatonia | Eu202-3 | Eu20213 | 191542003 | 294742019 | 32 |
| 294743012 | [X]Schizophrenic flexibilatis cerea | Eu202-4 | Eu20214 | 191542003 | 294743012 | 32 |
| 294725013 | Schizophrenic disorders | E10 | E10..00 | 191526005 | 294725013 | 32 |
| 294727017 | Simple schizophrenia | E100 | E100.00 | 191527001 | 294727017 | 32 |
| 294726014 | Schizophrenia simplex | E100-1 | E100.11 | 191527001 | 294726014 | 32 |
| 294728010 | Unspecified schizophrenia | E1000 | E100000 | 58214004 | 96745016 | 32 |
| 28758018 | Subchronic schizophrenia | E1001 | E100100 | 16990005 | 28758018 | 32 |
| 1234861017 | Chronic schizophrenic | E1002 | E100200 | 83746006 | 1234861017 | 32 |
| 294730012 | Acute exacerbation of subchronic schizophrenia | E1003 | E100300 | 111482003 | 178722014 | 32 |
| 294731011 | Acute exacerbation of chronic schizophrenia | E1004 | E100400 | 191531007 | 294731011 | 32 |
| 9225016 | Schizophrenia in remission | E1005 | E100500 | 4926007 | 9225016 | 32 |
| 294734015 | Simple schizophrenia NOS | E100z | E100z00 | 191527001 | 294727017 | 32 |
| 401763015 | Acute schizophrenic episode | E104 | E104.00 | 268617001 | 401763015 | 32 |
| 43595011 | Residual schizophrenia | E106 | E106.00 | 26025008 | 43595011 | 32 |
| 401764014 | Other schizophrenia | E10y | E10y.00 | 58214004 | 96745016 | 32 |
| 294788013 | Cenesthopathic schizophrenia | E10y-1 | E10y.11 | 191577003 | 294788013 | 32 |
| 1219653018 | Atypical schizophrenia | E10y0 | E10y000 | 111484002 | 1219653018 | 32 |
| 294789017 | Other schizophrenia NOS | E10yz | E10yz00 | 58214004 | 96745016 | 32 |
| 294790014 | Schizophrenia | E10z | E10z.00 | 58214004 | 96745016 | 32 |
| 425601000006114 | [X]Schizophrenia | Eu20 | Eu20.00 | 58214004 | 96745016 | 32 |
| 296031017 | Undifferentiated schizophrenia | Eu203 | Eu20300 | 111484002 | 178724010 | 32 |
| 366571000006114 | [X]Atypical schizophrenia | Eu203-1 | Eu20311 | 111484002 | 1219653018 | 32 |
| 424841000006113 | [X]Residual schizophrenia | Eu205 | Eu20500 | 26025008 | 43595011 | 32 |
| 371031000006115 | [X]Chronic undifferentiated schizophrenia | Eu205-1 | Eu20511 | 111484002 | 178724010 | 32 |
| 426881000006111 | [X]Simple schizophrenia | Eu206 | Eu20600 | 191527001 | 294727017 | 32 |
| 401855010 | [X]Other schizophrenia | Eu20y | Eu20y00 | 58214004 | 96745016 | 32 |
| 425681000006117 | [X]Schizophreniform disord NOS | Eu20y-2 | Eu20y12 | 58214004 | 96745016 | 32 |
| 425711000006116 | [X]Schizophrenifrm psychos NOS | Eu20y-3 | Eu20y13 | 58214004 | 96745016 | 32 |
| 296040018 | [X]Schizophrenia, unspecified | Eu20z | Eu20z00 | 58214004 | 96745016 | 32 |
| 294764011 | Latent schizophrenia | E105 | E105.00 | 191559008 | 294764011 | 32 |
| 294767016 | Chronic latent schizophrenia | E1052 | E105200 | 191562006 | 294767016 | 32 |
| 294770017 | Latent schizophrenia in remission | E1055 | E105500 | 191565008 | 294770017 | 32 |
| 155281000006119 | Schizotypal personality | E2122 | E212200 | 31027006 | 51933014 | 32 |
| 296022017 | [X]Schizophrenia, schizotypal and delusional disorders | Eu2 | Eu2..00 | 417601000000102 | 939641000000117 | 32 |
| 401856011 | Schizotypal personality disorder | Eu21 | Eu21.00 | 31027006 | 51933014 | 32 |
| 395031000006114 | [X]Latent schizophrenic reaction | Eu21-1 | Eu21.11 | 191559008 | 294764011 | 32 |
| 367951000006113 | Borderline schizophrenia | Eu21-2 | Eu21.12 | 274952002 | 410869013 | 32 |
| 395021000006111 | [X]Latent schizophrenia | Eu21-3 | Eu21.13 | 191559008 | 294764011 | 32 |
| 423271000006116 | Prepsychotic schizophrenia | Eu21-4 | Eu21.14 | 247804008 | 369984013 | 32 |
| 423471000006117 | Prodromal schizophrenia | Eu21-5 | Eu21.15 | 247804008 | 369985014 | 32 |
| 423731000006114 | [X]Pseudoneurotic schizophrenia | Eu21-6 | Eu21.16 | 31027006 | 51933014 | 32 |
| 423741000006116 | [X]Pseudopsychopathic schizophrenia | Eu21-7 | Eu21.17 | 31027006 | 51933014 | 32 |
| 425731000006110 | [X]Schizotypal personality disorder | Eu21-8 | Eu21.18 | 31027006 | 51933014 | 32 |
| 155141000006116 | Schizoaffective schizophrenia | E107 | E107.00 | 191567000 | 294772013 | 32 |
| 78531000006116 | Unspecified schizo-affective schizophrenia | E1070 | E107000 | 191567000 | 294772013 | 32 |
| 123611000006110 | Subchronic schizo-affective schizophrenia | E1071 | E107100 | 191569002 | 294775010 | 32 |
| 556631000006116 | Chronic schizoaffective schizophrenia | E1072 | E107200 | 191570001 | 294776011 | 32 |
| 456801000006115 | Acute exacerbation subchronic schizo-affective schizophrenia | E1073 | E107300 | 191571002 | 294777019 | 32 |
| 456731000006115 | Acute exacerbation of chronic schizo-affective schizophrenia | E1074 | E107400 | 191572009 | 294778012 | 32 |
| 155151000006119 | Schizoaffective schizophrenia in remission | E1075 | E107500 | 191574005 | 294780018 | 32 |
| 155161000006117 | Schizo-affective schizophrenia NOS | E107z | E107z00 | 191567000 | 294772013 | 32 |
| 425541000006110 | Schizoaffective disorder | Eu25 | Eu25.00 | 68890003 | 114425016 | 32 |
| 425511000006111 | Schizoaffective disorder, manic type | Eu250 | Eu25000 | 271428004 | 406210011 | 32 |
| 425571000006119 | [X]Schizoaffective psychosis, manic type | Eu250-1 | Eu25011 | 271428004 | 406210011 | 32 |
| 425701000006119 | [X]Schizophreniform psychosis, manic type | Eu250-2 | Eu25012 | 271428004 | 406211010 | 32 |
| 425501000006113 | Schizoaffective disorder, depressive type | Eu251 | Eu25100 | 84760002 | 140519016 | 32 |
| 425561000006114 | [X]Schizoaffective psychosis, depressive type | Eu251-1 | Eu25111 | 84760002 | 140519016 | 32 |
| 425691000006119 | [X]Schizophreniform psychosis, depressive type | Eu251-2 | Eu25112 | 84760002 | 1234980016 | 32 |
| 425521000006115 | Schizoaffective disorder, mixed type | Eu252 | Eu25200 | 270901009 | 405368015 | 32 |
| 398631000006113 | Mixed schizophrenic and affective pschosis | Eu252-2 | Eu25212 | 270901009 | 405369011 | 32 |
| 296095012 | [X]Other schizoaffective disorders | Eu25y | Eu25y00 | 68890003 | 114425016 | 32 |
| 296096013 | [X]Schizoaffective disorder, unspecified | Eu25z | Eu25z00 | 68890003 | 114425016 | 32 |
| 425551000006112 | [X]Schizoaffective psychosis NOS | Eu25z-1 | Eu25z11 | 68890003 | 114425016 | 32 |
| 1780205015 | H/O: manic depressive disorder | 146D | 146D.00 | 400998002 | 1780205015 | 32 |
| 492761000000114 | Bipolar affective disorder resolved | 212V | 212V.00 | 285491000000101 | 492761000000114 | 27 |
| 294817019 | Recurrent manic episodes, in full remission | E1116 | E111600 | 191597008 | 294817019 | 32 |
| 294876019 | Mixed bipolar affective disorder, in full remission | E1166 | E116600 | 191643001 | 294876019 | 32 |
| 294887010 | Bipolar disorder in full remission | E1176 | E117600 | 41836007 | 69794013 | 32 |
| 296130018 | Bipolar disorder in remission | Eu317 | Eu31700 | 85248005 | 141328010 | 32 |
| 345141000006111 | History of mood disorder | ZV111-1 | ZV11111 | 429124005 | 2693532011 | 32 |
| 1227584015 | [V]Personal history of manic-depressive psychosis | ZV111-2 | ZV11112 | 429124005 | 2693532011 | 32 |
| 996171000006114 | Psychosis, schizophrenia and bipolar affective disorder resolved | 212T | 212T.00 | 200951000000109 | 305361000000112 | 27 |
| 513801000006112 | Bipolar affective disorder, current episode manic | E114 | E114.00 | 191618007 | 294846014 | 32 |
| 294847017 | Manic-depressive - now manic | E114-1 | E114.11 | 191618007 | 294847017 | 32 |
| 294848010 | Bipolar affective disorder, currently manic, unspecified | E1140 | E114000 | 191618007 | 294846014 | 32 |
| 294849019 | Bipolar affective disorder, currently manic, mild | E1141 | E114100 | 191620005 | 294849019 | 32 |
| 294850019 | Bipolar affective disorder, currently manic, moderate | E1142 | E114200 | 191621009 | 294850019 | 32 |
| 513691000006116 | Severe manic bipolar I disorder without psychotic features | E1143 | E114300 | 162004 | 1352013 | 32 |
| 513701000006116 | Bipolar affect disord, currently manic,severe with psychosis | E1144 | E114400 | 191623007 | 294852010 | 32 |
| 513741000006119 | Manic bipolar I disorder in partial remission | E1145 | E114500 | 63249007 | 105141010 | 32 |
| 513811000006110 | Bipolar affective disorder, currently manic, in full remission | E1146 | E114600 | 191625000 | 294857016 | 32 |
| 294858014 | Bipolar affective disorder, currently manic, NOS | E114z | E114z00 | 191618007 | 294846014 | 32 |
| 367111000006110 | Bipolar affective disorder, current episode hypomanic | Eu310 | Eu31000 | 31446002 | 484876019 | 32 |
| 367081000006116 | [X]Bipolar affect disorder cur epi manic wout psychotic symp | Eu311 | Eu31100 | 191618007 | 294846014 | 32 |
| 367071000006119 | Bipolar affective disorder, currently manic, severe, with psychosis | Eu312 | Eu31200 | 191623007 | 294852010 | 32 |
| 513751000006117 | Bipolar affective disorder, current episode depression | E115 | E115.00 | 191627008 | 294859018 | 32 |
| 294860011 | Manic-depressive - now depressed | E115-1 | E115.11 | 191627008 | 294860011 | 32 |
| 294861010 | Bipolar affective disorder, currently depressed, unspecified | E1150 | E115000 | 191627008 | 294859018 | 32 |
| 294862015 | Bipolar affective disorder, currently depressed, mild | E1151 | E115100 | 191629006 | 294862015 | 32 |
| 294863013 | Bipolar affective disorder, currently depressed, moderate | E1152 | E115200 | 191630001 | 294863013 | 32 |
| 513731000006112 | Bipolar affect disord, now depressed, severe, no psychosis | E1153 | E115300 | 61403008 | 102016011 | 32 |
| 513721000006114 | Bipolar affect disord, now depressed, severe with psychosis | E1154 | E115400 | 765176007 | 3657334019 | 32 |
| 513711000006118 | Depressed bipolar I disorder in partial remission | E1155 | E115500 | 49512000 | 82468013 | 32 |
| 513861000006113 | Bipolar affective disorder, currently depressed, in full remission | E1156 | E115600 | 191634005 | 294867014 | 32 |
| 294868016 | Bipolar affective disorder, currently depressed, NOS | E115z | E115z00 | 191627008 | 294859018 | 32 |
| 367091000006118 | [X]Bipolar affect disorder cur epi mild or moderate depressn | Eu313 | Eu31300 | 191630001 | 294863013 | 32 |
| 367051000006112 | Severe depressed bipolar I disorder without psychotic features | Eu314 | Eu31400 | 61403008 | 102016011 | 32 |
| 367061000006114 | Psychosis and severe depression co-occurrent and due to bipolar affective disorder | Eu315 | Eu31500 | 765176007 | 3657334019 | 32 |
| 396771000006117 | [X] Manic-depressive psychosis, depressed type without psychotic symptoms | Eu332-3 | Eu33213 | 36474008 | 60885016 | 32 |
| 396781000006119 | [X]Manic-depress psychosis,depressed type+psychotic symptoms | Eu333-2 | Eu33312 | 765176007 | 3657334019 | 32 |
| 294869012 | Mixed bipolar affective disorder | E116 | E116.00 | 191636007 | 294869012 | 32 |
| 294870013 | Mixed bipolar affective disorder, unspecified | E1160 | E116000 | 191636007 | 294869012 | 32 |
| 294871012 | Mixed bipolar affective disorder, mild | E1161 | E116100 | 191638008 | 294871012 | 32 |
| 294872017 | Mixed bipolar affective disorder, moderate | E1162 | E116200 | 191639000 | 294872017 | 32 |
| 701071000006111 | Mixed bipolar affective disorder, severe | E1163 | E116300 | 764591000000108 | 1696181000000118 | 32 |
| 294874016 | Mixed bipolar affective disorder, severe, with psychosis | E1164 | E116400 | 191641004 | 294874016 | 32 |
| 701051000006118 | Mixed bipolar affective disorder, partial/unspec remission | E1165 | E116500 | 760721000000109 | 1683621000000116 | 32 |
| 294877011 | Mixed bipolar affective disorder, NOS | E116z | E116z00 | 191636007 | 294869012 | 32 |
| 294895014 | Mixed bipolar I disorder | E11y3 | E11y300 | 16506000 | 27929014 | 32 |
| 367121000006119 | [X]Bipolar affective disorder, current episode mixed | Eu316 | Eu31600 | 192362008 | 296129011 | 32 |
| 513871000006118 | Bipolar disorder | E11-1 | E11..11 | 13746004 | 23447014 | 32 |
| 294810017 | Recurrent manic episodes | E111 | E111.00 | 191590005 | 294810017 | 32 |
| 294811018 | Recurrent manic episodes, unspecified | E1110 | E111000 | 191590005 | 294810017 | 32 |
| 294812013 | Recurrent manic episodes, mild | E1111 | E111100 | 191592002 | 294812013 | 32 |
| 294813015 | Recurrent manic episodes, moderate | E1112 | E111200 | 191593007 | 294813015 | 32 |
| 182871000006115 | Recurrent manic episodes, severe | E1113 | E111300 | 764621000000106 | 1696241000000116 | 32 |
| 294815010 | Recurrent manic episodes, severe, with psychosis | E1114 | E111400 | 191595000 | 294815010 | 32 |
| 182861000006110 | Recurrent manic episodes, in partial remission | E1115 | E111500 | 764671000000105 | 1696341000000111 | 32 |
| 294818012 | Recurrent manic episode NOS | E111z | E111z00 | 191590005 | 294810017 | 32 |
| 294880012 | Unspecified bipolar affective disorder | E117 | E117.00 | 13746004 | 2839307017 | 32 |
| 294881011 | Unspecified bipolar affective disorder, unspecified | E1170 | E117000 | 13746004 | 2839307017 | 32 |
| 294882016 | Mild bipolar disorder | E1171 | E117100 | 13313007 | 22777014 | 32 |
| 294883014 | Moderate bipolar disorder | E1172 | E117200 | 79584002 | 132034014 | 32 |
| 82151000006114 | Severe bipolar disorder without psychotic features | E1173 | E117300 | 53049002 | 88241016 | 32 |
| 82171000006116 | Severe bipolar disorder with psychotic features | E1174 | E117400 | 4441000 | 7545018 | 32 |
| 82091000006116 | Bipolar disorder in partial remission | E1175 | E117500 | 5703000 | 10501015 | 32 |
| 294888017 | Unspecified bipolar affective disorder, NOS | E117z | E117z00 | 13746004 | 2839307017 | 32 |
| 294891017 | Other and unspecified manic-depressive psychoses | E11y | E11y.00 | 13746004 | 1221011015 | 32 |
| 294892012 | Unspecified manic-depressive psychoses | E11y0 | E11y000 | 13746004 | 1221011015 | 32 |
| 294893019 | Atypical manic disorder | E11y1 | E11y100 | 191658009 | 294893019 | 32 |
| 294896010 | Other and unspecified manic-depressive psychoses NOS | E11yz | E11yz00 | 13746004 | 1221011015 | 32 |
| 367101000006112 | Bipolar affective disorder | Eu31 | Eu31.00 | 13746004 | 2839307017 | 32 |
| 396791000006116 | Manic-depressive illness | Eu31-1 | Eu31.11 | 13746004 | 1221012010 | 32 |
| 396801000006115 | Manic-depressive psychosis | Eu31-2 | Eu31.12 | 13746004 | 1221011015 | 32 |
| 396071000006119 | [X]Manic-depressive reaction | Eu31-3 | Eu31.13 | 13746004 | 23447014 | 32 |
| 401865016 | [X]Other bipolar affective disorders | Eu31y | Eu31y00 | 13746004 | 23447014 | 32 |
| 367161000006113 | [X]Bipolar II disorder | Eu31y-1 | Eu31y11 | 83225003 | 138029017 | 32 |
| 424661000006111 | [X]Recurrent manic episodes | Eu31y-2 | Eu31y12 | 191590005 | 294810017 | 32 |
| 296135011 | [X]Bipolar affective disorder, unspecified | Eu31z | Eu31z00 | 13746004 | 2839307017 | 32 |
| 789221000006116 | Hypomanic psychoses | E110-1 | E110.11 | 231496004 | 346969013 | 32 |
| 389401000006111 | Hypomania | Eu300 | Eu30000 | 231496004 | 346969013 | 32 |
| 223601000000119 | Manic psychosis | E11-3 | E11..13 | 231494001 | 346966018 | 32 |
| 401765010 | Manic disorder, single episode | E110 | E110.00 | 268619003 | 401765010 | 32 |
| 294802018 | Single manic episode, unspecified | E1100 | E110000 | 268619003 | 401765010 | 32 |
| 294803011 | Single manic episode, mild | E1101 | E110100 | 191583000 | 294803011 | 32 |
| 294804017 | Single manic episode, moderate | E1102 | E110200 | 191584006 | 294804017 | 32 |
| 294805016 | Single manic episode, severe | E1103 | E110300 | 764641000000104 | 1696281000000112 | 32 |
| 294806015 | Single manic episode, severe, with psychosis | E1104 | E110400 | 191586008 | 294806015 | 32 |
| 294808019 | Single manic episode in full remission | E1106 | E110600 | 191588009 | 294808019 | 32 |
| 294809010 | Manic disorder, single episode NOS | E110z | E110z00 | 268619003 | 401765010 | 32 |
| 396741000006113 | [X]Manic episode | Eu30 | Eu30.00 | 268619003 | 401765010 | 32 |
| 296110017 | Mania | Eu301 | Eu30100 | 231494001 | 346965019 | 32 |
| 401863011 | [X]Mania with psychotic symptoms | Eu302 | Eu30200 | 231494001 | 346965019 | 32 |
| 396701000006111 | [X]Mania with mood-congruent psychotic symptoms | Eu302-1 | Eu30211 | 231494001 | 346965019 | 32 |
| 396711000006114 | [X]Mania with mood-incongruent psychotic symptoms | Eu302-2 | Eu30212 | 231494001 | 346965019 | 32 |
| 296118012 | [X]Other manic episodes | Eu30y | Eu30y00 | 231494001 | 346965019 | 32 |
| 401864017 | [X]Manic episode, unspecified | Eu30z | Eu30z00 | 268619003 | 401765010 | 32 |
| 396691000006111 | [X]Mania NOS | Eu30z-1 | Eu30z11 | 268619003 | 401765010 | 32 |
| 135278017 | Elevated mood | 1BY | 1BY..00 | 81548002 | 135278017 | 27 |
| 2157096015 | Manic mood | 1S42 | 1S42.00 | 405273008 | 2157096015 | 27 |
| MedCodeId | Term | OriginalReadCode | CleansedReadCode | SnomedCTConceptId | SnomedCTDescriptionId | EmisCodeCategoryId |
| 294725013 | Schizophrenic disorders | E10 | E10..00 | 191526005 | 294725013 | 32 |
| 367101000006112 | [X]Bipolar affective disorder | Eu31 | Eu31.00 | 13746004 | 2839307017 | 32 |
| 107878010 | Paranoid schizophrenia | E103 | E103.00 | 64905009 | 107878010 | 32 |
| 424231000006116 | [X]Psychosis NOS | Eu2z-1 | Eu2z.11 | 69322001 | 2920943016 | 32 |
| 389401000006111 | [X]Hypomania | Eu300 | Eu30000 | 231496004 | 346969013 | 32 |
| 294660013 | Senile or presenile psychoses NOS | E00z | E00z.00 | 268612007 | 401757012 | 32 |
| 294790014 | Schizophrenia NOS | E10z | E10z.00 | 58214004 | 96745016 | 32 |
| 215851000000112 | [X]Paranoid psychosis | Eu220-1 | Eu22011 | 191667009 | 3012206013 | 32 |
| 251628010 | H/O: schizophrenia | 1464 | 1464.00 | 161468000 | 251628010 | 32 |
| 396791000006116 | [X]Manic-depressive illness | Eu31-1 | Eu31.11 | 13746004 | 1221012010 | 32 |
| 425541000006110 | [X]Schizoaffective disorders | Eu25 | Eu25.00 | 68890003 | 114425016 | 32 |
| 155141000006116 | Schizo-affective schizophrenia | E107 | E107.00 | 191567000 | 294772013 | 32 |
| 294880012 | Unspecified bipolar affective disorder | E117 | E117.00 | 13746004 | 2839307017 | 32 |
| 513871000006118 | Bipolar psychoses | E11-1 | E11..11 | 13746004 | 23447014 | 32 |
| 613791000006115 | Depressive psychoses | E11-2 | E11..12 | 35489007 | 59212011 | 32 |
| 294913019 | Paranoid psychosis NOS | E12z | E12z.00 | 191667009 | 3012206013 | 32 |
| 473201000006114 | Affective psychoses | E11 | E11..00 | 46206005 | 77054013 | 32 |
| 425601000006114 | [X]Schizophrenia | Eu20 | Eu20.00 | 58214004 | 96745016 | 32 |
| 1234861017 | Chronic schizophrenic | E1002 | E100200 | 83746006 | 1234861017 | 32 |
| 401869010 | [X]Severe depressive episode with psychotic symptoms | Eu323 | Eu32300 | 191604000 | 294828015 | 32 |
| 300411000000110 | H/O: psychosis | 146H | 146H.00 | 198991000000103 | 300411000000110 | 32 |
| 44335019 | Paraphrenia | E122 | E122.00 | 26472000 | 44335019 | 32 |
| 401759010 | Other senile and presenile organic psychoses | E00y | E00y.00 | 268612007 | 401757012 | 32 |
| 401768012 | Chronic paranoid psychosis | E121 | E121.00 | 268622001 | 401768012 | 32 |
| 396741000006113 | [X]Manic episode | Eu30 | Eu30.00 | 268619003 | 401765010 | 32 |
| 396691000006111 | [X]Mania NOS | Eu30z-1 | Eu30z11 | 268619003 | 401765010 | 32 |
| 294728010 | Unspecified schizophrenia | E1000 | E100000 | 58214004 | 96745016 | 32 |
| 418221000006118 | [X]Paranoid schizophrenia | Eu200 | Eu20000 | 64905009 | 107878010 | 32 |
| 1780205015 | H/O: manic depressive disorder | 146D | 146D.00 | 400998002 | 1780205015 | 32 |
| 996171000006114 | "Psychosis, schizophrenia + bipolar affective disord resolved" | 212T | 212T.00 | 200951000000109 | 305361000000112 | 27 |
| 294724012 | Non-organic psychoses | E1 | E1...00 | 191525009 | 294724012 | 32 |
| 296022017 | "[X]Schizophrenia, schizotypal and delusional disorders" | Eu2 | Eu2..00 | 417601000000102 | 939641000000117 | 32 |
| 396801000006115 | [X]Manic-depressive psychosis | Eu31-2 | Eu31.12 | 13746004 | 1221011015 | 32 |
| 294869012 | Mixed bipolar affective disorder | E116 | E116.00 | 191636007 | 294869012 | 32 |
| 428451000006119 | [X]Symptomatic psychosis NOS | Eu0z-2 | Eu0z.12 | 280427006 | 418027011 | 32 |
| 215841000000114 | [X]Delusional disorder | Eu220 | Eu22000 | 48500005 | 80801017 | 32 |
| 789221000006116 | Hypomanic psychoses | E110-1 | E110.11 | 231496004 | 346969013 | 32 |
| 401763015 | Acute schizophrenic episode | E104 | E104.00 | 268617001 | 401763015 | 32 |
| 296110017 | [X]Mania without psychotic symptoms | Eu301 | Eu30100 | 231494001 | 346965019 | 32 |
| 294860011 | Manic-depressive - now depressed | E115-1 | E115.11 | 191627008 | 294860011 | 32 |
| 294892012 | Unspecified manic-depressive psychoses | E11y0 | E11y000 | 13746004 | 1221011015 | 32 |
| 367111000006110 | "[X]Bipolar affective disorder, current episode hypomanic" | Eu310 | Eu31000 | 31446002 | 484876019 | 32 |
| 294727017 | Simple schizophrenia | E100 | E100.00 | 191527001 | 294727017 | 32 |
| 460273017 | [V]Personal history of schizophrenia | ZV110 | ZV11000 | 161464003 | 251624012 | 32 |
| 367151000006111 | "[X]Bipolar disorder, single manic episode" | Eu30-1 | Eu30.11 | 268619003 | 401765010 | 32 |
| 401863011 | [X]Mania with psychotic symptoms | Eu302 | Eu30200 | 231494001 | 346965019 | 32 |
| 296130018 | "[X]Bipolar affective disorder, currently in remission" | Eu317 | Eu31700 | 85248005 | 141328010 | 32 |
| 223601000000119 | Manic psychoses | E11-3 | E11..13 | 231494001 | 346966018 | 32 |
| 294847017 | Manic-depressive - now manic | E114-1 | E114.11 | 191618007 | 294847017 | 32 |
| 371031000006115 | [X]Chronic undifferentiated schizophrenia | Eu205-1 | Eu20511 | 111484002 | 178724010 | 32 |
| 9225016 | Schizophrenia in remission | E1005 | E100500 | 4926007 | 9225016 | 32 |
| 419861000006117 | [X]Persistent delusional disorders | Eu22 | Eu22.00 | 231487004 | 346958010 | 32 |
| 296135011 | "[X]Bipolar affective disorder, unspecified" | Eu31z | Eu31z00 | 13746004 | 2839307017 | 32 |
| 294840015 | "Recurrent major depressive episodes, severe, with psychosis" | E1134 | E113400 | 191613003 | 294840015 | 32 |
| 1667591000000111 | [X]Nonorganic psychosis in remission | Eu26 | Eu26.00 | 755311000000100 | 1667591000000111 | 32 |
| 513751000006117 | "Bipolar affective disorder, currently depressed" | E115 | E115.00 | 191627008 | 294859018 | 32 |
| 513801000006112 | "Bipolar affective disorder, currently manic" | E114 | E114.00 | 191618007 | 294846014 | 32 |
| 425501000006113 | "[X]Schizoaffective disorder, depressive type" | Eu251 | Eu25100 | 84760002 | 140519016 | 32 |
| 296096013 | "[X]Schizoaffective disorder, unspecified" | Eu25z | Eu25z00 | 68890003 | 114425016 | 32 |
| 2157096015 | Manic mood | 1S42 | 1S42.00 | 405273008 | 2157096015 | 27 |
| 215691000006112 | Poor insight into psychotic condition | 286-1 | 286..11 | 12200008 | 21006010 | 27 |
| 25461000006115 | Other nonorganic psychoses | E13 | E13..00 | 191525009 | 294724012 | 32 |
| 367091000006118 | [X]Bipolar affect disorder cur epi mild or moderate depressn | Eu313 | Eu31300 | 191630001 | 294863013 | 32 |
| 402504012 | "Psychotic condition, insight present" | 285-1 | 285..11 | 268957000 | 402504012 | 27 |
| 401771016 | Non-organic psychosis NOS | E1z | E1z..00 | 191525009 | 2575698010 | 32 |
| 424671000006116 | [X]Recurrent severe episodes of psychotic depression | Eu333-5 | Eu33315 | 191613003 | 294840015 | 32 |
| 819351000006115 | Hebephrenic schizophrenia | E101 | E101.00 | 35252006 | 2881679010 | 32 |
| 294811018 | "Recurrent manic episodes, unspecified" | E1110 | E111000 | 191590005 | 294810017 | 32 |
| 359161000006110 | [X] Senile psychosis NOS | Eu02z-5 | Eu02z15 | 268612007 | 401757012 | 32 |
| 294760019 | Paranoid schizophrenia NOS | E103z | E103z00 | 64905009 | 107878010 | 32 |
| 412201000006113 | [X]Other nonorganic psychotic disorders | Eu2y | Eu2y.00 | 191525009 | 2575698010 | 32 |
| 294742019 | Catatonic schizophrenia | E102 | E102.00 | 191542003 | 294742019 | 32 |
| 294888017 | "Unspecified bipolar affective disorder, NOS" | E117z | E117z00 | 13746004 | 2839307017 | 32 |
| 43595011 | Residual schizophrenia | E106 | E106.00 | 26025008 | 43595011 | 32 |
| 492761000000114 | Bipolar affective disorder resolved | 212V | 212V.00 | 285491000000101 | 492761000000114 | 27 |
| 294891017 | Other and unspecified manic-depressive psychoses | E11y | E11y.00 | 13746004 | 1221011015 | 32 |
| 345141000006111 | [V]Personal history of manic-depressive psychosis | ZV111-1 | ZV11111 | 429124005 | 2693532011 | 32 |
| 294870013 | "Mixed bipolar affective disorder, unspecified" | E1160 | E116000 | 191636007 | 294869012 | 32 |
| 294810017 | Recurrent manic episodes | E111 | E111.00 | 191590005 | 294810017 | 32 |
| 52897013 | Chronic paranoid schizophrenia | E1032 | E103200 | 31658008 | 52897013 | 32 |
| 425551000006112 | [X]Schizoaffective psychosis NOS | Eu25z-1 | Eu25z11 | 68890003 | 114425016 | 32 |
| 294902017 | Other affective psychosis NOS | E11zz | E11zz00 | 441704009 | 2817266014 | 32 |
| 401856011 | [X]Schizotypal disorder | Eu21 | Eu21.00 | 31027006 | 51933014 | 32 |
| 1715191000006112 | "[X]Major depression, severe with psychotic symptoms" | Eu328 | Eu32800 | 73867007 | 122670017 | 32 |
| 155161000006117 | Schizo-affective schizophrenia NOS | E107z | E107z00 | 191567000 | 294772013 | 32 |
| 425711000006116 | [X]Schizophrenifrm psychos NOS | Eu20y-3 | Eu20y13 | 58214004 | 96745016 | 32 |
| 424071000006112 | [X]Psychogenic paranoid psychosis | Eu233-2 | Eu23312 | 191680007 | 294924017 | 32 |
| 294877011 | "Mixed bipolar affective disorder, NOS" | E116z | E116z00 | 191636007 | 294869012 | 32 |
| 362781000006116 | [X]Affective psychosis NOS | Eu3z-1 | Eu3z.11 | 46206005 | 77054013 | 32 |
| 294887010 | "Unspecified bipolar affective disorder, in full remission" | E1176 | E117600 | 41836007 | 69794013 | 32 |
| 425521000006115 | "[X]Schizoaffective disorder, mixed type" | Eu252 | Eu25200 | 270901009 | 405368015 | 32 |
| 396771000006117 | "[X]Manic-depress psychosis,depressd,no psychotic symptoms" | Eu332-3 | Eu33213 | 36474008 | 60885016 | 32 |
| 425561000006114 | "[X]Schizoaffective psychosis, depressive type" | Eu251-1 | Eu25111 | 84760002 | 140519016 | 32 |
| 396781000006119 | "[X]Manic-depress psychosis,depressed type+psychotic symptoms" | Eu333-2 | Eu33312 | 765176007 | 3657334019 | 32 |
| 1227584015 | [V]Personal history of manic-depressive psychosis | ZV111-2 | ZV11112 | 429124005 | 2693532011 | 32 |
| 296040018 | "[X]Schizophrenia, unspecified" | Eu20z | Eu20z00 | 58214004 | 96745016 | 32 |
| 401864017 | "[X]Manic episode, unspecified" | Eu30z | Eu30z00 | 268619003 | 401765010 | 32 |
| 105029017 | Paranoid schizophrenia in remission | E1035 | E103500 | 63181006 | 105029017 | 32 |
| 398631000006113 | [X]Mixed schizophrenic and affective psychosis | Eu252-2 | Eu25212 | 270901009 | 405369011 | 32 |
| 294897018 | Other and unspecified affective psychoses | E11z | E11z.00 | 441704009 | 2817266014 | 32 |
| 367071000006119 | [X]Bipolar affect disorder cur epi manic with psychotic symp | Eu312 | Eu31200 | 191623007 | 294852010 | 32 |
| 425511000006111 | "[X]Schizoaffective disorder, manic type" | Eu250 | Eu25000 | 271428004 | 406210011 | 32 |
| 370981000006112 | [X]Chronic hallucinatory psychosis | Eu2y-1 | Eu2y.11 | 480111000000107 | 885251000000119 | 32 |
| 367121000006119 | "[X]Bipolar affective disorder, current episode mixed" | Eu316 | Eu31600 | 192362008 | 296129011 | 32 |
| 294818012 | Recurrent manic episode NOS | E111z | E111z00 | 191590005 | 294810017 | 32 |
| 367061000006114 | [X]Bipolar affect dis cur epi severe depres with psyc symp | Eu315 | Eu31500 | 765176007 | 3657334019 | 32 |
| 155151000006119 | Schizo-affective schizophrenia in remission | E1075 | E107500 | 191574005 | 294780018 | 32 |
| 1219653018 | Atypical schizophrenia | E10y0 | E10y000 | 111484002 | 1219653018 | 32 |
| 296066015 | "[X]Persistent delusional disorder, unspecified" | Eu22z | Eu22z00 | 231487004 | 346958010 | 32 |
| 294754019 | Unspecified paranoid schizophrenia | E1030 | E103000 | 64905009 | 107878010 | 32 |
| 424661000006111 | [X]Recurrent manic episodes | Eu31y-2 | Eu31y12 | 191590005 | 294810017 | 32 |
| 401865016 | [X]Other bipolar affective disorders | Eu31y | Eu31y00 | 13746004 | 23447014 | 32 |
| 1785861000006112 | [X]Bipolar affective disorder type II | Eu319 | Eu31900 | 83225003 | 138029017 | 32 |
| 389451000006110 | [X]Hysterical psychosis | Eu44-4 | Eu44.14 | 44376007 | 74016017 | 32 |
| 294758016 | Acute exacerbation of chronic paranoid schizophrenia | E1034 | E103400 | 191555002 | 294758016 | 32 |
| 401764014 | Other schizophrenia | E10y | E10y.00 | 58214004 | 96745016 | 32 |
| 294898011 | Unspecified affective psychoses NOS | E11z0 | E11z000 | 441704009 | 2817266014 | 32 |
| 424681000006118 | [X]Recurrent severe episodes/reactive depressive psychosis | Eu333-6 | Eu33316 | 1086471000000103 | 2721771000000114 | 32 |
| 78531000006116 | Unspecified schizo-affective schizophrenia | E1070 | E107000 | 191567000 | 294772013 | 32 |
| 294868016 | "Bipolar affective disorder, currently depressed, NOS" | E115z | E115z00 | 191627008 | 294859018 | 32 |
| 492821000000113 | Schizophrenia resolved | 212W | 212W.00 | 285521000000103 | 492821000000113 | 27 |
| 294924017 | Psychogenic paranoid psychosis | E134 | E134.00 | 191680007 | 294924017 | 32 |
| 294881011 | "Unspecified bipolar affective disorder, unspecified" | E1170 | E117000 | 13746004 | 2839307017 | 32 |
| 294862015 | "Bipolar affective disorder, currently depressed, mild" | E1151 | E115100 | 191629006 | 294862015 | 32 |
| 556631000006116 | Chronic schizo-affective schizophrenia | E1072 | E107200 | 191570001 | 294776011 | 32 |
| 425571000006119 | "[X]Schizoaffective psychosis, manic type" | Eu250-1 | Eu25011 | 271428004 | 406210011 | 32 |
| 367081000006116 | [X]Bipolar affect disorder cur epi manic wout psychotic symp | Eu311 | Eu31100 | 191618007 | 294846014 | 32 |
| 294817019 | "Recurrent manic episodes, in full remission" | E1116 | E111600 | 191597008 | 294817019 | 32 |
| 294848010 | "Bipolar affective disorder, currently manic, unspecified" | E1140 | E114000 | 191618007 | 294846014 | 32 |
| 294861010 | "Bipolar affective disorder, currently depressed, unspecified" | E1150 | E115000 | 191627008 | 294859018 | 32 |
| 425651000006113 | [X]Schizophrenic catatonia | Eu202-3 | Eu20213 | 191542003 | 294742019 | 32 |
| 294731011 | Acute exacerbation of chronic schizophrenia | E1004 | E100400 | 191531007 | 294731011 | 32 |
| 423041000006119 | [X]Post-schizophrenic depression | Eu204 | Eu20400 | 231485007 | 346956014 | 32 |
| 294789017 | Other schizophrenia NOS | E10yz | E10yz00 | 58214004 | 96745016 | 32 |
| 359091000006115 | [X] Presenile psychosis NOS | Eu02z-2 | Eu02z12 | 268612007 | 401757012 | 32 |
| 294863013 | "Bipolar affective disorder, currently depressed, moderate" | E1152 | E115200 | 191630001 | 294863013 | 32 |
| 294874016 | "Mixed bipolar affective disorder, severe, with psychosis" | E1164 | E116400 | 191641004 | 294874016 | 32 |
| 294876019 | "Mixed bipolar affective disorder, in full remission" | E1166 | E116600 | 191643001 | 294876019 | 32 |
| 296031017 | [X]Undifferentiated schizophrenia | Eu203 | Eu20300 | 111484002 | 178724010 | 32 |
| 376271000006119 | [X]Cycloid psychosis | Eu230-2 | Eu23012 | 307417003 | 450678015 | 32 |
| 294849019 | "Bipolar affective disorder, currently manic, mild" | E1141 | E114100 | 191620005 | 294849019 | 32 |
| 425691000006119 | "[X]Schizophreniform psychosis, depressive type" | Eu251-2 | Eu25112 | 84760002 | 1234980016 | 32 |
| 418251000006110 | [X]Paraphrenia - late | Eu220-3 | Eu22013 | 38295006 | 1229409019 | 32 |
| 367161000006113 | [X]Bipolar II disorder | Eu31y-1 | Eu31y11 | 83225003 | 138029017 | 32 |
| 424551000006113 | [X]Recurr severe episodes/psychogenic depressive psychosis | Eu333-4 | Eu33314 | 191613003 | 294840015 | 32 |
| 294949018 | Other specified non-organic psychoses | E1y | E1y..00 | 191525009 | 294724012 | 32 |
| 294850019 | "Bipolar affective disorder, currently manic, moderate" | E1142 | E114200 | 191621009 | 294850019 | 32 |
| 294858014 | "Bipolar affective disorder, currently manic, NOS" | E114z | E114z00 | 191618007 | 294846014 | 32 |
| 425701000006119 | "[X]Schizophreniform psychosis, manic type" | Eu250-2 | Eu25012 | 271428004 | 406211010 | 32 |
| 513861000006113 | "Bipolar affective disorder, now depressed, in full remission" | E1156 | E115600 | 191634005 | 294867014 | 32 |
| 396701000006111 | [X]Mania with mood-congruent psychotic symptoms | Eu302-1 | Eu30211 | 231494001 | 346965019 | 32 |
| 914461000006118 | [X]Capgras syndrome | Eu221-1 | Eu22111 | 44906001 | 1230158013 | 32 |
| 296118012 | [X]Other manic episodes | Eu30y | Eu30y00 | 231494001 | 346965019 | 32 |
| 401855010 | [X]Other schizophrenia | Eu20y | Eu20y00 | 58214004 | 96745016 | 32 |
| 513701000006116 | "Bipolar affect disord, currently manic,severe with psychosis" | E1144 | E114400 | 191623007 | 294852010 | 32 |
| 296095012 | [X]Other schizoaffective disorders | Eu25y | Eu25y00 | 68890003 | 114425016 | 32 |
| 388741000006110 | [X]Hebephrenic schizophrenia | Eu201 | Eu20100 | 35252006 | 2881679010 | 32 |
| 294815010 | "Recurrent manic episodes, severe, with psychosis" | E1114 | E111400 | 191595000 | 294815010 | 32 |
| 294896010 | Other and unspecified manic-depressive psychoses NOS | E11yz | E11yz00 | 13746004 | 1221011015 | 32 |
| 294813015 | "Recurrent manic episodes, moderate" | E1112 | E111200 | 191593007 | 294813015 | 32 |
| 424841000006113 | [X]Residual schizophrenia | Eu205 | Eu20500 | 26025008 | 43595011 | 32 |
| 294872017 | "Mixed bipolar affective disorder, moderate" | E1162 | E116200 | 191639000 | 294872017 | 32 |
| 513731000006112 | "Bipolar affect disord, now depressed, severe, no psychosis" | E1153 | E115300 | 61403008 | 102016011 | 32 |
| 513811000006110 | "Bipolar affective disorder, currently manic, full remission" | E1146 | E114600 | 191625000 | 294857016 | 32 |
| 294871012 | "Mixed bipolar affective disorder, mild" | E1161 | E116100 | 191638008 | 294871012 | 32 |
| 513691000006116 | "Bipolar affect disord, currently manic, severe, no psychosis" | E1143 | E114300 | 162004 | 1352013 | 32 |
| 294812013 | "Recurrent manic episodes, mild" | E1111 | E111100 | 191592002 | 294812013 | 32 |
| 513721000006114 | "Bipolar affect disord, now depressed, severe with psychosis" | E1154 | E115400 | 765176007 | 3657334019 | 32 |
| 425681000006117 | [X]Schizophreniform disord NOS | Eu20y-2 | Eu20y12 | 58214004 | 96745016 | 32 |
| 294933015 | Psychoses with origin in childhood | E14 | E14..00 | 191687005 | 294933015 | 32 |
| 294757014 | Acute exacerbation of subchronic paranoid schizophrenia | E1033 | E103300 | 191554003 | 294757014 | 32 |
| 370451000006110 | [X]Catatonic schizophrenia | Eu202 | Eu20200 | 191542003 | 294742019 | 32 |
| 456731000006115 | Acute exacerbation of chronic schizo-affective schizophrenia | E1074 | E107400 | 191572009 | 294778012 | 32 |
| 182871000006115 | "Recurrent manic episodes, severe without mention psychosis" | E1113 | E111300 | 764621000000106 | 1696241000000116 | 32 |
| 82171000006116 | "Unspecified bipolar affective disorder,severe with psychosis" | E1174 | E117400 | 4441000 | 7545018 | 32 |
| 294883014 | "Unspecified bipolar affective disorder, moderate" | E1172 | E117200 | 79584002 | 132034014 | 32 |
| 396711000006114 | [X]Mania with mood-incongruent psychotic symptoms | Eu302-2 | Eu30212 | 231494001 | 346965019 | 32 |
| 396071000006119 | [X]Manic-depressive reaction | Eu31-3 | Eu31.13 | 13746004 | 23447014 | 32 |
| 376281000006116 | [X]Cycloid psychosis with symptoms of schizophrenia | Eu231-2 | Eu23112 | 307417003 | 450678015 | 32 |
| 294730012 | Acute exacerbation of subchronic schizophrenia | E1003 | E100300 | 111482003 | 178722014 | 32 |
| 294882016 | "Unspecified bipolar affective disorder, mild" | E1171 | E117100 | 13313007 | 22777014 | 32 |
| 401857019 | [X]Other persistent delusional disorders | Eu22y | Eu22y00 | 231487004 | 346958010 | 32 |
| 418261000006112 | [X]Paraphrenic schizophrenia | Eu200-1 | Eu20011 | 64905009 | 1232626010 | 32 |
| 119579013 | Disintegrative psychosis | E141 | E141.00 | 71961003 | 119579013 | 32 |
| 378051000006119 | [X]Disorganised schizophrenia | Eu201-1 | Eu20111 | 35252006 | 486103011 | 32 |
| 423731000006114 | [X]Pseudoneurotic schizophrenia | Eu21-6 | Eu21.16 | 31027006 | 51933014 | 32 |
| 701071000006111 | "Mixed bipolar affective disorder, severe, without psychosis" | E1163 | E116300 | 764591000000108 | 1696181000000118 | 32 |
| 701051000006118 | "Mixed bipolar affective disorder, partial/unspec remission" | E1165 | E116500 | 760721000000109 | 1683621000000116 | 32 |
| 425671000006115 | [X]Schizophrenic reaction | Eu232-4 | Eu23214 | 278853003 | 415877014 | 32 |
| 1785871000006117 | [X]Bipolar II disorder | Eu319-1 | Eu31911 | 83225003 | 138029017 | 32 |
| 28758018 | Subchronic schizophrenia | E1001 | E100100 | 16990005 | 28758018 | 32 |
| 914471000006113 | [X]Delusional misidentification syndrome | Eu221 | Eu22100 | 44906001 | 1230157015 | 32 |
| 294753013 | Catatonic schizophrenia NOS | E102z | E102z00 | 191542003 | 294742019 | 32 |
| 82091000006116 | "Unspecified bipolar affect disord, partial/unspec remission" | E1175 | E117500 | 5703000 | 10501015 | 32 |
| 294741014 | Hebephrenic schizophrenia NOS | E101z | E101z00 | 35252006 | 2881679010 | 32 |
| 513741000006119 | "Bipolar affect disord,currently manic, part/unspec remission" | E1145 | E114500 | 63249007 | 105141010 | 32 |
| 123611000006110 | Subchronic schizo-affective schizophrenia | E1071 | E107100 | 191569002 | 294775010 | 32 |
| 456801000006115 | Acute exacerbation subchronic schizo-affective schizophrenia | E1073 | E107300 | 191571002 | 294777019 | 32 |
| 425641000006111 | [X]Schizophrenic catalepsy | Eu202-2 | Eu20212 | 191542003 | 294742019 | 32 |
| 294895014 | Other mixed manic-depressive psychoses | E11y3 | E11y300 | 16506000 | 27929014 | 32 |
| 513711000006118 | "Bipolar affect disord, now depressed, part/unspec remission" | E1155 | E115500 | 49512000 | 82468013 | 32 |
| 294726014 | Schizophrenia simplex | E100-1 | E100.11 | 191527001 | 294726014 | 32 |
| 366571000006114 | [X]Atypical schizophrenia | Eu203-1 | Eu20311 | 111484002 | 1219653018 | 32 |
| 178723016 | Catatonic schizophrenia in remission | E1025 | E102500 | 111483008 | 178723016 | 32 |
| 1785851000006110 | [X]Bipolar affective disorder type I | Eu318 | Eu31800 | 371596008 | 1210225015 | 32 |
| 294740010 | Hebephrenic schizophrenia in remission | E1015 | E101500 | 31373002 | 3425623018 | 32 |
| 294788013 | Cenesthopathic schizophrenia | E10y-1 | E10y.11 | 191577003 | 294788013 | 32 |
| 967791000006111 | [X]Cotard syndrome | Eu222 | Eu22200 | 357705009 | 2840462017 | 32 |
| 294743012 | [X]Schizophrenic flexibilatis cerea | Eu202-4 | Eu20214 | 191542003 | 294743012 | 32 |
| 346897015 | Presbyophrenic psychosis | E00y-1 | E00y.11 | 231438001 | 346897015 | 32 |
| 294744018 | Unspecified catatonic schizophrenia | E1020 | E102000 | 191542003 | 294742019 | 32 |
| 182861000006110 | "Recurrent manic episodes, partial or unspecified remission" | E1115 | E111500 | 764671000000105 | 1696341000000111 | 32 |
| 377461000006113 | [X]Disintegrative psychosis | Eu843-2 | Eu84312 | 35919005 | 59939011 | 32 |
| 294735019 | Unspecified hebephrenic schizophrenia | E1010 | E101000 | 35252006 | 2881679010 | 32 |
| 294893019 | Atypical manic disorder | E11y1 | E11y100 | 191658009 | 294893019 | 32 |
| 132503015 | Subchronic paranoid schizophrenia | E1031 | E103100 | 79866005 | 132503015 | 32 |
| 294939016 | Residual disintegrative psychoses | E1411 | E141100 | 191693002 | 294939016 | 32 |
| 423741000006116 | [X]Pseudopsychopathic schizophrenia | Eu21-7 | Eu21.17 | 31027006 | 51933014 | 32 |
| 82151000006114 | "Unspecified bipolar affective disorder, severe, no psychosis" | E1173 | E117300 | 53049002 | 88241016 | 32 |
| 294739013 | Acute exacerbation of chronic hebephrenic schizophrenia | E1014 | E101400 | 191539009 | 294739013 | 32 |
| 294773015 | Cyclic schizophrenia | E107-1 | E107.11 | 191567000 | 294773015 | 32 |
| 294787015 | Coenesthopathic schizophrenia | E10y1 | E10y100 | 191577003 | 294787015 | 32 |
| 71539017 | Subchronic catatonic schizophrenia | E1021 | E102100 | 42868002 | 71539017 | 32 |
| 376251000006112 | [X]Cyclic schizophrenia | Eu252-1 | Eu25211 | 270901009 | 405368015 | 32 |
| 294751010 | Acute exacerbation of chronic catatonic schizophrenia | E1024 | E102400 | 191548004 | 294751010 | 32 |

Supplementary Table S20: Read Codes for Diabetes

| MedCode Id | Term | Original Read Code | Cleansed Read Code | Snomed CT Concept Id | Snomed CT Description Id |
| --- | --- | --- | --- | --- | --- |
| 84281000006115 | Type 1 diabetes mellitus | C108-2 | C108.12 | 46635009 | 84281000006115 |
| 84651000006114 | Type I diabetes mellitus | C108-3 | C108.13 | 46635009 | 84651000006114 |
| 84801000006111 | Type I diabetes mellitus with renal complications | C1080-1 | C108011 | 421893009 | 84801000006111 |
| 84431000006117 | Type 1 diabetes mellitus with renal complications | C1080-2 | C108012 | 421893009 | 84431000006117 |
| 84751000006116 | Type I diabetes mellitus with neurological complications | C1082-1 | C108211 | 421468001 | 84751000006116 |
| 84381000006113 | Type 1 diabetes mellitus with neurological complications | C1082-2 | C108212 | 421468001 | 84381000006113 |
| 72721000006113 | Unstable type I diabetes mellitus | C1084-1 | C108411 | 290002008 | 72721000006113 |
| 72711000006117 | Unstable type 1 diabetes mellitus | C1084-2 | C108412 | 290002008 | 72711000006117 |
| 84821000006118 | Type I diabetes mellitus with ulcer | C1085-1 | C108511 | 190368000 | 84821000006118 |
| 84451000006112 | Type 1 diabetes mellitus with ulcer | C1085-2 | C108512 | 190368000 | 84451000006112 |
| 84811000006114 | Type I diabetes mellitus with retinopathy | C1087-1 | C108711 | 420789003 | 84811000006114 |
| 84441000006110 | Type 1 diabetes mellitus with retinopathy | C1087-2 | C108712 | 420789003 | 84441000006110 |
| 84661000006111 | Type I diabetes mellitus - poor control | C1088-1 | C108811 | 444073006 | 84661000006111 |
| 84291000006117 | Type 1 diabetes mellitus - poor control | C1088-2 | C108812 | 444073006 | 84291000006117 |
| 84671000006116 | Type I diabetes mellitus maturity onset | C1089-1 | C108911 | 190372001 | 84671000006116 |
| 84301000006116 | Type 1 diabetes mellitus maturity onset | C1089-2 | C108912 | 190372001 | 84301000006116 |
| 84831000006115 | Type I diabetes mellitus without complication | C108A-1 | C108A11 | 313435000 | 84831000006115 |
| 84741000006118 | Type I diabetes mellitus with nephropathy | C108D-1 | C108D11 | 421893009 | 84741000006118 |
| 84711000006117 | Type I diabetes mellitus with hypoglycaemic coma | C108E-1 | C108E11 | 314771006 | 84711000006117 |
| 84341000006119 | Type 1 diabetes mellitus with hypoglycaemic coma | C108E-2 | C108E12 | 314771006 | 84341000006119 |
| 84691000006115 | Type I diabetes mellitus with diabetic cataract | C108F-1 | C108F11 | 421920002 | 84691000006115 |
| 84681000006118 | Type I diabetes mellitus with arthropathy | C108H-1 | C108H11 | 314893005 | 84681000006118 |
| 84761000006119 | Type I diabetes mellitus with neuropathic arthropathy | C108J-1 | C108J11 | 71771000119100 | 84761000006119 |
| 84391000006111 | Type 1 diabetes mellitus with neuropathic arthropathy | C108J-2 | C108J12 | 71771000119100 | 84391000006111 |
| 197984010 | Type 1 diabetes mellitus | C10E | C10E.00 | 46635009 | 197984010 |
| 494564012 | Type I diabetes mellitus | C10E-1 | C10E.11 | 46635009 | 494564012 |
| 913451000006117 | Type 1 diabetes mellitus with renal complications | C10E0 | C10E000 | 421893009 | 913451000006117 |
| 913481000006113 | Type 1 diabetes mellitus with ophthalmic complications | C10E1 | C10E100 | 421165007 | 913481000006113 |
| 913511000006117 | Type 1 diabetes mellitus with neurological complications | C10E2 | C10E200 | 421468001 | 913511000006117 |
| 913541000006118 | Type 1 diabetes mellitus with multiple complications | C10E3 | C10E300 | 422228004 | 913541000006118 |
| 913551000006116 | Type I diabetes mellitus with multiple complications | C10E3-1 | C10E311 | 422228004 | 913551000006116 |
| 429971019 | Unstable type 1 diabetes mellitus | C10E4 | C10E400 | 290002008 | 429971019 |
| 429970018 | Unstable type I diabetes mellitus | C10E4-1 | C10E411 | 290002008 | 429970018 |
| 292540012 | Type 1 diabetes mellitus with ulcer | C10E5 | C10E500 | 190368000 | 292540012 |
| 292538019 | Type I diabetes mellitus with ulcer | C10E5-1 | C10E511 | 190368000 | 292538019 |
| 292541011 | Type 1 diabetes mellitus with gangrene | C10E6 | C10E600 | 190369008 | 292541011 |
| 913661000006118 | Type 1 diabetes mellitus with retinopathy | C10E7 | C10E700 | 420789003 | 913661000006118 |
| 913671000006113 | Type I diabetes mellitus with retinopathy | C10E7-1 | C10E711 | 420789003 | 913671000006113 |
| 292548017 | Type 1 diabetes mellitus - poor control | C10E8 | C10E800 | 444073006 | 913691000006114 |
| 292553010 | Type 1 diabetes mellitus maturity onset | C10E9 | C10E900 | 190372001 | 292553010 |
| 292551012 | Type I diabetes mellitus maturity onset | C10E9-1 | C10E911 | 190372001 | 292551012 |
| 457326014 | Type 1 diabetes mellitus without complication | C10EA | C10EA00 | 313435000 | 457326014 |
| 457325013 | Type I diabetes mellitus without complication | C10EA-1 | C10EA11 | 313435000 | 457325013 |
| 913781000006117 | Type 1 diabetes mellitus with mononeuropathy | C10EB | C10EB00 | 420918009 | 913781000006117 |
| 913811000006115 | Type 1 diabetes mellitus with polyneuropathy | C10EC | C10EC00 | 713705003 | 913811000006115 |
| 913821000006111 | Type I diabetes mellitus with polyneuropathy | C10EC-1 | C10EC11 | 713705003 | 913821000006111 |
| 913841000006116 | Type 1 diabetes mellitus with nephropathy | C10ED | C10ED00 | 421893009 | 913841000006116 |
| 459161015 | Type 1 diabetes mellitus with hypoglycaemic coma | C10EE | C10EE00 | 314771006 | 459161015 |
| 913901000006112 | Type 1 diabetes mellitus with diabetic cataract | C10EF | C10EF00 | 421920002 | 913901000006112 |
| 913931000006116 | Type 1 diabetes mellitus with peripheral angiopathy | C10EG | C10EG00 | 31211000119101 | 913931000006116 |
| 459292011 | Type 1 diabetes mellitus with arthropathy | C10EH | C10EH00 | 314893005 | 459292011 |
| 459296014 | Type 1 diabetes mellitus with neuropathic arthropathy | C10EJ | C10EJ00 | 71771000119100 | 3010513018 |
| 928461000006119 | Type 1 diabetes mellitus with persistent proteinuria | C10EK | C10EK00 | 420514000 | 928461000006119 |
| 1780311019 | Type 1 diabetes mellitus with persistent microalbuminuria | C10EL | C10EL00 | 401110002 | 1780311019 |
| 928501000006119 | Type 1 diabetes mellitus with ketoacidosis | C10EM | C10EM00 | 420270002 | 928501000006119 |
| 928511000006116 | Type I diabetes mellitus with ketoacidosis | C10EM-1 | C10EM11 | 420270002 | 928511000006116 |
| 928521000006112 | Type 1 diabetes mellitus with ketoacidotic coma | C10EN | C10EN00 | 421075007 | 928521000006112 |
| 928531000006110 | Type I diabetes mellitus with ketoacidotic coma | C10EN-1 | C10EN11 | 421075007 | 928531000006110 |
| 938301000006114 | Type 1 diabetes mellitus with exudative maculopathy | C10EP | C10EP00 | 420486006 | 938301000006114 |
| 938311000006112 | Type I diabetes mellitus with exudative maculopathy | C10EP-1 | C10EP11 | 420486006 | 938311000006112 |
| 299601000000114 | Type 1 diabetes mellitus with gastroparesis | C10EQ | C10EQ00 | 713702000 | 1001361000006116 |
| 84471000006119 | Type 2 diabetes mellitus | C109-2 | C109.12 | 44054006 | 84471000006119 |
| 84841000006113 | Type II diabetes mellitus | C109-3 | C109.13 | 44054006 | 84841000006113 |
| 84981000006114 | Type II diabetes mellitus with renal complications | C1090-1 | C109011 | 420279001 | 84981000006114 |
| 84611000006113 | Type 2 diabetes mellitus with renal complications | C1090-2 | C109012 | 420279001 | 84611000006113 |
| 84951000006118 | Type II diabetes mellitus with ophthalmic complications | C1091-1 | C109111 | 422099009 | 84951000006118 |
| 84581000006117 | Type 2 diabetes mellitus with ophthalmic complications | C1091-2 | C109112 | 422099009 | 84581000006117 |
| 84931000006113 | Type II diabetes mellitus with neurological complications | C1092-1 | C109211 | 421326000 | 84931000006113 |
| 84561000006110 | Type 2 diabetes mellitus with neurological complications | C1092-2 | C109212 | 421326000 | 84561000006110 |
| 85001000006117 | Type II diabetes mellitus with ulcer | C1094-1 | C109411 | 190389009 | 85001000006117 |
| 84631000006119 | Type 2 diabetes mellitus with ulcer | C1094-2 | C109412 | 190389009 | 84631000006119 |
| 84881000006119 | Type II diabetes mellitus with gangrene | C1095-1 | C109511 | 190390000 | 84881000006119 |
| 84511000006112 | Type 2 diabetes mellitus with gangrene | C1095-2 | C109512 | 190390000 | 84511000006112 |
| 84991000006112 | Type II diabetes mellitus with retinopathy | C1096-1 | C109611 | 422034002 | 84991000006112 |
| 84621000006117 | Type 2 diabetes mellitus with retinopathy | C1096-2 | C109612 | 422034002 | 84621000006117 |
| 84851000006110 | Type II diabetes mellitus - poor control | C1097-1 | C109711 | 443694000 | 84851000006110 |
| 84481000006116 | Type 2 diabetes mellitus - poor control | C1097-2 | C109712 | 443694000 | 84481000006116 |
| 84901000006117 | Type II diabetes mellitus with mononeuropathy | C109A-1 | C109A11 | 420436000 | 84901000006117 |
| 84971000006111 | Type II diabetes mellitus with polyneuropathy | C109B-1 | C109B11 | 713706002 | 84971000006111 |
| 84921000006110 | Type II diabetes mellitus with nephropathy | C109C-1 | C109C11 | 420279001 | 84921000006110 |
| 84551000006113 | Type 2 diabetes mellitus with nephropathy | C109C-2 | C109C12 | 420279001 | 84551000006113 |
| 84891000006116 | Type II diabetes mellitus with hypoglycaemic coma | C109D-1 | C109D11 | 719216001 | 84891000006116 |
| 84521000006116 | Type 2 diabetes mellitus with hypoglycaemic coma | C109D-2 | C109D12 | 719216001 | 84521000006116 |
| 84871000006117 | Type II diabetes mellitus with diabetic cataract | C109E-1 | C109E11 | 420756003 | 84871000006117 |
| 84501000006114 | Type 2 diabetes mellitus with diabetic cataract | C109E-2 | C109E12 | 420756003 | 84501000006114 |
| 84961000006116 | Type II diabetes mellitus with peripheral angiopathy | C109F-1 | C109F11 | 314902007 | 84961000006116 |
| 84591000006119 | Type 2 diabetes mellitus with peripheral angiopathy | C109F-2 | C109F12 | 314902007 | 84591000006119 |
| 84861000006112 | Type II diabetes mellitus with arthropathy | C109G-1 | C109G11 | 314903002 | 84861000006112 |
| 84491000006118 | Type 2 diabetes mellitus with arthropathy | C109G-2 | C109G12 | 314903002 | 84491000006118 |
| 84941000006115 | Type II diabetes mellitus with neuropathic arthropathy | C109H-1 | C109H11 | 314904008 | 84941000006115 |
| 84571000006115 | Type 2 diabetes mellitus with neuropathic arthropathy | C109H-2 | C109H12 | 314904008 | 84571000006115 |
| 840951000006119 | Insulin treated Type 2 diabetes mellitus | C109J | C109J00 | 237599002 | 840951000006119 |
| 841351000006110 | Insulin treated Type II diabetes mellitus | C109J-2 | C109J12 | 237599002 | 841351000006110 |
| 850691000006118 | Hyperosmolar non-ketotic state in type 2 diabetes mellitus | C109K | C109K00 | 395204000 | 850691000006118 |
| 197761014 | Type 2 diabetes mellitus | C10F | C10F.00 | 44054006 | 197761014 |
| 493774016 | Type II diabetes mellitus | C10F-1 | C10F.11 | 44054006 | 493774016 |
| 914031000006118 | Type 2 diabetes mellitus with renal complications | C10F0 | C10F000 | 420279001 | 914031000006118 |
| 914041000006111 | Type II diabetes mellitus with renal complications | C10F0-1 | C10F011 | 420279001 | 914041000006111 |
| 914051000006113 | Type 2 diabetes mellitus with ophthalmic complications | C10F1 | C10F100 | 422099009 | 914051000006113 |
| 914071000006115 | Type 2 diabetes mellitus with neurological complications | C10F2 | C10F200 | 421326000 | 914071000006115 |
| 914081000006117 | Type II diabetes mellitus with neurological complications | C10F2-1 | C10F211 | 421326000 | 914081000006117 |
| 292577016 | Type 2 diabetes mellitus with multiple complications | C10F3 | C10F300 | 190388001 | 292577016 |
| 292576013 | Type II diabetes mellitus with multiple complications | C10F3-1 | C10F311 | 190388001 | 292576013 |
| 292579018 | Type 2 diabetes mellitus with ulcer | C10F4 | C10F400 | 190389009 | 292579018 |
| 292581016 | Type II diabetes mellitus with ulcer | C10F4-1 | C10F411 | 190389009 | 292581016 |
| 292583018 | Type 2 diabetes mellitus with gangrene | C10F5 | C10F500 | 190390000 | 292583018 |
| 914151000006112 | Type 2 diabetes mellitus with retinopathy | C10F6 | C10F600 | 422034002 | 914151000006112 |
| 914161000006114 | Type II diabetes mellitus with retinopathy | C10F6-1 | C10F611 | 422034002 | 914161000006114 |
| 292590011 | Type 2 diabetes mellitus - poor control | C10F7 | C10F700 | 443694000 | 914171000006119 |
| 292589019 | Type II diabetes mellitus - poor control | C10F7-1 | C10F711 | 443694000 | 914181000006116 |
| 457329019 | Type 2 diabetes mellitus without complication | C10F9 | C10F900 | 313436004 | 457329019 |
| 457330012 | Type II diabetes mellitus without complication | C10F9-1 | C10F911 | 313436004 | 457330012 |
| 914221000006113 | Type 2 diabetes mellitus with mononeuropathy | C10FA | C10FA00 | 420436000 | 914221000006113 |
| 914231000006111 | Type II diabetes mellitus with mononeuropathy | C10FA-1 | C10FA11 | 420436000 | 914231000006111 |
| 914241000006118 | Type 2 diabetes mellitus with polyneuropathy | C10FB | C10FB00 | 713706002 | 914241000006118 |
| 914251000006116 | Type II diabetes mellitus with polyneuropathy | C10FB-1 | C10FB11 | 713706002 | 914251000006116 |
| 914261000006119 | Type 2 diabetes mellitus with nephropathy | C10FC | C10FC00 | 420279001 | 914261000006119 |
| 459167016 | Type 2 diabetes mellitus with hypoglycaemic coma | C10FD | C10FD00 | 719216001 | 914281000006112 |
| 459169018 | Type II diabetes mellitus with hypoglycaemic coma | C10FD-1 | C10FD11 | 719216001 | 914291000006110 |
| 914301000006111 | Type 2 diabetes mellitus with diabetic cataract | C10FE | C10FE00 | 420756003 | 914301000006111 |
| 914311000006114 | Type II diabetes mellitus with diabetic cataract | C10FE-1 | C10FE11 | 420756003 | 914311000006114 |
| 459308015 | Type 2 diabetes mellitus with peripheral angiopathy | C10FF | C10FF00 | 314902007 | 459308015 |
| 459310018 | Type 2 diabetes mellitus with arthropathy | C10FG | C10FG00 | 314903002 | 459310018 |
| 459313016 | Type 2 diabetes mellitus with neuropathic arthropathy | C10FH | C10FH00 | 314904008 | 459313016 |
| 914391000006116 | Insulin treated Type 2 diabetes mellitus | C10FJ | C10FJ00 | 237599002 | 914391000006116 |
| 1223147012 | Insulin treated Type II diabetes mellitus | C10FJ-1 | C10FJ11 | 237599002 | 1223147012 |
| 1488898011 | Hyperosmolar non-ketotic state in type 2 diabetes mellitus | C10FK | C10FK00 | 395204000 | 1488898011 |
| 928541000006117 | Type 2 diabetes mellitus with persistent proteinuria | C10FL | C10FL00 | 421986006 | 928541000006117 |
| 928551000006115 | Type II diabetes mellitus with persistent proteinuria | C10FL-1 | C10FL11 | 421986006 | 928551000006115 |
| 928561000006118 | Type 2 diabetes mellitus with persistent microalbuminuria | C10FM | C10FM00 | 420715001 | 928561000006118 |
| 928571000006113 | Type II diabetes mellitus with persistent microalbuminuria | C10FM-1 | C10FM11 | 420715001 | 928571000006113 |
| 928581000006111 | Type 2 diabetes mellitus with ketoacidosis | C10FN | C10FN00 | 421750000 | 928581000006111 |
| 928601000006118 | Type 2 diabetes mellitus with ketoacidotic coma | C10FP | C10FP00 | 421847006 | 928601000006118 |
| 938321000006116 | Type 2 diabetes mellitus with exudative maculopathy | C10FQ | C10FQ00 | 421779007 | 938321000006116 |
| 299621000000117 | Type 2 diabetes mellitus with gastroparesis | C10FR | C10FR00 | 713703005 | 1001371000006111 |
| 251591016 | H/O: diabetes mellitus | 1434 | 1434.00 | 161445009 | 251591016 |
| 453100011 | H/O: Admission in last year for diabetes foot problem | 14F4 | 14F4.00 | 309635005 | 453100011 |
| 251892015 | H/O: insulin therapy | 14P3 | 14P3.00 | 161649006 | 251892015 |
| 616801000006114 | Diabetes resolved | 21263 | 2126300 | 315051004 | 616801000006114 |
| 459520010 | Diabetes resolved | 212H | 212H.00 | 315051004 | 459520010 |
| 285759019 | Diabetes monitoring deleted | 9OL9 | 9OL9.00 | 185761008 | 285759019 |
| 251187019 | Diabetic association member | 13Y1 | 13Y1.00 | 161102006 | 251187019 |
| 453050019 | Education score - diabetes | 3881 | 3881.00 | 309593006 | 453050019 |
| 616821000006116 | Diabetes well being questionnaire | 3882 | 3882.00 | 273413007 | 616821000006116 |
| 264676010 | Diabetic monitoring | 66A | 66A..00 | 170742000 | 264676010 |
| 264677018 | Initial diabetic assessment | 66A1 | 66A1.00 | 170743005 | 264677018 |
| 264678011 | Follow-up diabetic assessment | 66A2 | 66A2.00 | 170744004 | 264678011 |
| 264719017 | Diabetic - follow-up default | 66AM | 66AM.00 | 170771004 | 264719017 |
| 264729012 | Diabetic monitoring NOS | 66AZ | 66AZ.00 | 170742000 | 617101000006115 |
| 2533107019 | Patient diabetes education review | 66Af | 66Af.00 | 415042006 | 2533107019 |
| 301611000000118 | Diabetic monitoring - lower risk albumin excretion | 66Ak | 66Ak.00 | 199481000000100 | 301611000000118 |
| 301641000000117 | Diabetic monitoring - higher risk albumin excretion | 66Al | 66Al.00 | 199491000000103 | 301641000000117 |
| 2693189019 | Health education - diabetes | 679L | 679L.00 | 385805005 | 930491000006114 |
| 2549196011 | Patient offered diabetes structured education programme | 679R | 679R.00 | 416672007 | 2549196011 |
| 282568013 | Diabetic crisis monitoring | 8A12 | 8A12.00 | 182780004 | 282568013 |
| 147491000006111 | Self monitoring of blood glucose | 8A17 | 8A17.00 | 308113006 | 147491000006111 |
| 147501000006115 | Self monitoring of urine glucose | 8A18 | 8A18.00 | 308114000 | 147501000006115 |
| 147481000006113 | Self monitoring of blood and urine glucose | 8A19 | 8A19.00 | 308115004 | 147481000006113 |
| 1780260010 | Self monitoring urine ketones | 8A1A | 8A1A.00 | 401055007 | 1780260010 |
| 2474334019 | Diabetes clinical management plan | 8CR2 | 8CR2.00 | 412777005 | 2474334019 |
| 2548271013 | Diabetes care plan agreed | 8CS0 | 8CS0.00 | 703040004 | 985891000006110 |
| 307661000000114 | Referral to diabetic register | 8HHy | 8HHy.00 | 201961000000104 | 307661000000114 |
| 2533106011 | Referral to diabetes preconception counselling clinic | 8HTe | 8HTe.00 | 415269004 | 2533106011 |
| 308811000000110 | Referral to diabetic eye clinic | 8HTk | 8HTk.00 | 202461000000102 | 308811000000110 |
| 2548430017 | Discharged from care of diabetes specialist nurse | 8Hg4 | 8Hg4.00 | 417467000 | 2548430017 |
| 2533110014 | Referral to diabetes structured education programme | 8Hj0 | 8Hj0.00 | 415270003 | 2533110014 |
| 546871000000112 | Referral to DAFNE diabetes structured education programme | 8Hj3 | 8Hj3.00 | 306701000000102 | 546871000000112 |
| 546961000000111 | Referral to DESMOND diabetes structured education programme | 8Hj4 | 8Hj4.00 | 306741000000104 | 546961000000111 |
| 547061000000110 | Referral to XPERT diabetes structured education programme | 8Hj5 | 8Hj5.00 | 306771000000105 | 547061000000110 |
| 2159960011 | Diabetic retinopathy screening not indicated | 8I6F | 8I6F.00 | 408396006 | 2159960011 |
| 2159961010 | Diabetic foot examination not indicated | 8I6G | 8I6G.00 | 408397002 | 2159961010 |
| 547331000000115 | Did not complete diabetes structured education programme | 8I81 | 8I81.00 | 306931000000106 | 547331000000115 |
| 547441000000118 | Did not complete DAFNE diabetes structured education program | 8I82 | 8I82.00 | 306961000000101 | 547441000000118 |
| 1627291000006113 | Did not complete DESMOND diabetes structured educat program | 8I83 | 8I83.00 | 306991000000107 | 1627291000006113 |
| 1628311000006111 | Did not complete XPERT diabetes structured education program | 8I84 | 8I84.00 | 307021000000105 | 1628311000006111 |
| 308071000000112 | Patient consent given for addition to diabetic register | 93C4 | 93C4.00 | 202151000000105 | 308071000000112 |
| 2549778010 | Seen in diabetic nurse consultant clinic | 9N0m | 9N0m.00 | 417566006 | 2549778010 |
| 2549069011 | Seen in community diabetes specialist clinic | 9N0n | 9N0n.00 | 416554009 | 2549069011 |
| 2549222013 | Seen in community diabetic specialist nurse clinic | 9N0o | 9N0o.00 | 416696002 | 2549222013 |
| 285223014 | Seen in diabetic clinic | 9N1Q | 9N1Q.00 | 185229005 | 285223014 |
| 2159271018 | Seen in diabetic foot clinic | 9N1i | 9N1i.00 | 407672007 | 2159271018 |
| 300021000000110 | Seen in multidisciplinary diabetic clinic | 9N1o | 9N1o.00 | 198841000000104 | 300021000000110 |
| 457231013 | Seen in diabetic eye clinic | 9N1v | 9N1v.00 | 313340009 | 457231013 |
| 1484875010 | Seen by diabetologist | 9N2d | 9N2d.00 | 390842003 | 1484875010 |
| 448261010 | Seen by diabetic liaison nurse | 9N2i | 9N2i.00 | 305738006 | 448261010 |
| 1484954015 | DNA - Did not attend diabetic clinic | 9N4I | 9N4I.00 | 390922008 | 1484954015 |
| 301191000000119 | Did not attend diabetic retinopathy clinic | 9N4p | 9N4p.00 | 199301000000100 | 301191000000119 |
| 456686014 | Attending diabetes clinic | 9NM0 | 9NM0.00 | 312888008 | 456686014 |
| 2159955012 | Under care of diabetologist | 9NN8 | 9NN8.00 | 408391001 | 2159955012 |
| 2159956013 | Under care of diabetes specialist nurse | 9NN9 | 9NN9.00 | 408392008 | 2159956013 |
| 2159958014 | Under care of diabetic foot screener | 9NND | 9NND.00 | 408394009 | 2159958014 |
| 547191000000117 | Did not attend diabetes structured education programme | 9NiA | 9NiA.00 | 306861000000107 | 547191000000117 |
| 1628621000006112 | Did not attend DESMOND diabetes structured education program | 9NiD | 9NiD.00 | 307171000000108 | 1628621000006112 |
| 548131000000119 | Did not attend XPERT diabetes structured education programme | 9NiE | 9NiE.00 | 307201000000109 | 548131000000119 |
| 1628691000006114 | Seen by general practitioner special interest in diabetes | 9Nl4 | 9Nl4.00 | 300641000000105 | 1628691000006114 |
| 21631000000117 | Diabetes monitoring admin. | 9OL | 9OL..00 | 713671000000109 | 616761000006117 |
| 285751016 | Attends diabetes monitoring | 9OL1 | 9OL1.00 | 185753003 | 285751016 |
| 285752011 | Refuses diabetes monitoring | 9OL2 | 9OL2.00 | 185754009 | 285752011 |
| 285753018 | Diabetes monitoring default | 9OL3 | 9OL3.00 | 185755005 | 285753018 |
| 616731000006114 | Diabetes monitoring 1st letter | 9OL4 | 9OL4.00 | 185756006 | 616731000006114 |
| 616741000006116 | Diabetes monitoring 2nd letter | 9OL5 | 9OL5.00 | 185757002 | 616741000006116 |
| 616751000006119 | Diabetes monitoring 3rd letter | 9OL6 | 9OL6.00 | 185758007 | 616751000006119 |
| 616711000006115 | Diabetes monitor.verbal invite | 9OL7 | 9OL7.00 | 185759004 | 616711000006115 |
| 616701000006118 | Diabetes monitor.phone invite | 9OL8 | 9OL8.00 | 185760009 | 616701000006118 |
| 616691000006118 | Diabetes monitor. check done | 9OLA | 9OLA.00 | 270445003 | 616691000006118 |
| 405057014 | Diabetes monitored | 9OLA-1 | 9OLA.11 | 270445003 | 405057014 |
| 2533102013 | Attended diabetes structured education programme | 9OLB | 9OLB.00 | 413597006 | 2533102013 |
| 532331000000119 | Diabetes structured education programme completed | 9OLF | 9OLF.00 | 755491000000100 | 1662411000000116 |
| 546001000000113 | Attended XPERT diabetes structured education programme | 9OLG | 9OLG.00 | 306401000000106 | 546001000000113 |
| 546071000000117 | Attended DAFNE diabetes structured education programme | 9OLH | 9OLH.00 | 306441000000109 | 546071000000117 |
| 546151000000110 | DAFNE diabetes structured education programme completed | 9OLJ | 9OLJ.00 | 306471000000103 | 546151000000110 |
| 546221000000114 | DESMOND diabetes structured education programme completed | 9OLK | 9OLK.00 | 306501000000105 | 546221000000114 |
| 546291000000112 | XPERT diabetes structured education programme completed | 9OLL | 9OLL.00 | 306531000000104 | 546291000000112 |
| 546471000000114 | Diabetes structured education programme declined | 9OLM | 9OLM.00 | 306591000000103 | 546471000000114 |
| 16171000000110 | Diabetes monitoring admin.NOS | 9OLZ | 9OLZ.00 | 713671000000109 | 616771000006112 |
| 139001000000110 | Exception reporting: diabetes quality indicators | 9h4 | 9h4..00 | 716911000000104 | 931181000006112 |
| 931191000006110 | Excepted from diabetes qual indicators: Patient unsuitable | 9h41 | 9h41.00 | 717421000000100 | 931191000006110 |
| 139121000000119 | Excepted from diabetes quality indicators: Informed dissent | 9h42 | 9h42.00 | 716031000000106 | 931201000006113 |
| 479511000000117 | Diabetes type 1 review | 66An | 66An.00 | 279291000000109 | 479511000000117 |
| 616621000006115 | Diabetes mellitus, juvenile type, no mention of complication | C1000 | C100000 | 313435000 | 616621000006115 |
| 772161000006119 | Insulin dependent diabetes mellitus | C1000-1 | C100011 | 73211009 | 772161000006119 |
| 616641000006110 | Diabetes mellitus, juvenile type, with ketoacidosis | C1010 | C101000 | 420270002 | 616641000006110 |
| 292482014 | Diabetes mellitus, juvenile type, with hyperosmolar coma | C1020 | C102000 | 190330002 | 292482014 |
| 616651000006112 | Diabetes mellitus, juvenile type, with ketoacidotic coma | C1030 | C103000 | 421075007 | 616651000006112 |
| 616661000006114 | Diabetes mellitus, juvenile type, with renal manifestation | C1040 | C104000 | 421893009 | 616661000006114 |
| 616601000006113 | Diabetes mellitus, juvenile type, + ophthalmic manifestation | C1050 | C105000 | 421165007 | 616601000006113 |
| 616671000006119 | Diabetes mellitus, juvenile, + neurological manifestation | C1060 | C106000 | 421468001 | 616671000006119 |
| 616591000006117 | Diabetes mellitus, juvenile +peripheral circulatory disorder | C1070 | C107000 | 421365002 | 616591000006117 |
| 787101000006114 | IDDM with peripheral circulatory disorder | C1073 | C107300 | 421895002 | 787101000006114 |
| 772171000006114 | Insulin dependent diabetes mellitus | C108 | C108.00 | 73211009 | 772171000006114 |
| 787111000006112 | IDDM-Insulin dependent diabetes mellitus | C108-1 | C108.11 | 73211009 | 787111000006112 |
| 84281000006115 | Type 1 diabetes mellitus | C108-2 | C108.12 | 46635009 | 84281000006115 |
| 84651000006114 | Type I diabetes mellitus | C108-3 | C108.13 | 46635009 | 84651000006114 |
| 771501000006119 | Insulin-dependent diabetes mellitus with renal complications | C1080 | C108000 | 127013003 | 771501000006119 |
| 84801000006111 | Type I diabetes mellitus with renal complications | C1080-1 | C108011 | 421893009 | 84801000006111 |
| 84431000006117 | Type 1 diabetes mellitus with renal complications | C1080-2 | C108012 | 421893009 | 84431000006117 |
| 771491000006110 | Insulin-dependent diabetes mellitus with ophthalmic comps | C1081 | C108100 | 25093002 | 771491000006110 |
| 771481000006112 | Insulin-dependent diabetes mellitus with neurological comps | C1082 | C108200 | 422088007 | 771481000006112 |
| 84751000006116 | Type I diabetes mellitus with neurological complications | C1082-1 | C108211 | 421468001 | 84751000006116 |
| 84381000006113 | Type 1 diabetes mellitus with neurological complications | C1082-2 | C108212 | 421468001 | 84381000006113 |
| 771381000006115 | Insulin dependent diabetes mellitus with multiple complicatn | C1083 | C108300 | 385041000000108 | 771381000006115 |
| 72651000006114 | Unstable insulin dependent diabetes mellitus | C1084 | C108400 | 11530004 | 72651000006114 |
| 72721000006113 | Unstable type I diabetes mellitus | C1084-1 | C108411 | 290002008 | 72721000006113 |
| 72711000006117 | Unstable type 1 diabetes mellitus | C1084-2 | C108412 | 290002008 | 72711000006117 |
| 771421000006113 | Insulin dependent diabetes mellitus with ulcer | C1085 | C108500 | 19429009 | 771421000006113 |
| 84821000006118 | Type I diabetes mellitus with ulcer | C1085-1 | C108511 | 190368000 | 84821000006118 |
| 84451000006112 | Type 1 diabetes mellitus with ulcer | C1085-2 | C108512 | 190368000 | 84451000006112 |
| 771351000006111 | Insulin dependent diabetes mellitus with gangrene | C1086 | C108600 | 422275004 | 771351000006111 |
| 771411000006117 | Insulin dependent diabetes mellitus with retinopathy | C1087 | C108700 | 4855003 | 771411000006117 |
| 84811000006114 | Type I diabetes mellitus with retinopathy | C1087-1 | C108711 | 420789003 | 84811000006114 |
| 84441000006110 | Type 1 diabetes mellitus with retinopathy | C1087-2 | C108712 | 420789003 | 84441000006110 |
| 772181000006112 | Insulin dependent diabetes mellitus - poor control | C1088 | C108800 | 268519009 | 772181000006112 |
| 84661000006111 | Type I diabetes mellitus - poor control | C1088-1 | C108811 | 444073006 | 84661000006111 |
| 84291000006117 | Type 1 diabetes mellitus - poor control | C1088-2 | C108812 | 444073006 | 84291000006117 |
| 772151000006116 | Insulin dependent diabetes maturity onset | C1089 | C108900 | 73211009 | 772151000006116 |
| 84671000006116 | Type I diabetes mellitus maturity onset | C1089-1 | C108911 | 190372001 | 84671000006116 |
| 84301000006116 | Type 1 diabetes mellitus maturity onset | C1089-2 | C108912 | 190372001 | 84301000006116 |
| 746791000006111 | Insulin-dependent diabetes without complication | C108A | C108A00 | 111552007 | 746791000006111 |
| 84831000006115 | Type I diabetes mellitus without complication | C108A-1 | C108A11 | 313435000 | 84831000006115 |
| 771371000006118 | Insulin dependent diabetes mellitus with mononeuropathy | C108B | C108B00 | 230577008 | 771371000006118 |
| 771401000006115 | Insulin dependent diabetes mellitus with polyneuropathy | C108C | C108C00 | 49455004 | 771401000006115 |
| 771391000006117 | Insulin dependent diabetes mellitus with nephropathy | C108D | C108D00 | 127013003 | 771391000006117 |
| 84741000006118 | Type I diabetes mellitus with nephropathy | C108D-1 | C108D11 | 421893009 | 84741000006118 |
| 771361000006113 | Insulin dependent diabetes mellitus with hypoglycaemic coma | C108E | C108E00 | 237632004 | 771361000006113 |
| 84711000006117 | Type I diabetes mellitus with hypoglycaemic coma | C108E-1 | C108E11 | 314771006 | 84711000006117 |
| 84341000006119 | Type 1 diabetes mellitus with hypoglycaemic coma | C108E-2 | C108E12 | 314771006 | 84341000006119 |
| 771341000006114 | Insulin dependent diabetes mellitus with diabetic cataract | C108F | C108F00 | 43959009 | 771341000006114 |
| 84691000006115 | Type I diabetes mellitus with diabetic cataract | C108F-1 | C108F11 | 421920002 | 84691000006115 |
| 772141000006118 | Insulin dependent diab mell with peripheral angiopathy | C108G | C108G00 | 421895002 | 772141000006118 |
| 771331000006116 | Insulin dependent diabetes mellitus with arthropathy | C108H | C108H00 | 39710007 | 771331000006116 |
| 84681000006118 | Type I diabetes mellitus with arthropathy | C108H-1 | C108H11 | 314893005 | 84681000006118 |
| 772131000006111 | Insulin dependent diab mell with neuropathic arthropathy | C108J | C108J00 | 201724008 | 772131000006111 |
| 84761000006119 | Type I diabetes mellitus with neuropathic arthropathy | C108J-1 | C108J11 | 71771000119100 | 84761000006119 |
| 84391000006111 | Type 1 diabetes mellitus with neuropathic arthropathy | C108J-2 | C108J12 | 71771000119100 | 84391000006111 |
| 2476117016 | Maturity onset diabetes in youth type 1 | C10C-2 | C10C.12 | 609562003 | 841371000006117 |
| 197984010 | Type 1 diabetes mellitus | C10E | C10E.00 | 46635009 | 197984010 |
| 494564012 | Type I diabetes mellitus | C10E-1 | C10E.11 | 46635009 | 494564012 |
| 77727018 | Insulin dependent diabetes mellitus | C10E-2 | C10E.12 | 73211009 | 913411000006118 |
| 913451000006117 | Type 1 diabetes mellitus with renal complications | C10E0 | C10E000 | 421893009 | 913451000006117 |
| 913481000006113 | Type 1 diabetes mellitus with ophthalmic complications | C10E1 | C10E100 | 421165007 | 913481000006113 |
| 913471000006110 | Insulin-dependent diabetes mellitus with ophthalmic comps | C10E1-2 | C10E112 | 25093002 | 913471000006110 |
| 913511000006117 | Type 1 diabetes mellitus with neurological complications | C10E2 | C10E200 | 421468001 | 913511000006117 |
| 913541000006118 | Type 1 diabetes mellitus with multiple complications | C10E3 | C10E300 | 422228004 | 913541000006118 |
| 913551000006116 | Type I diabetes mellitus with multiple complications | C10E3-1 | C10E311 | 422228004 | 913551000006116 |
| 913531000006111 | Insulin dependent diabetes mellitus with multiple complicat | C10E3-2 | C10E312 | 385041000000108 | 913531000006111 |
| 429971019 | Unstable type 1 diabetes mellitus | C10E4 | C10E400 | 290002008 | 429971019 |
| 429970018 | Unstable type I diabetes mellitus | C10E4-1 | C10E411 | 290002008 | 429970018 |
| 429972014 | Unstable insulin dependent diabetes mellitus | C10E4-2 | C10E412 | 11530004 | 913561000006119 |
| 292540012 | Type 1 diabetes mellitus with ulcer | C10E5 | C10E500 | 190368000 | 292540012 |
| 292538019 | Type I diabetes mellitus with ulcer | C10E5-1 | C10E511 | 190368000 | 292538019 |
| 913591000006110 | Insulin dependent diabetes mellitus with ulcer | C10E5-2 | C10E512 | 19429009 | 913591000006110 |
| 292541011 | Type 1 diabetes mellitus with gangrene | C10E6 | C10E600 | 190369008 | 292541011 |
| 913661000006118 | Type 1 diabetes mellitus with retinopathy | C10E7 | C10E700 | 420789003 | 913661000006118 |
| 913671000006113 | Type I diabetes mellitus with retinopathy | C10E7-1 | C10E711 | 420789003 | 913671000006113 |
| 913651000006115 | Insulin dependent diabetes mellitus with retinopathy | C10E7-2 | C10E712 | 4855003 | 913651000006115 |
| 292548017 | Type 1 diabetes mellitus - poor control | C10E8 | C10E800 | 444073006 | 913691000006114 |
| 913681000006111 | Insulin dependent diabetes mellitus - poor control | C10E8-2 | C10E812 | 268519009 | 913681000006111 |
| 292553010 | Type 1 diabetes mellitus maturity onset | C10E9 | C10E900 | 190372001 | 292553010 |
| 292551012 | Type I diabetes mellitus maturity onset | C10E9-1 | C10E911 | 190372001 | 292551012 |
| 913711000006112 | Insulin dependent diabetes maturity onset | C10E9-2 | C10E912 | 73211009 | 913711000006112 |
| 457326014 | Type 1 diabetes mellitus without complication | C10EA | C10EA00 | 313435000 | 457326014 |
| 457325013 | Type I diabetes mellitus without complication | C10EA-1 | C10EA11 | 313435000 | 457325013 |
| 913781000006117 | Type 1 diabetes mellitus with mononeuropathy | C10EB | C10EB00 | 420918009 | 913781000006117 |
| 913811000006115 | Type 1 diabetes mellitus with polyneuropathy | C10EC | C10EC00 | 713705003 | 913811000006115 |
| 913821000006111 | Type I diabetes mellitus with polyneuropathy | C10EC-1 | C10EC11 | 713705003 | 913821000006111 |
| 913841000006116 | Type 1 diabetes mellitus with nephropathy | C10ED | C10ED00 | 421893009 | 913841000006116 |
| 459161015 | Type 1 diabetes mellitus with hypoglycaemic coma | C10EE | C10EE00 | 314771006 | 459161015 |
| 913901000006112 | Type 1 diabetes mellitus with diabetic cataract | C10EF | C10EF00 | 421920002 | 913901000006112 |
| 913931000006116 | Type 1 diabetes mellitus with peripheral angiopathy | C10EG | C10EG00 | 31211000119101 | 913931000006116 |
| 459292011 | Type 1 diabetes mellitus with arthropathy | C10EH | C10EH00 | 314893005 | 459292011 |
| 459296014 | Type 1 diabetes mellitus with neuropathic arthropathy | C10EJ | C10EJ00 | 71771000119100 | 3010513018 |
| 928461000006119 | Type 1 diabetes mellitus with persistent proteinuria | C10EK | C10EK00 | 420514000 | 928461000006119 |
| 1780311019 | Type 1 diabetes mellitus with persistent microalbuminuria | C10EL | C10EL00 | 401110002 | 1780311019 |
| 928501000006119 | Type 1 diabetes mellitus with ketoacidosis | C10EM | C10EM00 | 420270002 | 928501000006119 |
| 928511000006116 | Type I diabetes mellitus with ketoacidosis | C10EM-1 | C10EM11 | 420270002 | 928511000006116 |
| 928521000006112 | Type 1 diabetes mellitus with ketoacidotic coma | C10EN | C10EN00 | 421075007 | 928521000006112 |
| 928531000006110 | Type I diabetes mellitus with ketoacidotic coma | C10EN-1 | C10EN11 | 421075007 | 928531000006110 |
| 938301000006114 | Type 1 diabetes mellitus with exudative maculopathy | C10EP | C10EP00 | 420486006 | 938301000006114 |
| 938311000006112 | Type I diabetes mellitus with exudative maculopathy | C10EP-1 | C10EP11 | 420486006 | 938311000006112 |
| 299601000000114 | Type 1 diabetes mellitus with gastroparesis | C10EQ | C10EQ00 | 713702000 | 1001361000006116 |
| 616611000006111 | Diabetes mellitus, juvenile type, + unspecified complication | C10z0 | C10z000 | 420868002 | 616611000006111 |
| 306112011 | Pre-existing diabetes mellitus, insulin-dependent | L1805 | L180500 | 385051000000106 | 213121000006116 |
| 358035014 | Insulin lipohypertrophy | M21yC | M21yC00 | 238894004 | 358035014 |
| 358036010 | Insulin site lipohypertrophy | M21yC-1 | M21yC11 | 238894004 | 358036010 |
| 479571000000110 | Diabetes type 2 review | 66Ao | 66Ao.00 | 279321000000104 | 479571000000110 |
| 616511000006110 | Diabetes mellitus, adult onset, no mention of complication | C1001 | C100100 | 313436004 | 616511000006110 |
| 223291000000111 | Maturity onset diabetes | C1001-1 | C100111 | 44054006 | 223291000000111 |
| 73466011 | Non-insulin dependent diabetes mellitus | C1001-2 | C100112 | 44054006 | 73466011 |
| 616531000006116 | Diabetes mellitus, adult onset, with ketoacidosis | C1011 | C101100 | 421750000 | 616531000006116 |
| 292483016 | Diabetes mellitus, adult onset, with hyperosmolar coma | C1021 | C102100 | 190331003 | 292483016 |
| 616541000006114 | Diabetes mellitus, adult onset, with ketoacidotic coma | C1031 | C103100 | 421847006 | 616541000006114 |
| 616551000006111 | Diabetes mellitus, adult onset, with renal manifestation | C1041 | C104100 | 420279001 | 616551000006111 |
| 616491000006116 | Diabetes mellitus, adult onset, + ophthalmic manifestation | C1051 | C105100 | 422099009 | 616491000006116 |
| 616481000006119 | Diabetes mellitus, adult onset, + neurological manifestation | C1061 | C106100 | 421326000 | 616481000006119 |
| 616581000006115 | Diabetes mellitus, adult, + peripheral circulatory disorder | C1071 | C107100 | 422166005 | 616581000006115 |
| 616561000006113 | Diabetes mellitus, adult with gangrene | C1072 | C107200 | 421631007 | 616561000006113 |
| 674961000006118 | NIDDM with peripheral circulatory disorder | C1074 | C107400 | 422166005 | 674961000006118 |
| 280571000006116 | Non-insulin dependent diabetes mellitus | C109 | C109.00 | 44054006 | 280571000006116 |
| 493773010 | NIDDM - Non-insulin dependent diabetes mellitus | C109-1 | C109.11 | 44054006 | 493773010 |
| 84471000006119 | Type 2 diabetes mellitus | C109-2 | C109.12 | 44054006 | 84471000006119 |
| 84841000006113 | Type II diabetes mellitus | C109-3 | C109.13 | 44054006 | 84841000006113 |
| 587521000006111 | Non-insulin-dependent diabetes mellitus with renal comps | C1090 | C109000 | 420279001 | 587521000006111 |
| 84981000006114 | Type II diabetes mellitus with renal complications | C1090-1 | C109011 | 420279001 | 84981000006114 |
| 84611000006113 | Type 2 diabetes mellitus with renal complications | C1090-2 | C109012 | 420279001 | 84611000006113 |
| 587111000006111 | Non-insulin-dependent diabetes mellitus with ophthalm comps | C1091 | C109100 | 422099009 | 587111000006111 |
| 84951000006118 | Type II diabetes mellitus with ophthalmic complications | C1091-1 | C109111 | 422099009 | 84951000006118 |
| 84581000006117 | Type 2 diabetes mellitus with ophthalmic complications | C1091-2 | C109112 | 422099009 | 84581000006117 |
| 280591000006115 | Non-insulin-dependent diabetes mellitus with neuro comps | C1092 | C109200 | 421326000 | 280591000006115 |
| 84931000006113 | Type II diabetes mellitus with neurological complications | C1092-1 | C109211 | 421326000 | 84931000006113 |
| 84561000006110 | Type 2 diabetes mellitus with neurological complications | C1092-2 | C109212 | 421326000 | 84561000006110 |
| 280581000006118 | Non-insulin-dependent diabetes mellitus with multiple comps | C1093 | C109300 | 190388001 | 280581000006118 |
| 280551000006114 | Non-insulin dependent diabetes mellitus with ulcer | C1094 | C109400 | 190389009 | 280551000006114 |
| 85001000006117 | Type II diabetes mellitus with ulcer | C1094-1 | C109411 | 190389009 | 85001000006117 |
| 84631000006119 | Type 2 diabetes mellitus with ulcer | C1094-2 | C109412 | 190389009 | 84631000006119 |
| 281211000006117 | Non-insulin dependent diabetes mellitus with gangrene | C1095 | C109500 | 190390000 | 281211000006117 |
| 84881000006119 | Type II diabetes mellitus with gangrene | C1095-1 | C109511 | 190390000 | 84881000006119 |
| 84511000006112 | Type 2 diabetes mellitus with gangrene | C1095-2 | C109512 | 190390000 | 84511000006112 |
| 641581000006115 | Non-insulin-dependent diabetes mellitus with retinopathy | C1096 | C109600 | 422034002 | 641581000006115 |
| 84991000006112 | Type II diabetes mellitus with retinopathy | C1096-1 | C109611 | 422034002 | 84991000006112 |
| 84621000006117 | Type 2 diabetes mellitus with retinopathy | C1096-2 | C109612 | 422034002 | 84621000006117 |
| 281171000006119 | Non-insulin dependent diabetes mellitus - poor control | C1097 | C109700 | 443694000 | 281171000006119 |
| 84851000006110 | Type II diabetes mellitus - poor control | C1097-1 | C109711 | 443694000 | 84851000006110 |
| 84481000006116 | Type 2 diabetes mellitus - poor control | C1097-2 | C109712 | 443694000 | 84481000006116 |
| 457328010 | Non-insulin-dependent diabetes mellitus without complication | C1099 | C109900 | 313436004 | 457328010 |
| 280521000006117 | Non-insulin dependent diabetes mellitus with mononeuropathy | C109A | C109A00 | 420436000 | 280521000006117 |
| 84901000006117 | Type II diabetes mellitus with mononeuropathy | C109A-1 | C109A11 | 420436000 | 84901000006117 |
| 280541000006112 | Non-insulin dependent diabetes mellitus with polyneuropathy | C109B | C109B00 | 713706002 | 280541000006112 |
| 280531000006119 | Non-insulin dependent diabetes mellitus with nephropathy | C109C | C109C00 | 420279001 | 280531000006119 |
| 84921000006110 | Type II diabetes mellitus with nephropathy | C109C-1 | C109C11 | 420279001 | 84921000006110 |
| 84551000006113 | Type 2 diabetes mellitus with nephropathy | C109C-2 | C109C12 | 420279001 | 84551000006113 |
| 280511000006113 | Non-insulin dependent diabetes mellitus with hypoglyca coma | C109D | C109D00 | 719216001 | 280511000006113 |
| 84891000006116 | Type II diabetes mellitus with hypoglycaemic coma | C109D-1 | C109D11 | 719216001 | 84891000006116 |
| 84521000006116 | Type 2 diabetes mellitus with hypoglycaemic coma | C109D-2 | C109D12 | 719216001 | 84521000006116 |
| 281161000006114 | Non-insulin depend diabetes mellitus with diabetic cataract | C109E | C109E00 | 420756003 | 281161000006114 |
| 84871000006117 | Type II diabetes mellitus with diabetic cataract | C109E-1 | C109E11 | 420756003 | 84871000006117 |
| 84501000006114 | Type 2 diabetes mellitus with diabetic cataract | C109E-2 | C109E12 | 420756003 | 84501000006114 |
| 280561000006111 | Non-insulin-dependent d m with peripheral angiopath | C109F | C109F00 | 314902007 | 280561000006111 |
| 84961000006116 | Type II diabetes mellitus with peripheral angiopathy | C109F-1 | C109F11 | 314902007 | 84961000006116 |
| 84591000006119 | Type 2 diabetes mellitus with peripheral angiopathy | C109F-2 | C109F12 | 314902007 | 84591000006119 |
| 459309011 | Non-insulin dependent diabetes mellitus with arthropathy | C109G | C109G00 | 314903002 | 459309011 |
| 84861000006112 | Type II diabetes mellitus with arthropathy | C109G-1 | C109G11 | 314903002 | 84861000006112 |
| 84491000006118 | Type 2 diabetes mellitus with arthropathy | C109G-2 | C109G12 | 314903002 | 84491000006118 |
| 281181000006116 | Non-insulin dependent d m with neuropathic arthropathy | C109H | C109H00 | 314904008 | 281181000006116 |
| 84941000006115 | Type II diabetes mellitus with neuropathic arthropathy | C109H-1 | C109H11 | 314904008 | 84941000006115 |
| 84571000006115 | Type 2 diabetes mellitus with neuropathic arthropathy | C109H-2 | C109H12 | 314904008 | 84571000006115 |
| 840951000006119 | Insulin treated Type 2 diabetes mellitus | C109J | C109J00 | 237599002 | 840951000006119 |
| 1223148019 | Insulin treated non-insulin dependent diabetes mellitus | C109J-1 | C109J11 | 237599002 | 1223148019 |
| 841351000006110 | Insulin treated Type II diabetes mellitus | C109J-2 | C109J12 | 237599002 | 841351000006110 |
| 850691000006118 | Hyperosmolar non-ketotic state in type 2 diabetes mellitus | C109K | C109K00 | 395204000 | 850691000006118 |
| 483886014 | Maturity onset diabetes in youth | C10C-1 | C10C.11 | 609561005 | 841361000006112 |
| 840971000006112 | Diabetes mellitus autosomal dominant type 2 | C10D | C10D.00 | 237604008 | 840971000006112 |
| 356085010 | Maturity onset diabetes in youth type 2 | C10D-1 | C10D.11 | 237604008 | 356085010 |
| 2674067015 | Latent autoimmune diabetes mellitus in adult | C10ER | C10ER00 | 426875007 | 2674067015 |
| 197761014 | Type 2 diabetes mellitus | C10F | C10F.00 | 44054006 | 197761014 |
| 493774016 | Type II diabetes mellitus | C10F-1 | C10F.11 | 44054006 | 493774016 |
| 914031000006118 | Type 2 diabetes mellitus with renal complications | C10F0 | C10F000 | 420279001 | 914031000006118 |
| 914041000006111 | Type II diabetes mellitus with renal complications | C10F0-1 | C10F011 | 420279001 | 914041000006111 |
| 914051000006113 | Type 2 diabetes mellitus with ophthalmic complications | C10F1 | C10F100 | 422099009 | 914051000006113 |
| 914071000006115 | Type 2 diabetes mellitus with neurological complications | C10F2 | C10F200 | 421326000 | 914071000006115 |
| 914081000006117 | Type II diabetes mellitus with neurological complications | C10F2-1 | C10F211 | 421326000 | 914081000006117 |
| 292577016 | Type 2 diabetes mellitus with multiple complications | C10F3 | C10F300 | 190388001 | 292577016 |
| 292576013 | Type II diabetes mellitus with multiple complications | C10F3-1 | C10F311 | 190388001 | 292576013 |
| 292579018 | Type 2 diabetes mellitus with ulcer | C10F4 | C10F400 | 190389009 | 292579018 |
| 292581016 | Type II diabetes mellitus with ulcer | C10F4-1 | C10F411 | 190389009 | 292581016 |
| 292583018 | Type 2 diabetes mellitus with gangrene | C10F5 | C10F500 | 190390000 | 292583018 |
| 914151000006112 | Type 2 diabetes mellitus with retinopathy | C10F6 | C10F600 | 422034002 | 914151000006112 |
| 914161000006114 | Type II diabetes mellitus with retinopathy | C10F6-1 | C10F611 | 422034002 | 914161000006114 |
| 292590011 | Type 2 diabetes mellitus - poor control | C10F7 | C10F700 | 443694000 | 914171000006119 |
| 292589019 | Type II diabetes mellitus - poor control | C10F7-1 | C10F711 | 443694000 | 914181000006116 |
| 457329019 | Type 2 diabetes mellitus without complication | C10F9 | C10F900 | 313436004 | 457329019 |
| 457330012 | Type II diabetes mellitus without complication | C10F9-1 | C10F911 | 313436004 | 457330012 |
| 914221000006113 | Type 2 diabetes mellitus with mononeuropathy | C10FA | C10FA00 | 420436000 | 914221000006113 |
| 914231000006111 | Type II diabetes mellitus with mononeuropathy | C10FA-1 | C10FA11 | 420436000 | 914231000006111 |
| 914241000006118 | Type 2 diabetes mellitus with polyneuropathy | C10FB | C10FB00 | 713706002 | 914241000006118 |
| 914251000006116 | Type II diabetes mellitus with polyneuropathy | C10FB-1 | C10FB11 | 713706002 | 914251000006116 |
| 914261000006119 | Type 2 diabetes mellitus with nephropathy | C10FC | C10FC00 | 420279001 | 914261000006119 |
| 459167016 | Type 2 diabetes mellitus with hypoglycaemic coma | C10FD | C10FD00 | 719216001 | 914281000006112 |
| 459169018 | Type II diabetes mellitus with hypoglycaemic coma | C10FD-1 | C10FD11 | 719216001 | 914291000006110 |
| 914301000006111 | Type 2 diabetes mellitus with diabetic cataract | C10FE | C10FE00 | 420756003 | 914301000006111 |
| 914311000006114 | Type II diabetes mellitus with diabetic cataract | C10FE-1 | C10FE11 | 420756003 | 914311000006114 |
| 459308015 | Type 2 diabetes mellitus with peripheral angiopathy | C10FF | C10FF00 | 314902007 | 459308015 |
| 459310018 | Type 2 diabetes mellitus with arthropathy | C10FG | C10FG00 | 314903002 | 459310018 |
| 459313016 | Type 2 diabetes mellitus with neuropathic arthropathy | C10FH | C10FH00 | 314904008 | 459313016 |
| 914391000006116 | Insulin treated Type 2 diabetes mellitus | C10FJ | C10FJ00 | 237599002 | 914391000006116 |
| 1223147012 | Insulin treated Type II diabetes mellitus | C10FJ-1 | C10FJ11 | 237599002 | 1223147012 |
| 1488898011 | Hyperosmolar non-ketotic state in type 2 diabetes mellitus | C10FK | C10FK00 | 395204000 | 1488898011 |
| 928541000006117 | Type 2 diabetes mellitus with persistent proteinuria | C10FL | C10FL00 | 421986006 | 928541000006117 |
| 928551000006115 | Type II diabetes mellitus with persistent proteinuria | C10FL-1 | C10FL11 | 421986006 | 928551000006115 |
| 928561000006118 | Type 2 diabetes mellitus with persistent microalbuminuria | C10FM | C10FM00 | 420715001 | 928561000006118 |
| 928571000006113 | Type II diabetes mellitus with persistent microalbuminuria | C10FM-1 | C10FM11 | 420715001 | 928571000006113 |
| 928581000006111 | Type 2 diabetes mellitus with ketoacidosis | C10FN | C10FN00 | 421750000 | 928581000006111 |
| 928601000006118 | Type 2 diabetes mellitus with ketoacidotic coma | C10FP | C10FP00 | 421847006 | 928601000006118 |
| 938321000006116 | Type 2 diabetes mellitus with exudative maculopathy | C10FQ | C10FQ00 | 421779007 | 938321000006116 |
| 299621000000117 | Type 2 diabetes mellitus with gastroparesis | C10FR | C10FR00 | 713703005 | 1001371000006111 |
| 616571000006118 | Diabetes mellitus, adult, + other specified manifestation | C10y1 | C10y100 | 73211009 | 616571000006118 |
| 616501000006112 | Diabetes mellitus, adult onset, + unspecified complication | C10z1 | C10z100 | 422014003 | 616501000006112 |
| 306113018 | Pre-existing diabetes mellitus, non-insulin-dependent | L1806 | L180600 | 199230006 | 306113018 |
| 616241000006112 | Diabetes mellitus induced by steroids | C10B | C10B.00 | 190447002 | 616241000006112 |
| 127051000006112 | Steroid induced diabetes mellitus without complication | C10B0 | C10B000 | 190416008 | 127051000006112 |
| 622221000000118 | Maternally inherited diabetes mellitus | C10FS | C10FS00 | 335621000000101 | 622221000000118 |
| 1230929011 | Secondary pancreatic diabetes mellitus | C10G | C10G.00 | 51002006 | 1230929011 |
| 198461000000116 | Secondary pancreatic diabetes mellitus without complication | C10G0 | C10G000 | 51002006 | 967611000006114 |
| 2160090014 | Diabetes mellitus induced by non-steroid drugs | C10H | C10H.00 | 408540003 | 2160090014 |
| 967631000006115 | DM induced by non-steroid drugs without complication | C10H0 | C10H000 | 413183008 | 967631000006115 |
| 15518018 | Secondary diabetes mellitus | C10N | C10N.00 | 8801005 | 15518018 |
| 189721000000113 | Secondary diabetes mellitus without complication | C10N0 | C10N000 | 8801005 | 967731000006112 |
| 494831000000119 | Cystic fibrosis related diabetes mellitus | C10N1 | C10N100 | 426705001 | 1591251000006112 |
| 127041000006110 | Steroid induced diabetes | C11y0 | C11y000 | 190447002 | 127041000006110 |
| 459694018 | Diabetic lipid lowering diet | 13AB | 13AB.00 | 315207000 | 459694018 |
| 459695017 | Diabetic weight reducing diet | 13AC | 13AC.00 | 315208005 | 459695017 |
| 250447011 | Diabetic diet | 13B1 | 13B1.00 | 160670007 | 250447011 |
| 169731000006118 | Retinal abnormality - diabetes related | 2BBF | 2BBF.00 | 4855003 | 169731000006118 |
| 1484887015 | O/E - diabetic maculopathy present both eyes | 2BBL | 2BBL.00 | 390854003 | 1484887015 |
| 1484888013 | O/E - diabetic maculopathy absent both eyes | 2BBM | 2BBM.00 | 390855002 | 1484888013 |
| 2159973010 | O/E - right eye background diabetic retinopathy | 2BBP | 2BBP.00 | 408409007 | 2159973010 |
| 2159974016 | O/E - left eye background diabetic retinopathy | 2BBQ | 2BBQ.00 | 408410002 | 2159974016 |
| 2159975015 | O/E - right eye preproliferative diabetic retinopathy | 2BBR | 2BBR.00 | 408411003 | 2159975015 |
| 2159976019 | O/E - left eye preproliferative diabetic retinopathy | 2BBS | 2BBS.00 | 408412005 | 2159976019 |
| 2159977011 | O/E - right eye proliferative diabetic retinopathy | 2BBT | 2BBT.00 | 408413000 | 2159977011 |
| 2159978018 | O/E - left eye proliferative diabetic retinopathy | 2BBV | 2BBV.00 | 408414006 | 2159978018 |
| 2159979014 | O/E - right eye diabetic maculopathy | 2BBW | 2BBW.00 | 408415007 | 2159979014 |
| 2159980012 | O/E - left eye diabetic maculopathy | 2BBX | 2BBX.00 | 408416008 | 2159980012 |
| 975251000006111 | O/E - right eye stable treated prolif diabetic retinopathy | 2BBk | 2BBk.00 | 414910007 | 975251000006111 |
| 975261000006113 | O/E - left eye stable treated prolif diabetic retinopathy | 2BBl | 2BBl.00 | 414894003 | 975261000006113 |
| 2549896013 | O/E - sight threatening diabetic retinopathy | 2BBo | 2BBo.00 | 417677008 | 2549896013 |
| 764191000006112 | Foot abnormality - diabetes related | 2G510 | 2G51000 | 280137006 | 764191000006112 |
| 451410016 | O/E - Right diabetic foot at risk | 2G5A | 2G5A.00 | 308105005 | 451410016 |
| 451411017 | O/E - Left diabetic foot at risk | 2G5B | 2G5B.00 | 308106006 | 451411017 |
| 764201000006110 | Foot abnormality - diabetes related | 2G5C | 2G5C.00 | 280137006 | 764201000006110 |
| 1488393013 | O/E - Right diabetic foot at low risk | 2G5E | 2G5E.00 | 394671009 | 1488393013 |
| 1488404014 | O/E - Right diabetic foot at moderate risk | 2G5F | 2G5F.00 | 394682001 | 1488404014 |
| 1488394019 | O/E - Right diabetic foot at high risk | 2G5G | 2G5G.00 | 394672002 | 1488394019 |
| 1488395018 | O/E - Right diabetic foot - ulcerated | 2G5H | 2G5H.00 | 394673007 | 1488395018 |
| 1488397014 | O/E - Left diabetic foot at low risk | 2G5I | 2G5I.00 | 394675000 | 1488397014 |
| 1488403015 | O/E - Left diabetic foot at moderate risk | 2G5J | 2G5J.00 | 394681008 | 1488403015 |
| 1488398016 | O/E - Left diabetic foot at high risk | 2G5K | 2G5K.00 | 394676004 | 1488398016 |
| 1488396017 | O/E - Left diabetic foot - ulcerated | 2G5L | 2G5L.00 | 394674001 | 1488396017 |
| 2532977011 | O/E - right chronic diabetic foot ulcer | 2G5V | 2G5V.00 | 414906009 | 2532977011 |
| 2532976019 | O/E - left chronic diabetic foot ulcer | 2G5W | 2G5W.00 | 414890007 | 2532976019 |
| 264679015 | Diabetic on diet only | 66A3 | 66A3.00 | 170745003 | 264679015 |
| 264681018 | Diabetic on oral treatment | 66A4 | 66A4.00 | 170746002 | 264681018 |
| 264682013 | Diabetic on insulin | 66A5 | 66A5.00 | 170747006 | 264682013 |
| 264693010 | Has seen dietician - diabetes | 66A8 | 66A8.00 | 170752001 | 264693010 |
| 264695015 | Understands diet - diabetes | 66A9 | 66A9.00 | 170753006 | 264695015 |
| 777231000006119 | Injection sites - diabetic | 66AA-1 | 66AA.11 | 268517006 | 777231000006119 |
| 264701014 | Fundoscopy - diabetic check | 66AD | 66AD.00 | 170757007 | 264701014 |
| 264704018 | Diabetic drug side effects | 66AG | 66AG.00 | 170760000 | 264704018 |
| 264705017 | Diabetic treatment changed | 66AH | 66AH.00 | 170761001 | 264705017 |
| 264707013 | Diabetic - good control | 66AI | 66AI.00 | 170763003 | 264707013 |
| 401531012 | Diabetic - poor control | 66AJ | 66AJ.00 | 268519009 | 401531012 |
| 411891014 | Unstable diabetes | 66AJ-1 | 66AJ.11 | 11530004 | 72621000006117 |
| 19931010 | Brittle diabetes | 66AJ1 | 66AJ100 | 11530004 | 19931010 |
| 264716012 | Diabetic - poor control NOS | 66AJz | 66AJz00 | 268519009 | 616911000006117 |
| 264717015 | Diabetic - cooperative patient | 66AK | 66AK.00 | 170769004 | 264717015 |
| 264718013 | Diabetic-uncooperative patient | 66AL | 66AL.00 | 170770003 | 264718013 |
| 264720011 | Date diabetic treatment start | 66AN | 66AN.00 | 170772006 | 264720011 |
| 607851000006113 | Date diabetic treatment stopp. | 66AO | 66AO.00 | 170773001 | 607851000006113 |
| 264723013 | Diabetes: practice programme | 66AP | 66AP.00 | 170774007 | 264723013 |
| 264724019 | Diabetes: shared care programme | 66AQ | 66AQ.00 | 170775008 | 264724019 |
| 264726017 | Diabetes management plan given | 66AR | 66AR.00 | 170742000 | 616121000006110 |
| 264727014 | Diabetic annual review | 66AS | 66AS.00 | 170777000 | 264727014 |
| 264728016 | Annual diabetic blood test | 66AT | 66AT.00 | 170778005 | 264728016 |
| 216195015 | Diabetes care by hospital only | 66AU | 66AU.00 | 243860001 | 838711000006116 |
| 458512016 | Diabetic on insulin and oral treatment | 66AV | 66AV.00 | 314194001 | 458512016 |
| 1488405010 | Diabetic foot risk assessment | 66AW | 66AW.00 | 394683006 | 1488405010 |
| 845341000006111 | Diabetes: shared care in pregnancy - diabetol and obstet | 66AX | 66AX.00 | 24451000000107 | 845341000006111 |
| 63021000000116 | Diabetic diet - good compliance | 66AY | 66AY.00 | 25351000000101 | 63021000000116 |
| 63041000000111 | Diabetic diet - poor compliance | 66Aa | 66Aa.00 | 25361000000103 | 63041000000111 |
| 1780386019 | Diabetic foot examination | 66Ab | 66Ab.00 | 401191002 | 1780386019 |
| 1780286017 | Diabetic peripheral neuropathy screening | 66Ac | 66Ac.00 | 401081006 | 1780286017 |
| 299191000000110 | Insulin needles changed daily | 66Ag | 66Ag.00 | 198471000000107 | 299191000000110 |
| 299211000000114 | Insulin needles changed for each injection | 66Ah | 66Ah.00 | 198481000000109 | 299211000000114 |
| 299261000000111 | Diabetic 6 month review | 66Ai | 66Ai.00 | 198501000000100 | 299261000000111 |
| 301161000000113 | Insulin needles changed less than once a day | 66Aj | 66Aj.00 | 199291000000104 | 301161000000113 |
| 472901000000116 | Insulin dose changed | 66Am | 66Am.00 | 703972004 | 3012379017 |
| 646031000000112 | Insulin treatment initiated | 66Ap | 66Ap.00 | 345041000000101 | 646031000000112 |
| 692951000000111 | Diabetic foot screen | 66Aq | 66Aq.00 | 367011000000100 | 692951000000111 |
| 1488406011 | Diabetic pre-pregnancy counselling | 6761 | 6761.00 | 394684000 | 1488406011 |
| 216201011 | Diabetic retinopathy screening | 68A7 | 68A7.00 | 134395001 | 216201011 |
| 2548205017 | Diabetic retinopathy screening offered | 68A9 | 68A9.00 | 417256004 | 2548205017 |
| 405561000000113 | Diabetic digital retinopathy screening offered | 68AB | 68AB.00 | 247771000000108 | 405561000000113 |
| 2474726011 | Pan retinal photocoagulation for diabetes | 7276 | 7276.00 | 413180006 | 2474726011 |
| 278718015 | Continuous subcutaneous infusion of insulin | 7L100 | 7L10000 | 180178009 | 278718015 |
| 452078013 | Subcutaneous injection of insulin | 7L198 | 7L19800 | 308755006 | 452078013 |
| 616081000006113 | Diab mellit insulin-glucose infus acute myocardial infarct | 889A | 889A.00 | 315287002 | 616081000006113 |
| 282570016 | Diabetic stabilisation | 8A13 | 8A13.00 | 182781000 | 282570016 |
| 1488441010 | Diabetes medication review | 8B3l | 8B3l.00 | 394725008 | 1488441010 |
| 2159170012 | Patient on maximal tolerated therapy for diabetes | 8BL2 | 8BL2.00 | 407569005 | 2159170012 |
| 200111000006116 | Pt advised re diabetic diet | 8CA41 | 8CA4100 | 284350006 | 200111000006116 |
| 1780274015 | Advice about blood glucose control | 8CAQ | 8CAQ.00 | 401069007 | 1780274015 |
| 2532974016 | Transition of diabetes care options discussed | 8CP2 | 8CP2.00 | 415744000 | 2532974016 |
| 283544013 | Admit diabetic emergency | 8H2J | 8H2J.00 | 183472000 | 283544013 |
| 283584016 | Non-urgent diabetic admission | 8H3O | 8H3O.00 | 183506001 | 283584016 |
| 1484923019 | Refer to diabetic foot screener | 8H7r | 8H7r.00 | 390890005 | 1484923019 |
| 2159948010 | Diabetic retinopathy 12 month review | 8HBG | 8HBG.00 | 408384004 | 2159948010 |
| 2159949019 | Diabetic retinopathy 6 month review | 8HBH | 8HBH.00 | 408385003 | 2159949019 |
| 283938017 | Diabetology D.V. done | 8HLE | 8HLE.00 | 183751004 | 283938017 |
| 406351000000114 | Referral for diabetic retinopathy screening | 8Hl1 | 8Hl1.00 | 248161000000101 | 406351000000114 |
| 2474309019 | Diabetic foot examination declined | 8I3W | 8I3W.00 | 412752009 | 2474309019 |
| 2474671011 | Diabetic retinopathy screening refused | 8I3X | 8I3X.00 | 413122001 | 2474671011 |
| 298811000000118 | Insulin therapy declined | 8I3k | 8I3k.00 | 198321000000100 | 298811000000118 |
| 2548633012 | Patient held diabetic record declined | 8I57 | 8I57.00 | 416135000 | 2548633012 |
| 2159969012 | Patient held diabetic record issued | 9360 | 9360.00 | 408405001 | 2159969012 |
| 2549901010 | Diabetic patient unsuitable for digital retinal photography | 9OLD | 9OLD.00 | 417681008 | 2549901010 |
| 121589010 | Diabetes mellitus | C10 | C10..00 | 73211009 | 121589010 |
| 292466013 | Diabetes mellitus with no mention of complication | C100 | C100.00 | 111552007 | 616411000006114 |
| 292475010 | Diabetes mellitus NOS with no mention of complication | C100z | C100z00 | 111552007 | 616291000006115 |
| 2622193012 | Diabetes mellitus with ketoacidosis | C101 | C101.00 | 420422005 | 2622193012 |
| 292478012 | Other specified diabetes mellitus with ketoacidosis | C101y | C101y00 | 420422005 | 13931000006114 |
| 292479016 | Diabetes mellitus NOS with ketoacidosis | C101z | C101z00 | 420422005 | 616261000006111 |
| 292480018 | Diabetes mellitus with hyperosmolar coma | C102 | C102.00 | 422126006 | 616361000006118 |
| 292484010 | Diabetes mellitus NOS with hyperosmolar coma | C102z | C102z00 | 422126006 | 616251000006114 |
| 616381000006111 | Diabetes mellitus with ketoacidotic coma | C103 | C103.00 | 26298008 | 616381000006111 |
| 292488013 | Other specified diabetes mellitus with coma | C103y | C103y00 | 420662003 | 13921000006111 |
| 292489017 | Diabetes mellitus NOS with ketoacidotic coma | C103z | C103z00 | 420422005 | 616271000006116 |
| 616461000006112 | Diabetes mellitus with renal manifestation | C104 | C104.00 | 127013003 | 616461000006112 |
| 205225016 | Diabetic nephropathy | C104-1 | C104.11 | 127013003 | 205225016 |
| 292495016 | Other specified diabetes mellitus with renal complications | C104y | C104y00 | 127013003 | 13801000006112 |
| 292496015 | Diabetes mellitis with nephropathy NOS | C104z | C104z00 | 127013003 | 616131000006113 |
| 616421000006118 | Diabetes mellitus with ophthalmic manifestation | C105 | C105.00 | 25093002 | 616421000006118 |
| 13771000006110 | Other specified diabetes mellitus with ophthalmic complicatn | C105y | C105y00 | 25093002 | 13771000006110 |
| 292503016 | Diabetes mellitus NOS with ophthalmic manifestation | C105z | C105z00 | 25093002 | 616301000006119 |
| 616391000006114 | Diabetes mellitus with neurological manifestation | C106 | C106.00 | 422088007 | 616391000006114 |
| 616921000006113 | Diabetic amyotrophy | C106-1 | C106.11 | 39058009 | 616921000006113 |
| 345487013 | Diabetes mellitus with neuropathy | C106-2 | C106.12 | 230572002 | 345487013 |
| 616451000006110 | Diabetes mellitus with polyneuropathy | C106-3 | C106.13 | 49455004 | 616451000006110 |
| 13761000006115 | Other specified diabetes mellitus with neurological comps | C106y | C106y00 | 422088007 | 13761000006115 |
| 292512019 | Diabetes mellitus NOS with neurological manifestation | C106z | C106z00 | 422088007 | 616281000006118 |
| 616441000006113 | Diabetes mellitus with peripheral circulatory disorder | C107 | C107.00 | 421895002 | 616441000006113 |
| 616351000006115 | Diabetes mellitus with gangrene | C107-1 | C107.11 | 422275004 | 616351000006115 |
| 616831000006118 | Diabetes with gangrene | C107-2 | C107.12 | 422275004 | 616831000006118 |
| 292523015 | Diabetes mellitus NOS with peripheral circulatory disorder | C107z | C107z00 | 421895002 | 616321000006112 |
| 13751000006117 | Other specified diabetes mellitus with multiple comps | C108y | C108y00 | 385041000000108 | 13751000006117 |
| 292565014 | Unspecified diabetes mellitus with multiple complications | C108z | C108z00 | 385041000000108 | 76061000006116 |
| 719531000006118 | Malnutrition-related diabetes mellitus | C10A | C10A.00 | 75524006 | 719531000006118 |
| 719541000006111 | Malnutrition-related diabetes mellitus with coma | C10A0 | C10A000 | 420996007 | 719541000006111 |
| 292606016 | Malnutrition-related diabetes mellitus with ketoacidosis | C10A1 | C10A100 | 190406000 | 292606016 |
| 483882011 | Diabetes mellitus autosomal dominant | C10C | C10C.00 | 609561005 | 840961000006117 |
| 967701000006116 | Lipoatrophic diabetes mellitus | C10M | C10M.00 | 127012008 | 967701000006116 |
| 292617015 | Diabetes mellitus with other specified manifestation | C10y | C10y.00 | 74627003 | 616431000006115 |
| 13781000006113 | Other specified diabetes mellitus with other spec comps | C10yy | C10yy00 | 73211009 | 13781000006113 |
| 292621010 | Diabetes mellitus NOS with other specified manifestation | C10yz | C10yz00 | 73211009 | 616311000006116 |
| 292622015 | Diabetes mellitus with unspecified complication | C10z | C10z.00 | 74627003 | 616471000006117 |
| 13811000006110 | Other specified diabetes mellitus with unspecified comps | C10zy | C10zy00 | 74627003 | 13811000006110 |
| 292626017 | Diabetes mellitus NOS with unspecified complication | C10zz | C10zz00 | 74627003 | 616331000006110 |
| 354316011 | Renal diabetes | C314-1 | C314.11 | 127013003 | 176691000006113 |
| 1786715019 | Bronzed diabetes | C3500-1 | C350011 | 399144008 | 1786715019 |
| 377001000006117 | [X]Diabetes mellitus | Cyu2 | Cyu2.00 | 73211009 | 377001000006117 |
| 293756010 | [X]Other specified diabetes mellitus | Cyu20 | Cyu2000 | 73211009 | 414581000006113 |
| 1230890017 | Autonomic neuropathy due to diabetes | F1711 | F171100 | 50620007 | 1230890017 |
| 297492014 | Diabetic mononeuritis multiplex | F3450 | F345000 | 193141005 | 297492014 |
| 297510017 | Diabetic mononeuritis NOS | F35z0 | F35z000 | 193141005 | 617121000006113 |
| 214921000006116 | Polyneuropathy in diabetes | F372 | F372.00 | 49455004 | 219301000000119 |
| 82373015 | Diabetic polyneuropathy | F372-1 | F372.11 | 49455004 | 82373015 |
| 345486016 | Diabetic neuropathy | F372-2 | F372.12 | 230572002 | 345486016 |
| 297550019 | Acute painful diabetic neuropathy | F3720 | F372000 | 193183000 | 297550019 |
| 297551015 | Chronic painful diabetic neuropathy | F3721 | F372100 | 193184006 | 297551015 |
| 297552010 | Asymptomatic diabetic neuropathy | F3722 | F372200 | 193185007 | 297552010 |
| 399419012 | Myasthenic syndrome due to diabetic amyotrophy | F3813 | F381300 | 267604001 | 399419012 |
| 65526011 | Diabetic amyotrophy | F3813-1 | F381311 | 39058009 | 65526011 |
| 345492010 | Diabetic mononeuropathy | F3y0 | F3y0.00 | 230577008 | 345492010 |
| 9093013 | Diabetic retinopathy | F420 | F420.00 | 4855003 | 9093013 |
| 1785332013 | Background diabetic retinopathy | F4200 | F420000 | 390834004 | 1785332013 |
| 98476015 | Proliferative diabetic retinopathy | F4201 | F420100 | 59276001 | 98476015 |
| 297754014 | Preproliferative diabetic retinopathy | F4202 | F420200 | 193349004 | 297754014 |
| 297755010 | Advanced diabetic maculopathy | F4203 | F420300 | 193350004 | 297755010 |
| 347657010 | Diabetic maculopathy | F4204 | F420400 | 232020009 | 347657010 |
| 455408014 | Advanced diabetic retinal disease | F4205 | F420500 | 311782002 | 455408014 |
| 1484867016 | Non proliferative diabetic retinopathy | F4206 | F420600 | 390834004 | 1484867016 |
| 1785163015 | High risk proliferative diabetic retinopathy | F4207 | F420700 | 312907002 | 1785163015 |
| 841011000006112 | High risk non proliferative diabetic retinopathy | F4208 | F420800 | 312905005 | 841011000006112 |
| 297758012 | Diabetic retinopathy NOS | F420z | F420z00 | 4855003 | 617231000006117 |
| 297964010 | Diabetic iritis | F4407 | F440700 | 193489006 | 297964010 |
| 73294018 | Diabetic cataract | F4640 | F464000 | 43959009 | 73294018 |
| 302011 | Diabetic peripheral angiopathy | G73y0 | G73y000 | 127014009 | 302011 |
| 303846016 | Nephrotic syndrome in diabetes mellitus | K01x1 | K01x100 | 197605007 | 303846016 |
| 303847013 | Kimmelstiel - Wilson disease | K01x1-1 | K01x111 | 197605007 | 303847013 |
| 213141000006111 | Pre-existing diabetes mellitus, unspecified | L180X | L180X00 | 385051000000106 | 213141000006111 |
| 308360012 | Cellulitis in diabetic foot | M0372 | M037200 | 200687002 | 308360012 |
| 309177010 | Ischaemic ulcer diabetic foot | M2710 | M271000 | 201250006 | 309177010 |
| 309179013 | Neuropathic diabetic ulcer - foot | M2711 | M271100 | 201251005 | 309179013 |
| 309180011 | Mixed diabetic ulcer - foot | M2712 | M271200 | 201252003 | 309180011 |
| 309737011 | Diabetic cheiroarthropathy | N0300 | N030000 | 201723002 | 309737011 |
| 309738018 | Diabetic cheiropathy | N0300-1 | N030011 | 201723002 | 309738018 |
| 616971000006114 | Diabetic Charcot arthropathy | N0301 | N030100 | 201724008 | 616971000006114 |
| 82979011 | Neonatal diabetes mellitus | Q441 | Q441.00 | 49817004 | 82979011 |
| 317347010 | [D]Gangrene of toe in diabetic | R0542 | R054200 | 195302000 | 298081000006114 |
| 317348017 | [D]Widespread diabetic foot gangrene | R0543 | R054300 | 195303005 | 303981000006110 |
| 468191000006114 | Adverse reaction to insulins and antidiabetic agents | TJ23 | TJ23.00 | 430400006 | 468191000006114 |
| 330806013 | Adverse reaction to insulins and antidiabetic agents NOS | TJ23z | TJ23z00 | 430400006 | 468201000006112 |
| 354661000006118 | [X] Adverse reaction to insulins and antidiabetic agents | U6023-1 | U602311 | 430400006 | 354661000006118 |
| 1227774019 | [V]Dietary counselling in diabetes mellitus | ZV653-2 | ZV65312 | 441901000000108 | 890121000000114 |
| 459705019 | Diabetes mellitus excluded | 1I0 | 1I0..00 | 315216001 | 459705019 |

Supplementary Table S21: Read Codes for Hypertension

| MedCode Id | Term | Original Read Code | Cleansed Read Code | Snomed CT Concept Id | Snomed CT Description Id |
| --- | --- | --- | --- | --- | --- |
| 251674014 | H/O: hypertension | 14A2 | 14A2.00 | 161501007 | 251674014 |
| 790131000006118 | Hypertension resolved | 21261 | 2126100 | 162659009 | 790131000006118 |
| 253532015 | Hypertension resolved | 212K | 212K.00 | 162659009 | 253532015 |
| 789981000006111 | Hypertens.monitor deleted | 9OI9 | 9OI9.00 | 185724004 | 789981000006111 |
| 2548266018 | Suspected hypertension | 1JD | 1JD..00 | 417312002 | 2548266018 |
| 413076015 | White coat hypertension | 246M | 246M.00 | 697930002 | 2968360017 |
| 411919011 | Hypertension monitoring | 662-2 | 662..12 | 275944005 | 411919011 |
| 264473010 | Hypertension:follow-up default | 6629 | 6629.00 | 170579000 | 264473010 |
| 264487010 | Hypertension treatm.stopped | 662H | 662H.00 | 170588009 | 264487010 |
| 790091000006115 | Hypertension monitoring | 662P | 662P.00 | 275944005 | 790091000006115 |
| 2474335018 | Hypertension clinical management plan | 8CR4 | 8CR4.00 | 412779008 | 2474335018 |
| 285265015 | Seen in hypertension clinic | 9N03 | 9N03.00 | 185264001 | 285265015 |
| 153941000006118 | Seen in hypertension clinic | 9N1y2 | 9N1y200 | 185264001 | 153941000006118 |
| 1484957010 | DNA - Did not attend hypertension clinic | 9N4L | 9N4L.00 | 390925005 | 1484957010 |
| 21601000000111 | Hypertension monitoring admin. | 9OI | 9OI..00 | 713641000000103 | 790111000006112 |
| 26091000000116 | Hypertension clinic admin. | 9OI-1 | 9OI..11 | 713661000000102 | 790041000006112 |
| 498791000006113 | Attends hypertension monitor. | 9OI1 | 9OI1.00 | 185716009 | 498791000006113 |
| 179371000006114 | Refuses hypertension monitor. | 9OI2 | 9OI2.00 | 185717000 | 179371000006114 |
| 789951000006115 | Hyperten.monitor offer default | 9OI3 | 9OI3.00 | 185718005 | 789951000006115 |
| 790011000006113 | Hypertens.monitor.1st letter | 9OI4 | 9OI4.00 | 185719002 | 790011000006113 |
| 789961000006118 | Hypertens.monitor 2nd letter | 9OI5 | 9OI5.00 | 185720008 | 789961000006118 |
| 789971000006113 | Hypertens.monitor 3rd letter | 9OI6 | 9OI6.00 | 185721007 | 789971000006113 |
| 790001000006110 | Hypertens.monitor verbal inv. | 9OI7 | 9OI7.00 | 185722000 | 790001000006110 |
| 789991000006114 | Hypertens.monitor phone invite | 9OI8 | 9OI8.00 | 185723005 | 789991000006114 |
| 790071000006116 | Hypertension monitor.chck done | 9OIA | 9OIA.00 | 270440008 | 790071000006116 |
| 405053013 | Hypertension monitored | 9OIA-1 | 9OIA.11 | 270440008 | 405053013 |
| 16051000000119 | Hypertens.monitoring admin.NOS | 9OIZ | 9OIZ.00 | 713641000000103 | 790021000006117 |
| 138991000000110 | Exception reporting: hypertension quality indicators | 9h3 | 9h3..00 | 717311000000102 | 931151000006116 |
| 931161000006119 | Excepted from hypertension qual indicators: Patient unsuit | 9h31 | 9h31.00 | 716821000000100 | 931161000006119 |
| 931171000006114 | Excepted from hypertension qual indicators: Informed dissent | 9h32 | 9h32.00 | 716771000000106 | 931171000006114 |
| 264467014 | Borderline hyperten:yearly obs | 6624 | 6624.00 | 170574005 | 264467014 |
| 264471012 | Good hypertension control | 6627 | 6627.00 | 170577003 | 264471012 |
| 264472017 | Poor hypertension control | 6628 | 6628.00 | 170578008 | 264472017 |
| 264485019 | Hypertension treatm. started | 662F | 662F.00 | 170586008 | 264485019 |
| 264486018 | Hypertensive treatm.changed | 662G | 662G.00 | 170587004 | 264486018 |
| 443764015 | On treatment for hypertension | 662O | 662O.00 | 302192008 | 443764015 |
| 1780318013 | Moderate hypertension control | 662b | 662b.00 | 401117004 | 1780318013 |
| 1780253016 | Hypertension six month review | 662c | 662c.00 | 401048005 | 1780253016 |
| 1780319017 | Hypertension annual review | 662d | 662d.00 | 401118009 | 1780319017 |
| 530221000000112 | Trial withdrawal of antihypertensive therapy | 662r | 662r.00 | 299561000000105 | 530221000000112 |
| 350601000000116 | High cost hypertension drugs | 7Q01 | 7Q01.00 | 220901000000101 | 350601000000116 |
| 451424017 | Antihypertensive therapy | 8B26 | 8B26.00 | 308116003 | 451424017 |
| 2159168015 | Patient on maximal tolerated antihypertensive therapy | 8BL0 | 8BL0.00 | 407567007 | 2159168015 |
| 1780252014 | Hypertension treatment refused | 8I3N | 8I3N.00 | 401047000 | 1780252014 |
| 1529013 | Blind hypertensive eye | F4042 | F404200 | 264008 | 1529013 |
| 12496011 | Hypertensive retinopathy | F4213 | F421300 | 6962006 | 12496011 |
| 64168014 | Hypertensive disease | G2 | G2...00 | 38341003 | 64168014 |
| 523801000006119 | BP - hypertensive disease | G2-1 | G2...11 | 38341003 | 523801000006119 |
| 99042012 | Essential hypertension | G20 | G20..00 | 59621000 | 99042012 |
| 131046010 | Malignant essential hypertension | G200 | G200.00 | 78975002 | 131046010 |
| 3135013 | Benign essential hypertension | G201 | G201.00 | 1201005 | 3135013 |
| 93494011 | Systolic hypertension | G202 | G202.00 | 56218007 | 93494011 |
| 80224019 | Diastolic hypertension | G203 | G203.00 | 48146000 | 80224019 |
| 395751018 | Essential hypertension NOS | G20z | G20z.00 | 59621000 | 648911000006113 |
| 790121000006116 | Hypertension NOS | G20z-1 | G20z.11 | 38341003 | 790121000006116 |
| 107545013 | Hypertensive heart disease | G21 | G21..00 | 64715009 | 107545013 |
| 90135019 | Malignant hypertensive heart disease | G210 | G210.00 | 54225002 | 90135019 |
| 728681000006116 | Malignant hypertensive heart disease without CCF | G2100 | G210000 | 36315003 | 728681000006116 |
| 728671000006119 | Malignant hypertensive heart disease with CCF | G2101 | G210100 | 83105008 | 728671000006119 |
| 60444016 | Benign hypertensive heart disease | G211 | G211.00 | 36221001 | 60444016 |
| 504911000006115 | Benign hypertensive heart disease without CCF | G2110 | G211000 | 77970009 | 504911000006115 |
| 504901000006118 | Benign hypertensive heart disease with CCF | G2111 | G211100 | 194767001 | 504901000006118 |
| 741661000006118 | Hypertensive heart disease NOS | G21z | G21z.00 | 64715009 | 741661000006118 |
| 741691000006114 | Hypertensive heart disease NOS without CCF | G21z0 | G21z000 | 64715009 | 741691000006114 |
| 411508017 | Cardiomegaly - hypertensive | G21z0-1 | G21z011 | 275516004 | 411508017 |
| 741681000006111 | Hypertensive heart disease NOS with CCF | G21z1 | G21z100 | 64715009 | 741681000006111 |
| 299655012 | Hypertensive heart disease NOS | G21zz | G21zz00 | 64715009 | 741671000006113 |
| 64282015 | Hypertensive renal disease | G22 | G22..00 | 38481006 | 64282015 |
| 108730018 | Malignant hypertensive renal disease | G220 | G220.00 | 65443008 | 108730018 |
| 1409014 | Benign hypertensive renal disease | G221 | G221.00 | 193003 | 1409014 |
| 299665018 | Hypertensive renal disease with renal failure | G222 | G222.00 | 194774006 | 299665018 |
| 395753015 | Hypertensive renal disease NOS | G22z | G22z.00 | 38481006 | 741721000006116 |
| 47076011 | Renal hypertension | G22z-1 | G22z.11 | 38481006 | 176911000006114 |
| 143003017 | Hypertensive heart AND renal disease | G23 | G23..00 | 86234004 | 143003017 |
| 110659019 | Malignant hypertensive heart AND renal disease | G230 | G230.00 | 66610008 | 110659019 |
| 109700019 | Benign hypertensive heart AND renal disease | G231 | G231.00 | 66052004 | 109700019 |
| 741701000006114 | Hypertensive heart&renal dis wth (congestive) heart failure | G232 | G232.00 | 194779001 | 741701000006114 |
| 299673010 | Hypertensive heart and renal disease with renal failure | G233 | G233.00 | 194780003 | 299673010 |
| 789941000006117 | Hyperten heart&renal dis+both(congestv)heart and renal fail | G234 | G234.00 | 194781004 | 789941000006117 |
| 299675015 | Hypertensive heart and renal disease NOS | G23z | G23z.00 | 86234004 | 741631000006110 |
| 299686018 | Other specified hypertensive disease | G2y | G2y..00 | 38341003 | 10441000006116 |
| 299687010 | Hypertensive disease NOS | G2z | G2z..00 | 38341003 | 741601000006119 |
| 84111015 | Hypertensive encephalopathy | G672 | G672.00 | 50490005 | 84111015 |
| 84112010 | Hypertensive crisis | G672-1 | G672.11 | 50490005 | 84112010 |
| 300869017 | [X]Hypertensive diseases | Gyu2 | Gyu2.00 | 38341003 | 389341000006117 |
| 305764016 | Other pre-existing hypertension in preg/childbirth/puerp | L122 | L122.00 | 86041002 | 19461000006113 |
| 19451000006111 | Other pre-existing hypertension in preg/childb/puerp unspec | L1220 | L122000 | 86041002 | 19451000006111 |
| 19431000006116 | Other pre-existing hypertension in preg/childb/puerp - deliv | L1221 | L122100 | 86041002 | 19431000006116 |
| 19421000006119 | Other pre-exist hypertension in preg/childb/puerp-not deliv | L1223 | L122300 | 86041002 | 19421000006119 |
| 19441000006114 | Other pre-existing hypertension in preg/childb/puerp NOS | L122z | L122z00 | 86041002 | 19441000006114 |
| 305818015 | Pre-eclampsia or eclampsia with pre-existing hypertension | L127 | L127.00 | 198997005 | 305818015 |
| 212961000006118 | Pre-eclampsia or eclampsia + pre-existing hypertension NOS | L127z | L127z00 | 198997005 | 212961000006118 |
| 213111000006112 | Pre-exist hypertension compl preg childbirth and puerperium | L128 | L128.00 | 199005000 | 213111000006112 |
| 213091000006115 | Pre-exist hyperten heart dis compl preg childbth+puerperium | L1280 | L128000 | 199006004 | 213091000006115 |
| 213081000006118 | Pre-exist 2ndry hypertens comp preg childbth and puerperium | L1282 | L128200 | 199008003 | 213081000006118 |
| 331291010 | Adverse reaction to other antihypertensives | TJC7 | TJC7.00 | 293495006 | 469731000006119 |
| 331302012 | Adverse reaction to antihypertensives NOS | TJC7z | TJC7z00 | 293495006 | 464931000006118 |
| 404471000006113 | [X]Oth antihyperten drug caus advers eff in therap use, NEC | U60C5 | U60C500 | 293495006 | 404471000006113 |
| 356121000006111 | [X] Adverse reaction to other antihypertensives | U60C5-1 | U60C511 | 293495006 | 356121000006111 |
| 351481000006111 | [X] Adverse reaction to antihypertensives NOS | U60C5-A | U60C51A | 293495006 | 351481000006111 |
| 262960017 | Hypertension induced by oral contraceptive pill | 61462 | 6146200 | 169465000 | 262960017 |
| 53452019 | Secondary hypertension | G24 | G24..00 | 31992008 | 53452019 |
| 151161000006115 | Secondary malignant hypertension | G240 | G240.00 | 89242004 | 151161000006115 |
| 299676019 | Secondary malignant renovascular hypertension | G2400 | G240000 | 194783001 | 299676019 |
| 299677011 | Secondary malignant hypertension NOS | G240z | G240z00 | 89242004 | 151171000006110 |
| 299678018 | Secondary benign hypertension | G241 | G241.00 | 194785008 | 299678018 |
| 2478822013 | Secondary benign renovascular hypertension | G2410 | G241000 | 73410007 | 2478822013 |
| 299680012 | Secondary benign hypertension NOS | G241z | G241z00 | 194785008 | 158011000006111 |
| 299681011 | Hypertension secondary to endocrine disorders | G244 | G244.00 | 194788005 | 299681011 |
| 158241000006117 | Secondary hypertension NOS | G24z | G24z.00 | 31992008 | 158241000006117 |
| 299683014 | Secondary renovascular hypertension NOS | G24z0 | G24z000 | 123799005 | 152141000006112 |
| 299684015 | Hypertension secondary to drug | G24z1 | G24z100 | 194791005 | 299684015 |
| 299682016 | Secondary hypertension NOS | G24zz | G24zz00 | 31992008 | 158251000006115 |
| 300871017 | [X]Hypertension secondary to other renal disorders | Gyu21 | Gyu2100 | 31992008 | 389331000006110 |

Supplementary Table S22: Statin Prescription Codes

| ProdCodeId | DMDCode | Term from EMIS | Product Name | Drug substance name | Substance strength | formulation | Route of administration | Bnf code | Release |
| --- | --- | --- | --- | --- | --- | --- | --- | --- | --- |
| 2892041000033111 | 4172111000001108 | Crestor 40mg tablets (AstraZeneca UK Ltd) | Crestor 40mg tablets | Rosuvastatin calcium | 40.000mg | Tablet | Oral | 2120000 | 2120000 |
| 2891741000033115 | 408024009 | Rosuvastatin 40mg tablets | Rosuvastatin 40mg tablets | Rosuvastatin calcium | 40.000mg | Tablet | Oral | 2120000 | 2120000 |
| 2891841000033113 | 4171011000001104 | Crestor 10mg tablets (AstraZeneca UK Ltd) | Crestor 10mg tablets | Rosuvastatin calcium | 10.000mg | Tablet | Oral | 2120000 | 2120000 |
| 2891541000033111 | 408036003 | Rosuvastatin 10mg tablets | Rosuvastatin 10mg tablets | Rosuvastatin calcium | 10.000mg | Tablet | Oral | 2120000 | 2120000 |
| 2891641000033112 | 408037007 | Rosuvastatin 20mg tablets | Rosuvastatin 20mg tablets | Rosuvastatin calcium | 20.000mg | Tablet | Oral | 2120000 | 2120000 |
| 2891941000033117 | 4171311000001101 | Crestor 20mg tablets (AstraZeneca UK Ltd) | Crestor 20mg tablets | Rosuvastatin calcium | 20.000mg | Tablet | Oral | 2120000 | 2120000 |
| 3836041000033114 | 409108001 | Rosuvastatin 5mg tablets | Rosuvastatin 5mg tablets | Rosuvastatin calcium | 5.000mg | Tablet | Oral |  |  |
| 3836141000033113 | 9747511000001107 | Crestor 5mg tablets (AstraZeneca UK Ltd) | Crestor 5mg tablets | Rosuvastatin calcium | 5.000mg | Tablet | Oral |  |  |
| 1130141000033111 | 320012008 | Pravastatin 10mg tablets | Pravastatin 10mg tablets | Pravastatin sodium | 10.000mg | Tablet | Oral | 2120000 | 2120000 |
| 834841000033118 | 802411000001108 | Lipostat 10mg tablets (Bristol-Myers Squibb Pharmaceuticals Ltd) | Lipostat 10mg tablets | Pravastatin sodium | 10.000mg | Tablet | Oral | 2120000 | 2120000 |
| 834941000033114 | 454111000001107 | Lipostat 20mg tablets (Bristol-Myers Squibb Pharmaceuticals Ltd) | Lipostat 20mg tablets | Pravastatin sodium | 20.000mg | Tablet | Oral | 2120000 | 2120000 |
| 1130241000033116 | 320013003 | Pravastatin 20mg tablets | Pravastatin 20mg tablets | Pravastatin sodium | 20.000mg | Tablet | Oral | 2120000 | 2120000 |
| 1136541000033116 | 320014009 | Pravastatin 40mg tablets | Pravastatin 40mg tablets | Pravastatin sodium | 40.000mg | Tablet | Oral | 2120000 | 2120000 |
| 836541000033110 | 535011000001102 | Lipostat 40mg tablets (Bristol-Myers Squibb Pharmaceuticals Ltd) | Lipostat 40mg tablets | Pravastatin sodium | 40.000mg | Tablet | Oral | 2120000 | 2120000 |
| 8493841000033111 | 14957711000001105 | Pravastatin 5mg/5ml oral solution | Pravastatin 5mg/5ml oral solution | Pravastatin sodium | 1.000mg/1.000ml | Oral solution | Oral |  |  |
| 10618641000033118 | 14957811000001102 | Pravastatin 5mg/5ml oral suspension | Pravastatin 5mg/5ml oral suspension | Pravastatin sodium | 1.000mg/1.000ml | Oral suspension | Oral |  |  |
| 5711041000033117 | 15534511000001107 | Pravastatin 40mg/5ml oral suspension | Pravastatin 40mg/5ml oral suspension | Pravastatin sodium | 8.000mg/1.000ml | Oral suspension | Oral |  |  |
| 578641000033114 | 320022002 | Fluvastatin 20mg capsules | Fluvastatin 20mg capsules | Fluvastatin sodium | 20.000mg | Capsule | Oral | 2120000 | 2120000 |
| 819941000033119 | 84811000001104 | Lescol 20mg capsules (Novartis Pharmaceuticals UK Ltd) | Lescol 20mg capsules | Fluvastatin sodium | 20.000mg | Capsule | Oral | 2120000 | 2120000 |
| 820041000033116 | 409611000001108 | Lescol 40mg capsules (Novartis Pharmaceuticals UK Ltd) | Lescol 40mg capsules | Fluvastatin sodium | 40.000mg | Capsule | Oral | 2120000 | 2120000 |
| 578741000033117 | 320023007 | Fluvastatin 40mg capsules | Fluvastatin 40mg capsules | Fluvastatin sodium | 40.000mg | Capsule | Oral | 2120000 | 2120000 |
| 5007141000033112 | 15364211000001102 | Luvinsta XL 80mg tablets (Actavis UK Ltd) | Luvinsta XL 80mg tablets | Fluvastatin sodium | 80.000mg | Modified-release tablet | Oral |  |  |
| 5711841000033112 | 17282011000001107 | Dorisin XL 80mg tablets (Aspire Pharma Ltd) | Dorisin XL 80mg tablets | Fluvastatin sodium | 80.000mg | Modified-release tablet | Oral |  |  |
| 5808641000033119 | 17332011000001106 | Stefluvin XL 80mg tablets (Zentiva) | Stefluvin XL 80mg tablets | Fluvastatin sodium | 80.000mg | Modified-release tablet | Oral |  |  |
| 5560541000033119 | 16735311000001101 | Pinmactil 80mg modified-release tablets (Mylan) | Pinmactil 80mg modified-release tablets | Fluvastatin sodium | 80.000mg | Modified-release tablet | Oral |  |  |
| 2189141000033114 | 378111000001106 | Lescol XL 80mg tablets (Novartis Pharmaceuticals UK Ltd) | Lescol XL 80mg tablets | Fluvastatin sodium | 80.000mg | Modified-release tablet | Oral | 2120000 | 2120000 |
| 2189041000033110 | 36566411000001105 | Fluvastatin 80mg modified-release tablets | Fluvastatin 80mg modified-release tablets | Fluvastatin sodium | 80.000mg | Modified-release tablet | Oral | 2120000 | 2120000 |
| 12987241000033111 | 36836411000001100 | Cadaff XL 80mg tablets (Torrent Pharma (UK) Ltd) | Cadaff XL 80mg tablets | Fluvastatin sodium | 80.000mg | Modified-release tablet | Oral |  |  |
| 8962441000033117 | 23864311000001100 | Nandovar XL 80mg tablets (Sandoz Ltd) | Nandovar XL 80mg tablets | Fluvastatin sodium | 80.000mg | Modified-release tablet | Oral |  |  |
| 2290141000033116 | 4568511000001103 | Lipobay 800microgram tablets (Bayer Plc) | Lipobay 800microgram tablets | Cerivastatin sodium | 800.000microgram | Tablet | Oral |  |  |
| 2290041000033115 | 134491009 | Cerivastatin 800microgram tablets | Cerivastatin 800microgram tablets | Cerivastatin sodium | 800.000microgram | Tablet | Oral |  |  |
| 836641000033111 | 4535911000001106 | Lipobay 100microgram tablets (Bayer Plc) | Lipobay 100microgram tablets | Cerivastatin sodium | 100.000microgram | Tablet | Oral |  |  |
| 233741000033115 | 320035006 | Cerivastatin 100microgram tablets | Cerivastatin 100microgram tablets | Cerivastatin sodium | 100.000microgram | Tablet | Oral |  |  |
| 233841000033113 | 320036007 | Cerivastatin 200microgram tablets | Cerivastatin 200microgram tablets | Cerivastatin sodium | 200.000microgram | Tablet | Oral |  |  |
| 836741000033119 | 4537511000001108 | Lipobay 200microgram tablets (Bayer Plc) | Lipobay 200microgram tablets | Cerivastatin sodium | 200.000microgram | Tablet | Oral |  |  |
| 836841000033112 | 4566311000001105 | Lipobay 300microgram tablets (Bayer Plc) | Lipobay 300microgram tablets | Cerivastatin sodium | 300.000microgram | Tablet | Oral |  |  |
| 233941000033117 | 320037003 | Cerivastatin 300microgram tablets | Cerivastatin 300microgram tablets | Cerivastatin sodium | 300.000microgram | Tablet | Oral |  |  |
| 1916841000033114 | 320041004 | Cerivastatin 400microgram tablets | Cerivastatin 400microgram tablets | Cerivastatin sodium | 400.000microgram | Tablet | Oral |  |  |
| 1916741000033116 | 4538111000001103 | Lipobay 400microgram tablets (Bayer Plc) | Lipobay 400microgram tablets | Cerivastatin sodium | 400.000microgram | Tablet | Oral |  |  |
| 2261041000033114 | 134489001 | Atorvastatin 80mg tablets | Atorvastatin 80mg tablets | Atorvastatin calcium trihydrate | 80.000mg | Tablet | Oral | 2120000 | 2120000 |
| 2261141000033113 | 756111000001109 | Lipitor 80mg tablets (Upjohn UK Ltd) | Lipitor 80mg tablets | Atorvastatin calcium trihydrate | 80.000mg | Tablet | Oral | 2120000 | 2120000 |
| 91941000033117 | 320029006 | Atorvastatin 10mg tablets | Atorvastatin 10mg tablets | Atorvastatin calcium trihydrate | 10.000mg | Tablet | Oral | 2120000 | 2120000 |
| 836241000033113 | 643911000001108 | Lipitor 10mg tablets (Upjohn UK Ltd) | Lipitor 10mg tablets | Atorvastatin calcium trihydrate | 10.000mg | Tablet | Oral | 2120000 | 2120000 |
| 836341000033115 | 232011000001102 | Lipitor 20mg tablets (Upjohn UK Ltd) | Lipitor 20mg tablets | Atorvastatin calcium trihydrate | 20.000mg | Tablet | Oral | 2120000 | 2120000 |
| 92041000033111 | 320030001 | Atorvastatin 20mg tablets | Atorvastatin 20mg tablets | Atorvastatin calcium trihydrate | 20.000mg | Tablet | Oral | 2120000 | 2120000 |
| 92141000033110 | 320031002 | Atorvastatin 40mg tablets | Atorvastatin 40mg tablets | Atorvastatin calcium trihydrate | 40.000mg | Tablet | Oral | 2120000 | 2120000 |
| 836441000033114 | 484211000001108 | Lipitor 40mg tablets (Upjohn UK Ltd) | Lipitor 40mg tablets | Atorvastatin calcium trihydrate | 40.000mg | Tablet | Oral | 2120000 | 2120000 |
| 5990541000033112 | 14018211000001101 | Atorvastatin 20mg/5ml oral solution | Atorvastatin 20mg/5ml oral solution | Atorvastatin calcium trihydrate | 4.000mg/1.000ml | Oral solution | Oral |  |  |
| 3868741000033118 | 14018311000001109 | Atorvastatin 20mg/5ml oral suspension | Atorvastatin 20mg/5ml oral suspension | Atorvastatin calcium trihydrate | 4.000mg/1.000ml | Oral suspension | Oral |  |  |
| 5897841000033117 | 14158611000001100 | Atorvastatin 10mg/5ml oral solution | Atorvastatin 10mg/5ml oral solution | Atorvastatin calcium trihydrate | 2.000mg/1.000ml | Oral solution | Oral |  |  |
| 5897741000033110 | 14158711000001109 | Atorvastatin 10mg/5ml oral suspension | Atorvastatin 10mg/5ml oral suspension | Atorvastatin calcium trihydrate | 2.000mg/1.000ml | Oral suspension | Oral |  |  |
| 6469041000033110 | 19719311000001104 | Lipitor 10mg chewable tablets (Upjohn UK Ltd) | Lipitor 10mg chewable tablets | Atorvastatin calcium trihydrate | 10.000mg | Chewable tablet | Oral | 2120000 | 2120000 |
| 6468641000033113 | 19722411000001106 | Atorvastatin 10mg chewable tablets sugar free | Atorvastatin 10mg chewable tablets sugar free | Atorvastatin calcium trihydrate | 10.000mg | Chewable tablet | Oral | 2120000 | 2120000 |
| 6468841000033114 | 19722511000001105 | Atorvastatin 20mg chewable tablets sugar free | Atorvastatin 20mg chewable tablets sugar free | Atorvastatin calcium trihydrate | 20.000mg | Chewable tablet | Oral | 2120000 | 2120000 |
| 6469141000033114 | 19719611000001109 | Lipitor 20mg chewable tablets (Upjohn UK Ltd) | Lipitor 20mg chewable tablets | Atorvastatin calcium trihydrate | 20.000mg | Chewable tablet | Oral | 2120000 | 2120000 |
| 7861441000033110 | 20528511000001106 | Atorvastatin 30mg tablets | Atorvastatin 30mg tablets | Atorvastatin calcium trihydrate | 30.000mg | Tablet | Oral |  |  |
| 7861541000033111 | 20528611000001105 | Atorvastatin 60mg tablets | Atorvastatin 60mg tablets | Atorvastatin calcium trihydrate | 60.000mg | Tablet | Oral |  |  |
| 1336841000033110 | 319996000 | Simvastatin 10mg tablets | Simvastatin 10mg tablets | Simvastatin | 10.000mg | Tablet | Oral | 2120000 | 2120000 |
| 1562841000033119 | 108111000001106 | Zocor 10mg tablets (Merck Sharp & Dohme Ltd) | Zocor 10mg tablets | Simvastatin | 10.000mg | Tablet | Oral | 2120000 | 2120000 |
| 2973941000033116 | 4896211000001101 | Simvador 10mg tablets (Dexcel-Pharma Ltd) | Simvador 10mg tablets | Simvastatin | 10.000mg | Tablet | Oral | 2120000 | 2120000 |
| 3140841000033113 | 7630211000001106 | Ranzolont 10mg tablets (Ranbaxy (UK) Ltd) | Ranzolont 10mg tablets | Simvastatin | 10.000mg | Tablet | Oral |  |  |
| 3140941000033117 | 7631911000001109 | Ranzolont 20mg tablets (Ranbaxy (UK) Ltd) | Ranzolont 20mg tablets | Simvastatin | 20.000mg | Tablet | Oral |  |  |
| 2974041000033119 | 4896511000001103 | Simvador 20mg tablets (Dexcel-Pharma Ltd) | Simvador 20mg tablets | Simvastatin | 20.000mg | Tablet | Oral | 2120000 | 2120000 |
| 1562941000033110 | 776811000001104 | Zocor 20mg tablets (Merck Sharp & Dohme Ltd) | Zocor 20mg tablets | Simvastatin | 20.000mg | Tablet | Oral | 2120000 | 2120000 |
| 1336941000033119 | 319997009 | Simvastatin 20mg tablets | Simvastatin 20mg tablets | Simvastatin | 20.000mg | Tablet | Oral | 2120000 | 2120000 |
| 1337541000033111 | 320000009 | Simvastatin 40mg tablets | Simvastatin 40mg tablets | Simvastatin | 40.000mg | Tablet | Oral | 2120000 | 2120000 |
| 1562241000033118 | 859611000001107 | Zocor 40mg tablets (Merck Sharp & Dohme Ltd) | Zocor 40mg tablets | Simvastatin | 40.000mg | Tablet | Oral | 2120000 | 2120000 |
| 2974141000033115 | 4896711000001108 | Simvador 40mg tablets (Dexcel-Pharma Ltd) | Simvador 40mg tablets | Simvastatin | 40.000mg | Tablet | Oral | 2120000 | 2120000 |
| 3141041000033110 | 7632811000001108 | Ranzolont 40mg tablets (Ranbaxy (UK) Ltd) | Ranzolont 40mg tablets | Simvastatin | 40.000mg | Tablet | Oral |  |  |
| 2066341000033111 | 113211000001106 | Zocor 80mg tablets (Merck Sharp & Dohme Ltd) | Zocor 80mg tablets | Simvastatin | 80.000mg | Tablet | Oral | 2120000 | 2120000 |
| 2066241000033118 | 320006003 | Simvastatin 80mg tablets | Simvastatin 80mg tablets | Simvastatin | 80.000mg | Tablet | Oral | 2120000 | 2120000 |
| 4941641000033116 | 15158611000001106 | Simvador 80mg tablets (Dexcel-Pharma Ltd) | Simvador 80mg tablets | Simvastatin | 80.000mg | Tablet | Oral | 2120000 | 2120000 |
| 3304141000033110 | 8722111000001104 | Simvastatin 20mg/5ml oral suspension | Simvastatin 20mg/5ml oral suspension | Simvastatin | 4.000mg/1.000ml | Oral suspension | Oral |  |  |
| 5888441000033115 | 13894211000001107 | Simvastatin 20mg/5ml oral solution | Simvastatin 20mg/5ml oral solution | Simvastatin | 4.000mg/1.000ml | Oral solution | Oral |  |  |
| 5888541000033119 | 13894311000001104 | Simvastatin 40mg/5ml oral solution | Simvastatin 40mg/5ml oral solution | Simvastatin | 8.000mg/1.000ml | Oral solution | Oral |  |  |
| 4152341000033115 | 13894411000001106 | Simvastatin 40mg/5ml oral suspension | Simvastatin 40mg/5ml oral suspension | Simvastatin | 8.000mg/1.000ml | Oral suspension | Oral |  |  |
| 5817741000033114 | 17369311000001105 | Simvastatin 20mg/5ml oral suspension sugar free | Simvastatin 20mg/5ml oral suspension sugar free | Simvastatin | 4.000mg/1.000ml | Oral suspension | Oral |  |  |
| 5817841000033116 | 17429811000001102 | Simvastatin 40mg/5ml oral suspension sugar free | Simvastatin 40mg/5ml oral suspension sugar free | Simvastatin | 8.000mg/1.000ml | Oral suspension | Oral |  |  |

Supplementary Table S23: Antihypertensive Medication codes.

| ProdCodeId | DMD Code | Term from EMIS | Product Name | Drug substance name | Substance strength | Formulation | Route of administration | Bnf code |
| --- | --- | --- | --- | --- | --- | --- | --- | --- |
| "215341000033112" | "215111000001101" | "Catapres 100microgram tablets (Boehringer Ingelheim Ltd)" | "Catapres 100microgram tablets" | "Clonidine hydrochloride" | "100.000microgram" | "Tablet" | "Oral" | "2050200" |
| "285841000033118" | "318667005" | "Clonidine 100microgram tablets" | "Clonidine 100microgram tablets" | "Clonidine hydrochloride" | "100.000microgram" | "Tablet" | "Oral" | "6040101" |
| "287241000033110" | "318668000" | "Clonidine 300microgram tablets" | "Clonidine 300microgram tablets" | "Clonidine hydrochloride" | "300.000microgram" | "Tablet" | "Oral" | "6040101" |
| "215441000033118" | "368711000001103" | "Catapres 300microgram tablets (Boehringer Ingelheim Ltd)" | "Catapres 300microgram tablets" | "Clonidine hydrochloride" | "300.000microgram" | "Tablet" | "Oral" | "2050200" |
| "285941000033114" | "322840006" | "Clonidine 25microgram tablets" | "Clonidine 25microgram tablets" | "Clonidine hydrochloride" | "25.000microgram" | "Tablet" | "Oral" | "6040101" |
| "464341000033111" | "344511000001105" | "Dixarit 25microgram tablets (Boehringer Ingelheim Ltd)" | "Dixarit 25microgram tablets" | "Clonidine hydrochloride" | "25.000microgram" | "Tablet" | "Oral" | "4070402" |
| "5900141000033118" | "8398511000001100" | "Clonidine 50micrograms/5ml oral solution" | "Clonidine 50micrograms/5ml oral solution" | "Clonidine hydrochloride" | "10.000microgram/1.000ml" | "Oral solution" | "Oral" | "" |
| "5900241000033113" | "8398611000001101" | "Clonidine 50micrograms/5ml oral suspension" | "Clonidine 50micrograms/5ml oral suspension" | "Clonidine hydrochloride" | "10.000microgram/1.000ml" | "Oral suspension" | "Oral" | "" |
| "12569041000033115" | "11813011000001109" | "Clonidine 100micrograms/5ml oral suspension" | "Clonidine 100micrograms/5ml oral suspension" | "Clonidine hydrochloride" | "20.000microgram/1.000ml" | "Oral suspension" | "Oral" | "" |
| "196241000033111" | "364911000001108" | "Catapres 150micrograms/1ml solution for injection ampoules (Boehringer Ingelheim Ltd)" | "Catapres 150micrograms/1ml solution for injection ampoules" | "Clonidine hydrochloride" | "150.000microgram/1.000ml" | "Solution for injection" | "Intravenous" | "" |
| "275641000033110" | "36089211000001103" | "Clonidine 150micrograms/1ml solution for injection ampoules" | "Clonidine 150micrograms/1ml solution for injection ampoules" | "Clonidine hydrochloride" | "150.000microgram/1.000ml" | "Solution for injection" | "Intravenous" | "" |
| "261841000033118" | "36089511000001100" | "Clonidine 250microgram modified-release capsules" | "Clonidine 250microgram modified-release capsules" | "Clonidine hydrochloride" | "250.000microgram" | "Modified-release capsule" | "Oral" | "" |
| "12684041000033114" | "36392711000001102" | "Clonidine 50micrograms/5ml oral solution sugar free" | "Clonidine 50micrograms/5ml oral solution sugar free" | "Clonidine hydrochloride" | "10.000microgram/1.000ml" | "Oral solution" | "Oral" | "" |
| "216841000033113" | "41811000001109" | "Cardura 2mg tablets (Pfizer Ltd)" | "Cardura 2mg tablets" | "Doxazosin mesilate" | "2.000mg " | "Tablet" | "Oral" | "7040100" |
| "216741000033115" | "907711000001109" | "Cardura 1mg tablets (Pfizer Ltd)" | "Cardura 1mg tablets" | "Doxazosin mesilate" | "1.000mg " | "Tablet" | "Oral" | "7040100" |
| "2760141000033114" | "179311000001107" | "Cascor 4mg tablets (Ranbaxy (UK) Ltd)" | "Cascor 4mg tablets" | "Doxazosin mesilate" | "4.000mg " | "Tablet" | "Oral" | "" |
| "5403641000033117" | "11757711000001107" | "Colixil XL 4mg tablets (Sandoz Ltd)" | "Colixil XL 4mg tablets" | "Doxazosin mesilate" | "4.000mg " | "Modified-release tablet" | "Oral" | "" |
| "480841000033112" | "318783003" | "Doxazosin 4mg tablets" | "Doxazosin 4mg tablets" | "Doxazosin mesilate" | "4.000mg " | "Tablet" | "Oral" | "7040100" |
| "480641000033111" | "318781001" | "Doxazosin 1mg tablets" | "Doxazosin 1mg tablets" | "Doxazosin mesilate" | "1.000mg " | "Tablet" | "Oral" | "7040100" |
| "480741000033119" | "318782008" | "Doxazosin 2mg tablets" | "Doxazosin 2mg tablets" | "Doxazosin mesilate" | "2.000mg " | "Tablet" | "Oral" | "7040100" |
| "2274041000033118" | "123911000001106" | "Cardura XL 4mg tablets (Pfizer Ltd)" | "Cardura XL 4mg tablets" | "Doxazosin mesilate" | "4.000mg " | "Modified-release tablet" | "Oral" | "7040100" |
| "4063741000033112" | "11269911000001101" | "Doxadura XL 4mg tablets (Discovery Pharmaceuticals)" | "Doxadura XL 4mg tablets" | "Doxazosin mesilate" | "4.000mg " | "Modified-release tablet" | "Oral" | "" |
| "2274141000033119" | "873411000001109" | "Cardura XL 8mg tablets (Pfizer Ltd)" | "Cardura XL 8mg tablets" | "Doxazosin mesilate" | "8.000mg " | "Modified-release tablet" | "Oral" | "7040100" |
| "5138341000033111" | "8483511000001102" | "Doxazosin 4mg/5ml oral suspension" | "Doxazosin 4mg/5ml oral suspension" | "Doxazosin mesilate" | "800.000microgram/1.000ml " | "Oral suspension" | "Oral" | "" |
| "2958141000033112" | "4858111000001103" | "Doxadura 4mg tablets (Discovery Pharmaceuticals)" | "Doxadura 4mg tablets" | "Doxazosin mesilate" | "4.000mg " | "Tablet" | "Oral" | "7040100" |
| "5809641000033111" | "17338211000001104" | "Larbex XL 4mg tablets (Teva UK Ltd)" | "Larbex XL 4mg tablets" | "Doxazosin mesilate" | "4.000mg " | "Modified-release tablet" | "Oral" | "" |
| "2273841000033111" | "134456001" | "Doxazosin 4mg modified-release tablets" | "Doxazosin 4mg modified-release tablets" | "Doxazosin mesilate" | "4.000mg " | "Modified-release tablet" | "Oral" | "7040100" |
| "2760041000033110" | "904511000001107" | "Cascor 2mg tablets (Ranbaxy (UK) Ltd)" | "Cascor 2mg tablets" | "Doxazosin mesilate" | "2.000mg " | "Tablet" | "Oral" | "" |
| "4021141000033118" | "11098311000001104" | "Slocinx XL 4mg tablets (Zentiva)" | "Slocinx XL 4mg tablets" | "Doxazosin mesilate" | "4.000mg " | "Modified-release tablet" | "Oral" | "" |
| "4936841000033112" | "18197411000001107" | "Cardozin XL 4mg tablets (Almus Pharmaceuticals Ltd)" | "Cardozin XL 4mg tablets" | "Doxazosin mesilate" | "4.000mg " | "Modified-release tablet" | "Oral" | "" |
| "2273941000033115" | "135921004" | "Doxazosin 8mg modified-release tablets" | "Doxazosin 8mg modified-release tablets" | "Doxazosin mesilate" | "8.000mg " | "Modified-release tablet" | "Oral" | "7040100" |
| "2957941000033110" | "4857511000001108" | "Doxadura 1mg tablets (Discovery Pharmaceuticals)" | "Doxadura 1mg tablets" | "Doxazosin mesilate" | "1.000mg " | "Tablet" | "Oral" | "7040100" |
| "2958041000033113" | "4857711000001103" | "Doxadura 2mg tablets (Discovery Pharmaceuticals)" | "Doxadura 2mg tablets" | "Doxazosin mesilate" | "2.000mg " | "Tablet" | "Oral" | "7040100" |
| "4288341000033111" | "11812211000001105" | "Oxandosin XL 4mg tablets (Ratiopharm UK Ltd)" | "Oxandosin XL 4mg tablets" | "Doxazosin mesilate" | "4.000mg " | "Modified-release tablet" | "Oral" | "" |
| "6036141000033119" | "18164911000001108" | "Raporsin XL 4mg tablets (Actavis UK Ltd)" | "Raporsin XL 4mg tablets" | "Doxazosin mesilate" | "4.000mg " | "Modified-release tablet" | "Oral" | "" |
| "8870641000033119" | "23466611000001105" | "Doxazosin 4mg/5ml oral solution" | "Doxazosin 4mg/5ml oral solution" | "Doxazosin mesilate" | "800.000microgram/1.000ml " | "Oral solution" | "Oral" | "" |
| "4432941000033119" | "8483411000001101" | "Doxazosin 1mg/5ml oral suspension" | "Doxazosin 1mg/5ml oral suspension" | "Doxazosin mesilate" | "200.000microgram/1.000ml " | "Oral suspension" | "Oral" | "" |
| "4575941000033115" | "13811611000001108" | "Doxzogen XL 4mg tablets (Mylan)" | "Doxzogen XL 4mg tablets" | "Doxazosin mesilate" | "4.000mg " | "Modified-release tablet" | "Oral" | "" |
| "9173941000033111" | "24509711000001103" | "Doxazosin 1mg/5ml oral solution" | "Doxazosin 1mg/5ml oral solution" | "Doxazosin mesilate" | "200.000microgram/1.000ml " | "Oral solution" | "Oral" | "" |
| "816341000033112" | "701611000001102" | "Lasix 20mg tablets (Borg Medicare)" | "Lasix 20mg tablets" | "Furosemide" | "20.000mg" | "Tablet" | "Oral" | "2020200" |
| "3092241000033113" | "317971007" | "Furosemide 20mg tablets" | "Furosemide 20mg tablets" | "Furosemide" | "20.000mg" | "Tablet" | "Oral" | "2020200" |
| "3092341000033115" | "317972000" | "Furosemide 40mg tablets" | "Furosemide 40mg tablets" | "Furosemide" | "40.000mg" | "Tablet" | "Oral" | "2020200" |
| "615541000033119" | "714311000001107" | "Froop 40mg tablets (Ashbourne Pharmaceuticals Ltd)" | "Froop 40mg tablets" | "Furosemide" | "40.000mg" | "Tablet" | "Oral" | "2020200" |
| "816441000033118" | "79411000001107" | "Lasix 40mg tablets (Sanofi)" | "Lasix 40mg tablets" | "Furosemide" | "40.000mg" | "Tablet" | "Oral" | "2020200" |
| "615141000033111" | "99311000001108" | "Frusid 40mg tablets (Dr Reddy's Laboratories (UK) Ltd)" | "Frusid 40mg tablets" | "Furosemide" | "40.000mg" | "Tablet" | "Oral" | "" |
| "816541000033117" | "829911000001102" | "Lasix 500mg tablets (Sanofi)" | "Lasix 500mg tablets" | "Furosemide" | "500.000mg" | "Tablet" | "Oral" | "2020200" |
| "3092441000033114" | "317973005" | "Furosemide 500mg tablets" | "Furosemide 500mg tablets" | "Furosemide" | "500.000mg" | "Tablet" | "Oral" | "2020200" |
| "9808241000033110" | "27992311000001106" | "Diuresal 500mg tablets (Ennogen Pharma Ltd)" | "Diuresal 500mg tablets" | "Furosemide" | "500.000mg" | "Tablet" | "Oral" | "" |
| "3091941000033111" | "318006003" | "Furosemide 20mg/5ml oral solution sugar free" | "Furosemide 20mg/5ml oral solution sugar free" | "Furosemide" | "4.000mg/1.000ml" | "Oral solution" | "Oral" | "2020200" |
| "1731541000033115" | "602811000001100" | "Frusol 20mg/5ml oral solution (Rosemont Pharmaceuticals Ltd)" | "Frusol 20mg/5ml oral solution" | "Furosemide" | "4.000mg/1.000ml" | "Oral solution" | "Oral" | "2020200" |
| "1731641000033119" | "494811000001105" | "Frusol 40mg/5ml oral solution (Rosemont Pharmaceuticals Ltd)" | "Frusol 40mg/5ml oral solution" | "Furosemide" | "8.000mg/1.000ml" | "Oral solution" | "Oral/ Gastroenteral" | "2020200" |
| "3092041000033117" | "318007007" | "Furosemide 40mg/5ml oral solution sugar free" | "Furosemide 40mg/5ml oral solution sugar free" | "Furosemide" | "8.000mg/1.000ml" | "Oral solution" | "Oral/ Gastroenteral" | "2020200" |
| "812041000033114" | "701111000001105" | "Lasix 5mg/5ml oral solution (Borg Medicare)" | "Lasix 5mg/5ml oral solution" | "Furosemide" | "1.000mg/1.000ml" | "Oral solution" | "Oral" | "" |
| "3924941000033113" | "13893411000001108" | "Furosemide 5mg/5ml oral solution" | "Furosemide 5mg/5ml oral solution" | "Furosemide" | "1.000mg/1.000ml" | "Oral solution" | "Oral" | "" |
| "5993341000033112" | "13893511000001107" | "Furosemide 5mg/5ml oral suspension" | "Furosemide 5mg/5ml oral suspension" | "Furosemide" | "1.000mg/1.000ml" | "Oral suspension" | "Oral" | "" |
| "3954441000033119" | "34193711000001108" | "Furosemide 250mg/25ml solution for injection ampoules" | "Furosemide 250mg/25ml solution for injection ampoules" | "Furosemide" | "10.000mg/1.000ml" | "Solution for injection" | "Intravenous/ Intramuscular" | "" |
| "811841000033111" | "9611000001104" | "Lasix 20mg/2ml solution for injection ampoules (Sanofi)" | "Lasix 20mg/2ml solution for injection ampoules" | "Furosemide" | "10.000mg/1.000ml" | "Solution for injection" | "Intravenous/ Intramuscular" | "2020200" |
| "3091841000033115" | "36061411000001102" | "Furosemide 20mg/2ml solution for injection ampoules" | "Furosemide 20mg/2ml solution for injection ampoules" | "Furosemide" | "10.000mg/1.000ml" | "Solution for injection" | "Intravenous/ Intramuscular" | "2020200" |
| "3091741000033113" | "36061511000001103" | "Furosemide 50mg/5ml solution for injection ampoules" | "Furosemide 50mg/5ml solution for injection ampoules" | "Furosemide" | "10.000mg/1.000ml" | "Solution for injection" | "Intravenous/ Intramuscular" | "" |
| "3092641000033111" | "2898811000001106" | "Furosemide 80mg/8ml solution for injection Minijet pre-filled syringes (UCB Pharma Ltd)" | "Furosemide 80mg/8ml solution for injection Minijet pre-filled syringes" | "Furosemide" | "10.000mg/1.000ml" | "Solution for injection" | "Intravenous/ Intramuscular" | "" |
| "3092541000033110" | "36061611000001104" | "Furosemide 80mg/8ml solution for injection pre-filled syringes" | "Furosemide 80mg/8ml solution for injection pre-filled syringes" | "Furosemide" | "10.000mg/1.000ml" | "Solution for injection" | "Intravenous/ Intramuscular" | "" |
| "1731741000033111" | "855611000001109" | "Frusol 50mg/5ml oral solution (Rosemont Pharmaceuticals Ltd)" | "Frusol 50mg/5ml oral solution" | "Furosemide" | "10.000mg/1.000ml" | "Oral solution" | "Oral" | "2020200" |
| "3092141000033118" | "36564411000001102" | "Furosemide 50mg/5ml oral solution sugar free" | "Furosemide 50mg/5ml oral solution sugar free" | "Furosemide" | "10.000mg/1.000ml" | "Oral solution" | "Oral" | "2020200" |
| "741941000033113" | "318649003" | "Hydralazine 25mg tablets" | "Hydralazine 25mg tablets" | "Hydralazine hydrochloride" | "25.000mg" | "Tablet" | "Oral" | "2050100" |
| "74341000033114" | "657011000001101" | "Apresoline 25mg tablets (Advanz Pharma)" | "Apresoline 25mg tablets" | "Hydralazine hydrochloride" | "25.000mg" | "Tablet" | "Oral" | "2050100" |
| "742041000033119" | "318650003" | "Hydralazine 50mg tablets" | "Hydralazine 50mg tablets" | "Hydralazine hydrochloride" | "50.000mg" | "Tablet" | "Oral" | "2050100" |
| "12877141000033119" | "414426001" | "Hydralazine 10mg tablets" | "Hydralazine 10mg tablets" | "Hydralazine hydrochloride" | "10.000mg" | "Tablet" | "Oral" | "" |
| "12877241000033114" | "10637411000001100" | "Apo-Hydralazine 10mg tablets (Imported (United States))" | "Apo-Hydralazine 10mg tablets" | "Hydralazine hydrochloride" | "10.000mg" | "Tablet" | "" | "" |
| "4456241000033111" | "8528611000001105" | "Hydralazine 10mg/5ml oral suspension" | "Hydralazine 10mg/5ml oral suspension" | "Hydralazine hydrochloride" | "2.000mg/1.000ml" | "Oral suspension" | "Oral" | "" |
| "11072741000033116" | "8580911000001106" | "Hydralazine 25mg/5ml oral solution" | "Hydralazine 25mg/5ml oral solution" | "Hydralazine hydrochloride" | "5.000mg/1.000ml" | "Oral solution" | "Oral" | "" |
| "73441000033117" | "3925011000001100" | "Apresoline 20mg powder for solution for injection ampoules (Advanz Pharma)" | "Apresoline 20mg powder for solution for injection ampoules" | "Hydralazine hydrochloride" | "20.000mg" | "Powder for solution for injection" | "Intravenous" | "2050100" |
| "734141000033114" | "34193811000001100" | "Hydralazine 20mg powder for solution for injection ampoules" | "Hydralazine 20mg powder for solution for injection ampoules" | "Hydralazine hydrochloride" | "20.000mg" | "Powder for solution for injection" | "Intravenous" | "2050100" |
| "116741000033118" | "3689111000001107" | "Baratol 25mg tablets (Amdipharm Plc)" | "Baratol 25mg tablets" | "Indoramin hydrochloride" | "25.000mg " | "Tablet" | "Oral" | "" |
| "480541000033110" | "3354611000001100" | "Doralese Tiltab 20mg tablets (Chemidex Pharma Ltd)" | "Doralese Tiltab 20mg tablets" | "Indoramin hydrochloride" | "20.000mg " | "Tablet" | "Oral" | "7040100" |
| "769841000033111" | "318740009" | "Indoramin 25mg tablets" | "Indoramin 25mg tablets" | "Indoramin hydrochloride" | "25.000mg " | "Tablet" | "Oral" | "2050400" |
| "768541000033119" | "318739007" | "Indoramin 20mg tablets" | "Indoramin 20mg tablets" | "Indoramin hydrochloride" | "20.000mg " | "Tablet" | "Oral" | "7040100" |
| "40941000033118" | "253711000001107" | "Aldomet 250mg tablets (Aspen Pharma Trading Ltd)" | "Aldomet 250mg tablets" | "Methyldopa anhydrous" | "250.000mg " | "Tablet" | "Oral" | "2050200" |
| "41041000033111" | "73611000001108" | "Aldomet 500mg tablets (Aspen Pharma Trading Ltd)" | "Aldomet 500mg tablets" | "Methyldopa anhydrous" | "500.000mg " | "Tablet" | "Oral" | "2050200" |
| "897541000033115" | "318673006" | "Methyldopa 500mg tablets" | "Methyldopa 500mg tablets" | "Methyldopa anhydrous" | "500.000mg " | "Tablet" | "Oral" | "2050200" |
| "892341000033112" | "8667311000001100" | "Methyldopa 250mg/5ml oral suspension" | "Methyldopa 250mg/5ml oral suspension" | "Methyldopa anhydrous" | "50.000mg/1.000ml " | "Oral suspension" | "Oral" | "" |
| "897441000033116" | "318672001" | "Methyldopa 250mg tablets" | "Methyldopa 250mg tablets" | "Methyldopa anhydrous" | "250.000mg " | "Tablet" | "Oral" | "2050200" |
| "897341000033110" | "318671008" | "Methyldopa 125mg tablets" | "Methyldopa 125mg tablets" | "Methyldopa anhydrous" | "125.000mg " | "Tablet" | "Oral" | "2050200" |
| "920841000033118" | "318655008" | "Minoxidil 2.5mg tablets" | "Minoxidil 2.5mg tablets" | "Minoxidil" | "2.500mg" | "Tablet" | "Oral" | "2050100" |
| "850541000033113" | "3667011000001104" | "Loniten 2.5mg tablets (Pfizer Ltd)" | "Loniten 2.5mg tablets" | "Minoxidil" | "2.500mg" | "Tablet" | "Oral" | "2050100" |
| "850641000033114" | "3666411000001106" | "Loniten 5mg tablets (Pfizer Ltd)" | "Loniten 5mg tablets" | "Minoxidil" | "5.000mg" | "Tablet" | "Oral" | "2050100" |
| "920941000033114" | "318656009" | "Minoxidil 5mg tablets" | "Minoxidil 5mg tablets" | "Minoxidil" | "5.000mg" | "Tablet" | "Oral" | "2050100" |
| "920641000033119" | "318657000" | "Minoxidil 10mg tablets" | "Minoxidil 10mg tablets" | "Minoxidil" | "10.000mg" | "Tablet" | "Oral" | "2050100" |
| "850441000033112" | "3666711000001100" | "Loniten 10mg tablets (Pfizer Ltd)" | "Loniten 10mg tablets" | "Minoxidil" | "10.000mg" | "Tablet" | "Oral" | "2050100" |
| "6448341000033111" | "421143007" | "Minoxidil 5% foam" | "Minoxidil 5% foam" | "Minoxidil" | "50.000mg/1.000gram" | "Foam" | "Cutaneous" | "13090000" |
| "6448241000033118" | "18550611000001107" | "Regaine for Men Extra Strength 5% scalp foam (McNeil Products Ltd)" | "Regaine for Men Extra Strength 5% scalp foam" | "Minoxidil" | "50.000mg/1.000gram" | "Foam" | "Cutaneous" | "13090000" |
| "11803241000033110" | "32066411000001107" | "Regaine for Women Once a Day 5% scalp foam (McNeil Products Ltd)" | "Regaine for Women Once a Day 5% scalp foam" | "Minoxidil" | "50.000mg/1.000gram" | "Foam" | "Cutaneous" | "" |
| "4125241000033118" | "11472511000001105" | "Minoxidil 2% gel" | "Minoxidil 2% gel" | "Minoxidil" | "20.000mg/1.000gram" | "Gel" | "Cutaneous" | "" |
| "4125341000033111" | "11465411000001105" | "Regaine for Men 2% gel (McNeil Products Ltd)" | "Regaine for Men 2% gel" | "Minoxidil" | "20.000mg/1.000gram" | "Gel" | "Cutaneous" | "" |
| "4026741000033114" | "9524811000001104" | "Regaine for Men Regular Strength 2% solution (McNeil Products Ltd)" | "Regaine for Men Regular Strength 2% solution" | "Minoxidil" | "20.000mg/1.000ml" | "Liquid" | "Cutaneous" | "" |
| "4026341000033113" | "9524011000001105" | "Regaine for Women Regular Strength 2% solution (McNeil Products Ltd)" | "Regaine for Women Regular Strength 2% solution" | "Minoxidil" | "20.000mg/1.000ml" | "Liquid" | "Cutaneous" | "13090000" |
| "921941000033115" | "36036211000001103" | "Minoxidil 2% solution" | "Minoxidil 2% solution" | "Minoxidil" | "20.000mg/1.000ml" | "Liquid" | "Cutaneous" | "13090000" |
| "4026941000033112" | "9525311000001107" | "Regaine for Men Extra Strength 5% scalp solution (Johnson & Johnson Ltd)" | "Regaine for Men Extra Strength 5% scalp solution" | "Minoxidil" | "50.000mg/1.000ml" | "Liquid" | "Cutaneous" | "13090000" |
| "921841000033111" | "36036311000001106" | "Minoxidil 5% solution" | "Minoxidil 5% solution" | "Minoxidil" | "50.000mg/1.000ml" | "Liquid" | "Cutaneous" | "13090000" |
| "938541000033115" | "318707000" | "Moxonidine 200microgram tablets" | "Moxonidine 200microgram tablets" | "Moxonidine" | "200.000microgram" | "Tablet" | "Oral" | "2050200" |
| "1080841000033119" | "41111000001102" | "Physiotens 200microgram tablets (Mylan)" | "Physiotens 200microgram tablets" | "Moxonidine" | "200.000microgram" | "Tablet" | "Oral" | "2050200" |
| "1080941000033110" | "142811000001107" | "Physiotens 400microgram tablets (Mylan)" | "Physiotens 400microgram tablets" | "Moxonidine" | "400.000microgram" | "Tablet" | "Oral" | "2050200" |
| "938641000033119" | "318708005" | "Moxonidine 400microgram tablets" | "Moxonidine 400microgram tablets" | "Moxonidine" | "400.000microgram" | "Tablet" | "Oral" | "2050200" |
| "2635641000033111" | "408604009" | "Moxonidine 300microgram tablets" | "Moxonidine 300microgram tablets" | "Moxonidine" | "300.000microgram" | "Tablet" | "Oral" | "2050200" |
| "2635741000033119" | "522011000001109" | "Physiotens 300microgram tablets (Mylan)" | "Physiotens 300microgram tablets" | "Moxonidine" | "300.000microgram" | "Tablet" | "Oral" | "2050200" |
| "37841000033111" | "570011000001106" | "Alphavase 2 tablets (Ashbourne Pharmaceuticals Ltd)" | "Alphavase 2 tablets" | "Prazosin hydrochloride" | "2.000mg " | "Tablet" | "Oral" | "" |
| "37941000033115" | "74711000001102" | "Alphavase 5 tablets (Ashbourne Pharmaceuticals Ltd)" | "Alphavase 5 tablets" | "Prazosin hydrochloride" | "5.000mg " | "Tablet" | "Oral" | "" |
| "37741000033118" | "934411000001100" | "Alphavase 1 tablets (Ashbourne Pharmaceuticals Ltd)" | "Alphavase 1 tablets" | "Prazosin hydrochloride" | "1.000mg " | "Tablet" | "Oral" | "" |
| "743641000033117" | "347411000001101" | "Hypovase 1mg tablets (Pfizer Ltd)" | "Hypovase 1mg tablets" | "Prazosin hydrochloride" | "1.000mg " | "Tablet" | "Oral" | "7040100" |
| "1131341000033118" | "318767003" | "Prazosin 500microgram tablets" | "Prazosin 500microgram tablets" | "Prazosin hydrochloride" | "500.000microgram " | "Tablet" | "Oral" | "7040100" |
| "743541000033118" | "321311000001100" | "Hypovase 500microgram tablets (Pfizer Ltd)" | "Hypovase 500microgram tablets" | "Prazosin hydrochloride" | "500.000microgram " | "Tablet" | "Oral" | "7040100" |
| "743741000033114" | "150911000001104" | "Hypovase 2mg tablets (Pfizer Ltd)" | "Hypovase 2mg tablets" | "Prazosin hydrochloride" | "2.000mg " | "Tablet" | "Oral" | "7040100" |
| "1131641000033114" | "318770004" | "Prazosin 5mg tablets" | "Prazosin 5mg tablets" | "Prazosin hydrochloride" | "5.000mg " | "Tablet" | "Oral" | "2050400" |
| "1131441000033112" | "318768008" | "Prazosin 1mg tablets" | "Prazosin 1mg tablets" | "Prazosin hydrochloride" | "1.000mg " | "Tablet" | "Oral" | "7040100" |
| "1131541000033113" | "318769000" | "Prazosin 2mg tablets" | "Prazosin 2mg tablets" | "Prazosin hydrochloride" | "2.000mg " | "Tablet" | "Oral" | "7040100" |
| "11567441000033119" | "13078011000001104" | "Prazosin 500micrograms/5ml oral solution" | "Prazosin 500micrograms/5ml oral solution" | "Prazosin hydrochloride" | "100.000microgram/1.000ml " | "Oral solution" | "Oral" | "" |
| "745841000033115" | "3154311000001103" | "Hytrin 5mg tablets (AMCo)" | "Hytrin 5mg tablets" | "Terazosin hydrochloride" | "5.000mg " | "Tablet" | "Oral" | "7040100" |
| "745641000033116" | "3150911000001107" | "Hytrin 10mg tablets (AMCo)" | "Hytrin 10mg tablets" | "Terazosin hydrochloride" | "10.000mg " | "Tablet" | "Oral" | "7040100" |
| "745741000033113" | "3147611000001100" | "Hytrin 2mg tablets (AMCo)" | "Hytrin 2mg tablets" | "Terazosin hydrochloride" | "2.000mg " | "Tablet" | "Oral" | "7040100" |
| "11799841000033112" | "33630711000001107" | "Benph 2mg tablets (Mylan)" | "Benph 2mg tablets" | "Terazosin hydrochloride" | "2.000mg " | "Tablet" | "Oral" | "" |
| "1429141000033119" | "318776005" | "Terazosin 5mg tablets" | "Terazosin 5mg tablets" | "Terazosin hydrochloride" | "5.000mg " | "Tablet" | "Oral" | "7040100" |
| "1429041000033118" | "318777001" | "Terazosin 10mg tablets" | "Terazosin 10mg tablets" | "Terazosin hydrochloride" | "10.000mg " | "Tablet" | "Oral" | "7040100" |
| "1425341000033119" | "318779003" | "Terazosin 2mg tablets" | "Terazosin 2mg tablets" | "Terazosin hydrochloride" | "2.000mg " | "Tablet" | "Oral" | "7040100" |
| "11807841000033119" | "33628611000001102" | "Benph 5mg tablets (Mylan)" | "Benph 5mg tablets" | "Terazosin hydrochloride" | "5.000mg " | "Tablet" | "Oral" | "" |
| "3038041000033111" | "319283006" | "Amlodipine 5mg tablets" | "Amlodipine 5mg tablets" | "Amlodipine" | "5.000mg" | "Tablet" | "Oral" | "2060200" |
| "3038141000033110" | "319284000" | "Amlodipine 10mg tablets" | "Amlodipine 10mg tablets" | "Amlodipine" | "10.000mg" | "Tablet" | "Oral" | "2060200" |
| "13422241000033114" | "429828006" | "Amlodipine 2.5mg tablets" | "Amlodipine 2.5mg tablets" | "Amlodipine" | "2.500mg" | "Tablet" | "Oral" | "" |
| "787941000033112" | "172711000001100" | "Istin 5mg tablets (Upjohn UK Ltd)" | "Istin 5mg tablets" | "Amlodipine" | "5.000mg" | "Tablet" | "Oral" | "2060200" |
| "788041000033110" | "408111000001107" | "Istin 10mg tablets (Upjohn UK Ltd)" | "Istin 10mg tablets" | "Amlodipine" | "10.000mg" | "Tablet" | "Oral" | "2060200" |
| "3188741000033111" | "8046211000001107" | "Amlostin 5mg tablets (Discovery Pharmaceuticals)" | "Amlostin 5mg tablets" | "Amlodipine" | "5.000mg" | "Tablet" | "Oral" | "2060200" |
| "3188841000033118" | "8046411000001106" | "Amlostin 10mg tablets (Discovery Pharmaceuticals)" | "Amlostin 10mg tablets" | "Amlodipine" | "10.000mg" | "Tablet" | "Oral" | "2060200" |
| "3963741000033115" | "8278111000001105" | "Amlodipine 10mg/5ml oral suspension" | "Amlodipine 10mg/5ml oral suspension" | "Amlodipine" | "2.000mg/1.000ml" | "Oral suspension" | "Oral" | "" |
| "5888741000033110" | "8278311000001107" | "Amlodipine 5mg/5ml oral suspension" | "Amlodipine 5mg/5ml oral suspension" | "Amlodipine" | "1.000mg/1.000ml" | "Oral suspension" | "Oral" | "" |
| "5490241000033113" | "13892511000001100" | "Amlodipine 5mg/5ml oral solution" | "Amlodipine 5mg/5ml oral solution" | "Amlodipine" | "1.000mg/1.000ml" | "Oral solution" | "Oral" | "" |
| "4897741000033118" | "15773511000001108" | "Amlodipine 1.5mg/5ml oral suspension" | "Amlodipine 1.5mg/5ml oral suspension" | "Amlodipine" | "300.000microgram/1.000ml" | "Oral suspension" | "Oral" | "" |
| "7740641000033112" | "20478011000001105" | "Amlodipine 10mg/5ml oral solution" | "Amlodipine 10mg/5ml oral solution" | "Amlodipine" | "2.000mg/1.000ml" | "Oral solution" | "Oral" | "" |
| "10387441000033111" | "29826211000001109" | "Amlodipine 10mg/5ml oral solution sugar free" | "Amlodipine 10mg/5ml oral solution sugar free" | "Amlodipine" | "2.000mg/1.000ml" | "Oral solution" | "Oral" | "" |
| "4508041000033119" | "29826311000001101" | "Amlodipine 5mg/5ml oral solution sugar free" | "Amlodipine 5mg/5ml oral solution sugar free" | "Amlodipine" | "1.000mg/1.000ml" | "Oral solution" | "Oral" | "" |
| "12682241000033110" | "36237311000001101" | "Amlodipine 5mg/5ml oral suspension sugar free" | "Amlodipine 5mg/5ml oral suspension sugar free" | "Amlodipine" | "1.000mg/1.000ml" | "Oral suspension" | "Oral" | "" |
| "452841000033118" | "5711000001106" | "Dilzem SR 120 capsules (Teva UK Ltd)" | "Dilzem SR 120 capsules" | "Diltiazem hydrochloride" | "120.000mg" | "Modified-release capsule" | "Oral" | "2060200" |
| "1520141000033118" | "32311000001105" | "Viazem XL 120mg capsules (Thornton & Ross Ltd)" | "Viazem XL 120mg capsules" | "Diltiazem hydrochloride" | "120.000mg" | "Modified-release capsule" | "Oral" | "2060200" |
| "1520241000033113" | "33211000001108" | "Viazem XL 180mg capsules (Thornton & Ross Ltd)" | "Viazem XL 180mg capsules" | "Diltiazem hydrochloride" | "180.000mg" | "Modified-release capsule" | "Oral" | "2060200" |
| "2676041000033111" | "34711000001104" | "Disogram SR 300mg capsules (Ranbaxy (UK) Ltd)" | "Disogram SR 300mg capsules" | "Diltiazem hydrochloride" | "300.000mg" | "Modified-release capsule" | "Oral" | "2060200" |
| "1570441000033110" | "75111000001104" | "Angitil XL 240 capsules (Ethypharm UK Ltd)" | "Angitil XL 240 capsules" | "Diltiazem hydrochloride" | "240.000mg" | "Modified-release capsule" | "Oral" | "2060200" |
| "1442841000033115" | "103611000001105" | "Tildiem Retard 90mg tablets (Sanofi)" | "Tildiem Retard 90mg tablets" | "Diltiazem hydrochloride" | "90.000mg" | "Modified-release tablet" | "Oral" | "2060200" |
| "199241000033119" | "104111000001100" | "Calcicard CR 120mg tablets (Teva UK Ltd)" | "Calcicard CR 120mg tablets" | "Diltiazem hydrochloride" | "120.000mg" | "Modified-release tablet" | "Oral" | "" |
| "1833041000033110" | "105211000001107" | "Zemtard 120 XL capsules (Galen Ltd)" | "Zemtard 120 XL capsules" | "Diltiazem hydrochloride" | "120.000mg" | "Modified-release capsule" | "Oral" | "2060200" |
| "2675641000033113" | "108711000001107" | "Disogram SR 240mg capsules (Ranbaxy (UK) Ltd)" | "Disogram SR 240mg capsules" | "Diltiazem hydrochloride" | "240.000mg" | "Modified-release capsule" | "Oral" | "2060200" |
| "2675041000033119" | "116711000001106" | "Disogram SR 120mg capsules (Ranbaxy (UK) Ltd)" | "Disogram SR 120mg capsules" | "Diltiazem hydrochloride" | "120.000mg" | "Modified-release capsule" | "Oral" | "2060200" |
| "1346541000033112" | "119211000001108" | "Slozem 180mg capsules (Zentiva)" | "Slozem 180mg capsules" | "Diltiazem hydrochloride" | "180.000mg" | "Modified-release capsule" | "Oral" | "2060200" |
| "1346441000033111" | "130211000001109" | "Slozem 120mg capsules (Zentiva)" | "Slozem 120mg capsules" | "Diltiazem hydrochloride" | "120.000mg" | "Modified-release capsule" | "Oral" | "2060200" |
| "66041000033110" | "144811000001101" | "Angiozem CR 120mg tablets (Ashbourne Pharmaceuticals Ltd)" | "Angiozem CR 120mg tablets" | "Diltiazem hydrochloride" | "120.000mg" | "Modified-release tablet" | "Oral" | "2060200" |
| "2739141000033114" | "157511000001105" | "Horizem SR 90mg capsules (Horizon lifecare)" | "Horizem SR 90mg capsules" | "Diltiazem hydrochloride" | "90.000mg" | "Modified-release capsule" | "Oral" | "" |
| "5816941000033110" | "158411000001105" | "Bi-Carzem XL 300mg capsules (Tillomed Laboratories Ltd)" | "Bi-Carzem XL 300mg capsules" | "Diltiazem hydrochloride" | "300.000mg" | "Modified-release capsule" | "Oral" | "" |
| "2952341000033113" | "200811000001104" | "Zildil 90mg modified-release capsules (Healthcare Pharma Ltd)" | "Zildil 90mg modified-release capsules" | "Diltiazem hydrochloride" | "90.000mg" | "Modified-release capsule" | "Oral" | "" |
| "199341000033112" | "219611000001107" | "Calcicard CR 90mg tablets (Teva UK Ltd)" | "Calcicard CR 90mg tablets" | "Diltiazem hydrochloride" | "90.000mg" | "Modified-release tablet" | "Oral" | "" |
| "453041000033116" | "243111000001108" | "Dilzem XL 120 capsules (Teva UK Ltd)" | "Dilzem XL 120 capsules" | "Diltiazem hydrochloride" | "120.000mg" | "Modified-release capsule" | "Oral" | "2060200" |
| "453141000033117" | "254911000001106" | "Dilzem XL 180 capsules (Teva UK Ltd)" | "Dilzem XL 180 capsules" | "Diltiazem hydrochloride" | "180.000mg" | "Modified-release capsule" | "Oral" | "2060200" |
| "2674941000033119" | "256611000001109" | "Disogram SR 90mg capsules (Ranbaxy (UK) Ltd)" | "Disogram SR 90mg capsules" | "Diltiazem hydrochloride" | "90.000mg" | "Modified-release capsule" | "Oral" | "2060200" |
| "1441841000033112" | "261611000001107" | "Tildiem LA 200 capsules (Sanofi)" | "Tildiem LA 200 capsules" | "Diltiazem hydrochloride" | "200.000mg" | "Modified-release capsule" | "Oral" | "2060200" |
| "65441000033110" | "298811000001103" | "Angitil SR 120 capsules (Ethypharm UK Ltd)" | "Angitil SR 120 capsules" | "Diltiazem hydrochloride" | "120.000mg" | "Modified-release capsule" | "Oral" | "2060200" |
| "65541000033111" | "336611000001101" | "Angitil SR 180 capsules (Ethypharm UK Ltd)" | "Angitil SR 180 capsules" | "Diltiazem hydrochloride" | "180.000mg" | "Modified-release capsule" | "Oral" | "2060200" |
| "1833241000033119" | "345411000001107" | "Zemtard 240 XL capsules (Galen Ltd)" | "Zemtard 240 XL capsules" | "Diltiazem hydrochloride" | "240.000mg" | "Modified-release capsule" | "Oral" | "2060200" |
| "1602541000033111" | "353011000001101" | "Dilcardia SR 90mg capsules (Mylan)" | "Dilcardia SR 90mg capsules" | "Diltiazem hydrochloride" | "90.000mg" | "Modified-release capsule" | "Oral" | "2060200" |
| "1442741000033113" | "383911000001109" | "Tildiem Retard 120mg tablets (Sanofi)" | "Tildiem Retard 120mg tablets" | "Diltiazem hydrochloride" | "120.000mg" | "Modified-release tablet" | "Oral" | "2060200" |
| "1520441000033114" | "407011000001105" | "Viazem XL 300mg capsules (Thornton & Ross Ltd)" | "Viazem XL 300mg capsules" | "Diltiazem hydrochloride" | "300.000mg" | "Modified-release capsule" | "Oral" | "2060200" |
| "452641000033119" | "417111000001109" | "Dilzem SR 60 capsules (Teva UK Ltd)" | "Dilzem SR 60 capsules" | "Diltiazem hydrochloride" | "60.000mg" | "Modified-release capsule" | "Oral" | "2060200" |
| "65941000033117" | "440711000001104" | "Angiozem CR 90mg tablets (Ashbourne Pharmaceuticals Ltd)" | "Angiozem CR 90mg tablets" | "Diltiazem hydrochloride" | "90.000mg" | "Modified-release tablet" | "Oral" | "2060200" |
| "1570541000033111" | "467111000001105" | "Angitil XL 300 capsules (Ethypharm UK Ltd)" | "Angitil XL 300 capsules" | "Diltiazem hydrochloride" | "300.000mg" | "Modified-release capsule" | "Oral" | "2060200" |
| "2674841000033110" | "469811000001106" | "Disogram SR 60mg capsules (Ranbaxy (UK) Ltd)" | "Disogram SR 60mg capsules" | "Diltiazem hydrochloride" | "60.000mg" | "Modified-release capsule" | "Oral" | "2060200" |
| "1661641000033116" | "527711000001108" | "Optil 60mg modified-release tablets (Opus Pharmaceuticals Ltd)" | "Optil 60mg modified-release tablets" | "Diltiazem hydrochloride" | "60.000mg" | "Modified-release tablet" | "Oral" | "2060200" |
| "2779141000033112" | "540011000001107" | "Bi-Carzem SR 60mg capsules (Tillomed Laboratories Ltd)" | "Bi-Carzem SR 60mg capsules" | "Diltiazem hydrochloride" | "60.000mg" | "Modified-release capsule" | "Oral" | "" |
| "2261241000033118" | "550211000001104" | "Slozem 300mg capsules (Zentiva)" | "Slozem 300mg capsules" | "Diltiazem hydrochloride" | "300.000mg" | "Modified-release capsule" | "Oral" | "2060200" |
| "2779341000033110" | "580711000001101" | "Bi-Carzem SR 120mg capsules (Tillomed Laboratories Ltd)" | "Bi-Carzem SR 120mg capsules" | "Diltiazem hydrochloride" | "120.000mg" | "Modified-release capsule" | "Oral" | "" |
| "1346641000033113" | "599811000001104" | "Slozem 240mg capsules (Zentiva)" | "Slozem 240mg capsules" | "Diltiazem hydrochloride" | "240.000mg" | "Modified-release capsule" | "Oral" | "2060200" |
| "2955341000033118" | "640311000001101" | "Zemret 300 XL capsules (Tillomed Laboratories Ltd)" | "Zemret 300 XL capsules" | "Diltiazem hydrochloride" | "300.000mg" | "Modified-release capsule" | "Oral" | "" |
| "1520541000033110" | "648211000001107" | "Viazem XL 360mg capsules (Thornton & Ross Ltd)" | "Viazem XL 360mg capsules" | "Diltiazem hydrochloride" | "360.000mg" | "Modified-release capsule" | "Oral" | "2060200" |
| "2952441000033119" | "655411000001104" | "Zildil SR 120mg capsules (Healthcare Pharma Ltd)" | "Zildil SR 120mg capsules" | "Diltiazem hydrochloride" | "120.000mg" | "Modified-release capsule" | "Oral" | "" |
| "71141000033114" | "672311000001103" | "Angiozem 60mg modified-release tablets (Ashbourne Pharmaceuticals Ltd)" | "Angiozem 60mg modified-release tablets" | "Diltiazem hydrochloride" | "60.000mg" | "Modified-release tablet" | "Oral" | "" |
| "452741000033111" | "682311000001105" | "Dilzem SR 90 capsules (Teva UK Ltd)" | "Dilzem SR 90 capsules" | "Diltiazem hydrochloride" | "90.000mg" | "Modified-release capsule" | "Oral" | "2060200" |
| "2675341000033117" | "713411000001103" | "Disogram SR 180mg capsules (Ranbaxy (UK) Ltd)" | "Disogram SR 180mg capsules" | "Diltiazem hydrochloride" | "180.000mg" | "Modified-release capsule" | "Oral" | "2060200" |
| "2952241000033115" | "721811000001108" | "Zildil SR 60mg capsules (Healthcare Pharma Ltd)" | "Zildil SR 60mg capsules" | "Diltiazem hydrochloride" | "60.000mg" | "Modified-release capsule" | "Oral" | "" |
| "1602341000033116" | "723811000001107" | "Dilcardia SR 120mg capsules (Mylan)" | "Dilcardia SR 120mg capsules" | "Diltiazem hydrochloride" | "120.000mg" | "Modified-release capsule" | "Oral" | "2060200" |
| "453241000033112" | "733511000001107" | "Dilzem XL 240 capsules (Teva UK Ltd)" | "Dilzem XL 240 capsules" | "Diltiazem hydrochloride" | "240.000mg" | "Modified-release capsule" | "Oral" | "2060200" |
| "2739041000033110" | "758511000001104" | "Horizem SR 120mg capsules (Horizon lifecare)" | "Horizem SR 120mg capsules" | "Diltiazem hydrochloride" | "120.000mg" | "Modified-release capsule" | "Oral" | "" |
| "1443841000033113" | "762011000001102" | "Tildiem 60mg modified-release tablets (Sanofi)" | "Tildiem 60mg modified-release tablets" | "Diltiazem hydrochloride" | "60.000mg" | "Modified-release tablet" | "Oral" | "2060200" |
| "2779241000033117" | "764511000001100" | "Bi-Carzem SR 90mg capsules (Tillomed Laboratories Ltd)" | "Bi-Carzem SR 90mg capsules" | "Diltiazem hydrochloride" | "90.000mg" | "Modified-release capsule" | "Oral" | "" |
| "1833141000033114" | "813611000001103" | "Zemtard 180 XL capsules (Galen Ltd)" | "Zemtard 180 XL capsules" | "Diltiazem hydrochloride" | "180.000mg" | "Modified-release capsule" | "Oral" | "2060200" |
| "2955241000033111" | "823511000001107" | "Zemret 240 XL capsules (Tillomed Laboratories Ltd)" | "Zemret 240 XL capsules" | "Diltiazem hydrochloride" | "240.000mg" | "Modified-release capsule" | "Oral" | "" |
| "65341000033116" | "857011000001109" | "Angitil SR 90 capsules (Ethypharm UK Ltd)" | "Angitil SR 90 capsules" | "Diltiazem hydrochloride" | "90.000mg" | "Modified-release capsule" | "Oral" | "2060200" |
| "4590741000033116" | "865711000001107" | "Retalzem 60 modified-release tablets (Kent Pharmaceuticals Ltd)" | "Retalzem 60 modified-release tablets" | "Diltiazem hydrochloride" | "60.000mg" | "Modified-release tablet" | "Oral" | "" |
| "1833341000033112" | "866811000001107" | "Zemtard 300 XL capsules (Galen Ltd)" | "Zemtard 300 XL capsules" | "Diltiazem hydrochloride" | "300.000mg" | "Modified-release capsule" | "Oral" | "2060200" |
| "5816841000033119" | "884811000001103" | "Bi-Carzem XL 240mg capsules (Tillomed Laboratories Ltd)" | "Bi-Carzem XL 240mg capsules" | "Diltiazem hydrochloride" | "240.000mg" | "Modified-release capsule" | "Oral" | "" |
| "1520341000033115" | "886511000001107" | "Viazem XL 240mg capsules (Thornton & Ross Ltd)" | "Viazem XL 240mg capsules" | "Diltiazem hydrochloride" | "240.000mg" | "Modified-release capsule" | "Oral" | "2060200" |
| "1441741000033119" | "893111000001107" | "Tildiem LA 300 capsules (Sanofi)" | "Tildiem LA 300 capsules" | "Diltiazem hydrochloride" | "300.000mg" | "Modified-release capsule" | "Oral" | "2060200" |
| "2955141000033116" | "924711000001109" | "Zemret 180 XL capsules (Tillomed Laboratories Ltd)" | "Zemret 180 XL capsules" | "Diltiazem hydrochloride" | "180.000mg" | "Modified-release capsule" | "Oral" | "" |
| "1602441000033110" | "937011000001101" | "Dilcardia SR 60mg capsules (Mylan)" | "Dilcardia SR 60mg capsules" | "Diltiazem hydrochloride" | "60.000mg" | "Modified-release capsule" | "Oral" | "2060200" |
| "19641000033110" | "2885611000001102" | "Adizem-SR 120mg tablets (Napp Pharmaceuticals Ltd)" | "Adizem-SR 120mg tablets" | "Diltiazem hydrochloride" | "120.000mg" | "Modified-release tablet" | "Oral" | "2060200" |
| "18041000033112" | "2886111000001104" | "Adizem-XL 300mg capsules (Napp Pharmaceuticals Ltd)" | "Adizem-XL 300mg capsules" | "Diltiazem hydrochloride" | "300.000mg" | "Modified-release capsule" | "Oral" | "2060200" |
| "17941000033114" | "2886311000001102" | "Adizem-XL 240mg capsules (Napp Pharmaceuticals Ltd)" | "Adizem-XL 240mg capsules" | "Diltiazem hydrochloride" | "240.000mg" | "Modified-release capsule" | "Oral" | "2060200" |
| "2653341000033118" | "2886511000001108" | "Adizem-XL 200mg capsules (Napp Pharmaceuticals Ltd)" | "Adizem-XL 200mg capsules" | "Diltiazem hydrochloride" | "200.000mg" | "Modified-release capsule" | "Oral" | "2060200" |
| "17441000033116" | "2886711000001103" | "Adizem-SR 180mg capsules (Napp Pharmaceuticals Ltd)" | "Adizem-SR 180mg capsules" | "Diltiazem hydrochloride" | "180.000mg" | "Modified-release capsule" | "Oral" | "2060200" |
| "17641000033119" | "2887011000001102" | "Adizem-SR 90mg capsules (Napp Pharmaceuticals Ltd)" | "Adizem-SR 90mg capsules" | "Diltiazem hydrochloride" | "90.000mg" | "Modified-release capsule" | "Oral" | "2060200" |
| "17341000033110" | "2887311000001104" | "Adizem-SR 120mg capsules (Napp Pharmaceuticals Ltd)" | "Adizem-SR 120mg capsules" | "Diltiazem hydrochloride" | "120.000mg" | "Modified-release capsule" | "Oral" | "2060200" |
| "17741000033111" | "2937811000001108" | "Adizem-XL 120mg capsules (Napp Pharmaceuticals Ltd)" | "Adizem-XL 120mg capsules" | "Diltiazem hydrochloride" | "120.000mg" | "Modified-release capsule" | "Oral" | "2060200" |
| "17841000033118" | "2938011000001101" | "Adizem-XL 180mg capsules (Napp Pharmaceuticals Ltd)" | "Adizem-XL 180mg capsules" | "Diltiazem hydrochloride" | "180.000mg" | "Modified-release capsule" | "Oral" | "2060200" |
| "4953741000033114" | "8456911000001108" | "Diltiazem 60mg/5ml oral suspension" | "Diltiazem 60mg/5ml oral suspension" | "Diltiazem hydrochloride" | "12.000mg/1.000ml" | "Oral suspension" | "Oral" | "" |
| "5991941000033112" | "8457011000001107" | "Diltiazem 60mg/5ml oral solution" | "Diltiazem 60mg/5ml oral solution" | "Diltiazem hydrochloride" | "12.000mg/1.000ml" | "Oral solution" | "Oral" | "" |
| "2920141000033113" | "8793111000001101" | "Diltiazem 2% cream" | "Diltiazem 2% cream" | "Diltiazem hydrochloride" | "20.000mg/1.000gram" | "Cream" | "Transdermal" | "" |
| "5991841000033116" | "8793311000001104" | "Diltiazem 2% ointment" | "Diltiazem 2% ointment" | "Diltiazem hydrochloride" | "20.000mg/1.000gram" | "Ointment" | "Transdermal" | "" |
| "9118241000033117" | "8885711000001100" | "Kenzem SR 60mg capsules (Kent Pharmaceuticals Ltd)" | "Kenzem SR 60mg capsules" | "Diltiazem hydrochloride" | "60.000mg" | "Modified-release capsule" | "Oral" | "" |
| "9118341000033110" | "8886211000001101" | "Kenzem SR 90mg capsules (Kent Pharmaceuticals Ltd)" | "Kenzem SR 90mg capsules" | "Diltiazem hydrochloride" | "90.000mg" | "Modified-release capsule" | "Oral" | "" |
| "9118441000033116" | "8886511000001103" | "Kenzem SR 120mg capsules (Kent Pharmaceuticals Ltd)" | "Kenzem SR 120mg capsules" | "Diltiazem hydrochloride" | "120.000mg" | "Modified-release capsule" | "Oral" | "" |
| "452941000033114" | "39023011000001107" | "Diltiazem 60mg modified-release capsules" | "Diltiazem 60mg modified-release capsules" | "Diltiazem hydrochloride" | "60.000mg" | "Modified-release capsule" | "Oral" | "2060200" |
| "452141000033112" | "39023111000001108" | "Diltiazem 90mg modified-release capsules" | "Diltiazem 90mg modified-release capsules" | "Diltiazem hydrochloride" | "90.000mg" | "Modified-release capsule" | "Oral" | "2060200" |
| "451841000033110" | "39023211000001102" | "Diltiazem 120mg modified-release capsules" | "Diltiazem 120mg modified-release capsules" | "Diltiazem hydrochloride" | "120.000mg" | "Modified-release capsule" | "Oral" | "2060200" |
| "451941000033119" | "39023311000001105" | "Diltiazem 180mg modified-release capsules" | "Diltiazem 180mg modified-release capsules" | "Diltiazem hydrochloride" | "180.000mg" | "Modified-release capsule" | "Oral" | "2060200" |
| "453941000033115" | "39023411000001103" | "Diltiazem 200mg modified-release capsules" | "Diltiazem 200mg modified-release capsules" | "Diltiazem hydrochloride" | "200.000mg" | "Modified-release capsule" | "Oral" | "2060200" |
| "453741000033118" | "39023511000001104" | "Diltiazem 240mg modified-release capsules" | "Diltiazem 240mg modified-release capsules" | "Diltiazem hydrochloride" | "240.000mg" | "Modified-release capsule" | "Oral" | "2060200" |
| "452041000033113" | "39023611000001100" | "Diltiazem 300mg modified-release capsules" | "Diltiazem 300mg modified-release capsules" | "Diltiazem hydrochloride" | "300.000mg" | "Modified-release capsule" | "Oral" | "2060200" |
| "1800341000033113" | "39023711000001109" | "Diltiazem 360mg modified-release capsules" | "Diltiazem 360mg modified-release capsules" | "Diltiazem hydrochloride" | "360.000mg" | "Modified-release capsule" | "Oral" | "2060200" |
| "454041000033118" | "39023811000001101" | "Diltiazem 60mg modified-release tablets" | "Diltiazem 60mg modified-release tablets" | "Diltiazem hydrochloride" | "60.000mg" | "Modified-release tablet" | "Oral" | "2060200" |
| "458841000033115" | "39023911000001106" | "Diltiazem 90mg modified-release tablets" | "Diltiazem 90mg modified-release tablets" | "Diltiazem hydrochloride" | "90.000mg" | "Modified-release tablet" | "Oral" | "2060200" |
| "459641000033113" | "39024011000001109" | "Diltiazem 120mg modified-release tablets" | "Diltiazem 120mg modified-release tablets" | "Diltiazem hydrochloride" | "120.000mg" | "Modified-release tablet" | "Oral" | "2060200" |
| "1094041000033119" | "48511000001101" | "Plendil 10mg modified-release tablets (AstraZeneca UK Ltd)" | "Plendil 10mg modified-release tablets" | "Felodipine" | "10.000mg" | "Modified-release tablet" | "Oral" | "2060200" |
| "1094141000033115" | "490211000001101" | "Plendil 5mg modified-release tablets (AstraZeneca UK Ltd)" | "Plendil 5mg modified-release tablets" | "Felodipine" | "5.000mg" | "Modified-release tablet" | "Oral" | "2060200" |
| "1093941000033116" | "562711000001104" | "Plendil 2.5mg modified-release tablets (AstraZeneca UK Ltd)" | "Plendil 2.5mg modified-release tablets" | "Felodipine" | "2.500mg" | "Modified-release tablet" | "Oral" | "2060200" |
| "2928841000033117" | "3800311000001106" | "Cabren 2.5mg modified-release tablets (Teva UK Ltd)" | "Cabren 2.5mg modified-release tablets" | "Felodipine" | "2.500mg" | "Modified-release tablet" | "Oral" | "" |
| "2928941000033113" | "3800511000001100" | "Cabren 5mg modified-release tablets (Teva UK Ltd)" | "Cabren 5mg modified-release tablets" | "Felodipine" | "5.000mg" | "Modified-release tablet" | "Oral" | "" |
| "2929041000033116" | "3800711000001105" | "Cabren 10mg modified-release tablets (Teva UK Ltd)" | "Cabren 10mg modified-release tablets" | "Felodipine" | "10.000mg" | "Modified-release tablet" | "Oral" | "" |
| "2979941000033119" | "4785111000001103" | "Felotens XL 5mg tablets (Thornton & Ross Ltd)" | "Felotens XL 5mg tablets" | "Felodipine" | "5.000mg" | "Modified-release tablet" | "Oral" | "2060200" |
| "2980041000033115" | "4785511000001107" | "Felotens XL 10mg tablets (Thornton & Ross Ltd)" | "Felotens XL 10mg tablets" | "Felodipine" | "10.000mg" | "Modified-release tablet" | "Oral" | "2060200" |
| "3177441000033110" | "4972811000001103" | "Felogen XL 5mg tablets (Mylan)" | "Felogen XL 5mg tablets" | "Felodipine" | "5.000mg" | "Modified-release tablet" | "Oral" | "2060200" |
| "3177541000033111" | "4973011000001100" | "Felogen XL 10mg tablets (Mylan)" | "Felogen XL 10mg tablets" | "Felodipine" | "10.000mg" | "Modified-release tablet" | "Oral" | "2060200" |
| "5576541000033113" | "5008511000001107" | "Folpik XL 5mg tablets (Teva UK Ltd)" | "Folpik XL 5mg tablets" | "Felodipine" | "5.000mg" | "Modified-release tablet" | "Oral" | "" |
| "5576341000033118" | "5008911000001100" | "Folpik XL 10mg tablets (Teva UK Ltd)" | "Folpik XL 10mg tablets" | "Felodipine" | "10.000mg" | "Modified-release tablet" | "Oral" | "" |
| "3034341000033117" | "5638311000001102" | "Vascalpha 5mg modified-release tablets (Accord Healthcare Ltd)" | "Vascalpha 5mg modified-release tablets" | "Felodipine" | "5.000mg" | "Modified-release tablet" | "Oral" | "2060200" |
| "3034441000033111" | "5638811000001106" | "Vascalpha 10mg modified-release tablets (Accord Healthcare Ltd)" | "Vascalpha 10mg modified-release tablets" | "Felodipine" | "10.000mg" | "Modified-release tablet" | "Oral" | "2060200" |
| "9121641000033116" | "7387911000001103" | "Parmid XL 5mg tablets (Sandoz Ltd)" | "Parmid XL 5mg tablets" | "Felodipine" | "5.000mg" | "Modified-release tablet" | "Oral" | "" |
| "9121741000033113" | "7388311000001103" | "Parmid XL 10mg tablets (Sandoz Ltd)" | "Parmid XL 10mg tablets" | "Felodipine" | "10.000mg" | "Modified-release tablet" | "Oral" | "" |
| "3154841000033119" | "7887011000001104" | "Cardioplen XL 5mg tablets (Chiesi Ltd)" | "Cardioplen XL 5mg tablets" | "Felodipine" | "5.000mg" | "Modified-release tablet" | "Oral" | "2060200" |
| "3154941000033110" | "7887511000001107" | "Cardioplen XL 10mg tablets (Chiesi Ltd)" | "Cardioplen XL 10mg tablets" | "Felodipine" | "10.000mg" | "Modified-release tablet" | "Oral" | "2060200" |
| "4521741000033117" | "8089811000001107" | "Neofel XL 10mg tablets (Kent Pharmaceuticals Ltd)" | "Neofel XL 10mg tablets" | "Felodipine" | "10.000mg" | "Modified-release tablet" | "Oral" | "2060200" |
| "4521641000033114" | "8090111000001106" | "Neofel XL 5mg tablets (Kent Pharmaceuticals Ltd)" | "Neofel XL 5mg tablets" | "Felodipine" | "5.000mg" | "Modified-release tablet" | "Oral" | "2060200" |
| "4152841000033112" | "11506711000001103" | "Cardioplen XL 2.5mg tablets (Chiesi Ltd)" | "Cardioplen XL 2.5mg tablets" | "Felodipine" | "2.500mg" | "Modified-release tablet" | "Oral" | "" |
| "4429241000033115" | "13127311000001107" | "Felotens XL 2.5mg tablets (Thornton & Ross Ltd)" | "Felotens XL 2.5mg tablets" | "Felodipine" | "2.500mg" | "Modified-release tablet" | "Oral" | "2060200" |
| "5576441000033112" | "13565311000001101" | "Folpik XL 2.5mg tablets (Teva UK Ltd)" | "Folpik XL 2.5mg tablets" | "Felodipine" | "2.500mg" | "Modified-release tablet" | "Oral" | "" |
| "4521541000033113" | "18167311000001103" | "Neofel XL 2.5mg tablets (Actavis UK Ltd)" | "Neofel XL 2.5mg tablets" | "Felodipine" | "2.500mg" | "Modified-release tablet" | "Oral" | "" |
| "9121541000033117" | "24221811000001105" | "Parmid XL 2.5mg tablets (Sandoz Ltd)" | "Parmid XL 2.5mg tablets" | "Felodipine" | "2.500mg" | "Modified-release tablet" | "Oral" | "" |
| "568041000033111" | "39020311000001104" | "Felodipine 2.5mg modified-release tablets" | "Felodipine 2.5mg modified-release tablets" | "Felodipine" | "2.500mg" | "Modified-release tablet" | "Oral" | "2060200" |
| "569041000033115" | "39020511000001105" | "Felodipine 10mg modified-release tablets" | "Felodipine 10mg modified-release tablets" | "Felodipine" | "10.000mg" | "Modified-release tablet" | "Oral" | "2060200" |
| "569141000033116" | "39020611000001109" | "Felodipine 5mg modified-release tablets" | "Felodipine 5mg modified-release tablets" | "Felodipine" | "5.000mg" | "Modified-release tablet" | "Oral" | "2060200" |
| "817541000033119" | "319300008" | "Lacidipine 2mg tablets" | "Lacidipine 2mg tablets" | "Lacidipine" | "2.000mg" | "Tablet" | "Oral" | "2060200" |
| "817641000033118" | "319301007" | "Lacidipine 4mg tablets" | "Lacidipine 4mg tablets" | "Lacidipine" | "4.000mg" | "Tablet" | "Oral" | "2060200" |
| "937141000033113" | "333011000001100" | "Motens 4mg tablets (GlaxoSmithKline UK Ltd)" | "Motens 4mg tablets" | "Lacidipine" | "4.000mg" | "Tablet" | "Oral" | "2060200" |
| "937041000033114" | "910911000001108" | "Motens 2mg tablets (GlaxoSmithKline UK Ltd)" | "Motens 2mg tablets" | "Lacidipine" | "2.000mg" | "Tablet" | "Oral" | "2060200" |
| "10217641000033116" | "28882211000001100" | "Molap 4mg tablets (Rivopharm (UK) Ltd)" | "Molap 4mg tablets" | "Lacidipine" | "4.000mg" | "Tablet" | "Oral" | "" |
| "13344441000033112" | "38019311000001102" | "Lacidipine 6mg tablets" | "Lacidipine 6mg tablets" | "Lacidipine" | "6.000mg" | "Tablet" | "Oral" | "" |
| "825941000033112" | "319316005" | "Lercanidipine 10mg tablets" | "Lercanidipine 10mg tablets" | "Lercanidipine hydrochloride" | "10.000mg" | "Tablet" | "Oral" | "2060200" |
| "1550841000033111" | "20011000001105" | "Zanidip 10mg tablets (Recordati Pharmaceuticals Ltd)" | "Zanidip 10mg tablets" | "Lercanidipine hydrochloride" | "10.000mg" | "Tablet" | "Oral" | "2060200" |
| "3908841000033116" | "10198711000001102" | "Zanidip 20mg tablets (Recordati Pharmaceuticals Ltd)" | "Zanidip 20mg tablets" | "Lercanidipine hydrochloride" | "20.000mg" | "Tablet" | "Oral" | "" |
| "3908741000033114" | "10225911000001102" | "Lercanidipine 20mg tablets" | "Lercanidipine 20mg tablets" | "Lercanidipine hydrochloride" | "20.000mg" | "Tablet" | "Oral" | "" |
| "967741000033115" | "319217004" | "Nicardipine 20mg capsules" | "Nicardipine 20mg capsules" | "Nicardipine hydrochloride" | "20.000mg" | "Capsule" | "Oral" | "2060200" |
| "967141000033119" | "319218009" | "Nicardipine 30mg capsules" | "Nicardipine 30mg capsules" | "Nicardipine hydrochloride" | "30.000mg" | "Capsule" | "Oral" | "2060200" |
| "199041000033110" | "118811000001102" | "Cardene SR 45mg capsules (Astellas Pharma Ltd)" | "Cardene SR 45mg capsules" | "Nicardipine hydrochloride" | "45.000mg" | "Modified-release capsule" | "Oral" | "2060200" |
| "176841000033116" | "291111000001102" | "Cardene 30mg capsules (Astellas Pharma Ltd)" | "Cardene 30mg capsules" | "Nicardipine hydrochloride" | "30.000mg" | "Capsule" | "Oral" | "2060200" |
| "176741000033114" | "344811000001108" | "Cardene 20mg capsules (Astellas Pharma Ltd)" | "Cardene 20mg capsules" | "Nicardipine hydrochloride" | "20.000mg" | "Capsule" | "Oral" | "2060200" |
| "198941000033118" | "540311000001105" | "Cardene SR 30mg capsules (Astellas Pharma Ltd)" | "Cardene SR 30mg capsules" | "Nicardipine hydrochloride" | "30.000mg" | "Modified-release capsule" | "Oral" | "2060200" |
| "9697741000033114" | "27126711000001103" | "Nicardipine 10mg/10ml solution for infusion ampoules" | "Nicardipine 10mg/10ml solution for infusion ampoules" | "Nicardipine hydrochloride" | "1.000mg/1.000ml" | "Solution for infusion" | "Intravenous" | "2060200" |
| "971441000033112" | "39021711000001108" | "Nicardipine 30mg modified-release capsules" | "Nicardipine 30mg modified-release capsules" | "Nicardipine hydrochloride" | "30.000mg" | "Modified-release capsule" | "Oral" | "2060200" |
| "971541000033113" | "39021811000001100" | "Nicardipine 45mg modified-release capsules" | "Nicardipine 45mg modified-release capsules" | "Nicardipine hydrochloride" | "45.000mg" | "Modified-release capsule" | "Oral" | "2060200" |
| "967341000033116" | "319222004" | "Nifedipine 5mg capsules" | "Nifedipine 5mg capsules" | "Nifedipine" | "5.000mg" | "Capsule" | "Oral" | "2060200" |
| "967241000033114" | "319223009" | "Nifedipine 10mg capsules" | "Nifedipine 10mg capsules" | "Nifedipine" | "10.000mg" | "Capsule" | "Oral" | "2060200" |
| "19341000033119" | "5011000001109" | "Adalat retard 20mg tablets (Bayer Plc)" | "Adalat retard 20mg tablets" | "Nifedipine" | "20.000mg" | "Modified-release tablet" | "Oral" | "2060200" |
| "2295741000033113" | "17011000001100" | "Calchan MR 20 tablets (Ranbaxy (UK) Ltd)" | "Calchan MR 20 tablets" | "Nifedipine" | "20.000mg" | "Modified-release tablet" | "Oral" | "" |
| "1580841000033116" | "25911000001100" | "Cardilate MR 10mg tablets (Teva UK Ltd)" | "Cardilate MR 10mg tablets" | "Nifedipine" | "10.000mg" | "Modified-release tablet" | "Oral" | "" |
| "14141000033116" | "27111000001107" | "Adalat 5mg capsules (Bayer Plc)" | "Adalat 5mg capsules" | "Nifedipine" | "5.000mg" | "Capsule" | "Oral" | "2060200" |
| "18241000033116" | "74111000001103" | "Adipine MR 20 tablets (Chiesi Ltd)" | "Adipine MR 20 tablets" | "Nifedipine" | "20.000mg" | "Modified-release tablet" | "Oral" | "2060200" |
| "336641000033114" | "126411000001108" | "Coracten SR 10mg capsules (UCB Pharma Ltd)" | "Coracten SR 10mg capsules" | "Nifedipine" | "10.000mg" | "Modified-release capsule" | "Oral" | "2060200" |
| "1724941000033113" | "162811000001100" | "Coracten XL 30mg capsules (UCB Pharma Ltd)" | "Coracten XL 30mg capsules" | "Nifedipine" | "30.000mg" | "Modified-release capsule" | "Oral" | "2060200" |
| "605941000033110" | "188711000001108" | "Fortipine LA 40 tablets (Advanz Pharma)" | "Fortipine LA 40 tablets" | "Nifedipine" | "40.000mg" | "Modified-release tablet" | "Oral" | "2060200" |
| "17241000033117" | "235511000001104" | "Adalat LA 60mg tablets (Bayer Plc)" | "Adalat LA 60mg tablets" | "Nifedipine" | "60.000mg" | "Modified-release tablet" | "Oral" | "2060200" |
| "2189541000033117" | "280811000001100" | "Nifopress Retard 20mg tablets (Advanz Pharma)" | "Nifopress Retard 20mg tablets" | "Nifedipine" | "20.000mg" | "Modified-release tablet" | "Oral" | "" |
| "2051241000033116" | "309611000001102" | "Coroday MR 20mg tablets (Mylan)" | "Coroday MR 20mg tablets" | "Nifedipine" | "20.000mg" | "Modified-release tablet" | "Oral" | "" |
| "1421341000033117" | "385611000001104" | "Tensipine MR 20 tablets (Genus Pharmaceuticals Ltd)" | "Tensipine MR 20 tablets" | "Nifedipine" | "20.000mg" | "Modified-release tablet" | "Oral" | "2060200" |
| "364241000033112" | "389611000001101" | "Coracten SR 20mg capsules (UCB Pharma Ltd)" | "Coracten SR 20mg capsules" | "Nifedipine" | "20.000mg" | "Modified-release capsule" | "Oral" | "2060200" |
| "1421241000033110" | "413111000001101" | "Tensipine MR 10 tablets (Genus Pharmaceuticals Ltd)" | "Tensipine MR 10 tablets" | "Nifedipine" | "10.000mg" | "Modified-release tablet" | "Oral" | "2060200" |
| "66141000033114" | "568911000001104" | "Angiopine MR 10mg tablets (Ashbourne Pharmaceuticals Ltd)" | "Angiopine MR 10mg tablets" | "Nifedipine" | "10.000mg" | "Modified-release tablet" | "Oral" | "" |
| "19441000033113" | "569011000001108" | "Adalat retard 10mg tablets (Bayer Plc)" | "Adalat retard 10mg tablets" | "Nifedipine" | "10.000mg" | "Modified-release tablet" | "Oral" | "2060200" |
| "1745141000033114" | "619111000001101" | "Nifedipress MR 20 tablets (Dexcel-Pharma Ltd)" | "Nifedipress MR 20 tablets" | "Nifedipine" | "20.000mg" | "Modified-release tablet" | "Oral" | "" |
| "2295641000033116" | "627111000001104" | "Calchan MR 10 tablets (Ranbaxy (UK) Ltd)" | "Calchan MR 10 tablets" | "Nifedipine" | "10.000mg" | "Modified-release tablet" | "Oral" | "" |
| "1753041000033113" | "630411000001107" | "Slofedipine XL 60 tablets (Zentiva)" | "Slofedipine XL 60 tablets" | "Nifedipine" | "60.000mg" | "Modified-release tablet" | "Oral" | "" |
| "738141000033119" | "677411000001108" | "Hypolar Retard 20 tablets (Sandoz Ltd)" | "Hypolar Retard 20 tablets" | "Nifedipine" | "20.000mg" | "Modified-release tablet" | "Oral" | "" |
| "2955041000033115" | "693311000001101" | "Valni 20 Retard tablets (Tillomed Laboratories Ltd)" | "Valni 20 Retard tablets" | "Nifedipine" | "20.000mg" | "Modified-release tablet" | "Oral" | "" |
| "14041000033115" | "782511000001108" | "Adalat 10mg capsules (Bayer Plc)" | "Adalat 10mg capsules" | "Nifedipine" | "10.000mg" | "Capsule" | "Oral" | "2060200" |
| "61841000033111" | "811311000001107" | "Angiopine 10 capsules (Ashbourne Pharmaceuticals Ltd)" | "Angiopine 10 capsules" | "Nifedipine" | "10.000mg" | "Capsule" | "Oral" | "" |
| "18341000033114" | "833611000001109" | "Adipine MR 10 tablets (Chiesi Ltd)" | "Adipine MR 10 tablets" | "Nifedipine" | "10.000mg" | "Modified-release tablet" | "Oral" | "2060200" |
| "1697641000033111" | "843411000001105" | "Slofedipine 20mg tablets (Sterwin Medicines)" | "Slofedipine 20mg tablets" | "Nifedipine" | "20.000mg" | "Modified-release tablet" | "Oral" | "" |
| "65241000033114" | "865011000001105" | "Angiopine MR 20mg tablets (Ashbourne Pharmaceuticals Ltd)" | "Angiopine MR 20mg tablets" | "Nifedipine" | "20.000mg" | "Modified-release tablet" | "Oral" | "" |
| "1766341000033113" | "881811000001100" | "Adalat LA 20mg tablets (Bayer Plc)" | "Adalat LA 20mg tablets" | "Nifedipine" | "20.000mg" | "Modified-release tablet" | "Oral" | "2060200" |
| "1745041000033110" | "904011000001104" | "Nifedipress MR 10 tablets (Dexcel-Pharma Ltd)" | "Nifedipress MR 10 tablets" | "Nifedipine" | "10.000mg" | "Modified-release tablet" | "Oral" | "2060200" |
| "199141000033114" | "905711000001103" | "Cardilate MR 20mg tablets (IVAX Pharmaceuticals UK Ltd)" | "Cardilate MR 20mg tablets" | "Nifedipine" | "20.000mg" | "Modified-release tablet" | "Oral" | "" |
| "17141000033112" | "2881311000001105" | "Adalat LA 30mg tablets (Bayer Plc)" | "Adalat LA 30mg tablets" | "Nifedipine" | "30.000mg" | "Modified-release tablet" | "Oral" | "2060200" |
| "2776541000033111" | "2881811000001101" | "Hypolar XL 30 tablets (Sandoz Ltd)" | "Hypolar XL 30 tablets" | "Nifedipine" | "30.000mg" | "Modified-release tablet" | "Oral" | "" |
| "1752941000033115" | "2882011000001104" | "Slofedipine XL 30mg tablets (Zentiva)" | "Slofedipine XL 30mg tablets" | "Nifedipine" | "30.000mg" | "Modified-release tablet" | "Oral" | "" |
| "1725041000033113" | "3381511000001105" | "Coracten XL 60mg capsules (UCB Pharma Ltd)" | "Coracten XL 60mg capsules" | "Nifedipine" | "60.000mg" | "Modified-release capsule" | "Oral" | "2060200" |
| "5973741000033117" | "8670111000001106" | "Nifedipine 10mg/5ml oral suspension" | "Nifedipine 10mg/5ml oral suspension" | "Nifedipine" | "2.000mg/1.000ml" | "Oral suspension" | "Oral" | "" |
| "5056241000033112" | "8670311000001108" | "Nifedipine 5mg/5ml oral suspension" | "Nifedipine 5mg/5ml oral suspension" | "Nifedipine" | "1.000mg/1.000ml" | "Oral suspension" | "Oral" | "" |
| "3225441000033117" | "9049711000001108" | "Adipine XL 30mg tablets (Chiesi Ltd)" | "Adipine XL 30mg tablets" | "Nifedipine" | "30.000mg" | "Modified-release tablet" | "Oral" | "2060200" |
| "3225541000033116" | "9049911000001105" | "Adipine XL 60mg tablets (Chiesi Ltd)" | "Adipine XL 60mg tablets" | "Nifedipine" | "60.000mg" | "Modified-release tablet" | "Oral" | "2060200" |
| "2639341000033117" | "9096811000001100" | "Nifedipine 20mg/ml oral drops" | "Nifedipine 20mg/ml oral drops" | "Nifedipine" | "20.000mg/1.000ml" | "Oral drops" | "Oral" | "" |
| "3952641000033110" | "10189111000001106" | "Nimodrel XL 30mg tablets (Zurich Pharmaceuticals)" | "Nimodrel XL 30mg tablets" | "Nifedipine" | "30.000mg" | "Modified-release tablet" | "Oral" | "" |
| "3952741000033118" | "10189311000001108" | "Nimodrel XL 60mg tablets (Zurich Pharmaceuticals)" | "Nimodrel XL 60mg tablets" | "Nifedipine" | "60.000mg" | "Modified-release tablet" | "Oral" | "" |
| "5817441000033119" | "10751211000001100" | "Neozipine XL 30mg tablets (Kent Pharmaceuticals Ltd)" | "Neozipine XL 30mg tablets" | "Nifedipine" | "30.000mg" | "Modified-release tablet" | "Oral" | "" |
| "5817541000033118" | "10751411000001101" | "Neozipine XL 60mg tablets (Kent Pharmaceuticals Ltd)" | "Neozipine XL 60mg tablets" | "Nifedipine" | "60.000mg" | "Modified-release tablet" | "Oral" | "" |
| "5402541000033112" | "12303311000001109" | "Nifedipine 2.5mg/5ml oral suspension" | "Nifedipine 2.5mg/5ml oral suspension" | "Nifedipine" | "500.000microgram/1.000ml" | "Oral suspension" | "Oral" | "" |
| "4451941000033110" | "13401911000001109" | "Valni XL 30mg tablets (Zentiva)" | "Valni XL 30mg tablets" | "Nifedipine" | "30.000mg" | "Modified-release tablet" | "Oral" | "2060200" |
| "4452041000033116" | "13402111000001101" | "Valni XL 60mg tablets (Zentiva)" | "Valni XL 60mg tablets" | "Nifedipine" | "60.000mg" | "Modified-release tablet" | "Oral" | "2060200" |
| "5891941000033115" | "17666011000001106" | "Adanif XL 30mg tablets (Advanz Pharma)" | "Adanif XL 30mg tablets" | "Nifedipine" | "30.000mg" | "Modified-release tablet" | "Oral" | "" |
| "5892041000033114" | "17666211000001101" | "Adanif XL 60mg tablets (Advanz Pharma)" | "Adanif XL 60mg tablets" | "Nifedipine" | "60.000mg" | "Modified-release tablet" | "Oral" | "" |
| "12372341000033117" | "34685211000001104" | "Nidef 30mg modified-release tablets (Morningside Healthcare Ltd)" | "Nidef 30mg modified-release tablets" | "Nifedipine" | "30.000mg" | "Modified-release tablet" | "Oral" | "" |
| "12372441000033111" | "34685811000001103" | "Nidef 60mg modified-release tablets (Morningside Healthcare Ltd)" | "Nidef 60mg modified-release tablets" | "Nifedipine" | "60.000mg" | "Modified-release tablet" | "Oral" | "" |
| "1778841000033111" | "38896511000001101" | "Nifedipine 60mg modified-release capsules" | "Nifedipine 60mg modified-release capsules" | "Nifedipine" | "60.000mg" | "Modified-release capsule" | "Oral" | "2060200" |
| "974141000033119" | "39022511000001106" | "Nifedipine 20mg modified-release capsules" | "Nifedipine 20mg modified-release capsules" | "Nifedipine" | "20.000mg" | "Modified-release capsule" | "Oral" | "2060200" |
| "971341000033118" | "39022611000001105" | "Nifedipine 60mg modified-release tablets" | "Nifedipine 60mg modified-release tablets" | "Nifedipine" | "60.000mg" | "Modified-release tablet" | "Oral" | "2060200" |
| "971841000033110" | "39022711000001101" | "Nifedipine 10mg modified-release tablets" | "Nifedipine 10mg modified-release tablets" | "Nifedipine" | "10.000mg" | "Modified-release tablet" | "Oral" | "2060200" |
| "970941000033113" | "39022811000001109" | "Nifedipine 20mg modified-release tablets" | "Nifedipine 20mg modified-release tablets" | "Nifedipine" | "20.000mg" | "Modified-release tablet" | "Oral" | "2060200" |
| "971141000033116" | "39022911000001104" | "Nifedipine 10mg modified-release capsules" | "Nifedipine 10mg modified-release capsules" | "Nifedipine" | "10.000mg" | "Modified-release capsule" | "Oral" | "2060200" |
| "1778741000033118" | "39107511000001109" | "Nifedipine 30mg modified-release capsules" | "Nifedipine 30mg modified-release capsules" | "Nifedipine" | "30.000mg" | "Modified-release capsule" | "Oral" | "2060200" |
| "972441000033116" | "39107611000001108" | "Nifedipine 40mg modified-release tablets" | "Nifedipine 40mg modified-release tablets" | "Nifedipine" | "40.000mg" | "Modified-release tablet" | "Oral" | "2060200" |
| "971241000033111" | "39111711000001105" | "Nifedipine 30mg modified-release tablets" | "Nifedipine 30mg modified-release tablets" | "Nifedipine" | "30.000mg" | "Modified-release tablet" | "Oral" | "2060200" |
| "977441000033119" | "323273000" | "Nimodipine 30mg tablets" | "Nimodipine 30mg tablets" | "Nimodipine" | "30.000mg" | "Tablet" | "Oral" | "2060200" |
| "977741000033114" | "3879211000001104" | "Nimotop 30mg tablets (Bayer Plc)" | "Nimotop 30mg tablets" | "Nimodipine" | "30.000mg" | "Tablet" | "Oral" | "2060200" |
| "970841000033117" | "4387211000001100" | "Nimotop 0.02% solution for infusion 50ml vials (Bayer Plc)" | "Nimotop 0.02% solution for infusion 50ml vials" | "Nimodipine" | "200.000microgram/1.000ml" | "Solution for infusion" | "Intravenous" | "2060200" |
| "970741000033110" | "36031711000001104" | "Nimodipine 10mg/50ml solution for infusion vials" | "Nimodipine 10mg/50ml solution for infusion vials" | "Nimodipine" | "200.000microgram/1.000ml" | "Solution for infusion" | "Intravenous" | "2060200" |
| "1513241000033114" | "318204006" | "Verapamil 40mg tablets" | "Verapamil 40mg tablets" | "Verapamil hydrochloride" | "40.000mg" | "Tablet" | "Oral" | "2060200" |
| "1513341000033116" | "318205007" | "Verapamil 80mg tablets" | "Verapamil 80mg tablets" | "Verapamil hydrochloride" | "80.000mg" | "Tablet" | "Oral" | "2060200" |
| "1512941000033111" | "318206008" | "Verapamil 120mg tablets" | "Verapamil 120mg tablets" | "Verapamil hydrochloride" | "120.000mg" | "Tablet" | "Oral" | "2060200" |
| "1513141000033119" | "318248001" | "Verapamil 160mg tablets" | "Verapamil 160mg tablets" | "Verapamil hydrochloride" | "160.000mg" | "Tablet" | "Oral" | "2060200" |
| "1489741000033113" | "65411000001102" | "Univer 240mg modified-release capsules (Teva UK Ltd)" | "Univer 240mg modified-release capsules" | "Verapamil hydrochloride" | "240.000mg" | "Modified-release capsule" | "Oral" | "2060200" |
| "1274741000033119" | "140411000001109" | "Securon 120mg tablets (Abbott Laboratories Ltd)" | "Securon 120mg tablets" | "Verapamil hydrochloride" | "120.000mg" | "Tablet" | "Oral" | "" |
| "1489541000033117" | "219711000001103" | "Univer 120mg modified-release capsules (Teva UK Ltd)" | "Univer 120mg modified-release capsules" | "Verapamil hydrochloride" | "120.000mg" | "Modified-release capsule" | "Oral" | "2060200" |
| "375141000033114" | "258911000001104" | "Cordilox 80mg tablets (IVAX Pharmaceuticals UK Ltd)" | "Cordilox 80mg tablets" | "Verapamil hydrochloride" | "80.000mg" | "Tablet" | "Oral" | "2060200" |
| "1489641000033116" | "391811000001102" | "Univer 180mg modified-release capsules (Teva UK Ltd)" | "Univer 180mg modified-release capsules" | "Verapamil hydrochloride" | "180.000mg" | "Modified-release capsule" | "Oral" | "2060200" |
| "1833541000033117" | "525311000001101" | "Verapress MR 240mg tablets (Dexcel-Pharma Ltd)" | "Verapress MR 240mg tablets" | "Verapamil hydrochloride" | "240.000mg" | "Modified-release tablet" | "Oral" | "2060200" |
| "1275141000033117" | "540111000001108" | "Securon SR 240mg tablets (Mylan)" | "Securon SR 240mg tablets" | "Verapamil hydrochloride" | "240.000mg" | "Modified-release tablet" | "Oral" | "2060200" |
| "375041000033110" | "557111000001106" | "Cordilox 40mg tablets (IVAX Pharmaceuticals UK Ltd)" | "Cordilox 40mg tablets" | "Verapamil hydrochloride" | "40.000mg" | "Tablet" | "Oral" | "2060200" |
| "1754841000033116" | "675611000001104" | "Vertab SR 240 tablets (Chiesi Ltd)" | "Vertab SR 240 tablets" | "Verapamil hydrochloride" | "240.000mg" | "Modified-release tablet" | "Oral" | "2060200" |
| "2129041000033118" | "740811000001102" | "Cordilox MR 240mg tablets (Teva UK Ltd)" | "Cordilox MR 240mg tablets" | "Verapamil hydrochloride" | "240.000mg" | "Modified-release tablet" | "Oral" | "2060200" |
| "2104741000033113" | "782111000001104" | "Ethimil MR 240mg tablets (Genus Pharmaceuticals Ltd)" | "Ethimil MR 240mg tablets" | "Verapamil hydrochloride" | "240.000mg" | "Modified-release tablet" | "Oral" | "" |
| "374941000033110" | "783511000001101" | "Cordilox 160mg tablets (IVAX Pharmaceuticals UK Ltd)" | "Cordilox 160mg tablets" | "Verapamil hydrochloride" | "160.000mg" | "Tablet" | "Oral" | "2060200" |
| "660641000033112" | "846611000001102" | "Half Securon SR 120mg tablets (Mylan)" | "Half Securon SR 120mg tablets" | "Verapamil hydrochloride" | "120.000mg" | "Modified-release tablet" | "Oral" | "2060200" |
| "374841000033119" | "867011000001103" | "Cordilox 120mg tablets (IVAX Pharmaceuticals UK Ltd)" | "Cordilox 120mg tablets" | "Verapamil hydrochloride" | "120.000mg" | "Tablet" | "Oral" | "" |
| "2845841000033119" | "3634111000001100" | "Ranvera MR 240mg tablets (Ranbaxy (UK) Ltd)" | "Ranvera MR 240mg tablets" | "Verapamil hydrochloride" | "240.000mg" | "Modified-release tablet" | "Oral" | "" |
| "2641041000033116" | "4129511000001107" | "Zolvera 40mg/5ml oral solution (Rosemont Pharmaceuticals Ltd)" | "Zolvera 40mg/5ml oral solution" | "Verapamil hydrochloride" | "8.000mg/1.000ml" | "Oral solution" | "Oral" | "2060200" |
| "1509841000033114" | "4139411000001106" | "Verapamil 40mg/5ml oral solution sugar free" | "Verapamil 40mg/5ml oral solution sugar free" | "Verapamil hydrochloride" | "8.000mg/1.000ml" | "Oral solution" | "Oral" | "2060200" |
| "1271741000033116" | "4399811000001108" | "Securon IV 5mg/2ml solution for injection ampoules (Mylan)" | "Securon IV 5mg/2ml solution for injection ampoules" | "Verapamil hydrochloride" | "2.500mg/1.000ml" | "Solution for injection" | "Intravenous" | "2060200" |
| "2890641000033116" | "7418511000001104" | "Vera-Til SR 120mg tablets (Tillomed Laboratories Ltd)" | "Vera-Til SR 120mg tablets" | "Verapamil hydrochloride" | "120.000mg" | "Modified-release tablet" | "Oral" | "" |
| "2890741000033113" | "7418711000001109" | "Vera-Til SR 240mg tablets (Tillomed Laboratories Ltd)" | "Vera-Til SR 240mg tablets" | "Verapamil hydrochloride" | "240.000mg" | "Modified-release tablet" | "Oral" | "" |
| "1509041000033119" | "35367911000001103" | "Verapamil 120mg modified-release tablets" | "Verapamil 120mg modified-release tablets" | "Verapamil hydrochloride" | "120.000mg" | "Modified-release tablet" | "Oral" | "2060200" |
| "1511241000033113" | "36149411000001103" | "Verapamil 180mg modified-release capsules" | "Verapamil 180mg modified-release capsules" | "Verapamil hydrochloride" | "180.000mg" | "Modified-release capsule" | "Oral" | "2060200" |
| "4260341000033110" | "36149611000001100" | "Verapamil 5mg/2ml solution for injection ampoules" | "Verapamil 5mg/2ml solution for injection ampoules" | "Verapamil hydrochloride" | "2.500mg/1.000ml" | "Solution for injection" | "Intravenous" | "2060200" |
| "1511141000033118" | "36565011000001105" | "Verapamil 120mg modified-release capsules" | "Verapamil 120mg modified-release capsules" | "Verapamil hydrochloride" | "120.000mg" | "Modified-release capsule" | "Oral" | "2060200" |
| "1509141000033115" | "38750211000001107" | "Verapamil 240mg modified-release tablets" | "Verapamil 240mg modified-release tablets" | "Verapamil hydrochloride" | "240.000mg" | "Modified-release tablet" | "Oral" | "2060200" |
| "1511341000033115" | "39021011000001106" | "Verapamil 240mg modified-release capsules" | "Verapamil 240mg modified-release capsules" | "Verapamil hydrochloride" | "240.000mg" | "Modified-release capsule" | "Oral" | "2060200" |
| "8.51841E+14" | "318955005" | "Losartan 25mg tablets" | "Losartan 25mg tablets" | "Losartan potassium" | "25.000mg" | "Tablet" | "Oral" | "2050502" |
| "8.51941E+14" | "318956006" | "Losartan 50mg tablets" | "Losartan 50mg tablets" | "Losartan potassium" | "50.000mg" | "Tablet" | "Oral" | "2050502" |
| "2.72014E+15" | "407784004" | "Losartan 100mg tablets" | "Losartan 100mg tablets" | "Losartan potassium" | "100.000mg" | "Tablet" | "Oral" | "2050502" |
| "3.70541E+14" | "5.3611E+13" | "Cozaar 50mg tablets (Organon Pharma (UK) Ltd)" | "Cozaar 50mg tablets" | "Losartan potassium" | "50.000mg" | "Tablet" | "Oral" | "2050502" |
| "2.72024E+15" | "2.45811E+14" | "Cozaar 100mg tablets (Organon Pharma (UK) Ltd)" | "Cozaar 100mg tablets" | "Losartan potassium" | "100.000mg" | "Tablet" | "Oral" | "2050502" |
| "3.70441E+14" | "2.66511E+14" | "Cozaar 25mg tablets (Organon Pharma (UK) Ltd)" | "Cozaar 25mg tablets" | "Losartan potassium" | "25.000mg" | "Tablet" | "Oral" | "2050502" |
| "5.97194E+15" | "1.41594E+16" | "Losartan 50mg/5ml oral solution" | "Losartan 50mg/5ml oral solution" | "Losartan potassium" | "10.000mg/1.000ml" | "Oral solution" | "Oral" | "" |
| "5.97204E+15" | "1.41595E+16" | "Losartan 50mg/5ml oral suspension" | "Losartan 50mg/5ml oral suspension" | "Losartan potassium" | "10.000mg/1.000ml" | "Oral suspension" | "Oral" | "" |
| "4.95764E+15" | "1.51389E+16" | "Cozaar 12.5mg tablets (Organon Pharma (UK) Ltd)" | "Cozaar 12.5mg tablets" | "Losartan potassium" | "12.500mg" | "Tablet" | "Oral" | "" |
| "4.95754E+15" | "1.51481E+16" | "Losartan 12.5mg tablets" | "Losartan 12.5mg tablets" | "Losartan potassium" | "12.500mg" | "Tablet" | "Oral" | "" |
| "5.97174E+15" | "1.54511E+16" | "Losartan 100mg/5ml oral solution" | "Losartan 100mg/5ml oral solution" | "Losartan potassium" | "20.000mg/1.000ml" | "Oral solution" | "Oral" | "" |
| "5.97184E+15" | "1.54512E+16" | "Losartan 100mg/5ml oral suspension" | "Losartan 100mg/5ml oral suspension" | "Losartan potassium" | "20.000mg/1.000ml" | "Oral suspension" | "Oral" | "" |
| "5.15004E+15" | "1.55068E+16" | "Cozaar 2.5mg/ml oral suspension (Organon Pharma (UK) Ltd)" | "Cozaar 2.5mg/ml oral suspension" | "Losartan potassium" | "2.500mg/1.000ml" | "Oral suspension" | "Oral" | "2050502" |
| "5.14994E+15" | "1.55074E+16" | "Losartan 2.5mg/ml oral suspension sugar free" | "Losartan 2.5mg/ml oral suspension sugar free" | "Losartan potassium" | "2.500mg/1.000ml" | "Oral suspension" | "Oral" | "2050502" |
| "4.02164E+15" | "1.11601E+16" | "Amlodipine 10mg / Valsartan 160mg tablets" | "Amlodipine 10mg / Valsartan 160mg tablets" | "Amlodipine besilate/ Valsartan" | "10.000mg + 160.000mg" | "Tablet" | "Oral" | "2060200" |
| "4.02154E+15" | "1.11602E+16" | "Amlodipine 5mg / Valsartan 160mg tablets" | "Amlodipine 5mg / Valsartan 160mg tablets" | "Amlodipine besilate/ Valsartan" | "5.000mg + 160.000mg" | "Tablet" | "Oral" | "2060200" |
| "4.02144E+15" | "1.11603E+16" | "Amlodipine 5mg / Valsartan 80mg tablets" | "Amlodipine 5mg / Valsartan 80mg tablets" | "Amlodipine besilate/ Valsartan" | "5.000mg + 80.000mg" | "Tablet" | "Oral" | "2060200" |
| "4.02194E+15" | "1.11607E+16" | "Exforge 10mg/160mg tablets (Novartis Pharmaceuticals UK Ltd)" | "Exforge 10mg/160mg tablets" | "Amlodipine besilate/ Valsartan" | "10.000mg + 160.000mg" | "Tablet" | "Oral" | "2060200" |
| "4.02184E+15" | "1.11615E+16" | "Exforge 5mg/160mg tablets (Novartis Pharmaceuticals UK Ltd)" | "Exforge 5mg/160mg tablets" | "Amlodipine besilate/ Valsartan" | "5.000mg + 160.000mg" | "Tablet" | "Oral" | "2060200" |
| "4.02174E+15" | "1.11618E+16" | "Exforge 5mg/80mg tablets (Novartis Pharmaceuticals UK Ltd)" | "Exforge 5mg/80mg tablets" | "Amlodipine besilate/ Valsartan" | "5.000mg + 80.000mg" | "Tablet" | "Oral" | "2060200" |
| "7.75494E+15" | "449109006" | "Azilsartan medoxomil 40mg tablets" | "Azilsartan medoxomil 40mg tablets" | "Azilsartan medoxomil" | "40.000mg" | "Tablet" | "Oral" | "2050502" |
| "7.75504E+15" | "449333009" | "Azilsartan medoxomil 80mg tablets" | "Azilsartan medoxomil 80mg tablets" | "Azilsartan medoxomil" | "80.000mg" | "Tablet" | "Oral" | "2050502" |
| "7.75514E+15" | "2.03509E+16" | "Edarbi 20mg tablets (Takeda UK Ltd)" | "Edarbi 20mg tablets" | "Azilsartan medoxomil" | "20.000mg" | "Tablet" | "Oral" | "2050502" |
| "7.75524E+15" | "2.03512E+16" | "Edarbi 40mg tablets (Takeda UK Ltd)" | "Edarbi 40mg tablets" | "Azilsartan medoxomil" | "40.000mg" | "Tablet" | "Oral" | "2050502" |
| "7.75534E+15" | "2.03518E+16" | "Edarbi 80mg tablets (Takeda UK Ltd)" | "Edarbi 80mg tablets" | "Azilsartan medoxomil" | "80.000mg" | "Tablet" | "Oral" | "2050502" |
| "7.75484E+15" | "2.04187E+16" | "Azilsartan medoxomil 20mg tablets" | "Azilsartan medoxomil 20mg tablets" | "Azilsartan medoxomil" | "20.000mg" | "Tablet" | "Oral" | "2050502" |
| "2.11641E+14" | "318977009" | "Candesartan 2mg tablets" | "Candesartan 2mg tablets" | "Candesartan cilexetil" | "2.000mg" | "Tablet" | "Oral" | "2050502" |
| "2.11741E+14" | "318978004" | "Candesartan 4mg tablets" | "Candesartan 4mg tablets" | "Candesartan cilexetil" | "4.000mg" | "Tablet" | "Oral" | "2050502" |
| "2.11841E+14" | "318979007" | "Candesartan 8mg tablets" | "Candesartan 8mg tablets" | "Candesartan cilexetil" | "8.000mg" | "Tablet" | "Oral" | "2050502" |
| "2.11541E+14" | "318980005" | "Candesartan 16mg tablets" | "Candesartan 16mg tablets" | "Candesartan cilexetil" | "16.000mg" | "Tablet" | "Oral" | "2050502" |
| "3.22724E+15" | "376998003" | "Candesartan 32mg tablets" | "Candesartan 32mg tablets" | "Candesartan cilexetil" | "32.000mg" | "Tablet" | "Oral" | "" |
| "5.9441E+13" | "3.6011E+13" | "Amias 8mg tablets (Takeda UK Ltd)" | "Amias 8mg tablets" | "Candesartan cilexetil" | "8.000mg" | "Tablet" | "Oral" | "2050502" |
| "5.9241E+13" | "9.7311E+13" | "Amias 2mg tablets (Takeda UK Ltd)" | "Amias 2mg tablets" | "Candesartan cilexetil" | "2.000mg" | "Tablet" | "Oral" | "2050502" |
| "5.9341E+13" | "8.57411E+14" | "Amias 4mg tablets (Takeda UK Ltd)" | "Amias 4mg tablets" | "Candesartan cilexetil" | "4.000mg" | "Tablet" | "Oral" | "2050502" |
| "5.9141E+13" | "9.08511E+14" | "Amias 16mg tablets (Takeda UK Ltd)" | "Amias 16mg tablets" | "Candesartan cilexetil" | "16.000mg" | "Tablet" | "Oral" | "2050502" |
| "3.22734E+15" | "8.98391E+15" | "Amias 32mg tablets (Takeda UK Ltd)" | "Amias 32mg tablets" | "Candesartan cilexetil" | "32.000mg" | "Tablet" | "Oral" | "" |
| "7.75641E+14" | "318968002" | "Irbesartan 75mg tablets" | "Irbesartan 75mg tablets" | "Irbesartan" | "75.000mg" | "Tablet" | "Oral" | "2050502" |
| "7.75441E+14" | "318969005" | "Irbesartan 150mg tablets" | "Irbesartan 150mg tablets" | "Irbesartan" | "150.000mg" | "Tablet" | "Oral" | "2050502" |
| "7.75541E+14" | "318970006" | "Irbesartan 300mg tablets" | "Irbesartan 300mg tablets" | "Irbesartan" | "300.000mg" | "Tablet" | "Oral" | "2050502" |
| "7.6041E+13" | "3.23211E+14" | "Aprovel 300mg tablets (Sanofi)" | "Aprovel 300mg tablets" | "Irbesartan" | "300.000mg" | "Tablet" | "Oral" | "2050502" |
| "7.6141E+13" | "4.34511E+14" | "Aprovel 75mg tablets (Sanofi)" | "Aprovel 75mg tablets" | "Irbesartan" | "75.000mg" | "Tablet" | "Oral" | "2050502" |
| "7.5941E+13" | "8.59711E+14" | "Aprovel 150mg tablets (Sanofi)" | "Aprovel 150mg tablets" | "Irbesartan" | "150.000mg" | "Tablet" | "Oral" | "2050502" |
| "5.97064E+15" | "8.58081E+15" | "Irbesartan 150mg/5ml oral suspension" | "Irbesartan 150mg/5ml oral suspension" | "Irbesartan" | "30.000mg/1.000ml" | "Oral suspension" | "Oral" | "" |
| "4.95434E+15" | "1.26395E+16" | "Irbesartan 300mg/5ml oral suspension" | "Irbesartan 300mg/5ml oral suspension" | "Irbesartan" | "60.000mg/1.000ml" | "Oral suspension" | "Oral" | "" |
| "6.16784E+15" | "1.88522E+16" | "Irbesartan 20mg oral powder sachets" | "Irbesartan 20mg oral powder sachets" | "Irbesartan" | "20.000mg" | "Powder" | "Oral" | "" |
| "6.42394E+15" | "1.92815E+16" | "Irbesartan 30mg oral powder sachets" | "Irbesartan 30mg oral powder sachets" | "Irbesartan" | "30.000mg" | "Powder" | "Oral" | "" |
| "6.43574E+15" | "1.94819E+16" | "Irbesartan 37.5mg oral powder sachets" | "Irbesartan 37.5mg oral powder sachets" | "Irbesartan" | "37.500mg" | "Powder" | "Oral" | "" |
| "8.19394E+15" | "2.15222E+16" | "Sabervel 75mg tablets (Aspire Pharma Ltd)" | "Sabervel 75mg tablets" | "Irbesartan" | "75.000mg" | "Tablet" | "Oral" | "" |
| "8.19404E+15" | "2.15224E+16" | "Sabervel 150mg tablets (Aspire Pharma Ltd)" | "Sabervel 150mg tablets" | "Irbesartan" | "150.000mg" | "Tablet" | "Oral" | "" |
| "8.19414E+15" | "2.15226E+16" | "Sabervel 300mg tablets (Aspire Pharma Ltd)" | "Sabervel 300mg tablets" | "Irbesartan" | "300.000mg" | "Tablet" | "Oral" | "" |
| "8.55414E+15" | "2.27203E+16" | "Ifirmasta 75mg tablets (Consilient Health Ltd)" | "Ifirmasta 75mg tablets" | "Irbesartan" | "75.000mg" | "Tablet" | "Oral" | "" |
| "8.55424E+15" | "2.27206E+16" | "Ifirmasta 150mg tablets (Consilient Health Ltd)" | "Ifirmasta 150mg tablets" | "Irbesartan" | "150.000mg" | "Tablet" | "Oral" | "" |
| "8.55434E+15" | "2.27208E+16" | "Ifirmasta 300mg tablets (Consilient Health Ltd)" | "Ifirmasta 300mg tablets" | "Irbesartan" | "300.000mg" | "Tablet" | "Oral" | "" |
| "2.94464E+15" | "385542009" | "Olmesartan medoxomil 20mg tablets" | "Olmesartan medoxomil 20mg tablets" | "Olmesartan medoxomil" | "20.000mg" | "Tablet" | "Oral" | "2050502" |
| "2.94474E+15" | "385543004" | "Olmesartan medoxomil 40mg tablets" | "Olmesartan medoxomil 40mg tablets" | "Olmesartan medoxomil" | "40.000mg" | "Tablet" | "Oral" | "2050502" |
| "2.94454E+15" | "408055003" | "Olmesartan medoxomil 10mg tablets" | "Olmesartan medoxomil 10mg tablets" | "Olmesartan medoxomil" | "10.000mg" | "Tablet" | "Oral" | "2050502" |
| "2.94484E+15" | "4.62401E+15" | "Olmetec 10mg tablets (Daiichi Sankyo UK Ltd)" | "Olmetec 10mg tablets" | "Olmesartan medoxomil" | "10.000mg" | "Tablet" | "Oral" | "2050502" |
| "2.94494E+15" | "4.62431E+15" | "Olmetec 20mg tablets (Daiichi Sankyo UK Ltd)" | "Olmetec 20mg tablets" | "Olmesartan medoxomil" | "20.000mg" | "Tablet" | "Oral" | "2050502" |
| "2.94504E+15" | "4.62461E+15" | "Olmetec 40mg tablets (Daiichi Sankyo UK Ltd)" | "Olmetec 40mg tablets" | "Olmesartan medoxomil" | "40.000mg" | "Tablet" | "Oral" | "2050502" |
| "4.27324E+15" | "1.46807E+16" | "Olmesartan medoxomil 10mg/5ml oral suspension" | "Olmesartan medoxomil 10mg/5ml oral suspension" | "Olmesartan medoxomil" | "2.000mg/1.000ml" | "Oral suspension" | "Oral" | "" |
| "2.27264E+15" | "134463001" | "Telmisartan 20mg tablets" | "Telmisartan 20mg tablets" | "Telmisartan" | "20.000mg" | "Tablet" | "Oral" | "2050502" |
| "1.84954E+15" | "318986004" | "Telmisartan 40mg tablets" | "Telmisartan 40mg tablets" | "Telmisartan" | "40.000mg" | "Tablet" | "Oral" | "2050502" |
| "1.84964E+15" | "318987008" | "Telmisartan 80mg tablets" | "Telmisartan 80mg tablets" | "Telmisartan" | "80.000mg" | "Tablet" | "Oral" | "2050502" |
| "1.84984E+15" | "5.27411E+14" | "Micardis 80mg tablets (Boehringer Ingelheim Ltd)" | "Micardis 80mg tablets" | "Telmisartan" | "80.000mg" | "Tablet" | "Oral" | "2050502" |
| "2.27274E+15" | "6.48711E+14" | "Micardis 20mg tablets (Boehringer Ingelheim Ltd)" | "Micardis 20mg tablets" | "Telmisartan" | "20.000mg" | "Tablet" | "Oral" | "2050502" |
| "1.84974E+15" | "9.24911E+14" | "Micardis 40mg tablets (Boehringer Ingelheim Ltd)" | "Micardis 40mg tablets" | "Telmisartan" | "40.000mg" | "Tablet" | "Oral" | "2050502" |
| "9.20934E+15" | "2.45552E+16" | "Tolura 20mg tablets (Consilient Health Ltd)" | "Tolura 20mg tablets" | "Telmisartan" | "20.000mg" | "Tablet" | "Oral" | "" |
| "9.20944E+15" | "2.45554E+16" | "Tolura 40mg tablets (Consilient Health Ltd)" | "Tolura 40mg tablets" | "Telmisartan" | "40.000mg" | "Tablet" | "Oral" | "" |
| "9.20954E+15" | "2.45556E+16" | "Tolura 80mg tablets (Consilient Health Ltd)" | "Tolura 80mg tablets" | "Telmisartan" | "80.000mg" | "Tablet" | "Oral" | "" |
| "1.49834E+15" | "318961008" | "Valsartan 40mg capsules" | "Valsartan 40mg capsules" | "Valsartan" | "40.000mg" | "Capsule" | "Oral" | "2050502" |
| "1.49844E+15" | "318962001" | "Valsartan 80mg capsules" | "Valsartan 80mg capsules" | "Valsartan" | "80.000mg" | "Capsule" | "Oral" | "2050502" |
| "1.49824E+15" | "318963006" | "Valsartan 160mg capsules" | "Valsartan 160mg capsules" | "Valsartan" | "160.000mg" | "Capsule" | "Oral" | "2050502" |
| "6.51544E+15" | "375034009" | "Valsartan 80mg tablets" | "Valsartan 80mg tablets" | "Valsartan" | "80.000mg" | "Tablet" | "Oral" | "" |
| "6.51554E+15" | "375035005" | "Valsartan 160mg tablets" | "Valsartan 160mg tablets" | "Valsartan" | "160.000mg" | "Tablet" | "Oral" | "" |
| "4.42494E+15" | "376487009" | "Valsartan 320mg tablets" | "Valsartan 320mg tablets" | "Valsartan" | "320.000mg" | "Tablet" | "Oral" | "" |
| "3.20134E+15" | "416515008" | "Valsartan 40mg tablets" | "Valsartan 40mg tablets" | "Valsartan" | "40.000mg" | "Tablet" | "Oral" | "2050502" |
| "4.37641E+14" | "1.17011E+14" | "Diovan 160mg capsules (Novartis Pharmaceuticals UK Ltd)" | "Diovan 160mg capsules" | "Valsartan" | "160.000mg" | "Capsule" | "Oral" | "2050502" |
| "4.37841E+14" | "5.54511E+14" | "Diovan 80mg capsules (Novartis Pharmaceuticals UK Ltd)" | "Diovan 80mg capsules" | "Valsartan" | "80.000mg" | "Capsule" | "Oral" | "2050502" |
| "4.37741E+14" | "7.77611E+14" | "Diovan 40mg capsules (Novartis Pharmaceuticals UK Ltd)" | "Diovan 40mg capsules" | "Valsartan" | "40.000mg" | "Capsule" | "Oral" | "2050502" |
| "3.20144E+15" | "8.26321E+15" | "Diovan 40mg tablets (Novartis Pharmaceuticals UK Ltd)" | "Diovan 40mg tablets" | "Valsartan" | "40.000mg" | "Tablet" | "Oral" | "2050502" |
| "4.42504E+15" | "1.31433E+16" | "Diovan 320mg tablets (Novartis Pharmaceuticals UK Ltd)" | "Diovan 320mg tablets" | "Valsartan" | "320.000mg" | "Tablet" | "Oral" | "" |
| "6.52854E+15" | "2.00017E+16" | "Diovan 3mg/1ml oral solution (Novartis Pharmaceuticals UK Ltd)" | "Diovan 3mg/1ml oral solution" | "Valsartan" | "3.000mg/1.000ml" | "Oral solution" | "Oral" | "" |
| "6.52844E+15" | "2.00074E+16" | "Valsartan 3mg/ml oral solution" | "Valsartan 3mg/ml oral solution" | "Valsartan" | "3.000mg/1.000ml" | "Oral solution" | "Oral" | "" |
| "837941000033119" | "318857003" | "Lisinopril 2.5mg tablets" | "Lisinopril 2.5mg tablets" | "Lisinopril" | "2.500mg" | "Tablet" | "Oral" | "2050501" |
| "838141000033117" | "318858008" | "Lisinopril 5mg tablets" | "Lisinopril 5mg tablets" | "Lisinopril" | "5.000mg" | "Tablet" | "Oral" | "2050501" |
| "837841000033110" | "318859000" | "Lisinopril 10mg tablets" | "Lisinopril 10mg tablets" | "Lisinopril" | "10.000mg" | "Tablet" | "Oral" | "2050501" |
| "838041000033116" | "318860005" | "Lisinopril 20mg tablets" | "Lisinopril 20mg tablets" | "Lisinopril" | "20.000mg" | "Tablet" | "Oral" | "2050501" |
| "216141000033119" | "56711000001109" | "Carace 20mg tablets (Bristol-Myers Squibb Pharmaceuticals Ltd)" | "Carace 20mg tablets" | "Lisinopril" | "20.000mg" | "Tablet" | "Oral" | "" |
| "215941000033111" | "315211000001104" | "Carace 10mg tablets (Bristol-Myers Squibb Pharmaceuticals Ltd)" | "Carace 10mg tablets" | "Lisinopril" | "10.000mg" | "Tablet" | "Oral" | "" |
| "216041000033118" | "321011000001103" | "Carace 2.5mg tablets (Bristol-Myers Squibb Pharmaceuticals Ltd)" | "Carace 2.5mg tablets" | "Lisinopril" | "2.500mg" | "Tablet" | "Oral" | "" |
| "1552041000033115" | "593111000001108" | "Zestril 2.5mg tablets (AstraZeneca UK Ltd)" | "Zestril 2.5mg tablets" | "Lisinopril" | "2.500mg" | "Tablet" | "Oral" | "2050501" |
| "216241000033114" | "778011000001109" | "Carace 5mg tablets (Bristol-Myers Squibb Pharmaceuticals Ltd)" | "Carace 5mg tablets" | "Lisinopril" | "5.000mg" | "Tablet" | "Oral" | "" |
| "1552241000033111" | "823211000001109" | "Zestril 5mg tablets (AstraZeneca UK Ltd)" | "Zestril 5mg tablets" | "Lisinopril" | "5.000mg" | "Tablet" | "Oral" | "2050501" |
| "1551941000033114" | "825311000001100" | "Zestril 10mg tablets (AstraZeneca UK Ltd)" | "Zestril 10mg tablets" | "Lisinopril" | "10.000mg" | "Tablet" | "Oral" | "2050501" |
| "1552141000033116" | "891711000001107" | "Zestril 20mg tablets (AstraZeneca UK Ltd)" | "Zestril 20mg tablets" | "Lisinopril" | "20.000mg" | "Tablet" | "Oral" | "2050501" |
| "5971341000033117" | "8622111000001109" | "Lisinopril 2.5mg/5ml oral solution" | "Lisinopril 2.5mg/5ml oral solution" | "Lisinopril" | "500.000microgram/1.000ml" | "Oral solution" | "Oral" | "" |
| "5971441000033111" | "8622211000001103" | "Lisinopril 2.5mg/5ml oral suspension" | "Lisinopril 2.5mg/5ml oral suspension" | "Lisinopril" | "500.000microgram/1.000ml" | "Oral suspension" | "Oral" | "" |
| "6044641000033119" | "8622311000001106" | "Lisinopril 20mg/5ml oral solution" | "Lisinopril 20mg/5ml oral solution" | "Lisinopril" | "4.000mg/1.000ml" | "Oral solution" | "Oral" | "" |
| "6044541000033115" | "8622411000001104" | "Lisinopril 20mg/5ml oral suspension" | "Lisinopril 20mg/5ml oral suspension" | "Lisinopril" | "4.000mg/1.000ml" | "Oral suspension" | "Oral" | "" |
| "5890241000033117" | "8622511000001100" | "Lisinopril 5mg/5ml oral solution" | "Lisinopril 5mg/5ml oral solution" | "Lisinopril" | "1.000mg/1.000ml" | "Oral solution" | "Oral" | "" |
| "3152141000033115" | "8622611000001101" | "Lisinopril 5mg/5ml oral suspension" | "Lisinopril 5mg/5ml oral suspension" | "Lisinopril" | "1.000mg/1.000ml" | "Oral suspension" | "Oral" | "" |
| "5971541000033112" | "8622811000001102" | "Lisinopril 7.5mg/5ml oral solution" | "Lisinopril 7.5mg/5ml oral solution" | "Lisinopril" | "1.500mg/1.000ml" | "Oral solution" | "Oral" | "" |
| "5971641000033113" | "8622911000001107" | "Lisinopril 7.5mg/5ml oral suspension" | "Lisinopril 7.5mg/5ml oral suspension" | "Lisinopril" | "1.500mg/1.000ml" | "Oral suspension" | "Oral" | "" |
| "5568741000033113" | "20556011000001103" | "Lisinopril 40mg/5ml oral suspension" | "Lisinopril 40mg/5ml oral suspension" | "Lisinopril" | "8.000mg/1.000ml" | "Oral suspension" | "Oral" | "" |
| "10645041000033114" | "30251811000001106" | "Lisinopril 5mg/5ml oral solution sugar free" | "Lisinopril 5mg/5ml oral solution sugar free" | "Lisinopril" | "1.000mg/1.000ml" | "Oral solution" | "Oral" | "" |
| "938141000033112" | "318934008" | "Moexipril 7.5mg tablets" | "Moexipril 7.5mg tablets" | "Moexipril hydrochloride" | "7.500mg" | "Tablet" | "Oral" | "2050501" |
| "938041000033113" | "318935009" | "Moexipril 15mg tablets" | "Moexipril 15mg tablets" | "Moexipril hydrochloride" | "15.000mg" | "Tablet" | "Oral" | "2050501" |
| "1065941000033115" | "4040311000001101" | "Perdix 15mg tablets (UCB Pharma Ltd)" | "Perdix 15mg tablets" | "Moexipril hydrochloride" | "15.000mg" | "Tablet" | "Oral" | "2050501" |
| "1066041000033113" | "4041111000001109" | "Perdix 7.5mg tablets (UCB Pharma Ltd)" | "Perdix 7.5mg tablets" | "Moexipril hydrochloride" | "7.500mg" | "Tablet" | "Oral" | "2050501" |
| "1067841000033115" | "318896009" | "Perindopril erbumine 2mg tablets" | "Perindopril erbumine 2mg tablets" | "Perindopril erbumine" | "2.000mg" | "Tablet" | "Oral" | "2050501" |
| "1067941000033111" | "318897000" | "Perindopril erbumine 4mg tablets" | "Perindopril erbumine 4mg tablets" | "Perindopril erbumine" | "4.000mg" | "Tablet" | "Oral" | "2050501" |
| "2846741000033119" | "374667004" | "Perindopril erbumine 8mg tablets" | "Perindopril erbumine 8mg tablets" | "Perindopril erbumine" | "8.000mg" | "Tablet" | "Oral" | "2050501" |
| "377541000033114" | "48211000001104" | "Coversyl 2mg tablets (Servier Laboratories Ltd)" | "Coversyl 2mg tablets" | "Perindopril erbumine" | "2.000mg" | "Tablet" | "Oral" | "2050501" |
| "377641000033110" | "902211000001100" | "Coversyl 4mg tablets (Servier Laboratories Ltd)" | "Coversyl 4mg tablets" | "Perindopril erbumine" | "4.000mg" | "Tablet" | "Oral" | "2050501" |
| "2846641000033111" | "3803711000001101" | "Coversyl 8mg tablets (Servier Laboratories Ltd)" | "Coversyl 8mg tablets" | "Perindopril erbumine" | "8.000mg" | "Tablet" | "Oral" | "2050501" |
| "8276041000033117" | "8671311000001103" | "Perindopril erbumine 4mg/5ml oral suspension" | "Perindopril erbumine 4mg/5ml oral suspension" | "Perindopril erbumine" | "800.000microgram/1.000ml" | "Oral suspension" | "Oral" | "" |
| "8275941000033110" | "14057311000001102" | "Perindopril erbumine 8mg/5ml oral solution" | "Perindopril erbumine 8mg/5ml oral solution" | "Perindopril erbumine" | "1.600mg/1.000ml" | "Oral solution" | "Oral" | "" |
| "8276141000033118" | "14057411000001109" | "Perindopril erbumine 8mg/5ml oral suspension" | "Perindopril erbumine 8mg/5ml oral suspension" | "Perindopril erbumine" | "1.600mg/1.000ml" | "Oral suspension" | "Oral" | "" |
| "8869441000033119" | "23471511000001106" | "Perindopril erbumine 4mg/5ml oral solution" | "Perindopril erbumine 4mg/5ml oral solution" | "Perindopril erbumine" | "800.000microgram/1.000ml" | "Oral solution" | "Oral" | "" |
| "213441000033119" | "318820009" | "Captopril 12.5mg tablets" | "Captopril 12.5mg tablets" | "Captopril" | "12.500mg" | "Tablet" | "Oral" | "2050501" |
| "214441000033117" | "318821008" | "Captopril 25mg tablets" | "Captopril 25mg tablets" | "Captopril" | "25.000mg" | "Tablet" | "Oral" | "2050501" |
| "214541000033116" | "318824000" | "Captopril 50mg tablets" | "Captopril 50mg tablets" | "Captopril" | "50.000mg" | "Tablet" | "Oral" | "2050501" |
| "214141000033113" | "134511000001103" | "Capoten 12.5mg tablets (Bristol-Myers Squibb Pharmaceuticals Ltd)" | "Capoten 12.5mg tablets" | "Captopril" | "12.500mg" | "Tablet" | "Oral" | "2050501" |
| "2945841000033112" | "221411000001104" | "Ecopace 12.5mg tablets (Advanz Pharma)" | "Ecopace 12.5mg tablets" | "Captopril" | "12.500mg" | "Tablet" | "Oral" | "2050501" |
| "214341000033111" | "386611000001109" | "Capoten 50mg tablets (Bristol-Myers Squibb Pharmaceuticals Ltd)" | "Capoten 50mg tablets" | "Captopril" | "50.000mg" | "Tablet" | "Oral" | "2050501" |
| "214241000033118" | "455611000001103" | "Capoten 25mg tablets (Bristol-Myers Squibb Pharmaceuticals Ltd)" | "Capoten 25mg tablets" | "Captopril" | "25.000mg" | "Tablet" | "Oral" | "2050501" |
| "1622141000033114" | "477111000001100" | "Kaplon 25mg tablets (Teva UK Ltd)" | "Kaplon 25mg tablets" | "Captopril" | "25.000mg" | "Tablet" | "Oral" | "2050501" |
| "10941000033110" | "517711000001103" | "Acepril 25mg tablets (Bristol-Myers Squibb Pharmaceuticals Ltd)" | "Acepril 25mg tablets" | "Captopril" | "25.000mg" | "Tablet" | "Oral" | "2050501" |
| "1622241000033119" | "572511000001102" | "Kaplon 50mg tablets (Teva UK Ltd)" | "Kaplon 50mg tablets" | "Captopril" | "50.000mg" | "Tablet" | "Oral" | "2050501" |
| "2928441000033115" | "597511000001109" | "Tensopril 25mg tablets (Teva UK Ltd)" | "Tensopril 25mg tablets" | "Captopril" | "25.000mg" | "Tablet" | "Oral" | "2050501" |
| "2928541000033119" | "653111000001108" | "Tensopril 50mg tablets (Teva UK Ltd)" | "Tensopril 50mg tablets" | "Captopril" | "50.000mg" | "Tablet" | "Oral" | "2050501" |
| "10841000033119" | "660111000001107" | "Acepril 12.5mg tablets (Bristol-Myers Squibb Pharmaceuticals Ltd)" | "Acepril 12.5mg tablets" | "Captopril" | "12.500mg" | "Tablet" | "Oral" | "2050501" |
| "2928341000033114" | "673711000001107" | "Tensopril 12.5mg tablets (Teva UK Ltd)" | "Tensopril 12.5mg tablets" | "Captopril" | "12.500mg" | "Tablet" | "Oral" | "2050501" |
| "2946041000033114" | "767911000001101" | "Ecopace 50mg tablets (Advanz Pharma)" | "Ecopace 50mg tablets" | "Captopril" | "50.000mg" | "Tablet" | "Oral" | "2050501" |
| "1622041000033110" | "777711000001105" | "Kaplon 12.5mg tablets (Teva UK Ltd)" | "Kaplon 12.5mg tablets" | "Captopril" | "12.500mg" | "Tablet" | "Oral" | "2050501" |
| "11041000033117" | "814111000001108" | "Acepril 50mg tablets (Bristol-Myers Squibb Pharmaceuticals Ltd)" | "Acepril 50mg tablets" | "Captopril" | "50.000mg" | "Tablet" | "Oral" | "2050501" |
| "2945941000033116" | "817411000001108" | "Ecopace 25mg tablets (Advanz Pharma)" | "Ecopace 25mg tablets" | "Captopril" | "25.000mg" | "Tablet" | "Oral" | "2050501" |
| "5008141000033111" | "8346811000001109" | "Captopril 10mg/5ml oral suspension" | "Captopril 10mg/5ml oral suspension" | "Captopril" | "2.000mg/1.000ml" | "Oral suspension" | "Oral" | "" |
| "5898741000033114" | "8347111000001104" | "Captopril 12.5mg/5ml oral solution" | "Captopril 12.5mg/5ml oral solution" | "Captopril" | "2.500mg/1.000ml" | "Oral solution" | "Oral" | "" |
| "5898841000033116" | "8347211000001105" | "Captopril 12.5mg/5ml oral suspension" | "Captopril 12.5mg/5ml oral suspension" | "Captopril" | "2.500mg/1.000ml" | "Oral suspension" | "Oral" | "" |
| "5401641000033118" | "8347411000001109" | "Captopril 15mg/5ml oral suspension" | "Captopril 15mg/5ml oral suspension" | "Captopril" | "3.000mg/1.000ml" | "Oral suspension" | "Oral" | "" |
| "2719841000033113" | "8348311000001101" | "Captopril 20mg/5ml oral suspension" | "Captopril 20mg/5ml oral suspension" | "Captopril" | "4.000mg/1.000ml" | "Oral suspension" | "Oral" | "" |
| "3012741000033115" | "8348511000001107" | "Captopril 25mg/5ml oral solution" | "Captopril 25mg/5ml oral solution" | "Captopril" | "5.000mg/1.000ml" | "Oral solution" | "Oral" | "" |
| "5132041000033112" | "8348611000001106" | "Captopril 25mg/5ml oral suspension" | "Captopril 25mg/5ml oral suspension" | "Captopril" | "5.000mg/1.000ml" | "Oral suspension" | "Oral" | "" |
| "3851341000033117" | "8350511000001107" | "Captopril 3mg/5ml oral solution" | "Captopril 3mg/5ml oral solution" | "Captopril" | "600.000microgram/1.000ml" | "Oral solution" | "Oral" | "" |
| "6001041000033115" | "8351611000001101" | "Captopril 5mg/5ml oral solution" | "Captopril 5mg/5ml oral solution" | "Captopril" | "1.000mg/1.000ml" | "Oral solution" | "Oral" | "" |
| "2656341000033110" | "8351811000001102" | "Captopril 5mg/5ml oral suspension" | "Captopril 5mg/5ml oral suspension" | "Captopril" | "1.000mg/1.000ml" | "Oral suspension" | "Oral" | "" |
| "5898941000033112" | "8351911000001107" | "Captopril 6.25mg/5ml oral solution" | "Captopril 6.25mg/5ml oral solution" | "Captopril" | "1.250mg/1.000ml" | "Oral solution" | "Oral" | "" |
| "5899041000033115" | "8352011000001100" | "Captopril 6.25mg/5ml oral suspension" | "Captopril 6.25mg/5ml oral suspension" | "Captopril" | "1.250mg/1.000ml" | "Oral suspension" | "Oral" | "" |
| "3940841000033110" | "8791811000001109" | "Captopril 2mg capsules" | "Captopril 2mg capsules" | "Captopril" | "2.000mg" | "Capsule" | "Oral" | "" |
| "5454041000033117" | "8791911000001104" | "Captopril 4mg capsules" | "Captopril 4mg capsules" | "Captopril" | "4.000mg" | "Capsule" | "Oral" | "" |
| "3333941000033117" | "19820711000001101" | "Captopril 8mg/5ml oral suspension" | "Captopril 8mg/5ml oral suspension" | "Captopril" | "1.600mg/1.000ml" | "Oral suspension" | "Oral" | "" |
| "8961341000033119" | "23681711000001107" | "Noyada 25mg/5ml oral solution (Martindale Pharmaceuticals Ltd)" | "Noyada 25mg/5ml oral solution" | "Captopril" | "5.000mg/1.000ml" | "Oral solution" | "Oral" | "2050501" |
| "8961241000033112" | "23682011000001102" | "Noyada 5mg/5ml oral solution (Martindale Pharmaceuticals Ltd)" | "Noyada 5mg/5ml oral solution" | "Captopril" | "1.000mg/1.000ml" | "Oral solution" | "Oral" | "2050501" |
| "9108041000033111" | "23707311000001108" | "Captopril 25mg/5ml oral solution sugar free" | "Captopril 25mg/5ml oral solution sugar free" | "Captopril" | "5.000mg/1.000ml" | "Oral solution" | "Oral" | "2050501" |
| "9108141000033110" | "23707511000001102" | "Captopril 5mg/5ml oral solution sugar free" | "Captopril 5mg/5ml oral solution sugar free" | "Captopril" | "1.000mg/1.000ml" | "Oral solution" | "Oral" | "2050501" |
| "3160641000033118" | "318892006" | "Quinapril 10mg / Hydrochlorothiazide 12.5mg tablets" | "Quinapril 10mg / Hydrochlorothiazide 12.5mg tablets" | "Hydrochlorothiazide/ Quinapril hydrochloride" | "12.500mg + 10.000mg" | "Tablet" | "Oral" | "2050501" |
| "11241000033113" | "260211000001104" | "Accuretic 10mg/12.5mg tablets (Pfizer Ltd)" | "Accuretic 10mg/12.5mg tablets" | "Hydrochlorothiazide/ Quinapril hydrochloride" | "12.500mg + 10.000mg" | "Tablet" | "Oral" | "2050501" |
| "1149241000033111" | "318885001" | "Quinapril 5mg tablets" | "Quinapril 5mg tablets" | "Quinapril hydrochloride" | "5.000mg" | "Tablet" | "Oral" | "2050501" |
| "1149341000033118" | "318886000" | "Quinapril 10mg tablets" | "Quinapril 10mg tablets" | "Quinapril hydrochloride" | "10.000mg" | "Tablet" | "Oral" | "2050501" |
| "1149441000033112" | "318887009" | "Quinapril 20mg tablets" | "Quinapril 20mg tablets" | "Quinapril hydrochloride" | "20.000mg" | "Tablet" | "Oral" | "2050501" |
| "1149541000033113" | "318894007" | "Quinapril 40mg tablets" | "Quinapril 40mg tablets" | "Quinapril hydrochloride" | "40.000mg" | "Tablet" | "Oral" | "2050501" |
| "11641000033111" | "86411000001109" | "Accupro 40mg tablets (Pfizer Ltd)" | "Accupro 40mg tablets" | "Quinapril hydrochloride" | "40.000mg" | "Tablet" | "Oral" | "2050501" |
| "13241000033114" | "231111000001106" | "Accupro 10mg tablets (Pfizer Ltd)" | "Accupro 10mg tablets" | "Quinapril hydrochloride" | "10.000mg" | "Tablet" | "Oral" | "2050501" |
| "13441000033110" | "582611000001106" | "Accupro 5mg tablets (Pfizer Ltd)" | "Accupro 5mg tablets" | "Quinapril hydrochloride" | "5.000mg" | "Tablet" | "Oral" | "2050501" |
| "13341000033116" | "829111000001100" | "Accupro 20mg tablets (Pfizer Ltd)" | "Accupro 20mg tablets" | "Quinapril hydrochloride" | "20.000mg" | "Tablet" | "Oral" | "2050501" |
| "3283141000033118" | "9207711000001100" | "Quinil 5mg tablets (Tillomed Laboratories Ltd)" | "Quinil 5mg tablets" | "Quinapril hydrochloride" | "5.000mg" | "Tablet" | "Oral" | "" |
| "3283241000033113" | "9208111000001100" | "Quinil 10mg tablets (Tillomed Laboratories Ltd)" | "Quinil 10mg tablets" | "Quinapril hydrochloride" | "10.000mg" | "Tablet" | "Oral" | "" |
| "3283341000033115" | "9208411000001105" | "Quinil 20mg tablets (Tillomed Laboratories Ltd)" | "Quinil 20mg tablets" | "Quinapril hydrochloride" | "20.000mg" | "Tablet" | "Oral" | "" |
| "3283441000033114" | "9208711000001104" | "Quinil 40mg tablets (Tillomed Laboratories Ltd)" | "Quinil 40mg tablets" | "Quinapril hydrochloride" | "40.000mg" | "Tablet" | "Oral" | "" |
| "1151341000033117" | "318900007" | "Ramipril 1.25mg capsules" | "Ramipril 1.25mg capsules" | "Ramipril" | "1.250mg" | "Capsule" | "Oral" | "2050501" |
| "1151441000033111" | "318901006" | "Ramipril 2.5mg capsules" | "Ramipril 2.5mg capsules" | "Ramipril" | "2.500mg" | "Capsule" | "Oral" | "2050501" |
| "1151541000033112" | "318902004" | "Ramipril 5mg capsules" | "Ramipril 5mg capsules" | "Ramipril" | "5.000mg" | "Capsule" | "Oral" | "2050501" |
| "1769841000033111" | "318906001" | "Ramipril 10mg capsules" | "Ramipril 10mg capsules" | "Ramipril" | "10.000mg" | "Capsule" | "Oral" | "2050501" |
| "2989541000033115" | "408040007" | "Ramipril 1.25mg tablets" | "Ramipril 1.25mg tablets" | "Ramipril" | "1.250mg" | "Tablet" | "Oral" | "2050501" |
| "2989641000033119" | "408050008" | "Ramipril 2.5mg tablets" | "Ramipril 2.5mg tablets" | "Ramipril" | "2.500mg" | "Tablet" | "Oral" | "2050501" |
| "2989741000033111" | "408051007" | "Ramipril 5mg tablets" | "Ramipril 5mg tablets" | "Ramipril" | "5.000mg" | "Tablet" | "Oral" | "2050501" |
| "2989841000033118" | "408052000" | "Ramipril 10mg tablets" | "Ramipril 10mg tablets" | "Ramipril" | "10.000mg" | "Tablet" | "Oral" | "2050501" |
| "1769941000033115" | "43711000001100" | "Tritace 10mg capsules (Sanofi)" | "Tritace 10mg capsules" | "Ramipril" | "10.000mg" | "Capsule" | "Oral" | "2050501" |
| "1455541000033110" | "111611000001109" | "Tritace 1.25mg capsules (Aventis Pharma)" | "Tritace 1.25mg capsules" | "Ramipril" | "1.250mg" | "Capsule" | "Oral" | "2050501" |
| "1455741000033119" | "802311000001101" | "Tritace 5mg capsules (Sanofi)" | "Tritace 5mg capsules" | "Ramipril" | "5.000mg" | "Capsule" | "Oral" | "2050501" |
| "1455641000033111" | "835411000001105" | "Tritace 2.5mg capsules (Sanofi)" | "Tritace 2.5mg capsules" | "Ramipril" | "2.500mg" | "Capsule" | "Oral" | "2050501" |
| "2989941000033114" | "5010511000001106" | "Tritace 1.25mg tablets (Sanofi)" | "Tritace 1.25mg tablets" | "Ramipril" | "1.250mg" | "Tablet" | "Oral" | "2050501" |
| "2990041000033116" | "5010811000001109" | "Tritace 2.5mg tablets (Sanofi)" | "Tritace 2.5mg tablets" | "Ramipril" | "2.500mg" | "Tablet" | "Oral" | "2050501" |
| "2990141000033117" | "5011111000001108" | "Tritace 5mg tablets (Sanofi)" | "Tritace 5mg tablets" | "Ramipril" | "5.000mg" | "Tablet" | "Oral" | "2050501" |
| "2990241000033112" | "5011411000001103" | "Tritace 10mg tablets (Sanofi)" | "Tritace 10mg tablets" | "Ramipril" | "10.000mg" | "Tablet" | "Oral" | "2050501" |
| "3159741000033111" | "7948711000001102" | "Lopace 2.5mg capsules (Discovery Pharmaceuticals)" | "Lopace 2.5mg capsules" | "Ramipril" | "2.500mg" | "Capsule" | "Oral" | "2050501" |
| "3159841000033118" | "7948911000001100" | "Lopace 5mg capsules (Discovery Pharmaceuticals)" | "Lopace 5mg capsules" | "Ramipril" | "5.000mg" | "Capsule" | "Oral" | "2050501" |
| "3159941000033114" | "7949111000001105" | "Lopace 10mg capsules (Discovery Pharmaceuticals)" | "Lopace 10mg capsules" | "Ramipril" | "10.000mg" | "Capsule" | "Oral" | "2050501" |
| "5998241000033112" | "8720211000001109" | "Ramipril 1.25mg/5ml oral solution" | "Ramipril 1.25mg/5ml oral solution" | "Ramipril" | "250.000microgram/1.000ml" | "Oral solution" | "Oral" | "" |
| "4152241000033113" | "8720311000001101" | "Ramipril 1.25mg/5ml oral suspension" | "Ramipril 1.25mg/5ml oral suspension" | "Ramipril" | "250.000microgram/1.000ml" | "Oral suspension" | "Oral" | "" |
| "5887841000033118" | "8720411000001108" | "Ramipril 10mg/5ml oral solution" | "Ramipril 10mg/5ml oral solution" | "Ramipril" | "2.000mg/1.000ml" | "Oral solution" | "Oral" | "" |
| "5887941000033114" | "8720511000001107" | "Ramipril 10mg/5ml oral suspension" | "Ramipril 10mg/5ml oral suspension" | "Ramipril" | "2.000mg/1.000ml" | "Oral suspension" | "Oral" | "" |
| "5890541000033115" | "8720611000001106" | "Ramipril 2.5mg/5ml oral solution" | "Ramipril 2.5mg/5ml oral solution" | "Ramipril" | "500.000microgram/1.000ml" | "Oral solution" | "Oral" | "" |
| "4805941000033114" | "8720711000001102" | "Ramipril 2.5mg/5ml oral suspension" | "Ramipril 2.5mg/5ml oral suspension" | "Ramipril" | "500.000microgram/1.000ml" | "Oral suspension" | "Oral" | "" |
| "5890341000033110" | "8720811000001105" | "Ramipril 5mg/5ml oral solution" | "Ramipril 5mg/5ml oral solution" | "Ramipril" | "1.000mg/1.000ml" | "Oral solution" | "Oral" | "" |
| "2883341000033118" | "8720911000001100" | "Ramipril 5mg/5ml oral suspension" | "Ramipril 5mg/5ml oral suspension" | "Ramipril" | "1.000mg/1.000ml" | "Oral suspension" | "Oral" | "" |
| "6517041000033110" | "19877111000001100" | "Ramipril 2.5mg/5ml oral solution sugar free" | "Ramipril 2.5mg/5ml oral solution sugar free" | "Ramipril" | "500.000microgram/1.000ml" | "Oral solution" | "Oral" | "" |
| "1893141000033119" | "318806002" | "Co-zidocapt 12.5mg/25mg tablets" | "Co-zidocapt 12.5mg/25mg tablets" | "Captopril/ Hydrochlorothiazide" | "25.000mg + 12.500mg" | "Tablet" | "Oral" | "2050501" |
| "1893241000033114" | "318807006" | "Co-zidocapt 25mg/50mg tablets" | "Co-zidocapt 25mg/50mg tablets" | "Captopril/ Hydrochlorothiazide" | "50.000mg + 25.000mg" | "Tablet" | "Oral" | "2050501" |
| "216441000033110" | "17311000001102" | "Capozide 25mg/50mg tablets (Bristol-Myers Squibb Pharmaceuticals Ltd)" | "Capozide 25mg/50mg tablets" | "Captopril/ Hydrochlorothiazide" | "50.000mg + 25.000mg" | "Tablet" | "Oral" | "2050501" |
| "13141000033119" | "263311000001101" | "Acezide 25mg/50mg tablets (Bristol-Myers Squibb Pharmaceuticals Ltd)" | "Acezide 25mg/50mg tablets" | "Captopril/ Hydrochlorothiazide" | "50.000mg + 25.000mg" | "Tablet" | "Oral" | "2050501" |
| "209241000033119" | "546711000001107" | "Capozide LS 12.5mg/25mg tablets (Bristol-Myers Squibb Pharmaceuticals Ltd)" | "Capozide LS 12.5mg/25mg tablets" | "Captopril/ Hydrochlorothiazide" | "25.000mg + 12.500mg" | "Tablet" | "Oral" | "" |
| "609141000033111" | "318909008" | "Fosinopril 10mg tablets" | "Fosinopril 10mg tablets" | "Fosinopril sodium" | "10.000mg" | "Tablet" | "Oral" | "2050501" |
| "609241000033116" | "318910003" | "Fosinopril 20mg tablets" | "Fosinopril 20mg tablets" | "Fosinopril sodium" | "20.000mg" | "Tablet" | "Oral" | "2050501" |
| "1387441000033115" | "348111000001107" | "Staril 20mg tablets (Bristol-Myers Squibb Pharmaceuticals Ltd)" | "Staril 20mg tablets" | "Fosinopril sodium" | "20.000mg" | "Tablet" | "Oral" | "" |
| "1387341000033114" | "462511000001104" | "Staril 10mg tablets (Bristol-Myers Squibb Pharmaceuticals Ltd)" | "Staril 10mg tablets" | "Fosinopril sodium" | "10.000mg" | "Tablet" | "Oral" | "" |
| "1406341000033117" | "3691211000001101" | "Tarka modified-release capsules (Abbott Laboratories Ltd)" | "Tarka modified-release capsules" | "Trandolapril/ Verapamil hydrochloride" | "2.000mg + 180.000mg" | "Modified-release capsule" | "Oral" | "" |
| "3163041000033116" | "36149211000001102" | "Verapamil 180mg modified-release / Trandolapril 2mg capsules" | "Verapamil 180mg modified-release / Trandolapril 2mg capsules" | "Trandolapril/ Verapamil hydrochloride" | "2.000mg + 180.000mg" | "Modified-release capsule" | "Oral" | "" |
| "1454141000033119" | "318924004" | "Trandolapril 500microgram capsules" | "Trandolapril 500microgram capsules" | "Trandolapril" | "500.000microgram" | "Capsule" | "Oral" | "2050501" |
| "1453941000033115" | "318925003" | "Trandolapril 1mg capsules" | "Trandolapril 1mg capsules" | "Trandolapril" | "1.000mg" | "Capsule" | "Oral" | "2050501" |
| "1454041000033118" | "318926002" | "Trandolapril 2mg capsules" | "Trandolapril 2mg capsules" | "Trandolapril" | "2.000mg" | "Capsule" | "Oral" | "2050501" |
| "3014741000033111" | "410958005" | "Trandolapril 4mg capsules" | "Trandolapril 4mg capsules" | "Trandolapril" | "4.000mg" | "Capsule" | "Oral" | "2050501" |
| "998641000033111" | "140511000001108" | "Odrik 2mg capsules (Aventis Pharma)" | "Odrik 2mg capsules" | "Trandolapril" | "2.000mg" | "Capsule" | "Oral" | "" |
| "998741000033119" | "227511000001106" | "Odrik 500microgram capsules (Aventis Pharma)" | "Odrik 500microgram capsules" | "Trandolapril" | "500.000microgram" | "Capsule" | "Oral" | "" |
| "648541000033117" | "253511000001102" | "Gopten 500microgram capsules (Abbott Laboratories Ltd)" | "Gopten 500microgram capsules" | "Trandolapril" | "500.000microgram" | "Capsule" | "Oral" | "" |
| "648441000033118" | "273111000001109" | "Gopten 2mg capsules (Abbott Laboratories Ltd)" | "Gopten 2mg capsules" | "Trandolapril" | "2.000mg" | "Capsule" | "Oral" | "" |
| "648341000033112" | "346811000001101" | "Gopten 1mg capsules (Abbott Laboratories Ltd)" | "Gopten 1mg capsules" | "Trandolapril" | "1.000mg" | "Capsule" | "Oral" | "" |
| "998541000033110" | "432911000001102" | "Odrik 1mg capsules (Aventis Pharma)" | "Odrik 1mg capsules" | "Trandolapril" | "1.000mg" | "Capsule" | "Oral" | "" |
| "3014841000033118" | "5651611000001103" | "Gopten 4mg capsules (Abbott Laboratories Ltd)" | "Gopten 4mg capsules" | "Trandolapril" | "4.000mg" | "Capsule" | "Oral" | "" |
| "258441000033119" | "318915008" | "Cilazapril 500microgram tablets" | "Cilazapril 500microgram tablets" | "Cilazapril monohydrate" | "500.000microgram" | "Tablet" | "Oral" | "" |
| "258141000033110" | "318916009" | "Cilazapril 1mg tablets" | "Cilazapril 1mg tablets" | "Cilazapril monohydrate" | "1.000mg" | "Tablet" | "Oral" | "" |
| "258241000033115" | "318917000" | "Cilazapril 2.5mg tablets" | "Cilazapril 2.5mg tablets" | "Cilazapril monohydrate" | "2.500mg" | "Tablet" | "Oral" | "" |
| "258641000033117" | "318923005" | "Cilazapril 5mg tablets" | "Cilazapril 5mg tablets" | "Cilazapril monohydrate" | "5.000mg" | "Tablet" | "Oral" | "" |
[truncated: 136,187 more chars]
